# Observation of shuttling on the one-second timescale in a [10]cycloparaphenylene/C_60_ [2]catenane

**DOI:** 10.1039/d5sc05734e

**Published:** 2025-10-03

**Authors:** Fabian M. Steudel, Clara Sabrià, Massimo Delle Piane, Ferran Feixas, Xavi Ribas, Giovanni M. Pavan, Max von Delius

**Affiliations:** a Institute of Organic Chemistry and Center for Integrated Quantum Science and Technology, University of Ulm Albert-Einstein-Allee 11 89081 Ulm Germany max.vondelius@uni-ulm.de; b Institut de Química Computacional i Catàlisi, Universitat de Girona C/M. Aurèlia Capmany 69 17003 Girona Catalonia Spain; c Department of Applied Science and Technology, Politecnico di Torino Corso Duca degli Abruzzi, 24 10129 Torino Italy

## Abstract

[2]Catenanes comprising two identical binding sites are an excellent platform to study the kinetics of non-covalent interactions. In this work, we show that the “shuttling” of the [10]CPP nanohoop between two identical fullerene bis-adduct binding sites occurs with regioisomer-dependent rates of 1–5 s^−1^ at room temperature, placing these among the slowest π–π and dispersion-based shuttling processes reported to date. The catenanes were accessed *via* Glaser–Eglinton macrocyclization from fullerene bis-adduct precursors, which were purified by extensive recycling chromatography, and characterized by variable-temperature ^1^H NMR spectroscopy. Molecular dynamic simulations employing well-tempered metadynamics closely reproduce the experimental activation barrier (Δ*G*^‡^*ca.* 70 kJ mol^−1^), offering insight into the nanohoop's motion and metastable states along the shuttling pathway. The kinetic data were further complemented by thermodynamic binding studies between [10]CPP and different fullerene bis-adduct regioisomers. These findings expand our understanding of the kinetics and thermodynamics of concave/convex π–π interactions and will inform the design of future mechanically interlocked machines and 2D materials with slow response to external stimuli.

## Abstract 2

[2]Catenanes comprising identical binding sites are an excellent platform to study the unusually slow kinetics of concave–convex π–π interactions.

## Graphical abstract

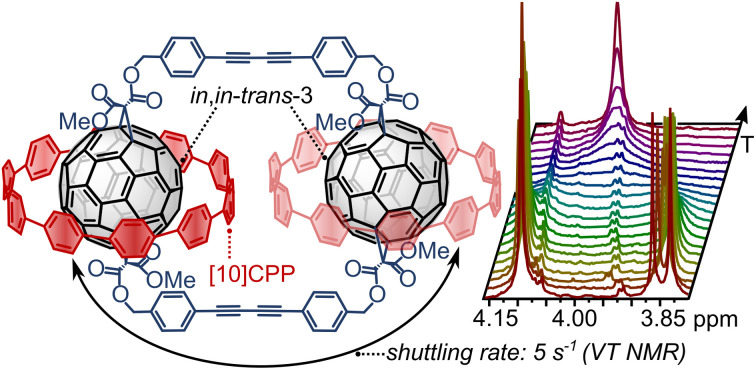

## Introduction

**Fig. 1:**
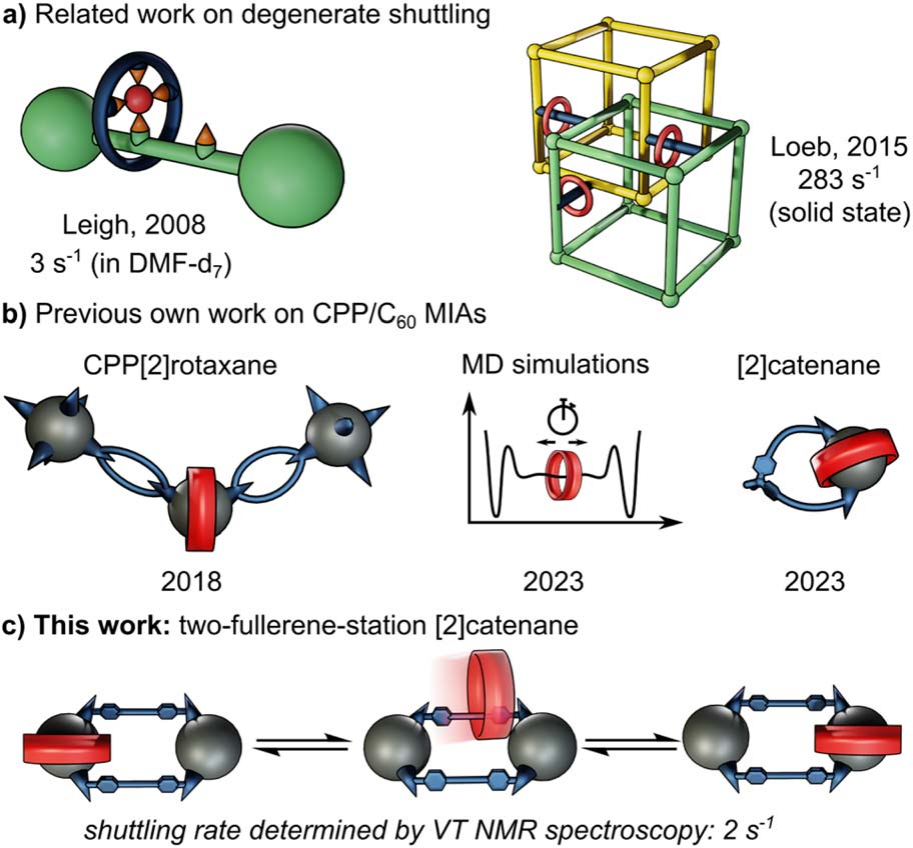
(a) Examples of relatively slow degenerate shuttling by Goldup *et al.*^9^ and Zhu *et al.*^10^ along with respective shuttling rates. (b) Relevant work by this group of authors on molecularly interlocked architectures (MIAs) comprising [10]CPP and fullerenes.^8*a*,*c*,11^ (c) Illustration of degenerate shuttling in MIM investigated in this work.

Mechanically interlocked molecules (MIMs) such as rotaxanes and catenanes have provided fundamental insights into molecular motion, particularly through the study of shuttling of macrocycles between suitable recognition sites.^1^ In degenerate molecular shuttles, where the binding sites are identical, shuttling rates have been shown to vary widely and are typically correlated with the strength of the non-covalent interactions involved.^2^ For this reason, “slow” exchange kinetics on the timescale of seconds are rare in degenerate mechanically interlocked architectures (MIAs, Fig. 1a). Slow threading processes are critical for the design of certain molecular machines.^3^ Stoddart's “molecular pumps” for instance require “valves” that operate on the timescale of minutes.^4^ This is often achieved by adding sterically demanding “speed bumps” in order to slow down the ring movement.^5^

The radially conjugated macrocycle [10]cycloparaphenylene ([10]CPP) has emerged as a highly effective host for C_60_, with near-perfect size and shape complementarity. Since the first reports of this host-guest interaction by Yamago and Jasti,^6^ the thermodynamics of similar CPP/fullerene systems have been investigated in much detail.^7^ [10]CPP/C_60_ complexes have also been employed as supramolecular templates for selective fullerene bis-functionalisation and the construction of interlocked structures.^8^

Despite these advances, the kinetics of [10]CPP complexation with fullerene bis-adducts, and how they relate to the binding affinities of different regioisomers, have not yet been experimentally explored. Earlier work only informed on the threading kinetics in pseudo-rotaxane architectures,^8*a*^ was performed *in silico*^8*c*,11^ or was unsuitable to give experimental insights into binding kinetics due to the lack of a second unoccupied binding site (Fig. 1b).^8*a*,*c*^

Herein we describe the synthesis and purification of a degenerate molecular shuttle comprising two single-isomer C_60_ bis-adduct binding sites and one [10]CPP ring, made possible by extensive use of recycling HPLC and GPC techniques that were critical for the isolation of the fullerene bis-adduct precursors. This unique molecular design allowed us to determine a degenerate shuttling rate of *ca.* 2 s^−1^ by VT-NMR spectroscopy.

## Results and discussion

### Synthesis of C_60_ bis-adduct precursors and [2]catenanes

**Fig. 2:**
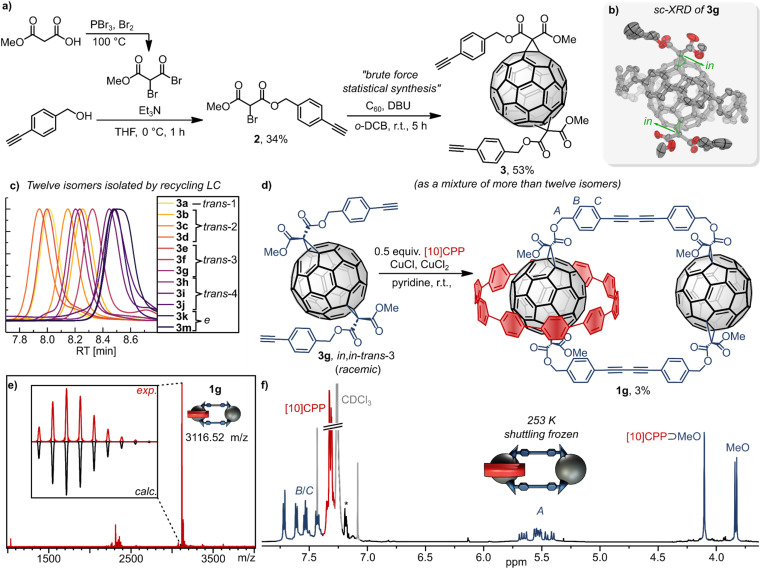
(a) Synthesis of fullerene bis-adduct precursors. (b) Single-crystal X-ray diffraction (sc-XRD) structure of one enantiomer in a [10]CPP co-crystal of the *in*,*in-trans*-3 bis-adduct 3g (thermal ellipsoids shown at the 50% probability level). (c) Normalised HPLC chromatogram stack plot of the twelve isolated bis-adducts 3a–m (Buckyprep-M, toluene, 0.5 mL min^−1^, 40 °C). (d) Synthesis of [2]catenane 1g from bis-adduct 3g. (e) MALDI mass spectrum (matrix: DCTB) and (f) ^1^H NMR spectrum (600 MHz, CDCl_3_, 253 K) of [2]catenane 1g, showing experimental and calculated isotopic patterns and diagnostic proton signals. *Toluene.

Our strategy to prepare the desired degenerate [2]catenane 1g was based on two key steps: (i) a statistical Bingel cyclopropanation reaction, giving rise to a mixture of fullerene bis-adducts (for a detailed discussion, see SI). This statistical synthesis was chosen, because our “Russian Doll” template approach, while perfectly *trans*-3-selective for symmetric malonates,^8*b*^ does not sufficiently differentiate the three *trans*-3 “*in*,*out*” diastereomers resulting from unsymmetric malonates.^8*c*,12^ (ii) Glaser–Eglinton oxidation to close the large ring in the presence of a suitable amount of [10]CPP to covalently capture the nanohoop (Fig. 2d).

We synthesised bromomalonate precursor 2 from 4-ethynylbenzyl alcohol and methyl hydrogen malonate. The malonate was first converted to methyl 2-bromomalonic acid bromide *via* Hell–Volhard–Zelinsky bromination, then esterified with the alcohol to afford methyl (4-ethynylbenzyl) bromomalonate 2. We chose methyl (as opposed to ethyl) esters on the malonate to simplify ^1^H NMR spectroscopic analysis (incl. the key VT NMR experiments) and to optimize chances for chromatographic separation of fullerene regioisomers. Compound 2 underwent Bingel cyclopropanation with C_60_ to produce the desired bis-adducts 3 bearing terminal alkynes (Fig. 2a).

The mixture of fullerene regioisomers^13^ was separated using recycling chromatography (HPLC and where necessary GPC, see SI) to afford twelve predominantly pure isomers 3a–m (Fig. 2c and Scheme S1). Minor cross-contamination by other regioisomers was detected in several cases (purity was assessed by NMR spectroscopy and HPLC). However, these small impurities are not expected to influence the outcome of the subsequent coupling reactions and the key isomer in this work (3g) was found to be 95% pure by HPLC. Regioisomer assignment was based on characteristic features in the fingerprint region of the UV-vis absorption spectra.^13*b*^ Assignment of the *in*,*out* isomerism^8*c*,12^ proved particularly challenging. The *C*_1_ symmetric *in*,*out* isomers were identified by their reduced symmetry, reflected in the number of signals in their ^1^H and ^13^C NMR spectra. Among the *C*_2_ symmetric isomers, only the *in*,*in-trans*-3 bis-adduct 3g could be unambiguously assigned through single-crystal X-ray diffraction (sc-XRD) (Fig. 2b). With isomers 3g (*in*,*in-trans-*3) and 3e (*in*,*out-trans*-3) identified, the *out*,*out-trans*-3 isomer 3f was assigned by exclusion.

Glaser–Eglinton^14^ coupling of the *trans*-3 isomer 3g at 0.35 mM concentration in the presence of 0.5 equivalents of [10]CPP yielded the desired [2]catenane 1g in 3% isolated yield. The low yield is a consequence of the occurrence of side reactions such as homocoupling between two [10]CPP-complexed or two uncomplexed bis-adducts, as well as oligomerization and formation of bigger cycles. Moreover, since two inherently chiral *trans*-3 bis-adducts (from a racemic mixture) were formed, [2]catenane 1g was obtained as a mixture of diastereomers, as evident from (fine) splitting of the methyl ester signals in the ^1^H NMR spectrum (Fig. 2d, 3.84 and 3.82 ppm). The most important signal splitting at low temperature (*e.g.* 253 K) is, however, the one into one set of signals for the [10]CPP-complexed (4.10 ppm) and one set of signals for the uncomplexed malonate (3.84 and 3.82 ppm).

Significant isomer-dependent reactivity was observed during coupling reactions starting from different regioisomers (Table 1). While the *in*,*in-trans*-3 isomer 3g cleanly formed the catenane, the *in*,*out*-trans-3 isomer 3e only gave trace amounts of [2]catenane (MALDI-MS). Instead, the main product was macrocycle 4e, formed *via* intramolecular Glaser coupling, in 15% yield.

**Table 1:** Yields for the formation of [2]catenanes 1 and/or intramolecular macrocycles 4 derived from all isolated *trans*-1 to *trans*-3 bis-adducts 3 (see Scheme S1 for overview of isomers)

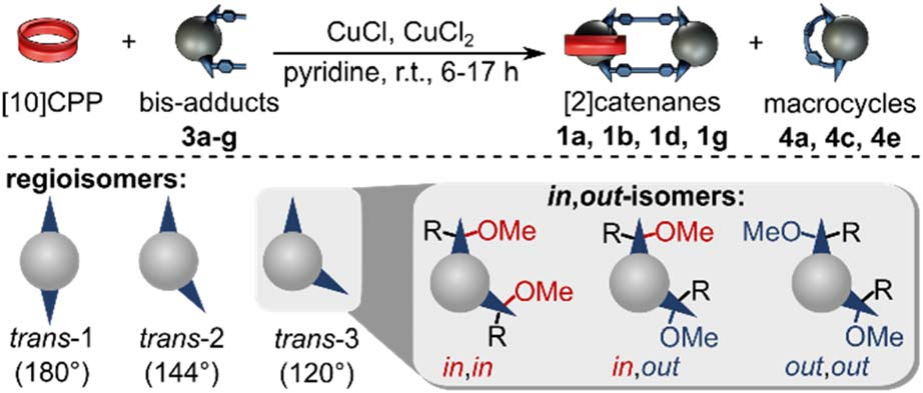		
Bis-adduct	Isomer	Isolated species (NMR yields)
3a	*out*,*out-trans*-1	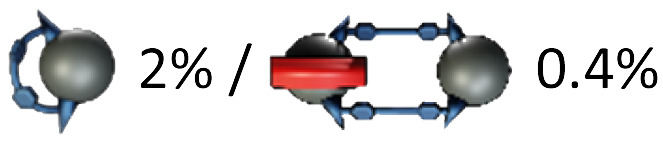
3b	*in*,*out-trans*-2	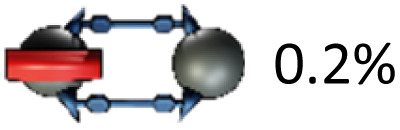 a
3c	*out*,*out-trans*-2	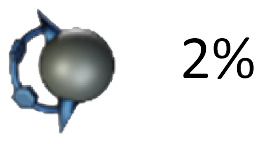
3d	*in*,*in-trans*-2	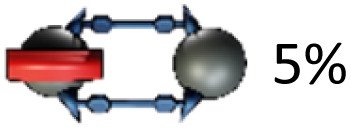
3e	*in*,*out-trans*-3	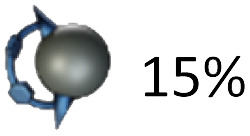 b
3f	*out*,*out-trans*-3	n.a.
3g	*in*,*in-trans*-3	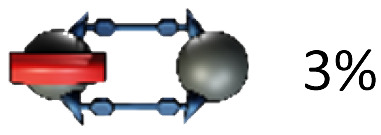

a Only one of two isomers isolated.

b Isolated yield by weight.

This observation prompted us to investigate catenane formation across all isolated *trans*-1, *trans*-2, and *trans*-3 isomers. Overall, [2]catenane formation was observed only for four isomers (3a, 3b, 3d, 3g), with isolated yields ranging from 0.2 to 5% (Table S1, determined by quantitative NMR). Intramolecular macrocyclisation rather than dimerisation occurred in three isomers (3a, 3c, 3e), with isomer 3a yielding both the [2]catenane 1a (0.4%) and macrocycle 4a (2%). The *out*,*out-trans*-3 isomer 3f did not yield any isolable products in sufficient quantity for NMR characterisation. Sc-XRD structures of macrocycles 4a (Fig. S54) and 4c (Fig. S55) enabled the unambiguous assignment of the *out,out-trans*-1 (4a) and *out*,*out-trans*-2 (4c) structure to these compounds and their bis-adduct precursors (3a and 3c). By exclusion, we were able to assign compounds 3d and 1d as the *in*,*in-trans*-2 bis-adduct and the respective (*in*,*in-trans*-2)_2_ [2]catenane. Notably, the sc-XRD structures of 4a and 4c revealed short contacts between the curved 1,4-diphenylbutadiene tether and C_60_, (Fig S54 and S55) indicative of attractive concave–convex π–π interactions. These interactions may contribute to the stabilization of the macrocyclic side-products and could play a role in promoting their formation from certain isomers. We conclude that only a few of the isolated bis-adducts possess a geometry suitable for [2]catenane formation. Our results furthermore suggest that the *in*,*out* isomerism (*e.g. in*,*out vs. in*,*in*) affects catenane formation more profoundly than regioisomerism (*e.g. trans*-2 *vs. trans*-3).

### VT NMR spectroscopic study on degenerate shuttling

**Fig. 3:**
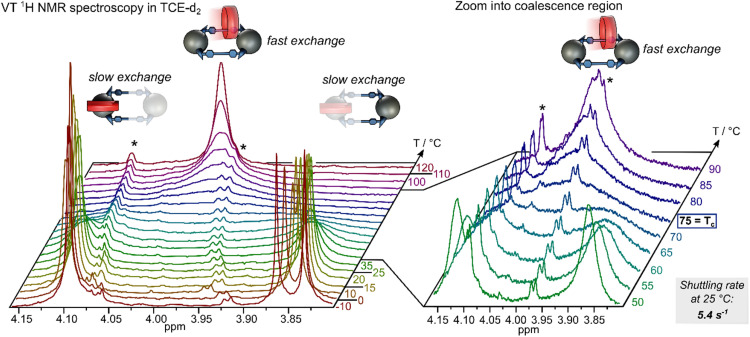
VT ^1^H NMR (600 MHz, C_2_Cl_4_D_2_) of the methyl ester region of [2]catenane 1g recorded from −10 to 120 °C, including a “zoom“ into the coalescence region. *Residual, unknown impurity after HPLC purification (note: in some VT-NMR spectra, the impurity appears artificially intense due to broadening of the [2]catenane signals).

Having synthesized a variety of [2]catenanes, we turned our attention to studying the shuttling behaviour of [10]CPP between two fullerene-bis-adduct binding sites using variable-temperature VT ^1^H NMR spectroscopy. Initial experiments were conducted in CDCl_3_, however coalescence of the methyl ester proton signals was not observed within the accessible temperature window (up to 323 K). Due to their large difference in chemical shift (4.1 *vs.* 3.8 ppm), these methyl protons are ideally suited to study nanohoop shuttling. Turning to tetrachloroethane-d_2_ (TCE-d_2_)^15^ we were able to record ^1^H NMR spectra up to 393 K. Under these conditions, coalescence of the methyl ester resonances corresponding to the [10]CPP-bound and unbound fullerene bis-adducts was observed at 348 K (75 °C, Fig. 3). From the frequency separation between the exchanging peaks (154 Hz) we were able to determine a Gibbs free energy of activation (Δ*G*^‡^) of 68.8 kJ mol^−1^ (see SI for details and experimental uncertainty), corresponding to a shuttling rate *k* of 5.4 s^−1^ (Table 2) at room temperature.^16^

**Table 2:** Shuttling rates determined by VT ^1^H NMR spectroscopy for fullerene bis-adduct (3) and [10]CPP mixtures (2 : 1) and [2]catenanes (1)

Isomer	Type (catenane *vs.* complex^a^)	Solvent	*k* [s^−1^]^b^
*out*,*out-trans*-1	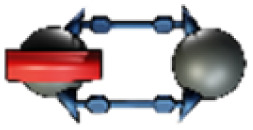	TCE-d_2_	<5.9^c^
*in*,*out-trans*-2	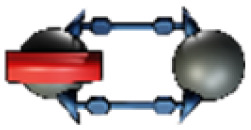	TCE-d_2_	2.4
*in*,*in-trans*-2	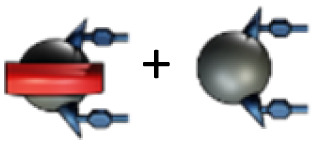	CDCl_3_	26
*in*,*in-trans*-2	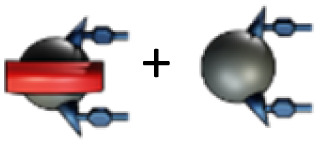	TCE-d_2_	8.3
*in*,*in-trans*-2	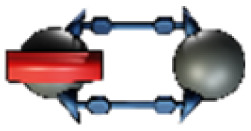	TCE-d_2_	1.3
*in*,*in-trans*-3	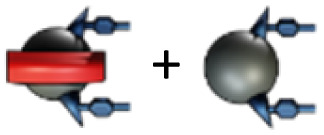	CDCl_3_	44
*in*,*in-trans*-3	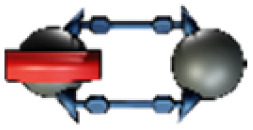	TCE-d_2_	5.4

a 2 : 1 mixture of bis-adduct with [10]CPP.

b See SI for experimental uncertainty (eqn (S3)–(S6), and Table S2).

c Exact shuttling rate could not be determined due to signal loss at elevated temperature from low sample concentration. The value is given as <5.9 s^−1^ based on the highest temperature at which coalescence was not yet observed.

Interestingly, a control experiment with the simple host-guest complex of the *in*,*in-trans*-3 fullerene bis-adduct with [10]CPP (2 : 1 ratio) had revealed a coalescence of the same methyl ester protons at 313 K (Fig. S41), corresponding to a shuttling rate at room temperature of 44 s^−1^, indicating that shuttling is significantly slower in the catenane as in this reference system. Tethering the bis-adduct moieties appears to increase the kinetic barrier for [10]CPP to slip off the fullerene binding site.

We hypothesize that this behaviour arises from our design that features a rigid linker between the two fullerene units. The rigidity likely prevents the system from adopting a more favourable conformation during the slip-off motion of [10]CPP, effectively hindering the exchange process.

To assess the impact of the solvent on the shuttling kinetics, we investigated the *in*,*in-trans*-2 bis-adduct 3d in both CDCl_3_ and TCE-d_2_, observing a rate of 26 s^−1^ (Δ*G*^‡^ = 64.9 kJ mol^−1^) in CDCl_3_ and a slower rate of 8.3 s^−1^ (Δ*G*^‡^ = 67.7 kJ mol^−1^) in TCE-d_2_ (Table 1). The corresponding [2]catenane 1d, derived from this bis-adduct mixture, showed coalescence at 358 K, with a Gibbs free energy barrier of 72.3 kJ mol^−1^ and a shuttling rate of 1.3 s^−1^. In this example, the change of solvent accounts for an increase of 2.8 kJ mol^−1^, while transformation into the catenane architecture adds a further 4.6 kJ mol^−1^ to the kinetic barrier. The higher Δ*G*^‡^ observed for the *trans*-2 catenane 1d compared to the *trans*-3 catenane 1g correlates with the stronger binding affinity of the *trans*-2 isomers toward [10]CPP (see Fig. 5a and Table S3 for *K*_a_ values). Overall, our comparison of isomers suggests that the shuttling barriers are influenced by the regioisomerism as well as steric constraints associated with the *in*,*out*-configuration, with the slowest shuttling rate measured for the (*in*,*in-trans*-2)_2_ [2]catenane 1d.

The observed shuttling rates of *ca.* 2 s^−1^ place our [2]catenanes 1b and 1d among the slowest degenerate molecular shuttles reported to date for systems relying solely on dispersion and π–π interactions, complementing previous examples by Ogoshi, Nierengarten and Stoddart.^17^

### Computational insights

**Fig. 4:**
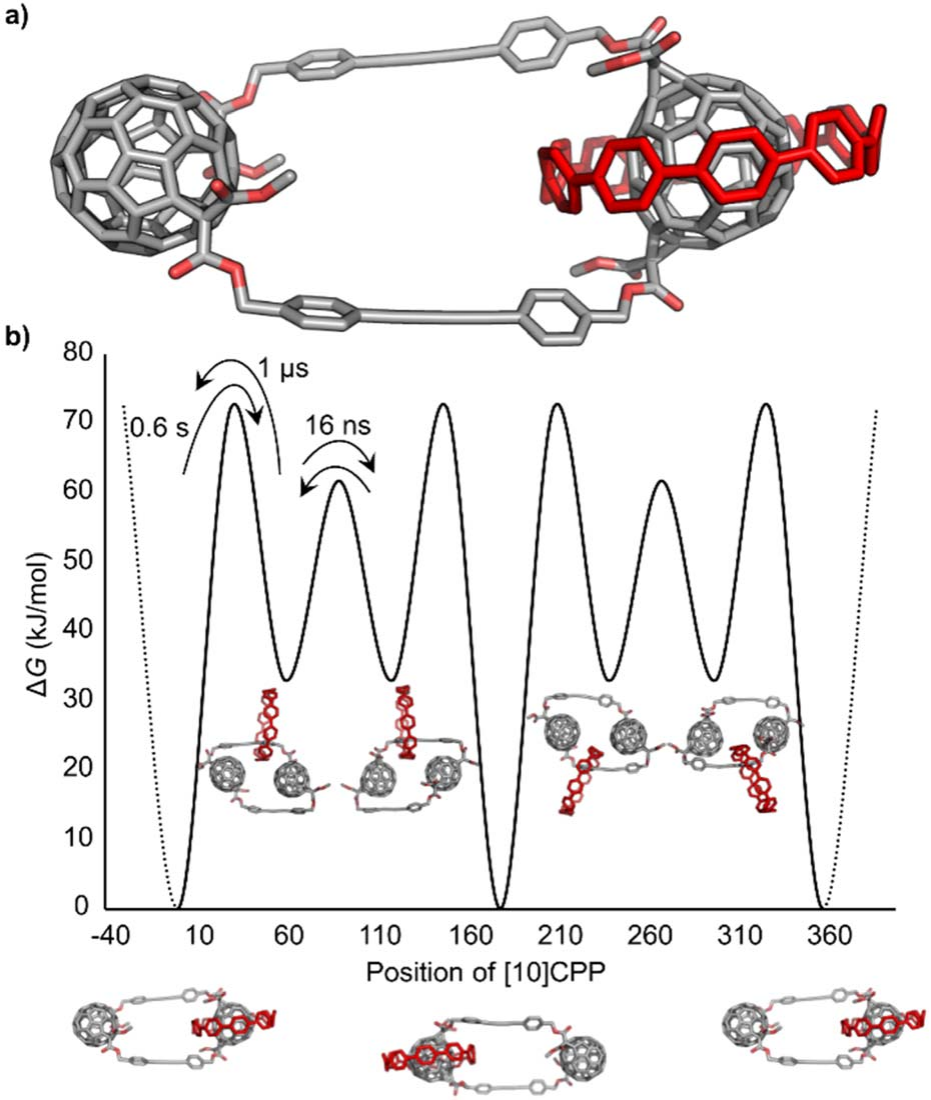
(a) Atomistic model of the (*in*,*in-trans-3*)_2_ [2]catenane 1g with [10]CPP shown in red. (b) Free energy surface (FES) as a function of the angular position of [10]CPP along the C_60_ based chain. Transition times and Δ*G* differences (in kJ mol^−1^) between metastable states can be observed.

Molecular simulations have been performed to gain insights into the dynamics of the [2]catenane 1g ((*in*,*in-trans-3*)_2_ isomer). In particular, the translation of the [10]CPP around the chain to go from one C_60_ station to the other has been studied. The use of classical molecular dynamics (MD) with combination of infrequent well-tempered metadynamics (WT-MetaD) allows the study of this dynamical behaviour. The model of [2]catenane 1g (Fig. 4a) was obtained and chloroform was used as solvent (see SI for details).

First, classical MD simulations were carried out, with the [10]CPP binding one C_60_ position as starting point. In this case, the unbinding and translation of [10]CPP cannot be observed, since this process requires crossing a large free-energy barrier. For this reason, WT-MetaD were used to be able to study the kinetics and thermodynamics of this process (SI for details).

Since the [2]catenane 1g is symmetric, the path through the top chain is equal as going through the bottom one. It was hypothesized that different metastable states were present in the path going from one C_60_ position to the other, since it was observed that the nanohoop can be stabilized by simultaneously binding the phenyl rings of two different linkers. The first process involved is the unbinding of the [10]CPP from the C_60_, which presents a characteristic time of ≈0.6 s with a kinetic barrier of ≈72.4 kJ mol^−1^. These results are in good agreement with the value obtained in the VT ^1^H NMR experiments (68.8 kJ mol^−1^) and also are quite similar to the previously reported work on a C_60_/[10]CPP-catenane.^8*c*^ This large energetic barrier is due to the interruption of concave–convex π–π interactions between the nanoring and the fullerene. The second process involves the translation of [10]CPP from one phenyl ring position to the other of the same chain. This is the fastest process, with a transition time of ≈16 ns and a kinetic barrier of ≈28.9 kJ mol^−1^. The last process is the inverse of the first one, the binding of [10]CPP to C_60_. In this case, the time scale of the transition is smaller as expected, ≈1 μs with an energetic barrier of ≈39.3 kJ mol^−1^, since the nanoring prefers to bind the C_60_. The [10]CPP translation is clearly dictated by the binding/unbinding with the C_60_ positions, and the interaction with the phenyl rings on the chains is relatively weak (see the free-energy landscape in Fig. 4b and the SI Video S1).

## Association constants

With valuable kinetics data in hand for various C_60_ bis-adduct regioisomers, we decided to also investigate their thermodynamics, for which only fragmented data exists^8*a*^ and a complete picture is still missing. This data will be valuable for potential uses of these motifs as “speed bumps” as the slow kinetics reported above are not due to steric bulk but due to decomplexation kinetics which is linked with the strong non-covalent interaction between shape-complementary (convex–concave) building blocks. To systematically investigate the influence of regioisomerism and steric demand of the addends on the association constants of bis-adducts with [10]CPP, we selected dimethyl malonate C_60_ bis-adducts 5 and diethyl malonate bis-adducts 6 as model systems. Bis-adduct 5 serves as a simplified analogue of our target [2]catenanes, which feature unsymmetrical malonate addends bearing methyl groups on one side, whereas bis-adduct 6, featuring bulkier ethyl groups, represents an increase in steric demand similar to that imposed by the 4-ethynylbenzyl residue present on the opposite side of our malonates in bis-adducts 3. Compounds 5 and 6 were synthesized according to literature procedures,^8*c*,18^ and individual regioisomers were isolated by preparative thin-layer chromatography.

**Fig. 5:**
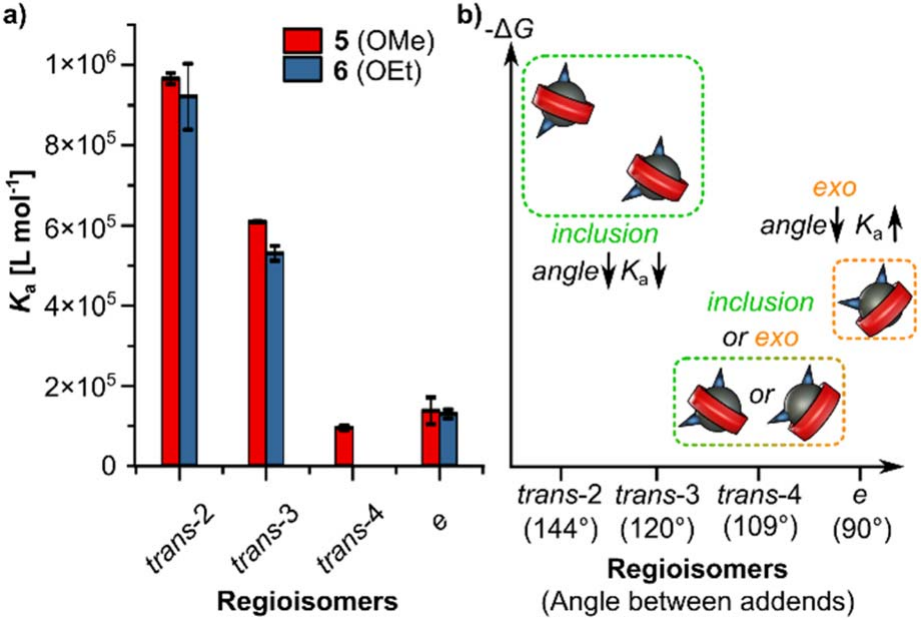
(a) Plot of average association constants *K*_a_ (Table S3) for [10]CPP binding to fullerene bis-adducts 5 and 6 as determined by fluorescence quenching titrations (1 : 1 model, Nelder–Mead fit, supramolecular.org).^19^ Error bars represent the standard deviation from triplicate measurements. (b) Conceptual chart illustrating the relative Δ*G* changes associated with complexation by [10]CPP, suggesting a transition in binding mode around the *trans*-4 regioisomer from an inclusion complex to an “*exo*” complex. The model relates the angle between addends to the favorability of complexation: for inclusion complexes, lower angles correspond to less favorable (higher) Δ*G*, whereas for *exo* complexes, lower angles are expected to result in more favorable (lower) Δ*G*.

While the *trans*-1 isomers of 5 and 6, as well as *trans*-4 of 6, could not be obtained in sufficient purity and quantity, all other *trans*-2 to *e* isomers were successfully isolated and used in the titration experiments. Fluorescence quenching was performed in triplicate by titrating a 57.5 μM solution of each bis-adduct into a 0.30 μM solution of [10]CPP in toluene, up to a total of 80 equivalents. Association constants (Fig. 5a, and Table S3) were extracted by fitting the quenching data to a 1 : 1 binding model using the Nelder–Mead optimization method, as implemented *via* supramolecular.org.^19^

As expected, we observed higher association constants for the *trans*-2 regioisomers compared to the *trans*-3 isomers, in agreement with previously reported data.^8*a*^ For the *trans*-4 regioisomer of 5, a tenfold decrease in the binding constant was observed, which increased slightly for the equatorial isomers. This particularly low *K*_a_ for the *trans*-4 isomer can be attributed to increased steric congestion between the two addends, which likely leads to a change in the binding mode. While *trans*-2 and *trans*-3 isomers lead to an inclusion complex (see Fig. 2b),^8*a*–*d*^ where the [10]CPP ring is threaded over the fullerene with the addends on opposite sides of the nanohoop, the *trans*-4 isomer may favor an *exo* binding mode, in which both addends are located on one side of the CPP cavity. Such a shift in complexation geometry could explain both the sharp decrease in association constant for the *trans*-4 isomer and the increase observed for the equatorial isomer, where the 90° angle between addends leads to less steric hindrance than the 109° separation in the *trans*-4 isomer, making *exo* complexation more favorable. Furthermore, we generally observed higher binding constants for the dimethyl bis-adducts 5 compared to the diethyl analogues 6, highlighting the impact of steric bulk. Notably, in the case of mono-adducts, this steric effect is less pronounced, with previous reports showing similar association constants for the dimethyl and diethyl malonate mono-adducts.^20^ A comparison of these association constants with the previously discussed shuttling rates (Tables 2 and S2) suggests a clear correlation, where stronger binding translates to slower exchange. Interestingly, the shuttling data also reveals a trend within individual regioisomers, with exchange rates increasing from *in*,*in*-over *in*,*out*-to the *out*,*out*-configuration. This trend may be rationalized by steric considerations: in the *in*-configuration, the smaller methyl ester residues are positioned closer to the equatorial region of the fullerene—the preferred binding site of [10]CPP—where reduced steric congestion could favor stronger interactions. Conversely, in the *out*-configuration, bulkier substituents are expected to increase steric congestion in this region, which may weaken association and facilitate faster shuttling. Together with our kinetic studies, these thermodynamic insights reveal design principles for tuning both association strength and exchange dynamics in nanohoop–fullerene systems.

## Conclusions

We report the design, synthesis, and dynamic characterization of a family of [2]catenanes in which one [10]cycloparaphenylene ([10]CPP) ring is shuttling between two fullerene bis-adduct binding sites. To the best of our knowledge, this is the first example of a [10]CPP/C_60_ MIM with a stoichiometry differing from 1 : 1 (ring/binding site). Variable-temperature ^1^H NMR spectroscopy revealed degenerate shuttling on the one-second timescale at room temperature, with Δ*G*^*‡*^ values around 70 kJ mol^−1^—placing these systems among the slowest reported for shuttles based solely on π–π and dispersion interactions. Molecular dynamics simulations support the experimental findings by reproducing the relevant energy barriers and timescales. Importantly, the origin of the slow shuttling lies in the slow decomplexation kinetics of the [10]CPP/C_60_ binding sites rather than in steric “speed bumps,” highlighting a distinct mechanism of motion control. Our findings establish the [10]CPP/C_60_ motif as a viable platform for realizing slow exchange in mechanically interlocked molecular machines and 2D materials^21^ with unique mechanical^22^ and optoelectronic properties.^23^

## Author contributions

F. M. S. synthesized, purified and characterised all compounds. C. S. contributed the molecular dynamics simulations under the supervision of M. Delle P., F. F., X. R. and G. M. P. The project was directed by M. v. D. and the manuscript (including the ESI) was written jointly by all authors.

## Conflicts of interest

There are no conflicts to declare.

## Supplementary Material

SC-016-D5SC05734E-s001

SC-016-D5SC05734E-s002

SC-016-D5SC05734E-s003

## Data Availability

Synthesis and characterization data supporting this article have been included as part of the SI. Host–guest titration data is available *via* supramolecular.org (links in the SI).^19^ Computational data is available *via* Zenodo under the following link: https://doi.org/10.5281/zenodo.17093025. CCDC 2448364 (4a), 2448366 (4c) and 2448367 (3g/[10]CPP) contain the supplementary crystallographic data for this paper.^24*a*–*c*^ Supplementary information: experimental and computational details, spectroscopic data, X-ray structures, and the synthesis of a C_70_–C_60_ dyad using a sequential Bingel followed by Bingel–Hirsch approach originally explored for [2]catenane synthesis. See DOI: https://doi.org/10.1039/d5sc05734e.
