# Supplementary material for: Observation of shuttling on the one-second timescale in a [10]cycloparaphenylene/C60 [2]catenane
Source: Chem Sci. 2025 Oct 3;16(46):21908–16. doi: 10.1039/d5sc05734e (PMC12532037; doi:10.1039/d5sc05734e)
Supplement: SC-016-D5SC05734E-s002 [file SC-016-D5SC05734E-s002.pdf]

Supplementary Information (SI) for Chemical Science.
This journal is © The Royal Society of Chemistry 2025

Supporting Information

Observation of shuttling on the one-second timescale in a [10]cycloparaphenylene/C_{60} [2]catenane

Fabian M. Steudel,[a] Clara Sabrià,[b,d] Massimo delle Piane,[d] Ferran Feixas,[b] Xavi Ribas,[b] Giovanni M. Pavan,[c,d] Max von Delius*[a]

[a]Institute of Organic Chemistry I, University of Ulm, Albert-Einstein-Allee 11,89081 Ulm (Germany)

[b]Institut de Química Computacional i Catàlisi, Universitat de Girona C/M. Aurèlia Capmany 69, 17003 Girona, Catalonia (Spain)

[c]Department of Innovative Technologies, University of Applied Sciences and Arts of Southern Switzerland, Polo Universitario Lugano, Campus Est Via la Santa 1, 6962 Lugano-Viganello (Switzerland)

[d]Department of Applied Science and Technology, Politecnico di Torino Corso Duca degli Abruzzi, 24, 10129 Torino (Italy)

Email: max.vondelius@uni-ulm.de

Content

1. General Experimental Section 3

1.1. General information 3

1.2. General analytical information 3

2. Synthetic Procedures and Characterization data 5

2.1. Synthesis of the bromomalonate and fullerene bis-adducts 5

2.2. Separation and analysis of fullerene bis-adducts **3** 11

2.2.1. Preparative LC separation of the isomers 11

2.2.2. Isomeric assignment and spectroscopic data 15

2.3. Synthesis of the [2]catenanes 28

2.4. Sequential synthesis of a C_{60}–C_{70} dyad *via* Bingel and Bingel–Hirsch reactions 38

3. NMR Studies on CPP Shuttling 54

4. Fluorescence Quenching Titrations 64

5. Crystallographic Data 73

6. Computational Data 83

6.1. Kinetics 83

7. References 85

8. Spectra 87

1. General Experimental Section

1.1. General information

All commercially available reagents were purchased from Sigma Aldrich, TCI, BLD Pharm, Fisher Scientific, Alfa Aesar, VWR International, Carl Roth, Acros Organics and Merck, and were used without further purification if not stated otherwise. Anhydrous dichloromethane, toluene, *N*,*N*-dimethylformamide, tetrahydrofuran and diethyl ether were dried prior to use in a MBraun SPS-800 instrument, others anhydrous solvents were bought. Copper(I) chloride was refluxed in concentrated hydrochloric acid, precipitated by dilution with water, filtered and dried under vacuum before being transferred into a MBraun glovebox.

1.2. General analytical information

NMR spectra were recorded on Bruker AVANCE 400, 500, and 600 MHz instruments. Chemical shifts (δ) are reported in ppm relative to residual solvent peaks: $CDCl_3$ (δ_H = 7.26, δ_C = 77.16 ppm), CD_2Cl_2 (δ_H = 5.32, δ_C = 53.84 ppm), DMSO-d_6 (δ_H = 2.50, δ_C = 39.52 ppm), and 1,1,2,2-tetrachloroethane-d_2 (δ_H = 6.00). Splitting patterns are designated as follows: s (singlet), d (doublet), t (triplet), q (quartet), m (multiplet) and AB for AB spin systems. Coupling constants *J* relate to proton-proton couplings.

High-resolution mass spectra (HRMS) were recorded on a Bruker SolariX FTICR mass spectrometer using either MALDI or APCI ionization. MALDI-FTICR analyses were performed with DCTB (trans-2-[3-(4-tert-butylphenyl)-2-methyl-2-propenylidene]malononitrile) as matrix. APCI-FTICR spectra were acquired using acetonitrile as solvent. MALDI-TOF measurements were performed on a Bruker Ultraflex MALDI-TOF/TOF instrument, also using DCTB as matrix.

Normal-phase flash column chromatography was performed manually using Machery-Nagel silica 60 or automatically on a Büchi FlashPrep C-810 using EcoFlex silica columns (40-63 µm particle size for both). Thin-layer chromatography (TLC) was performed using TLC

sheets with a particle size of 60 µm. Spots were detected using UV light of 254 and 365 nm wavelengths.

Preparative HPLC was performed in higher milligram scale on preparative Shimadzu CBM-20A with an SPD-20A UV-Vis detector and a LC-81 pump on a Machery-Nagel VP 250/1 Nucleosil 100-5 silica column. Recycling gel-permeation chromatography was performed on a JAI LaboACE LC-7080 Plus equipped with a JAIGEL-2.5HR and 2HR columns. Compounds were detected by UV-Vis at 254 nm and 300 nm when using DCM or toluene, respectively. Preparative HPLC on the lower milligram scale and analytical HPLC were performed on a Shimadzu CBM-20A equipped with two LC-20AD pumps, a SIL-20A autosampler, an SPD-M20A photodiodearray detector module and an FRC-10A fraction collector on a Cosmosil Buckyprep-M (4.6x250mm) column.

Bulb-to-bulb distillation was performed using a BÜCHI GKR-50 glastube oven for mikrodistillation.

Fluorescence spectra were recorded on a Perkin Elmer FL6500 fluorescence spectrophotometer using HPLC grade toluene (ROTISOLV ≥99.9%) and Hellma Analytics fluorescence cuvettes (10x10 mm).

Single-crystal X-ray diffraction data for the co-crystal of [10]cycloparaphenylene and the *in*,*in-trans*-3 C_{60} bis-adduct **3g** were collected at 161 K on a Bruker D8 KAPPA diffractometer equipped with a Photon III M14 CPAD detector. A microfocus sealed tube with Cu Kα radiation (λ = 1.54178 Å) and a multilayer mirror monochromator was used as the X-ray source. Temperature control was provided by a Cryostream device. Data collection and reduction were conducted using BIS 8.5.5.5 Beta (2024-10-02). Single-crystal X-ray diffraction data for the macrocycles **4a** and **4c** obtained from intramolecular Glaser coupling of the *out*,*out-trans*-2 bisadduct **3c** and the *out*,*out-trans*-1 bis-adduct **3a** were collected at 150 K on a Rigaku SuperNova, Cu at zero, Atlas CCD using graphite-monochromated Cu K_α radiation. Data reduction and cell refinement were performed using the CrysAlisPro software.[S1] The structures were solved in Olex2 using direct methods and refined with ShelXL.[S2] All non-hydrogen atoms were refined anisotropically.

2. Synthetic Procedures and Characterization data

2.1. Synthesis of the bromomalonate and fullerene bis-adducts

Synthesis of methyl 4-ethynylbenzyl bromomalonate (2)

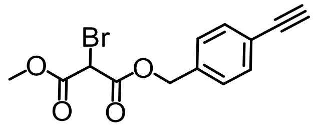

In a heat-dried 100 mL pressure tube was added bromine (1.6 mL, 5.0 g, 31 mmol, 4.5 equiv.) to a mixture of phosphorous tribromide (0.48 mL, 1.4 g, 5.0 mmol, 0.73 equiv.) and methyl hydrogen malonate (1.4 mL, 1.6 g, 12 mmol, 1.8 equiv.) at 100 °C. The reaction mixture was subsequently stirred for 4 h at 100 °C before excess bromine and hydrogen bromide were removed under reduced pressure. The residue was taken up with anhydrous (anh.) THF (10 mL) and added dropwise to a stirred solution of triethyl amine (3.0 mL, 2.2 g, 22 mmol, 2.1 equiv.) and 4-ethynylphenyl methanol (0.91 g, 6.9 mmol, 1.0 equiv.) in anh. THF (100 mL) at 0 °C. The reaction mixture was stirred for 1 h before the reaction was quenched by addition of saturated aqueous sodium thiosulfate solution and the aqueous phase was extracted with DCM (3 x 100 mL). The combined organic phases were dried over sodium sulfate, filtered, and evaporated. The product **2** was obtained 85% pure after column chromatography (silica (Büchi-Ecoflex-40g), CH→CH/EA 10:1 over 40 min) as a yellow oil (1.00 g, 3.22 mmol, 47%).

Due to a dimethyl malonate impurity in the used technical grade methyl hydrogen malonate (90% purity) the product after flash column chromatography contained roughly 15% dimethyl bromomalonate.

Therefore, a part of the product (725 mg) was further purified by bulb-to-bulb distillation (165 °C, 0.15 mbar) to yield the product as a yellow oil (532 mg, 1.71 mmol, 34%*).

*Yield after distillation calculated based on distilled fraction of the whole.

^{1}H NMR (400 MHz, $CDCl_3$, 298 K): δ = 7.54 – 7.46 (m, 2H, H_{Ar}), 7.36 – 7.28 (m, 2H, H_{Ar}), 5.25 (s, 2H, H_{Benzyl}), 4.89 (s, 1H, $H_{Bromomalonate}$), 3.81 (s, 3H, -OCH_3), 3.10 (s, 1H, $H_{Acetylene}$) ppm.

^{13}C NMR (101 MHz, $CDCl_3$, 298 K): δ = 165.0, 164.4, 135.3, 132.5, 128.2, 122.7, 83.2, 78.1, 68.2, 54.1, 41.9 ppm.

HRMS (APCI): found m/z = 310.9916; calc. for $[M+H]^+$ $C_{13}H_{12}BrO_4^+$: 310.9914; Δm/m = 0.67 ppm.

Synthesis of methyl 4-ethynylbenzyl malonate C_{60}MA (7)

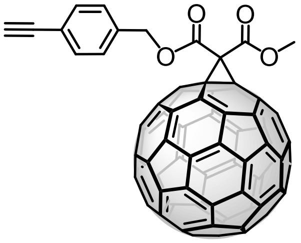

To a solution of C_{60} (0.62 g, 0.85 mmol, 1.9 equiv.) in *o*-DCB (50 mL) was added a solution of the bromomalonate **2** (0.14 g, 0.44 mmol, 1.0 equiv.) in DCM (1 mL) and the reaction was started by dropwise addition of a solution of DBU (0.80 mL, 0.82 g, 5.4 mmol, 12 equiv.) in *o*-DCB (5 mL). The reaction was stirred for 2.5 h at r.t. and subsequently purified by flash column chromatography (silica, PE/*o*-DCB 3:1 → PE → PE/EA 4:1). Product **7** was obtained as a brown solid (0.16 g, 0.17 mmol, 38%).

^{1}H NMR (600 MHz, $CDCl_3$, 298 K): δ = 7.54 (d, ^3J = 8.1 Hz, 2H, H_{Ar}), 7.46 (d, ^3J = 8.0 Hz, 2H, H_{Ar}), 5.51 (s, 2H, H_{Benzyl}), 4.02 (s, 2H, -OCH_3) ppm.

^{13}C NMR (151 MHz, $CDCl_3$, 298 K): δ = 164.1, 163.5, 145.4 (C_{60}-sp^2, 4C), 145.4 (C_{60}-sp^2, 2C), 145.4 (C_{60}-sp^2, 2C), 145.3 (C_{60}-sp^2, 2C), 145.2 (C_{60}-sp^2, 2C), 145.2 (C_{60}-sp^2, 2C), 145.1 (C_{60}-sp^2, 2C), 145.0 (C_{60}-sp^2, 2C), 144.9 (C_{60}-sp^2, 2C), 144.9 (C_{60}-sp^2, 2C), 144.8 (C_{60}-sp^2, 3C), 144.8 (C_{60}-sp^2, 2C), 144.7 (C_{60}-sp^2, 1C), 144.1 (C_{60}-sp^2, 2C), 144.0 (C_{60}-sp^2, 2C), 143.3 (C_{60}-sp^2, 2C), 143.2 (C_{60}-sp^2, 6C), 143.1 (C_{60}-sp^2, 2C), 142.4 (C_{60}-sp^2, 4C), 142.1 (C_{60}-sp^2, 2C), 142.0 (C_{60}-sp^2, 2C), 141.1 (C_{60}-sp^2, 4C), 139.6 (C_{60}-sp^2, 2C), 138.9 (C_{60}-sp^2, 2C), 135.4, 132.6, 129.0, 123.0, 83.2, 78.3, 71.5 (C_{60}-sp^3, 2C), 68.5, 54.1, 51.9 ppm.

Due to overlap, only 25 of the expected 32 C_{60} peaks were found.

HRMS (MALDI): found m/z = 950.0559; calc. for $C_{73}H_{10}O_4$: 950.0579; Δm/m = 2.1 ppm.

Synthesis of methyl 4-ethynylbenzyl malonate C_{60}BAs (3)

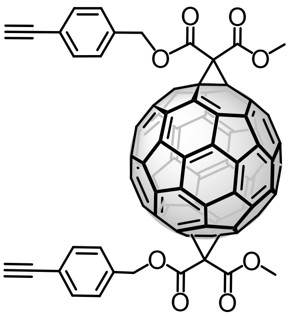

To a stirred solution of bromomalonate **2** (0.25 g, 0.81 mmol, 2.0 equiv.) and C_{60} (0.31 g, 0.43 mmol, 1.1 equiv.) in 1,2-dichlorobenzene (75 mL) was added a solution of DBU (0.20 mL, 0.20 g, 1.3 mmol, 3.3 equiv.) in 1,2-dichlorobenzene (5 mL) dropwise and the reaction mixture was stirred at r.t. for 6 h. The reaction was quenched by addition of saturated aqueous ammonium chloride solution and the aqueous phase was extracted with dichloromethane. The combined organic phases were dried over sodium sulfate, filtered and evaporated. The product **3** was obtained as a mixture of isomers after flash column chromatography (silica, PE/toluene 3:1 → DCM/PE 2:1 → DCM) and further purified by HPlC (silica, toluene, 24 mL/min, r.t.) , followed by recycling HPLC (silica, toluene, 24 mL/min, r.t., recycling mode) of each fraction. Fractions, which still lacked purity were subsequently further purified by recycling GPC (JAIGEL-2.5HR & 2HR, dichloromethane, 10 mL/min).

HRMS (MALDI): found m/z = 1180.1188; calc. for $[M]^+$ $C_{86}H_{20}O_8$: 1180.1158; Δm/m = 2.5 ppm; found m/z = 1203.10972; calc. for $[M+Na]^+$ $C_{86}H_{20}NaO_8^+$: 1203.10504; Δm/m = 3.9 ppm.

Assignment of the regioisomers was conducted by comparing the fingerprint region (400–800 nm) of their absorption spectra with literature data.[S3] The asymmetric *in,out*-diastereomers, arising from the malonate substituent asymmetry, were assigned based on ^{1}H and ^{13}C NMR spectra. In contrast, the assignment of the symmetrical *in,in*- and *out,out*-isomers remained inconclusive for the *trans*-4 and *equatorial* isomers, which were labelled sequentially as (a), (b), and (c), with (a) denoting the initially assigned structure within each set.

Sc-XRD structures of **3g** as a co-crystal with [10]CPP as well as **4a** and **4c** allowed assignment of all symmetrical isolated *trans*-1, *trans*-2 and *trans*-3 bis-adducts.

No melting points were measured due to the low amounts of product.

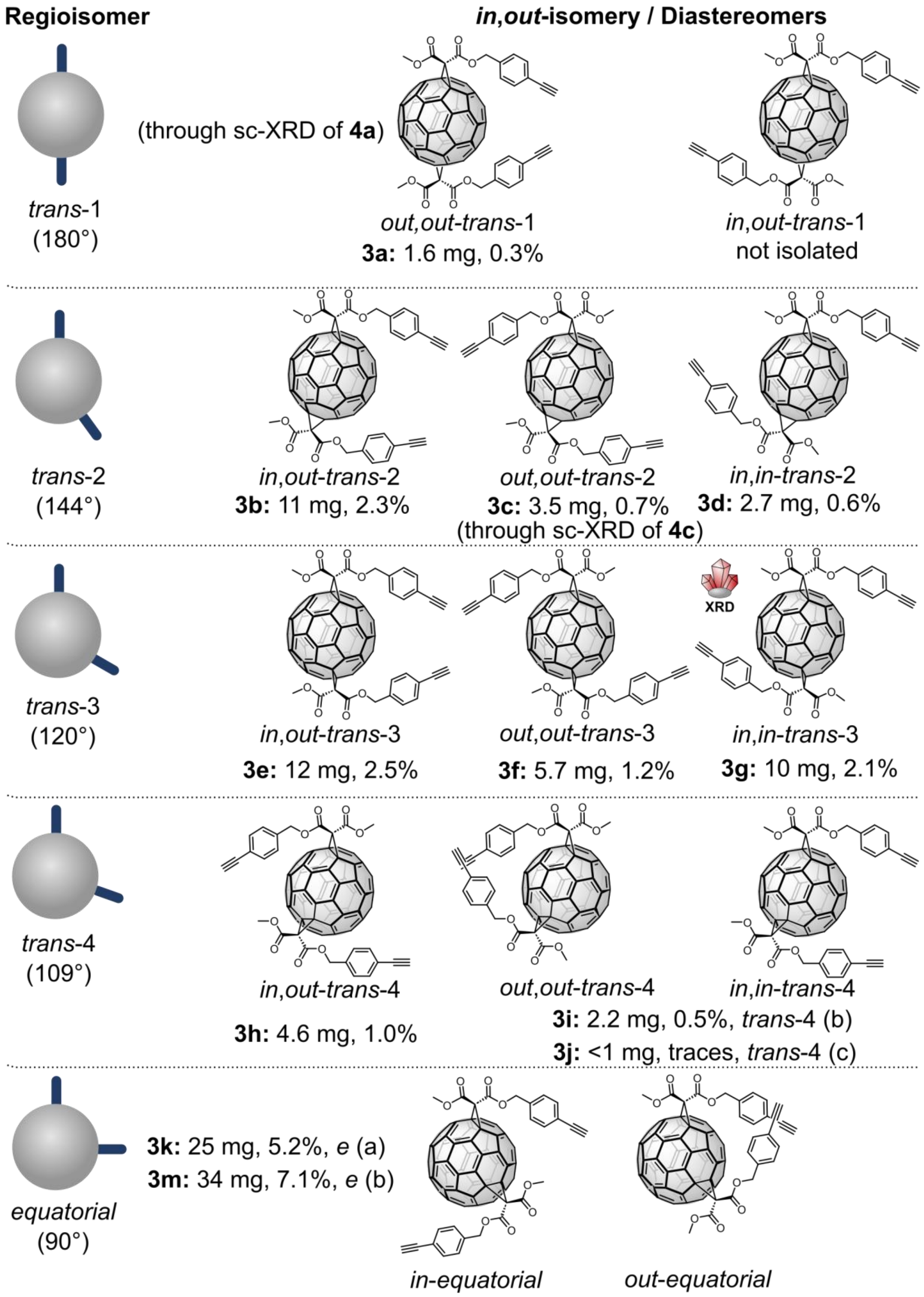

Scheme S1: Overview of all possible diastereoisomers for the *trans* and *equatorial* addition patterns of bromomalonate **2** to C_{60}, as well as yields of the 12 isolated isomers.

2.2. Separation and analysis of fullerene bis-adducts **3**

2.2.1. Preparative LC separation of the isomers

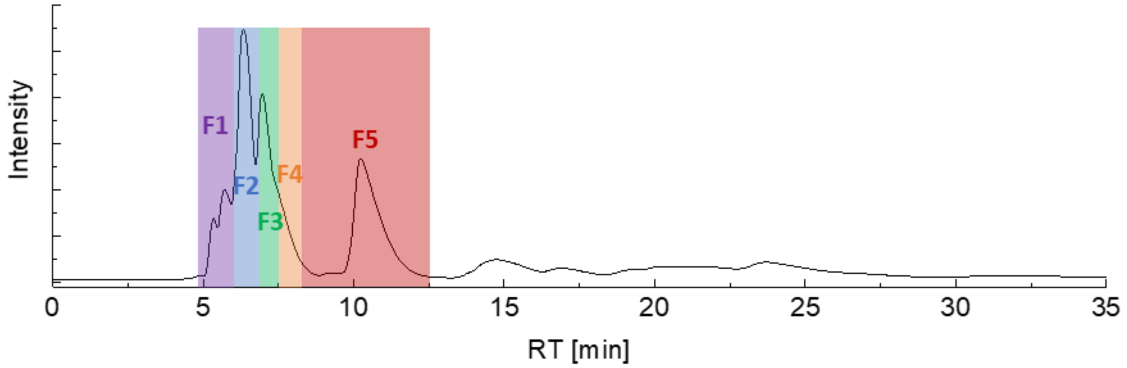

Figure S1: Fractioning of bis-adducts by HPLC (silica, toluene, 24 mL/min, r.t.) for subsequent recycling HPLC purification.

Total yield of fractions 1-5: 0.25 g, 0.22 mmol, 53% (based on bromomalonate and accounted for 2:1 stoichiometry)

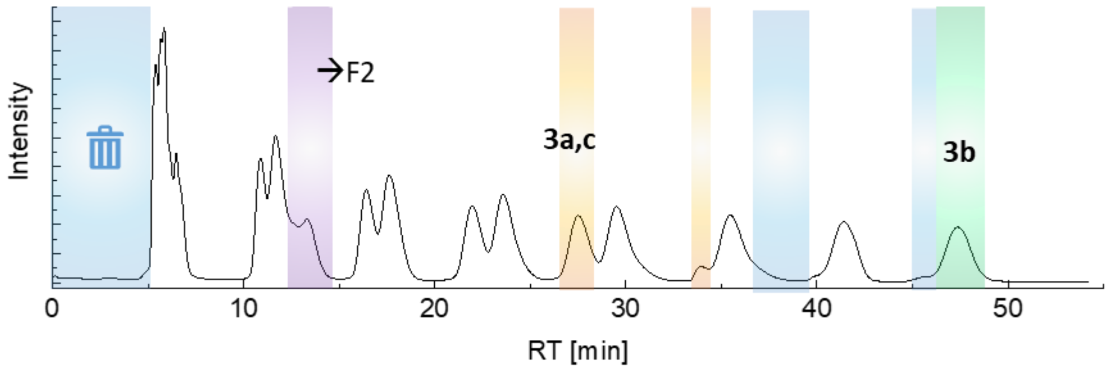

Figure S2: Representative chromatogram of recycling HPLC purification of fraction 1. The violet peak collection was combined with fraction 2 for further purification.

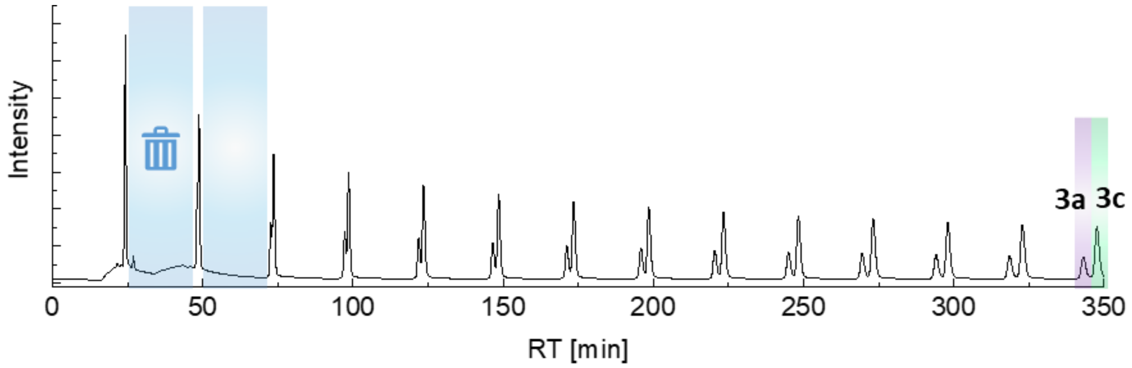

Figure S3: Chromatogram and collection for the recycling GPC (JAIGEL-2.5HR + 2HR, DCM, 10 mL/min, recycling) of bis-adducts **3a** and **3c**.

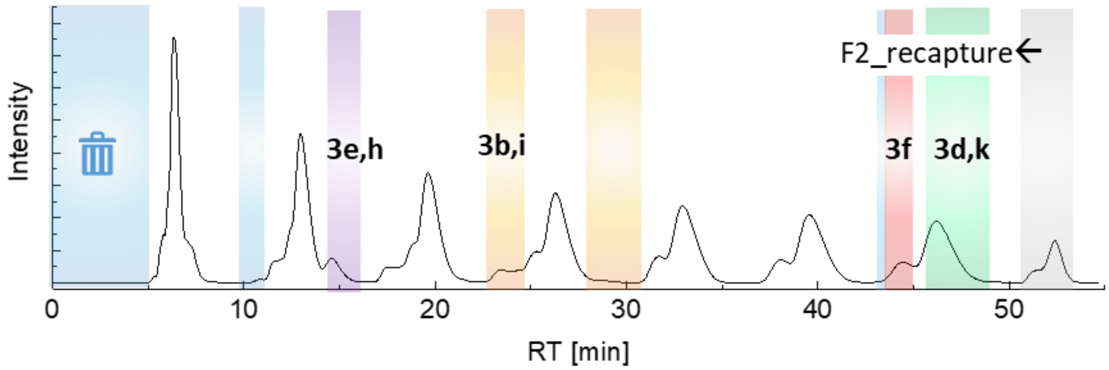

Figure S4: Representative chromatogram of recycling HPLC purification of fraction 2. The grey peak collection was recovered, combined, and purified again (**Figure S5**).

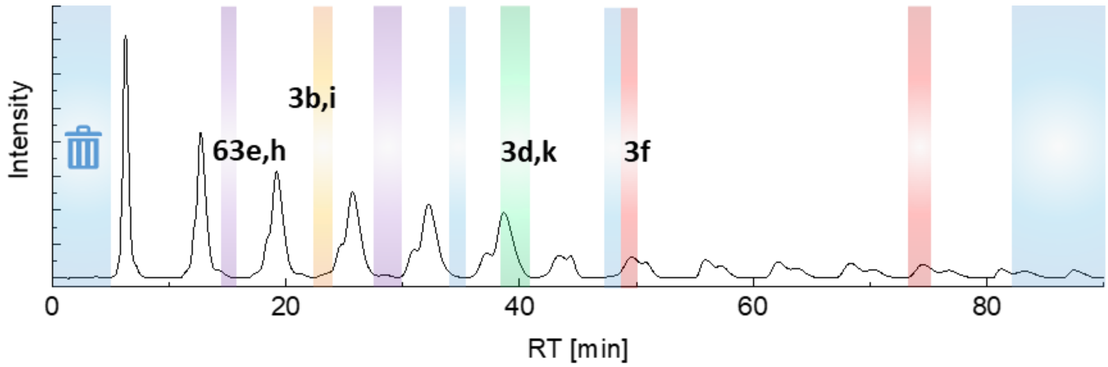

Figure S5: Recycling HPLC chromatogram of recovered fractions from prior recycling runs (compare **Figure S4**).

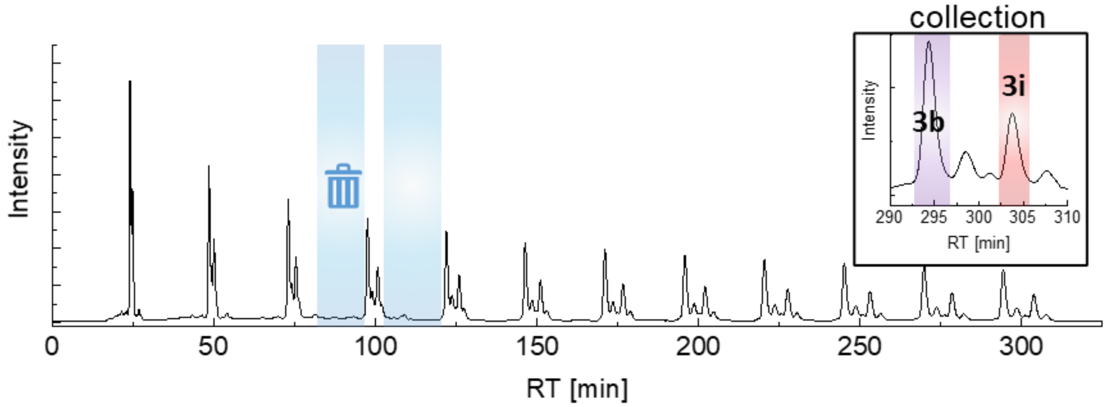

Figure S6: Chromatogram and collection for the recycling GPC (JAIGEL-2.5HR + 2HR, DCM, 10 mL/min, recycling) separation of bis-adducts **3b** and **3i**.

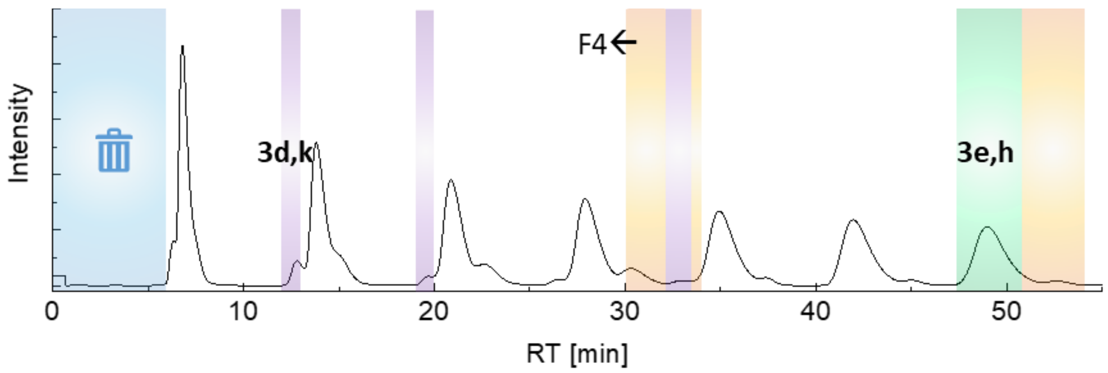

Figure S7: Representative chromatogram of recycling HPLC purification of fraction 3. The orange-colored collection was added to fraction 4 for further purification.

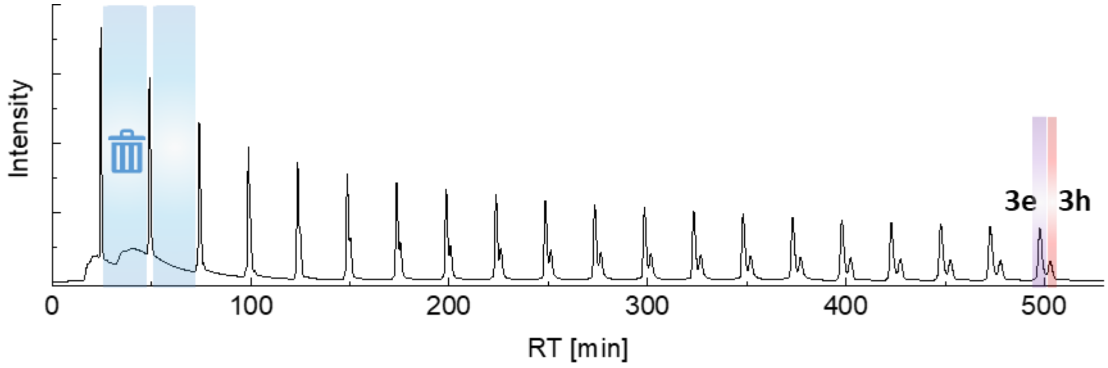

Figure S8: Chromatogram and collection for the recycling GPC (JAIGEL-2.5HR + 2HR, DCM, 10 mL/min, recycling) separation of bis-adducts **3e** and **3h**.

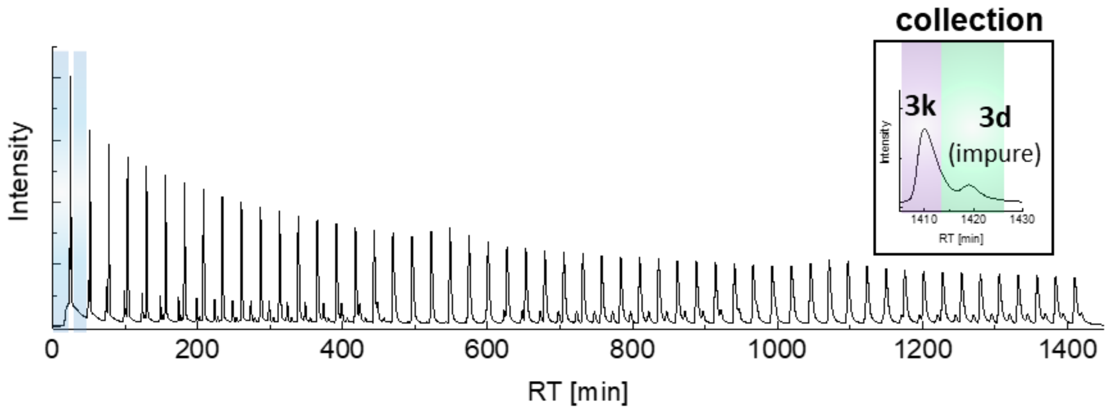

Figure S9: Chromatogram and collection for the recycling GPC (JAIGEL 2.5HR + 2HR, DCM, 10 mL/min, recycling) separation of bis-adducts **3k** and **3d**.

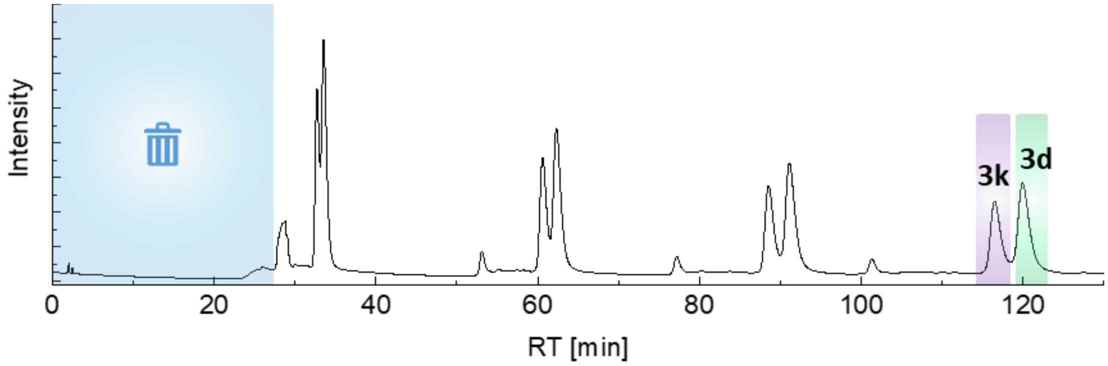

Figure S10: Chromatogram and collection for the recycling GPC (JAIGEL-2.5HR + 2HR, Toluene, 10 mL/min, recycling) separation of bis-adducts **3k** and **3d**.

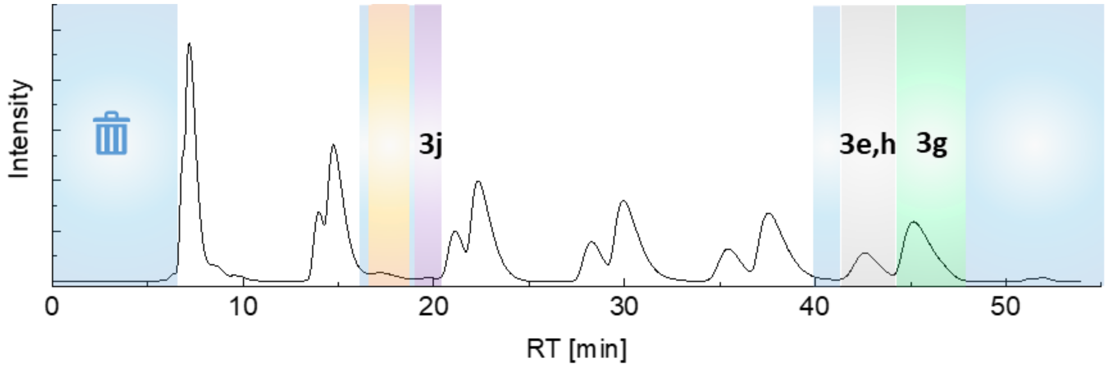

Figure S11: Representative chromatogram of recycling HPLC purification of fraction 4.

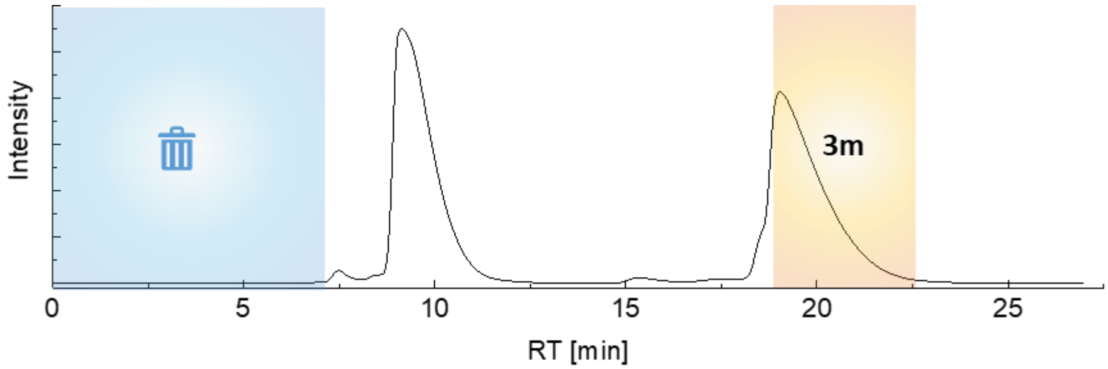

Figure S12: Chromatogram of recycling HPLC purification of fraction 5.

2.2.2. Isomeric assignment and spectroscopic data

3a: *out*,*out*-*trans*-1 (1 of 2 possible isomers)

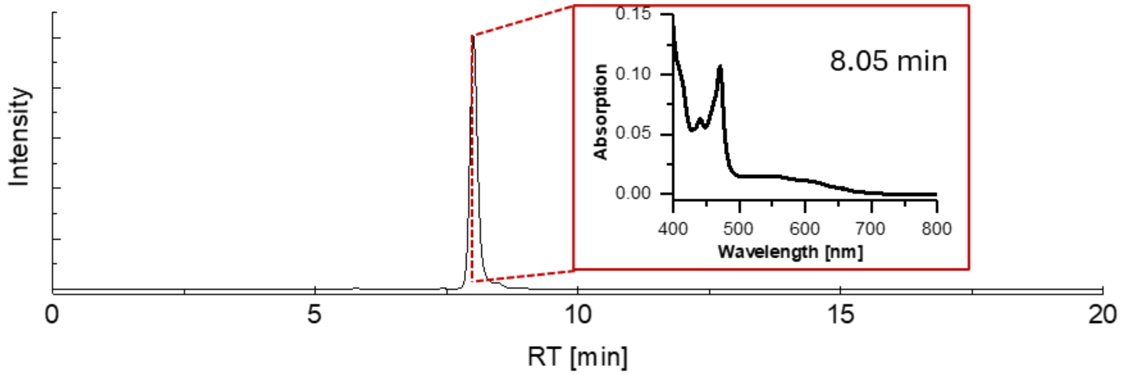

Figure S13: HPLC chromatogram (Buckyprep-M, toluene, 0.5 mL/min, 40 °C) of **3a**. Fingerprint region indicative of *trans*-1 regioisomer from PDA-detector shown in red box at the given retention time.

^{1}**H NMR** (600 MHz, $CDCl_3$, 298 K): δ = 7.59 – 7.51 (m, 8H, H_{Ar}), 5.61 (s, 4H, H_{Benzyl}), 4.12 (s, 6H, -OCH_3), 3.12 (d, ^{5}J = 2.6 Hz, 2H, $H_{Acetylene}$) ppm.

Yield: 1.6 mg, 1.4 µmol, 0.3%

3b: *in,out-trans*-2

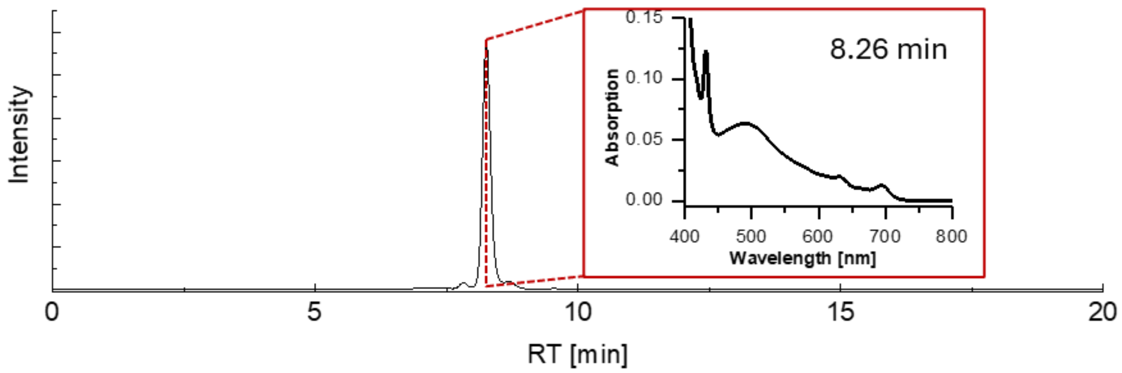

Figure S14: HPLC chromatogram (Buckyprep-M, toluene, 0.5 mL/min, 40 °C) of **3b**. Fingerprint region indicative of *trans*-2 regioisomer from PDA-detector shown in red box at the given retention time.

^{1}H NMR (400 MHz, $CDCl_3$, 298 K): δ = 7.60 – 7.56 (m, 4H, H_{Ar}), 7.51 – 7.49 (m, 2H, H_{Ar}), 7.44 – 7.42 (m, 2H, H_{Ar}), 5.65 (s, 2H, H_{Benzyl}), 5.49 – 5.45 (m, 2H, H_{Benzyl}), 4.14 (s, 3H, -OCH_3), 3.99 (s, 3H, -OCH_3), 3.13 (s, 1H, $H_{Acetylene}$), 3.10 (s, 1H, $H_{Acetylene}$) ppm.

^{13}C NMR (101 MHz, $CDCl_3$, 298 K): δ = 164.5, 164.0, 163.8, 163.5, 147.9 (C_{60}-sp^2), 147.60 (C_{60}-sp^2), 147.1 (C_{60}-sp^2), 147.0 (C_{60}-sp^2), 146.5 (C_{60}-sp^2), 146.4 (C_{60}-sp^2), 146.4 (C_{60}-sp^2), 146.3 (C_{60}-sp^2), 145.9 (C_{60}-sp^2), 145.8 (C_{60}-sp^2), 145.8 (C_{60}-sp^2), 145.7 (C_{60}-sp^2), 145.7 (C_{60}-sp^2), 145.4 (C_{60}-sp^2), 145.3 (C_{60}-sp^2), 145.2 (C_{60}-sp^2), 145.1 (C_{60}-sp^2), 144.7 (C_{60}-sp^2), 144.5 (C_{60}-sp^2), 144.5 (C_{60}-sp^2), 144.4 (C_{60}-sp^2), 144.4 (C_{60}-sp^2), 144.3 (C_{60}-sp^2), 144.2 (C_{60}-sp^2), 144.2 (C_{60}-sp^2), 144.0 (C_{60}-sp^2), 143.9 (C_{60}-sp^2), 143.9 (C_{60}-sp^2), 143.2 (C_{60}-sp^2, 2C), 143.1 (C_{60}-sp^2), 143.1 (C_{60}-sp^2), 142.9 (C_{60}-sp^2), 142.8 (C_{60}-sp^2), 142.7 (C_{60}-sp^2, 2C), 142.6 (C_{60}-sp^2), 142.5 (C_{60}-sp^2), 142.5 (C_{60}-sp^2), 142.4 (C_{60}-sp^2), 142.4 (C_{60}-sp^2), 142.2 (C_{60}-sp^2), 142.2 (C_{60}-sp^2), 142.0 (C_{60}-sp^2), 141.8 (C_{60}-sp^2), 141.8 (C_{60}-sp^2), 141.3 (C_{60}-sp^2), 140.6 (C_{60}-sp^2), 140.3 (C_{60}-sp^2), 140.3 (C_{60}-sp^2), 138.2 (C_{60}-sp^2), 138.1 (C_{60}-sp^2), 137.5 (C_{60}-sp^2), 137.4 (C_{60}-sp^2), 135.5 (C_{60}-sp^2), 135.4 (C_{60}-sp^2), 132.7, 132.5, 129.1, 128.9, 123.0, 122.9, 83.2, 83.2, 78.3, 78.2, 71.5 (C_{60}-sp^3, 2C), 70.9 (C_{60}-sp^3, 2C), 68.6, 68.4, 54.3, 54.1, 49.5 ppm.

Yield: 11 mg, 9.3 µmol, 2.3%

3c: *out,out-trans*-2

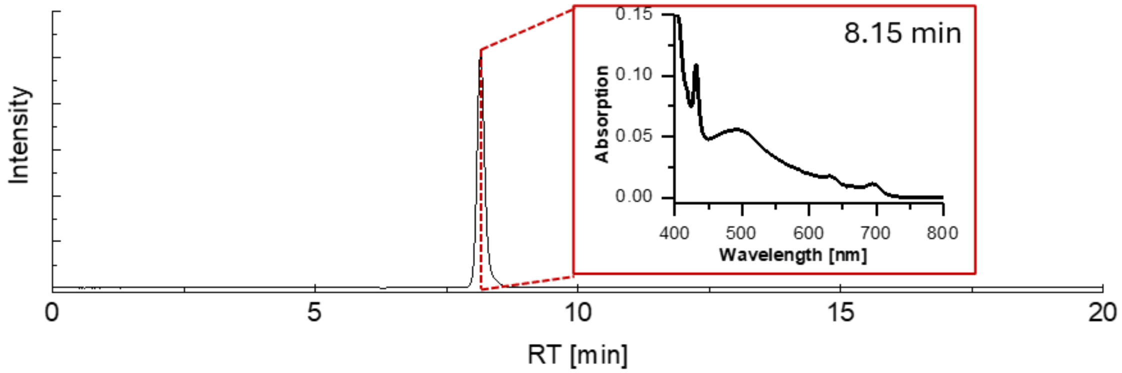

Figure S15: HPLC chromatogram (Buckyprep-M, toluene, 0.5 mL/min, 40 °C) of **3c**. Fingerprint region indicative of *trans*-2 regioisomer from PDA-detector shown in red box at the given retention time.

^{1}H NMR (400 MHz, $CDCl_3$, 298 K): δ = 7.51 (d, ^{3}J = 8.3 Hz, 8H, H_{Ar}), 7.43 (d, ^{3}J = 8.3 Hz, 1H, H_{Ar}), 5.47 (s, 4H, H_{Benzyl}), 4.14 (s, 6H, -OCH_3), 3.10 (s, 2H, $H_{Acetylene}$) ppm.

^{13}C NMR (101 MHz, $CDCl_3$, 298 K): δ = 164.5, 164.0, 163.8, 163.5, 147.9 (C_{60}-sp^2), 147.6 (C_{60}-sp^2), 147.1 (C_{60}-sp^2), 147.0 (C_{60}-sp^2), 146.5 (C_{60}-sp^2), 146.4 (C_{60}-sp^2), 146.4 (C_{60}-sp^2), 146.3 (C_{60}-sp^2), 145.9 (C_{60}-sp^2), 145.8 (C_{60}-sp^2), 145.8 (C_{60}-sp^2), 145.7 (C_{60}-sp^2), 145.7 (C_{60}-sp^2), 145.6 (C_{60}-sp^2), 145.4 (C_{60}-sp^2), 145.3 (C_{60}-sp^2), 145.2 (C_{60}-sp^2), 145.1 (C_{60}-sp^2), 144.7 (C_{60}-sp^2), 144.5 (C_{60}-sp^2), 144.5 (C_{60}-sp^2), 144.4 (C_{60}-sp^2), 144.4 (C_{60}-sp^2), 144.3 (C_{60}-sp^2), 144.2 (C_{60}-sp^2), 144.2 (C_{60}-sp^2), 144.1 (C_{60}-sp^2), 143.9 (C_{60}-sp^2, 2C), 143.9 (C_{60}-sp^2), 143.2 (C_{60}-sp^2, 2C), 143.1 (C_{60}-sp^2), 143.1 (C_{60}-sp^2), 142.9 (C_{60}-sp^2), 142.8 (C_{60}-sp^2), 142.7 (C_{60}-sp^2, 2C), 142.6 (C_{60}-sp^2), 142.5 (C_{60}-sp^2), 142.5 (C_{60}-sp^2), 142.4 (C_{60}-sp^2), 142.4 (C_{60}-sp^2), 142.2 (C_{60}-sp^2), 142.2 (C_{60}-sp^2), 142.0 (C_{60}-sp^2), 141.8 (C_{60}-sp^2), 141.8 (C_{60}-sp^2), 141.3 (C_{60}-sp^2), 140.6 (C_{60}-sp^2), 140.3 (C_{60}-sp^2), 140.3 (C_{60}-sp^2), 138.2 (C_{60}-sp^2), 138.0 (C_{60}-sp^2), 137.5 (C_{60}-sp^2), 137.4 (C_{60}-sp^2), 135.5, 135.4, 132.7, 132.5, 129.1, 128.9, 123.0, 122.9, 83.2, 83.2, 78.3, 78.2, 71.5 (C_{60}-sp^3, 2C), 70.9 (C_{60}-sp^3, 2C), 68.6, 68.4, 54.3, 54.1, 49.5, 49.5 ppm.

Yield: 3.5 mg, 3.0 µmol, 0.7%

3d: *in*,*in*-*trans*-2

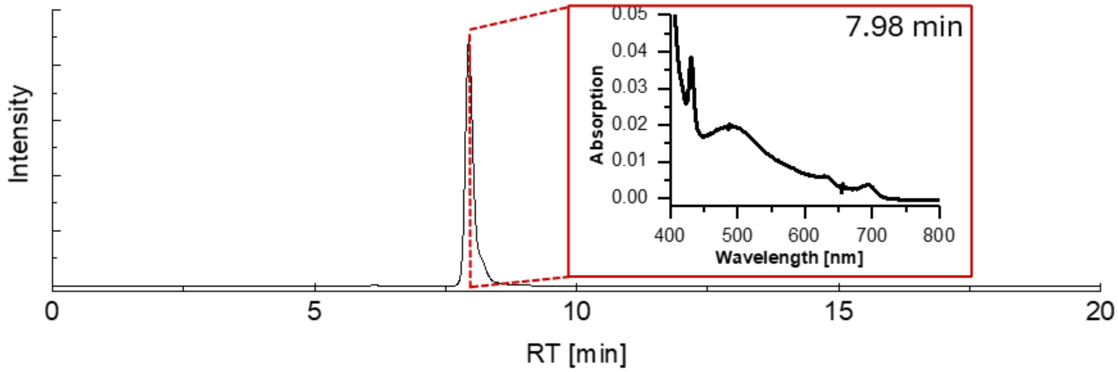

Figure S16: HPLC chromatogram (Buckyprep-M, toluene, 0.5 mL/min, 40 °C) of **3d**. Fingerprint region indicative of *trans*-2 regioisomer from PDA-detector shown in red box at the given retention time.

^{1}H NMR (600 MHz, $CDCl_3$, 298 K): δ = 7.62 – 7.54 (m, 8H, H_{Ar}), 5.65 (s, 4H, H_{Benzyl}), 3.98 (s, 6H, -OCH_3), 3.13 (s, 2H, $H_{Acetylene}$) ppm.

^{13}C NMR (151 MHz, $CDCl_3$, 298 K): δ = 164.0, 163.8, 147.9 (C_{60}-sp^2), 147.0 (C_{60}-sp^2), 146.4 (C_{60}-sp^2), 146.3 (C_{60}-sp^2), 145.9 (C_{60}-sp^2), 145.8 (C_{60}-sp^2), 145.6 (C_{60}-sp^2), 145.3 (C_{60}-sp^2), 145.2 (C_{60}-sp^2), 144.6 (C_{60}-sp^2), 144.4 (C_{60}-sp^2), 144.4 (C_{60}-sp^2), 144.2 (C_{60}-sp^2), 143.9 (C_{60}-sp^2), 143.9 (C_{60}-sp^2), 143.2 (C_{60}-sp^2), 143.1 (C_{60}-sp^2), 142.8 (C_{60}-sp^2), 142.7 (C_{60}-sp^2), 142.5 (C_{60}-sp^2), 142.5 (C_{60}-sp^2), 142.3 (C_{60}-sp^2), 142.0 (C_{60}-sp^2), 141.8 (C_{60}-sp^2), 140.6 (C_{60}-sp^2), 140.3 (C_{60}-sp^2), 138.2 (C_{60}-sp^2), 138.1 (C_{60}-sp^2), 135.5, 132.7, 129.1, 123.0, 83.2, 78.3, 71.5 (C_{60}-sp^3), 70.9 (C_{60}-sp^3), 68.6, 54.1, 49.5 ppm.

Yield: 2.7 mg, 2.3 µmol, 0.6%

3e: *in*,*out*-*trans*-3

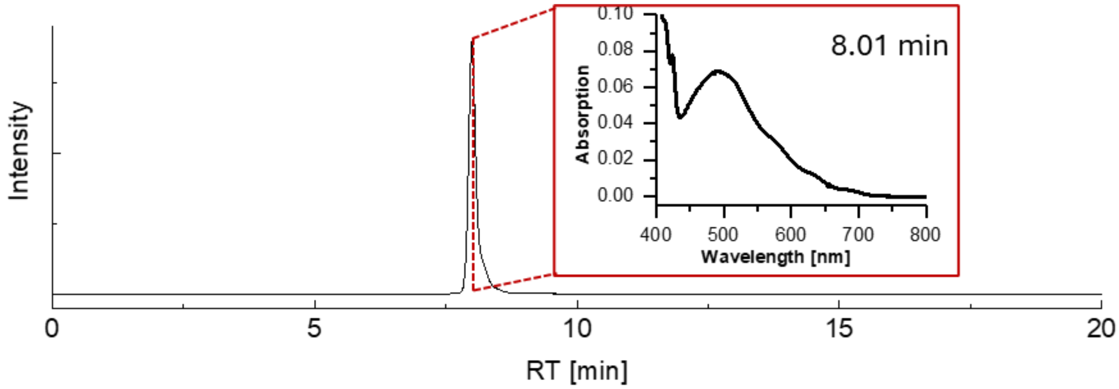

Figure S17: HPLC chromatogram (Buckyprep-M, toluene, 0.5 mL/min, 40 °C) of **3e**. Fingerprint region indicative of *trans*-3 regioisomer from PDA-detector shown in red box at the given retention time.

^{1}H NMR (400 MHz, $CDCl_3$, 298 K): δ = 7.57 – 7.54 (m, 2H, H_{Ar}),7.50 – 7.48 (m, 4H, H_{Ar}), 7.42 – 7.34 (m, 2H, H_{Ar}), 5.53 (AB, J_{AB} = 12.25 Hz, 2H, H_{Benzyl}), 5.41 (s, 2H, H_{Benzyl}), 4.03 (s, 3H, -OCH_3), 3.93 (s, 3H, -OCH_3), 3.12 (s, 1H, $H_{Acetylene}$), 3.10 (s, 1H, $H_{Acetylene}$) ppm.

^{13}C NMR (101 MHz, $CDCl_3$, 298 K): δ = 164.0, 164.0, 163.4, 163.4, 147.3 (C_{60}-sp^2), 147.2 (C_{60}-sp^2), 147.2 (C_{60}-sp^2), 147.1 (C_{60}-sp^2), 146.8 (C_{60}-sp^2), 146.7 (C_{60}-sp^2), 146.7 (C_{60}-sp^2), 146.6 (C_{60}-sp^2), 146.5 (C_{60}-sp^2, 2C), 146.5 (C_{60}-sp^2), 146.4 (C_{60}-sp^2), 146.3 (C_{60}-sp^2), 146.1 (C_{60}-sp^2), 146.0 (C_{60}-sp^2), 145.9 (C_{60}-sp^2), 145.8 (C_{60}-sp^2), 145.8 (C_{60}-sp^2), 145.7 (C_{60}-sp^2), 145.6 (C_{60}-sp^2), 145.5 (C_{60}-sp^2), 145.5 (C_{60}-sp^2), 145.4 (C_{60}-sp^2), 145.1 (C_{60}-sp^2), 144.8 (C_{60}-sp^2), 144.8 (C_{60}-sp^2), 144.6 (C_{60}-sp^2), 144.6 (C_{60}-sp^2), 144.3 (C_{60}-sp^2), 144.3 (C_{60}-sp^2), 144.0 (C_{60}-sp^2, 2C), 143.7 (C_{60}-sp^2, 2C), 143.7 (C_{60}-sp^2), 143.6(C_{60}-sp^2, 2C), 143.5 (C_{60}-sp^2), 143.2 (C_{60}-sp^2), 143.1 (C_{60}-sp^2), 142.7 (C_{60}-sp^2), 142.7 (C_{60}-sp^2), 142.7 (C_{60}-sp^2), 142.6 (C_{60}-sp^2), 142.1 (C_{60}-sp^2), 142.0 (C_{60}-sp^2, 2C), 142.0 (C_{60}-sp^2), 141.8 (C_{60}-sp^2), 141.7 (C_{60}-sp^2), 140.5 (C_{60}-sp^2), 140.5 (C_{60}-sp^2), 139.7 (C_{60}-sp^2), 139.0 (C_{60}-sp^2), 139.0 (C_{60}-sp^2), 138.4 (C_{60}-sp^2), 135.4, 135.3, 132.6, 132.5, 129.0, 128.9, 123.0, 122.9, 83.2, 83.2, 78.3, 78.3, 71.6 (C_{60}-sp^3), 71.6 (C_{60}-sp^3), 71.2 (C_{60}-sp^3), 71.1 (C_{60}-sp^3), 68.5, 68.4, 54.1, 54.0, 51.3, 51.3 ppm.

Yield: 12 mg, 10 µmol, 2.5%

3f: *out*,*out*-*trans*-3

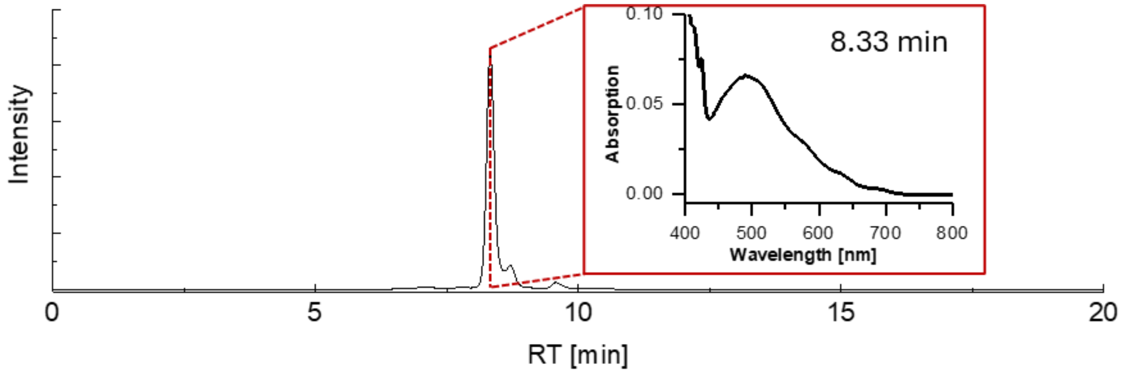

Figure S18: HPLC chromatogram (Buckyprep-M, toluene, 0.5 mL/min, 40 °C) of **3f**. Fingerprint region indicative of *trans*-3 regioisomer from PDA-detector shown in red box at the given retention time.

^{1}H NMR (600 MHz, $CDCl_3$, 298 K): δ = 7.49 (d, ^3J = 8.0 Hz, 4H, H_{Ar}), 7.38 (d, ^3J = 8.0 Hz, 4H, H_{Ar}), 5.42 (s, 4H, H_{Benzyl}), 4.03 (s, 6H, -OCH_3), 3.10 (s, 2H, $H_{Acetylene}$) ppm.

^{13}C NMR (151 MHz, $CDCl_3$, 298 K): δ = 164.0, 163.4, 147.2 (C_{60}-sp^2, 2C), 147.2 (C_{60}-sp^2, 2C), 146.8 (C_{60}-sp^2, 2C), 146.6 (C_{60}-sp^2, 2C), 146.6 (C_{60}-sp^2, 2C), 146.6 (C_{60}-sp^2, 2C), 146.1 (C_{60}-sp^2, 2C), 146.0 (C_{60}-sp^2, 2C), 145.8 (C_{60}-sp^2, 2C), 145.8 (C_{60}-sp^2, 2C), 145.5 (C_{60}-sp^2, 2C), 145.4 (C_{60}-sp^2, 2C), 144.8 (C_{60}-sp^2, 2C), 144.6 (C_{60}-sp^2, 2C), 144.3 (C_{60}-sp^2, 2C), 144.0 (C_{60}-sp^2, 2C), 143.7 (C_{60}-sp^2, 2C), 143.6 (C_{60}-sp^2, 2C), 143.6 (C_{60}-sp^2, 2C), 143.2 (C_{60}-sp^2, 2C), 142.8 (C_{60}-sp^2, 2C), 142.7 (C_{60}-sp^2, 2C), 142.6 (C_{60}-sp^2, 2C), 142.1 (C_{60}-sp^2, 2C), 141.8 (C_{60}-sp^2, 2C), 140.5 (C_{60}-sp^2, 2C), 139.1 (C_{60}-sp^2, 2C), 138.4 (C_{60}-sp^2, 2C), 135.3, 132.6, 132.5, 128.9, 122.9, 83.2, 78.2, 71.6 (C_{60}-sp^3, 2C), 71.2 (C_{60}-sp^3, 2C), 68.4, 54.1, 51.3 ppm.

All of the expected 30 C_{60} signals were found for a molecule with C_2 symmetry.

Yield: 5.7 mg, 4.8 µmol, 1.2%

3g: *in*,*in*-*trans*-3

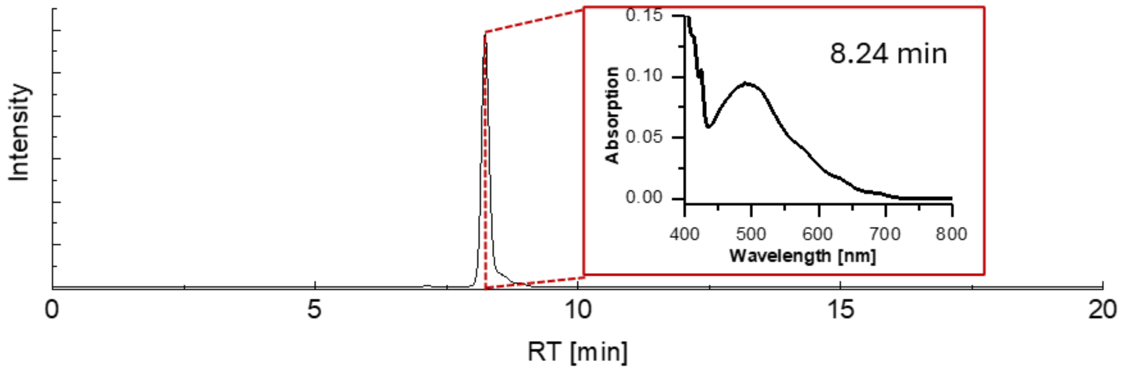

Figure S19: HPLC chromatogram (Buckyprep-M, toluene, 0.5 mL/min, 40 °C) of **3g**. Fingerprint region indicative of *trans*-3 regioisomer from PDA-detector shown in red box at the given retention time.

^{1}H NMR (600 MHz, $CDCl_3$, 298 K): δ = 7.55 (d, ^3J = 8.3 Hz, 4H), 7.48 (d, ^3J = 8.3 Hz, 4H), 5.53 (AB, J_{AB} = 12.27 Hz, 4H, H_{Benzyl}), 3.93 (s, 6H, -OCH_3), 3.12 (s, 2H, $H_{Acetylene}$) ppm.

^{13}C NMR (151 MHz, $CDCl_3$, 298 K) δ = 164.0, 163.4, 147.4 (C_{60}-sp^2, 2C), 147.2 (C_{60}-sp^2, 2C), 146.7 (C_{60}-sp^2, 2C), 146.6 (C_{60}-sp^2, 2C), 146.5 (C_{60}-sp^2, 2C), 146.5 (C_{60}-sp^2, 2C), 146.4 (C_{60}-sp^2, 2C), 146.0 (C_{60}-sp^2, 2C), 145.8 (C_{60}-sp^2, 2C), 145.7 (C_{60}-sp^2, 2C), 145.5 (C_{60}-sp^2, 2C), 145.2 (C_{60}-sp^2, 2C), 144.9 (C_{60}-sp^2, 2C), 144.6 (C_{60}-sp^2, 2C), 144.3 (C_{60}-sp^2, 2C), 144.0 (C_{60}-sp^2, 2C), 143.7 (C_{60}-sp^2, 2C), 143.7 (C_{60}-sp^2, 2C), 143.6 (C_{60}-sp^2, 2C), 143.1 (C_{60}-sp^2, 2C), 142.7 (C_{60}-sp^2, 2C), 142.1 (C_{60}-sp^2, 2C), 142.1 (C_{60}-sp^2, 2C), 142.0 (C_{60}-sp^2, 2C), 141.7 (C_{60}-sp^2, 2C), 140.5 (C_{60}-sp^2, 2C), 139.7 (C_{60}-sp^2, 2C), 139.0 (C_{60}-sp^2, 2C), 135.4, 132.6, 129.0, 123.0, 83.2, 78.3, 71.6 (C_{60}-sp^3, 2C), 71.2 (C_{60}-sp^3, 2C), 68.5, 54.0, 51.3 ppm.

All of the expected 30 C_{60} signals were found for a molecule with C_2 symmetry.

Yield: 10 mg, 8.7 µmol, 2.1%

3h: *in,out-trans*-4

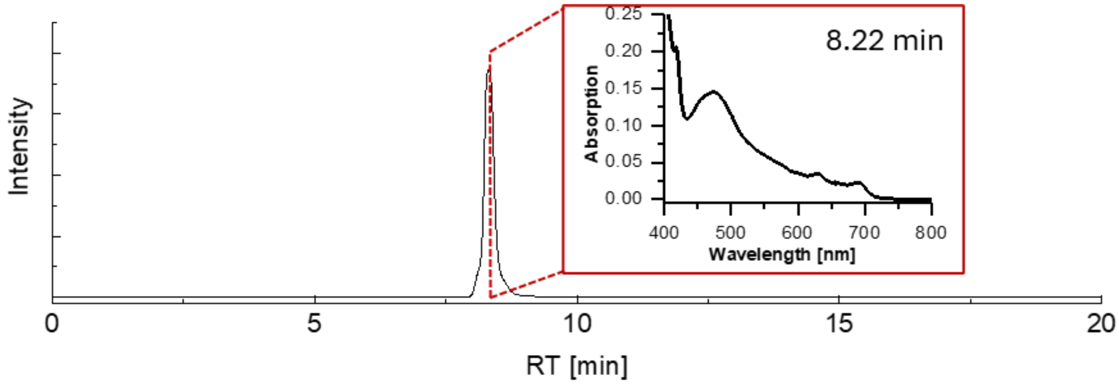

Figure S20: HPLC chromatogram (Buckyprep-M, toluene, 0.5 mL/min, 40 °C) of **3h**. Fingerprint region indicative of *trans*-4 regioisomer from PDA-detector shown in red box at the given retention time.

^{1}**H NMR** (400 MHz, $CDCl_3$, 298 K): δ = 7.56 – 7.38 (m, 8H, H_{Ar}), 5.49 (s, 2H, H_{Benzyl}), 5.41 (AB, J_{AB} = 12.28 Hz, 2H, H_{Benzyl}), 3.99 (s, 3H, -OCH_3), 3.95 (s, 3H, -OCH_3), 3.13 (s, 1H, $H_{Acetylene}$), 3.10 (s, 1H, $H_{Acetylene}$) ppm.

^{13}**C NMR** (101 MHz, $CDCl_3$, 298 K): δ = 164.1, 164.1, 163.5, 163.5, 148.4 (C_{60}-sp^2), 147.2 (C_{60}-sp^2), 147.1(C_{60}-sp^2), 146.7(C_{60}-sp^2), 146.3 (C_{60}-sp^2, 2C), 146.2(C_{60}-sp^2), 146.2 (C_{60}-sp^2), 145.8 (C_{60}-sp^2, 2C), 145.6 (C_{60}-sp^2), 145.5(C_{60}-sp^2), 145.5 (C_{60}-sp^2, 2C), 145.5 (C_{60}-sp^2), 145.3 (C_{60}-sp^2, 2C), 145.2 (C_{60}-sp^2), 145.0 (C_{60}-sp^2), 144.9 (C_{60}-sp^2), 144.9 (C_{60}-sp^2), 144.8 (C_{60}-sp^2), 144.7 (C_{60}-sp^2), 144.4 (C_{60}-sp^2), 144.3 (C_{60}-sp^2), 144.0 (C_{60}-sp^2), 143.9 (C_{60}-sp^2), 143.3 (C_{60}-sp^2), 143.3 (C_{60}-sp^2), 143.2 (C_{60}-sp^2), 143.1 (C_{60}-sp^2), 143.0 (C_{60}-sp^2), 143.0 (C_{60}-sp^2, 2C), 142.8 (C_{60}-sp^2), 142.4 (C_{60}-sp^2), 142.4 (C_{60}-sp^2), 142.3 (C_{60}-sp^2, 2C), 142.1 (C_{60}-sp^2, 2C), 142.1 (C_{60}-sp^2), 141.9 (C_{60}-sp^2), 141.8 (C_{60}-sp^2), 141.8 (C_{60}-sp^2), 141.5 (C_{60}-sp^2), 141.2 (C_{60}-sp^2), 141.2 (C_{60}-sp^2), 141.0 (C_{60}-sp^2), 140.5 (C_{60}-sp^2), 139.5 (C_{60}-sp^2), 139.0 (C_{60}-sp^2), 138.9 (C_{60}-sp^2), 138.4 (C_{60}-sp^2), 136.1 (C_{60}-sp^2), 135.6 (C_{60}-sp^2), 135.4, 135.4, 132.6, 132.5, 128.9, 128.7, 123.0, 122.8, 83.2, 78.3, 78.2, 71.1 (C_{60}-sp^3, 2C), 70.8 (C_{60}-sp^3, 2C), 68.4, 68.3, 54.1, 54.0, 49.9, 49.8 ppm.

Yield: 4.6 mg, 3.9 µmol, 1.0%

3i: *trans*-4 (b)

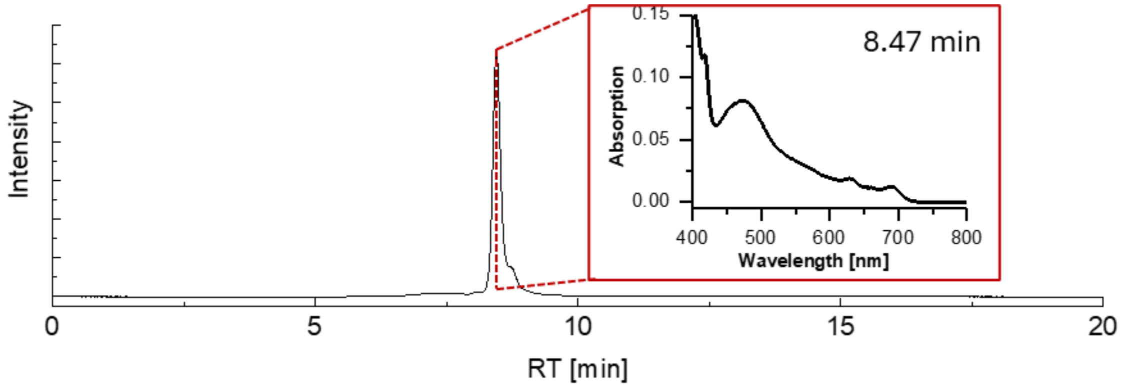

Figure S21: HPLC chromatogram (Buckyprep-M, toluene, 0.5 mL/min, 40 °C) of **3i**. Fingerprint region indicative of *trans*-4 regioisomer from PDA-detector shown in red box at the given retention time.

^{1}H NMR (400 MHz, $CDCl_3$, 298 K): δ = 7.52 (d, ^3J = 8.0 Hz, 4H), 7.40 (d, ^3J = 8.0 Hz, 4H), 5.49 – 5.37 (m, 4H, H_{Benzyl}), 3.99 (s, 6H, -OCH_3), 3.10 (s, 2H, $H_{Acetylene}$) ppm.

Yield: 2.2 mg, 1.9 µmol, 0.5%

3j: *trans*-4 (c)

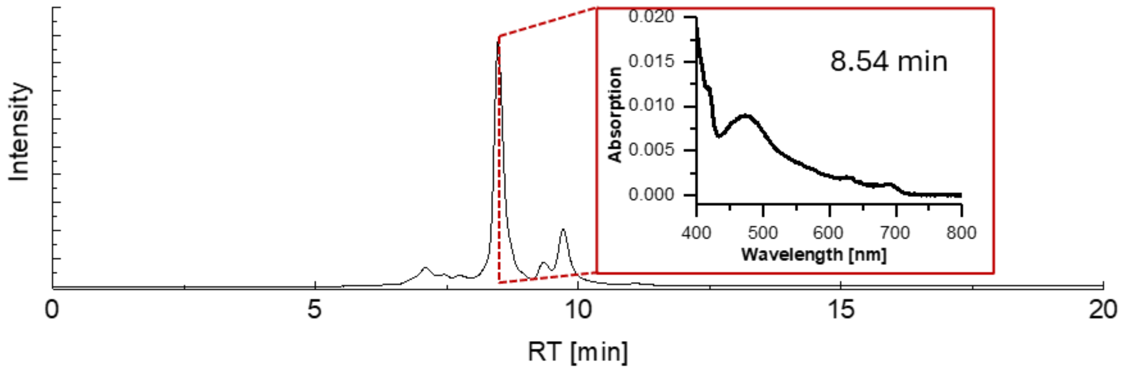

Figure S22: HPLC chromatogram (Buckyprep-M, toluene, 0.5 mL/min, 40 °C) of **3j**. Fingerprint region indicative of *trans*-4 regioisomer from PDA-detector shown in red box at the given retention time.

^{1}H NMR (600 MHz, $CDCl_3$, 298 K): δ = 7.55 – 7.54 (m, 4H, H_{Ar}), 7.46 – 7.45 (m, 4H, H_{Ar}), 5.49 (s, 4H, H_{Benzyl}), 3.93 (s, 6H, -OCH_3), 3.12 (s, 2H, $H_{Acetylene}$) ppm.

Yield: <1 mg

While the isomer sample contained significant amounts of impurities (**Figure S22**), these did not interfere with the regioisomeric assignment by the fingerprint region of its UV-Vis absorption spectrum, nor the assignment of the resonances in its ^{1}H NMR spectrum. As the *trans*-4 isomers are not relevant for the subsequent [2]catenane syntheses, no further purification efforts were undertaken.

3k: *equatorial* (a)

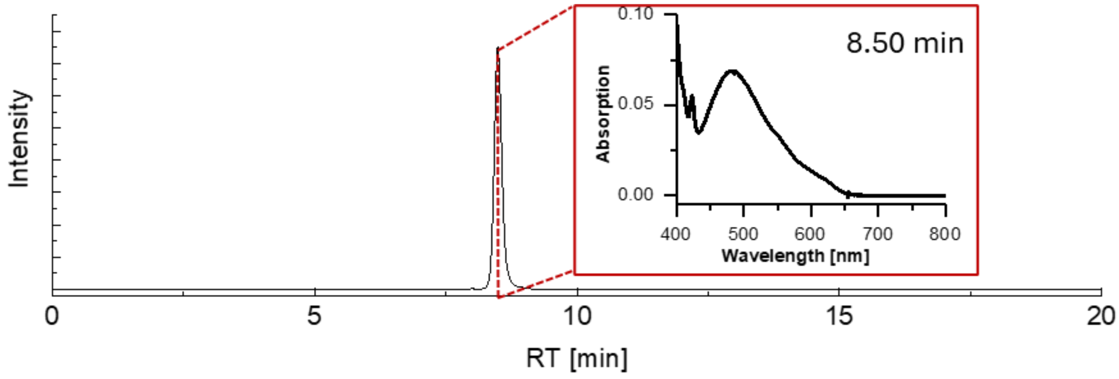

Figure S23: HPLC chromatogram (Buckyprep-M, toluene, 0.5 mL/min, 40 °C) of **3k**. Fingerprint region indicative of *equatorial* regioisomer from PDA-detector shown in red box at the given retention time.

^{1}H NMR (600 MHz, $CDCl_3$, 298 K): δ = 7.61 – 7.44 (m, 4H, H_{Ar}), 7.43 – 7.35 (m, 4H, H_{Ar}), 5.44 (AB, J_{AB} = 12.3 Hz, 2H, H_{Benzyl}), 5.38 (s, 2H, H_{Benzyl}), 3.94 (s, 3H, -OCH_3), 3.94 (s, 3H, -OCH_3), 3.11 (s, 1H, $H_{Acetylene}$), 3.10 (s, 1H, $H_{Acetylene}$) ppm.

^{13}C NMR (151 MHz, $CDCl_3$, 298 K): δ = 164.0, 163.8, 163.4, 163.3, 147.5 (C_{60}-sp^2), 147.4 (C_{60}-sp^2, 2C), 147.4 (C_{60}-sp^2), 146.7 (C_{60}-sp^2, 2C), 146.6 (C_{60}-sp^2), 146.3 (C_{60}-sp^2), 146.2 (C_{60}-sp^2, 2C), 146.2 (C_{60}-sp^2), 145.8 (C_{60}-sp^2), 145.7 (C_{60}-sp^2), 145.5 (C_{60}-sp^2), 145.5 (C_{60}-sp^2), 145.3 (C_{60}-sp^2, 2C), 145.2 (C_{60}-sp^2), 144.9 (C_{60}-sp^2), 144.9 (C_{60}-sp^2), 144.9 (C_{60}-sp^2, 2C), 144.8 (C_{60}-sp^2, 2C), 144.5 (C_{60}-sp^3, 2C), 144.2 (C_{60}-sp^2), 144.2 (C_{60}-sp^2, 2C), 144.2 (C_{60}-sp^2), 144.1 (C_{60}-sp^2, 2C), 143.9 (C_{60}-sp^2, 2C), 143.9 (C_{60}-sp^2), 143.8 (C_{60}-sp^2), 143.6 (C_{60}-sp^2), 143.6 (C_{60}-sp^2), 143.1 (C_{60}-sp^2, 2C), 143.1 (C_{60}-sp^2), 143.1 (C_{60}-sp^2), 143.0 (C_{60}-sp^2), 142.9 (C_{60}-sp^2), 142.8 (C_{60}-sp^2), 142.33 (C_{60}-sp^2), 142.07 (C_{60}-sp^2, 2C), 142.04 (C_{60}-sp^2), 141.68 (C_{60}-sp^2), 141.66 (C_{60}-sp^2), 141.5 (C_{60}-sp^2), 139.2 (C_{60}-sp^2, 2C), 138.9 (C_{60}-sp^2), 138.8 (C_{60}-sp^2), 135.4, 135.3, 132.5, 132.5, 128.9, 128.6, 122.9, 122.8, 83.3, 83.2, 78.2, 78.2, 71.5 (C_{60}-sp^3), 71.5 (C_{60}-sp^3), 71.4 (C_{60}-sp^3), 70.3 (C_{60}-sp^3), 68.3, 68.3, 54.0, 54.0, 53.2, 51.0 ppm.

Yield: 25 mg, 21 μmol, 5.2%

3m: *equatorial* (b)

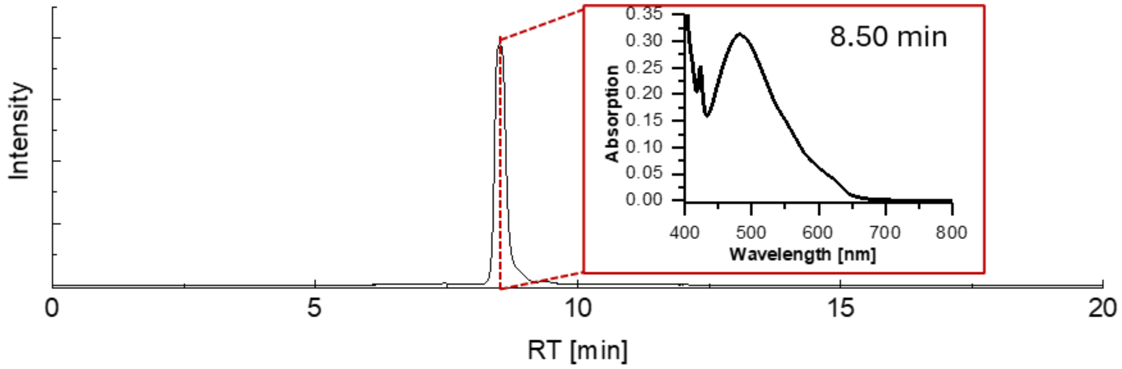

Figure S24: HPLC chromatogram (Buckyprep-M, toluene, 0.5 mL/min, 40 °C) of **3m**. Fingerprint region indicative of *equatorial* regioisomer from PDA-detector shown in red box at the given retention time.

^{1}**H NMR** (600 MHz, $CDCl_3$, 298 K): δ = 7.61 – 7.44 (m, 4H, H_{Ar}), 7.44 – 7.38 (m, 4H, H_{Ar}), 5.44 (s, 2H, H_{Benzyl}), 5.42 (AB, J_{AB} = 12.43 Hz, 2H, H_{Benzyl}), 3.92 (s, 3H, -OCH_3), 3.90 (s, 3H, -OCH_3), 3.13 (s, 1H, $H_{Acetylene}$), 3.10 (s, 1H, $H_{Acetylene}$) ppm.

^{13}**C NMR** (151 MHz, $CDCl_3$, 298 K): δ = 164.0, 163.9, 163.5, 163.3, 147.5 (C_{60}-sp^2), 147.4 (C_{60}-sp^2, 2C), 147.4 (C_{60}-sp^2), 146.6 (C_{60}-sp^2, 2C), 146.5 (C_{60}-sp^2), 146.3 (C_{60}-sp^2), 146.2 (C_{60}-sp^2, 2C), 146.2 (C_{60}-sp^2), 145.8 (C_{60}-sp^2), 145.7 (C_{60}-sp^2), 145.6 (C_{60}-sp^2), 145.5 (C_{60}-sp^2), 145.2 (C_{60}-sp^2), 145.1 (C_{60}-sp^2, 2C), 144.9 (C_{60}-sp^2), 144.9 (C_{60}-sp^2), 144.9 (C_{60}-sp^2, 2C), 144.8 (C_{60}-sp^2, 2C), 144.5 (C_{60}-sp^2, 2C), 144.3 (C_{60}-sp^2), 144.3 (C_{60}-sp^2), 144.2 (C_{60}-sp^2), 144.2 (C_{60}-sp^2), 143.9 (C_{60}-sp^2), 143.9 (C_{60}-sp^2, 4C), 143.8 (C_{60}-sp^2), 143.6 (C_{60}-sp^2), 143.6 (C_{60}-sp^2, 2C), 143.5 (C_{60}-sp^2), 143.2 (C_{60}-sp^2), 143.0 (C_{60}-sp^2), 143.0 (C_{60}-sp^2), 143.0 (C_{60}-sp^2), 142.8 (C_{60}-sp^2), 142.4 (C_{60}-sp^2), 142.1 (C_{60}-sp^2, 3C), 141.7 (C_{60}-sp^2), 141.7 (C_{60}-sp^2), 141.5 (C_{60}-sp^2), 138.9 (C_{60}-sp^2), 138.8 (C_{60}-sp^2), 138.7 (C_{60}-sp^2, 2C), 135.4, 135.4, 132.6, 132.5, 129.0, 128.9, 123.0, 122.9, 83.2, 83.2, 78.3, 78.2, 71.5 (C_{60}-sp^3), 71.5 (C_{60}-sp^3), 71.5 (C_{60}-sp^3), 70.2 (C_{60}-sp^3), 68.4, 68.3, 54.0, 54.0, 53.2, 51.0 ppm.

Yield: 34 mg, 29 µmol, 7.1%

2.3. Synthesis of the [2]catenanes

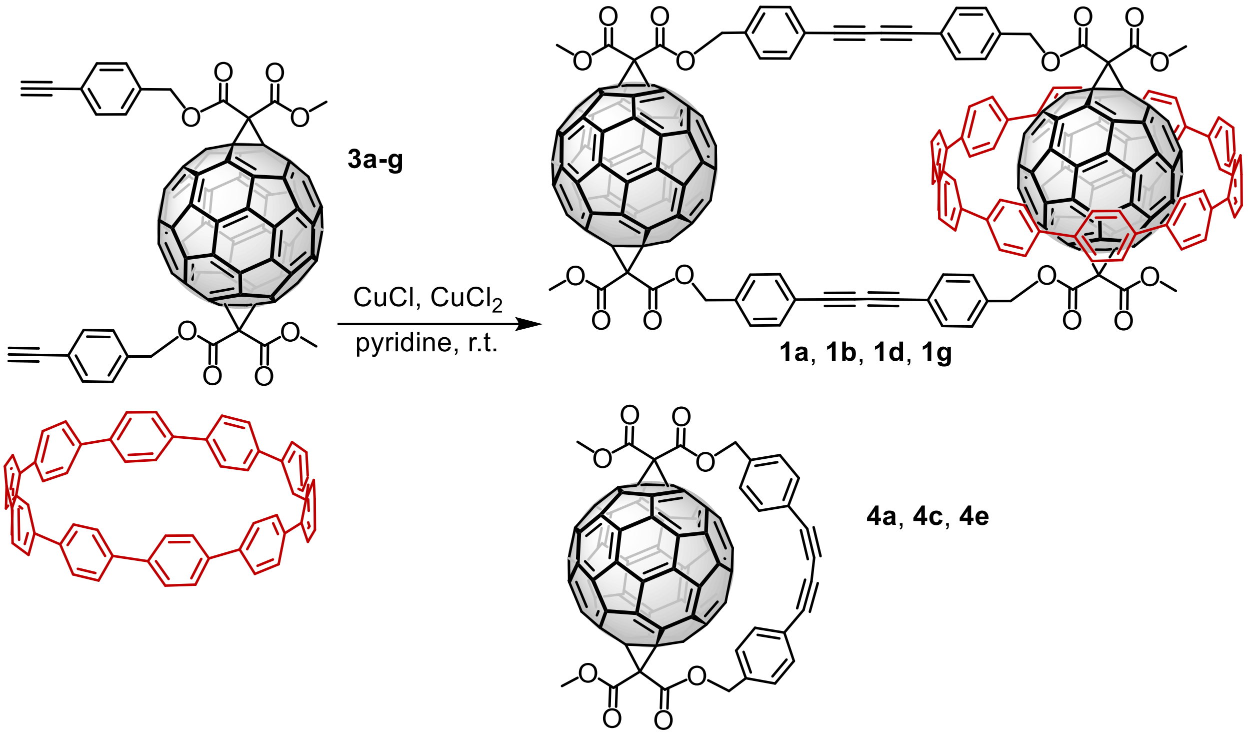

General Procedure:

The general procedure and equivalents for the oxidative coupling were adapted from literature.[S4]

In a glovebox under nitrogen atmosphere a solution of methyl 4-ethynylbenzyl malonate C_{60} bis-adduct **3a-g** and [10]CPP (0.5 equiv.) in anh. pyridine was added slowly to a suspension of copper(I) chloride (170 equiv.) and copper(II) chloride (17 equiv.) in anh. pyridine. The reaction mixture (0.35 mM) was stirred for 6-17 h at r.t. before the reaction was taken out of the glovebox and the pyridine was evaporated under reduced pressure. The residue was taken up with dichloromethane and washed with 2 M HCl and saturated ammonium chloride solution to remove copper and pyridine residues. The aqueous phase was extracted with dichloromethane and the combined organic phases were dried over sodium sulfate, filtered, and evaporated.

The residue was dissolved in toluene and a solution of C_{60} in toluene (6 equiv.) was added. The mixture was left for 16 h at r.t. and a precipitate of C_{60}@[10]CPP was filtered off. In a first

chromatographic workup by preparative thin-layer chromatography (silica, toluene/chloroform 2:1), the product containing fraction could be separated from excess C_{60}, starting material and in some cases intramolecular Glaser side-product. The final purity was obtained by preparative HPLC (Buckyprep-M, toluene 0.5 mL/min, 40 °C).

Yields were obtained by quantitative NMR using 1,2,4,5-tetramethylbenzene as internal standard and integrating the catenanes methyl ester signals versus the aromatic protons of the internal standard. The yields are calculated from the bis-adducts and account for the stoichiometry in the product. No melting points were measured due to low amount of the products.

Table S1: Yields of isolated [2]catenanes and macrocyclic side-products from intramolecular Glaser coupling for the catenation reactions of all isolated *trans*-1, *trans*-2 and *trans*-3 bis-adducts **3a-g**. Yields given, were obtained from quantitative NMR (qNMR) with 1,2,4,5-tetramethylbenzene as internal standard with calculated masses in brackets.

Bis-adduct	Isomer	[2]Catenanes (**1**)	Macrocycles (**4**)
**3a**	*out,out-trans*-1	**1a**, 2 nmol (6 μg), 0.4%	**4a**, 23 nmol (27 μg), 2%
**3b**	*in*,*out-trans*-2	**1b**, 5.4 nmol (17 μg), 0.2%[a]	traces
**3c**	*out,out-trans*-2	not found	**4c**, 45 nmol (54 μg),1.6%
**3d**	*in,in-trans*-2	**1d**, 62 nmol (0.19 mg), 5%	not found
**3e**	*in*,*out-trans*-3	traces	**4e**, 1.7 mg, 1.5 μmol, 15%[b]
**3f**	*out*,*out-trans*-3	traces	not found
**3g**	*in*,*in-trans*-3	**1g**, 0.12 μmol (0.36 mg), 3%	not found

[a] only one of two isomers could be obtained clean enough for assignment of its key resonances and subsequent qNMR spectrometric determination of its yield. [b] Yield determined by weight.

[2]Catenane **1a**:

Prepared according to the general procedure.

3a (1 mg, 1 µmol, 2 equiv.), [10]CPP (0.4 mg, 0.5µmol, 1 equiv.), CuCl (0.02 g, 0.2 mmol, 340 equiv.), $CuCl_2$ (2 mg, 0.02 µmol, 35 equiv.), pyridine (3 mL).

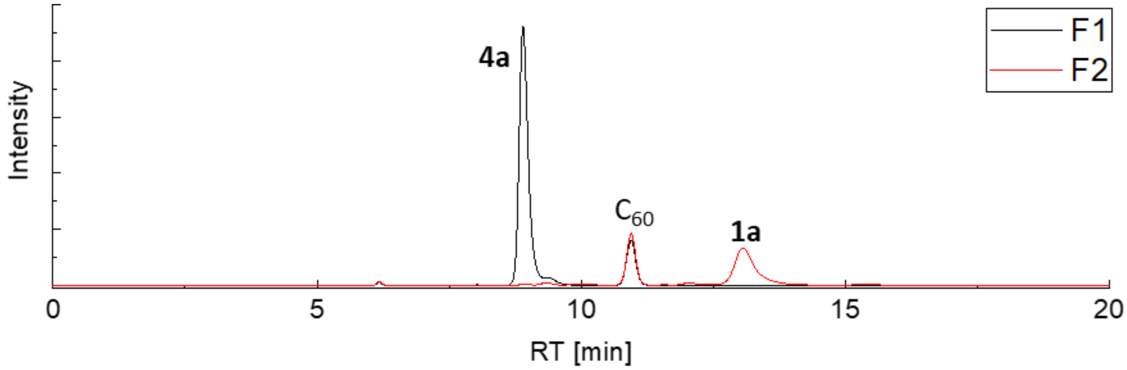

Figure S25: HPLC chromatogram stack plot of fractions 1 and 2 (F1 and F2) obtained from preparative TLC purification of the crude reaction mixture after removal of [10]CPP by precipitation with C_{60} in toluene.

1a:

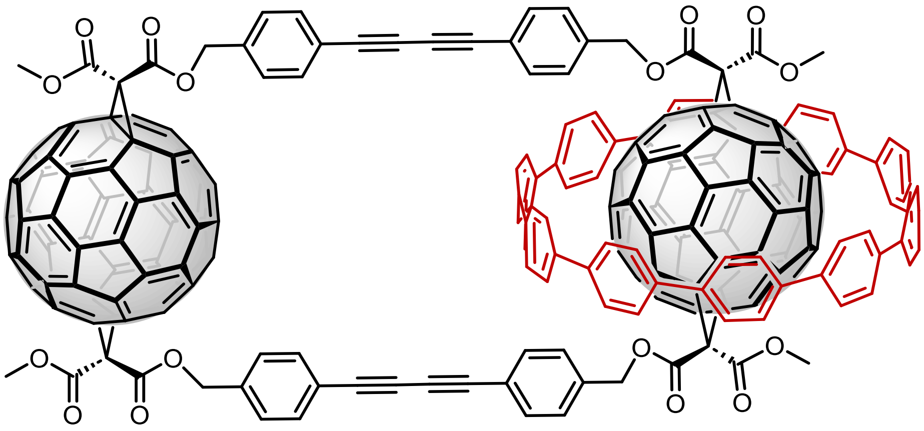

^{1}H NMR (600 MHz, $CDCl_3$, 263 K): δ = 7.67 (m, 8H, H_{Ar}), 7.50 (m, 8H, HAr), 5.69 (s, 4H, H_{Benzyl}), 5.57 (s, 4H, H_{Benzyl}), 4.29 (s, 6H, -OCH_3), 3.79 (s, 6H, -OCH_3) ppm.

LRMS (MALDI-TOF): found m/z = 3116.5155; calc. for $C_{232}H_{76}O_{16}$: 3116.5133; Δm/m = 0.71 ppm.

Yield: 2 nmol (6 μg), 0.4%

4a:

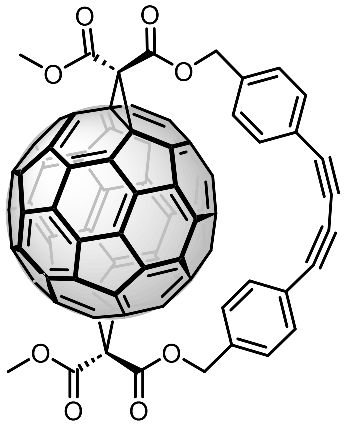

^{1}**H NMR** (600 MHz, $CDCl_3$, 298 K): δ = 7.53 (d, ^{3}J = 8.4 Hz, 4H, H_{Ar}), 7.36 (d, ^{3}J = 8.4 Hz, 4H, H_{Ar}), 5.65 (s, 4H, H_{Benzyl}), 4.20 (s, 6H, -OCH_3) ppm.

HRMS (MALDI): found m/z = 1178.0996; calc. for $C_{86}H_{18}O_8$: 1178.1002; Δm/m = 0.51 ppm.

Yield: 23 nmol (27 μg), 2%

[2]Catenane **1b**:

Prepared according to the general procedure.

3b (6.5 mg, 5.5 μmol, 2.0 equiv.), [10]CPP (2.2 mg, 2.9 μmol, 1.0 equiv.), CuCl (96 mg, 350 equiv.), $CuCl_2$ (12 mg, 89 μmol, 32 equiv.), pyridine (15 mL).

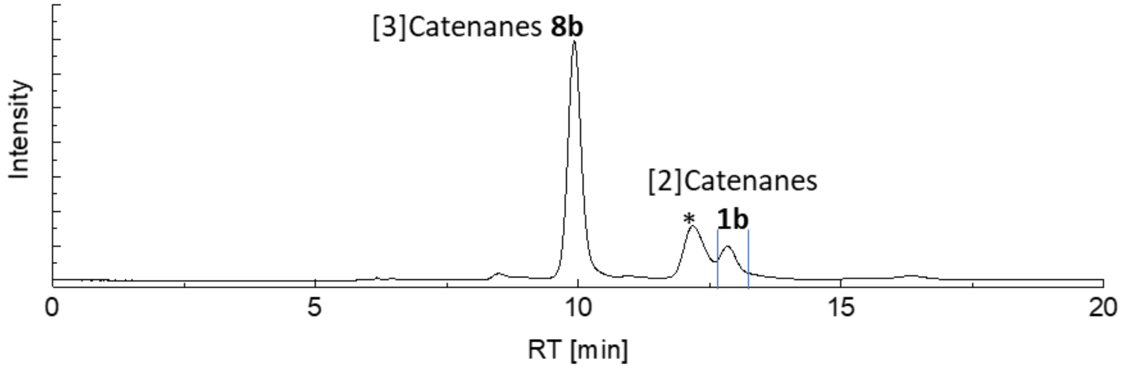

Figure S26: HPLC chromatogram of the [2]catenane containing fraction obtained from preparative TLC purification of the crude reaction mixture after removal of [10]CPP by precipitation with C_{60} in toluene. *key resonances could not be assigned for this isomer and therefore yield could not be determined by qNMR.

1b:

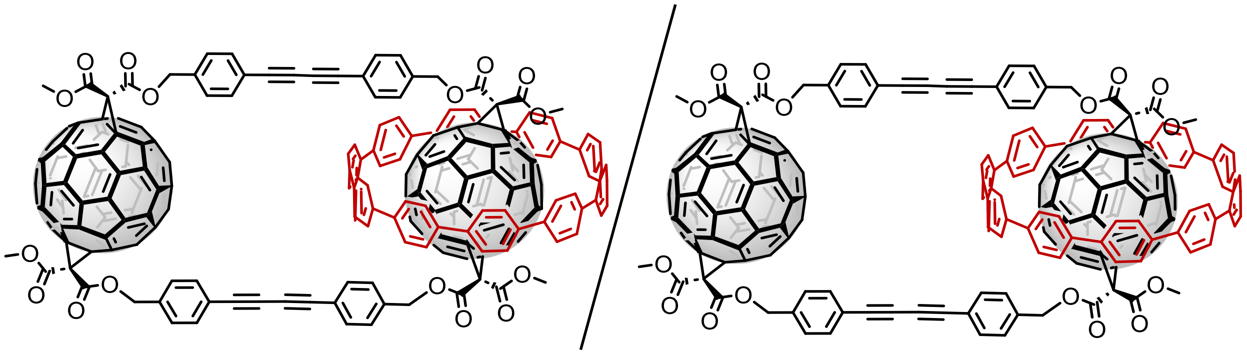

^{1}H NMR (600 MHz, $CDCl_3$, 273 K):

Selected resonances: δ = 7.41 – 7.37 (m, 40H, [10]CPP), 4.43 (s, 3H, -OCH_3), 4.24 (s, 3H, -OCH_3), 4.10 (s, 3H, -OCH_3), 3.72 (s, 3H, -OCH_3) ppm.

Due to the low concentration and spectral overlap with trace impurities, full assignment of all resonances was not possible. However, key diagnostic signals are visible and consistent with similar compounds. Structural identity is further supported by high-resolution mass spectrometry.

HRMS (MALDI): found m/z = 3116.5214; calc. for $C_{232}H_{76}O_{16}$: 3116.5133; Δm/m = 2.6 ppm.

Yield: 5.4 nmol (17 µg), 0.2%

8b:

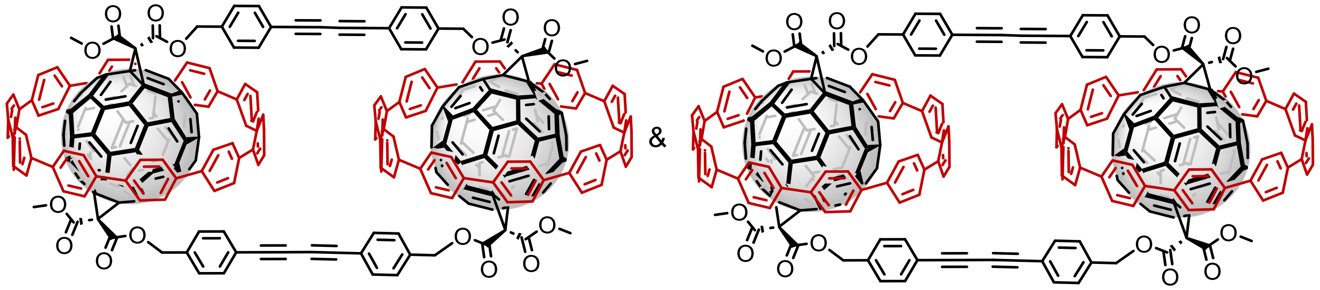

^{1}H NMR (600 MHz, $CDCl_3$, 298 K):

Selected resonances: δ = 7.68 – 7.63 (m, 12H, H_{Ar}), 7.48 – 7.46 (m, 4H, H_{Ar}), 7.40 – 7.37 (m, 80H, [10]CPP), 5.79 (d, *J* = 11.8 Hz, 1H, H_{Benzyl}), 5.63 (d, *J* = 13.0 Hz, 1H, H_{Benzyl}), 5.46 (d, *J* = 11.8 Hz, 1H, H_{Benzyl}), 5.42 (d, *J* = 13.09 Hz, 1H, H_{Benzyl}), 4.28 (s, 3H, $-OCH_3$), 4.27 (s, 3H, $-OCH_3$), 4.07 (s, 3H, $-OCH_3$), 4.02 (s, 3H, $-OCH_3$) ppm.

Due to the low concentration and spectral overlap with trace impurities, one benzylic proton resonance could not be assigned. However, key diagnostic signals are visible and consistent with similar compounds. Structural identity is further supported by high-resolution mass spectrometry.

HRMS (MALDI): found m/z = 3876.8345; calc. for $C_{292}H_{116}O_{16}$: 3876.8263; Δm/m = 2.1 ppm.

Yield: 6.2 nmol (24 µg), 0.5%

Attempted catenation of **3c**:

Reaction performed according to the general procedure.

3c (3.5 mg, 2.9 µmol, 2.0 equiv.), [10]CPP (1.2 mg, 1.5 µmol, 1.0 equiv.), CuCl (55 mg, 0.55 mmol, 370 equiv.), $CuCl_2$ (7.1 mg, 53 µmol, 36 equiv.), pyridine (8 mL).

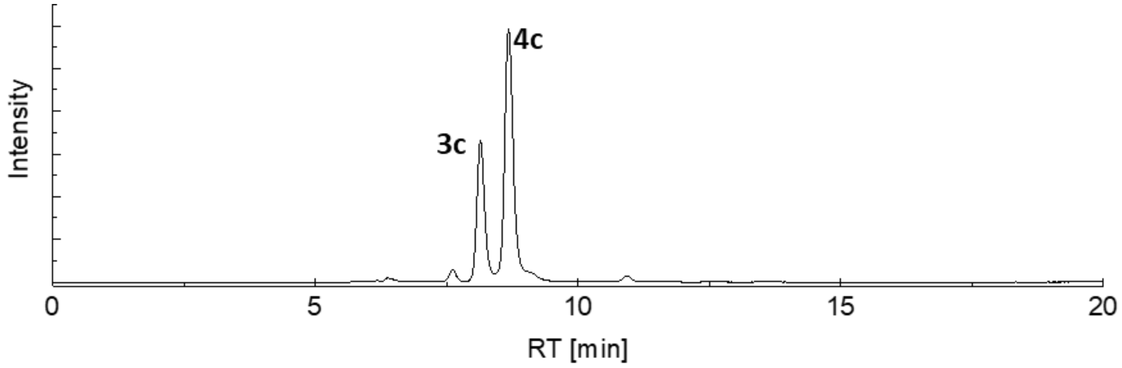

Figure S27: HPLC chromatogram of the main fraction obtained from preparative TLC purification of the crude reaction mixture after removal of [10]CPP by precipitation with C_{60} in toluene.

4c:

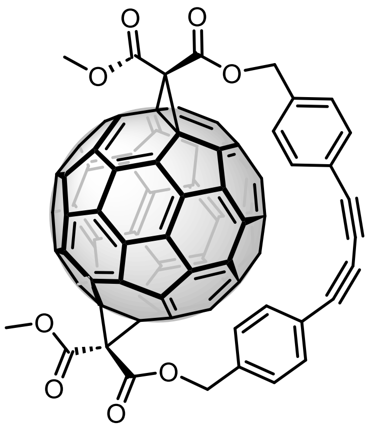

^{1}H NMR (600 MHz, $CDCl_3$, 298 K): δ = 7.47 (d, ^3J = 8.1 Hz, 4H, H_{Ar}), 7.33 (d, ^3J = 8.2 Hz, 4H, H_{Ar}), 5.49 (d, ^2J = 11.2 Hz, 2H, H_{Benzyl}), 5.38 (d, ^2J = 11.2 Hz, 2H, H_{Benzyl}), 4.24 (s, 6H, -OCH_3) ppm.

HRMS (MALDI): found m/z = 1178.0996; calc. for $C_{86}H_{18}O_8$: 1178.1002; Δm/m = 0.51 ppm.

Yield: 45 nmol (54 µg),1.6%

[2]Catenane **1d**:

Prepared according to the general procedure.

3d (3.0 mg, 2.5 mmol, 2.0 equiv.), [10]CPP (1.1 mg, 1.4 µmol, 1.2 equiv.), CuCl (43 mg, 0.43 mmol, 340 equiv.), $CuCl_2$ (6.0 mg, 45 µmol, 35 equiv.), pyridine (8 mL).

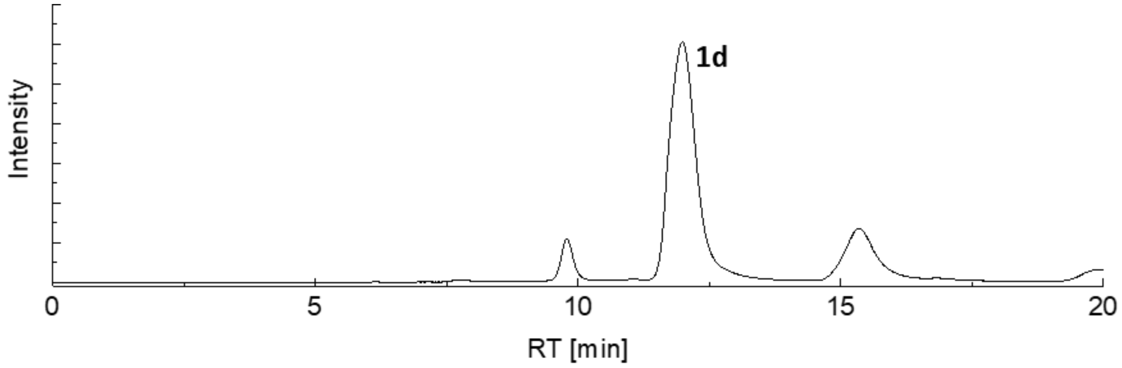

Figure S28: HPLC chromatogram of the [2]catenane containing fraction obtained from preparative TLC purification of the crude reaction mixture after removal of [10]CPP by precipitation with C_{60} in toluene.

1d:

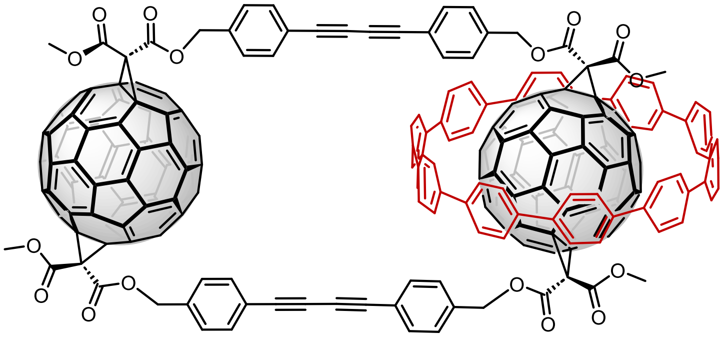

^{1}H NMR (600 MHz, $CDCl_3$, 263 K): δ = 7.74 – 7.52 (m, 16H, H_{Ar}), 7.43 (s, 40H, [10]CPP), 5.86 (d, J = 12.21 Hz, 1H, H_{Benzyl}), 5.66 – 5.62 (m, 4H, H_{Benzyl}), 5.56 – 5.54 (m, 2H, H_{Benzyl}), 5.49 (d, J = 12.24 Hz, 1H, H_{Benzyl}), 4.23 (s, 3H, -OCH_3), 4.21 (s, 3H, -OCH_3), 4.06 (s, 3H, -OCH_3), 4.05 (s, 3H, -OCH_3) ppm.

HRMS (MALDI): found m/z = 3116.5139; calc. for $C_{232}H_{76}O_{16}$: 3116.5133; Δm/m = 0.19 ppm.

Yield: 62 nmol (0.19 mg), 5%

Attempted catenation of **3e**:

Reaction performed according to the general procedure.

3e (12 mg, 9.9 µmol, 2.0 equiv.), [10]CPP (3.8 mg, 5.0 µmol, 1.0 equiv.), CuCl (0.18 g, 1.8 mmol, 360 equiv.), $CuCl_2$ (53 mg, 0.20 mmol, 40 equiv.), pyridine (30 mL).

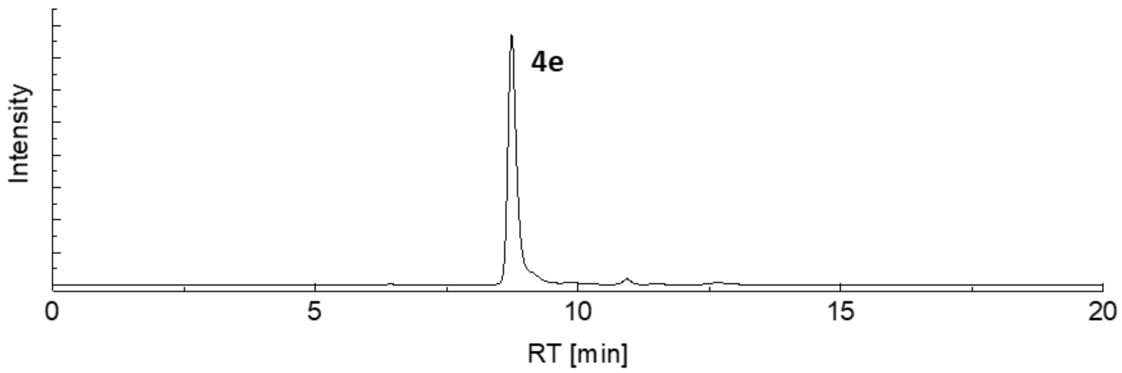

Figure S29: HPLC chromatogram of the main fraction obtained from preparative TLC purification of the crude reaction mixture after removal of [10]CPP by precipitation with C_{60} in toluene.

4e:

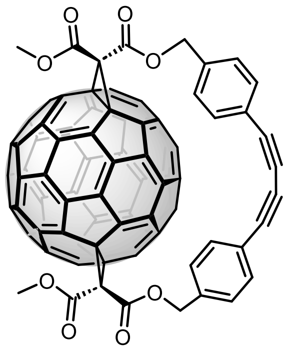

¹H NMR (600 MHz, CD_2Cl_2, 298 K): δ = 7.65 – 7.59 (m, 2H, H_{Ar}), 7.45 (d, ^3J = 8.4 Hz, 2H, H_{Ar}), 7.40 (d, ^3J = 8.3 Hz, 2H, H_{Ar}), 7.27 – 7.25 (m, 2H, H_{Ar}), 5.82 (d, J = 10.8 Hz, 1H, H_{Benzyl}), 5.78 (d, J = 11.0 Hz, 1H, H_{Benzyl}), 5.37 (d, J = 10.7 Hz, 1H, H_{Benzyl}), 5.07 (d, J = 11.0 Hz, 1H, H_{Benzyl}), 4.07 (s, 3H, -OCH_3), 3.97 (s, 3H, -OCH_3) ppm.

HRMS (MALDI): found m/z = 1178.1011; calc. for $C_{86}H_{18}O_8$: 1178.1002; Δm/m = 0.76 ppm.

Yield: 1.7 mg, 1.5 µmol, 15%

[2]Catenane **1g**:

Prepared according to the general procedure.

3g (9.6 mg, 8.1 µmol, 2.0 equiv.), [10]CPP (3.3 mg, 4.3 µmol, 1.0 equiv.), CuCl (0.15 g, 1.5 mmol, 370 equiv.), $CuCl_2$ (20 mg, 0.15 mmol, 36 equiv.), pyridine (24 mL).

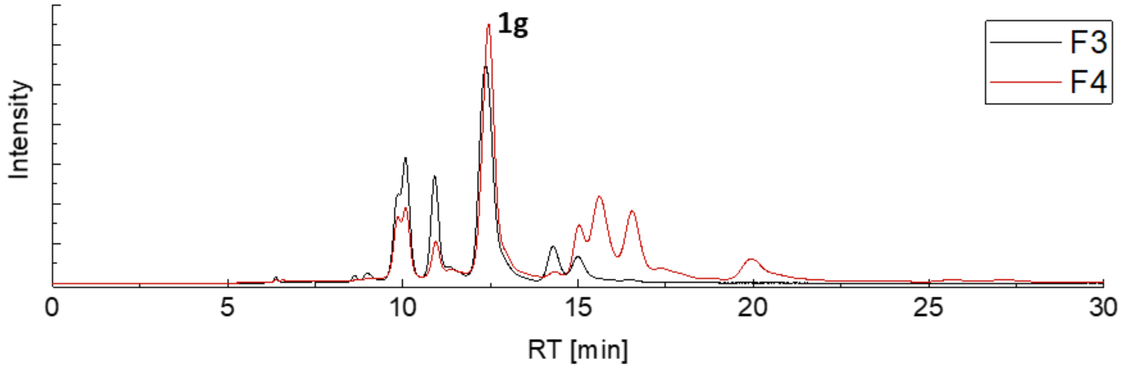

Figure S30: HPLC chromatogram stack plot of the [2]catenane containing fractions 3 and 4 (F3 and F4) obtained from preparative TLC purification of the crude reaction mixture after removal of [10]CPP by precipitation with C_{60} in toluene.

1g:

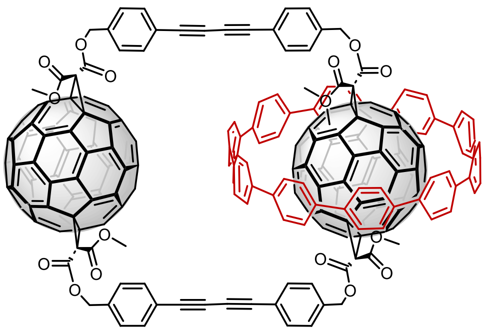

^{1}H NMR (600 MHz, $CDCl_3$, 253 K): δ = 7.72 (d, ^3J = 8.1 Hz, 4H, H_{Ar}), 7.61 (d, ^3J = 7.9 Hz, 4H, H_{Ar}), 7.56 – 7.51 (m, 4H, H_{Ar}), 7.44 – 7.42 (m, 4H, H_{Ar}), 7.35 – 7.28 (m, 40H, [10]CPP), 5.71 – 5.61 (m, 2H, H_{Benzyl}), 5.58 – 5.50 (m, 4H, H_{Benzyl}), 5.48 – 5.38 (m, 2H, H_{Benzyl}), 4.10 (s, 3H, -OCH_3), 4.10 (s, 3H, -OCH_3), 3.84 (s, 3H, -OCH_3), 3.82 (s, 3H, -OCH_3) ppm.

HRMS (MALDI): found m/z = 3116.5248; calc. for $C_{232}H_{76}O_{16}$: 3116.5133; Δm/m = 3.7 ppm.

Yield: 0.12 µmol (0.36 mg), 3%

2.4. Sequential synthesis of a C_{60}–C_{70} dyad *via* Bingel and Bingel–Hirsch reactions

We initially explored an alternative strategy for synthesizing catenanes **1**, leveraging the orthogonality between the Bingel and Bingel–Hirsch reactions. Although a sequential approach of this kind has previously been investigated by Hirsch and co-workers for the preparation of fullerene hexakis-adducts, extended reaction times were found to promote side reactions, such as bromide scrambling. To mitigate this, the authors employed less reactive chloromalonates instead.[S5] In our case, however, the rapid conversion of C_{60} and C_{70} to their corresponding mono-adducts using bromomalonates enabled us to perform this key step efficiently.

Although this route ultimately proved unsuitable for constructing [2]catenanes, we consider the strategy elegant and report it here for its conceptual merit.

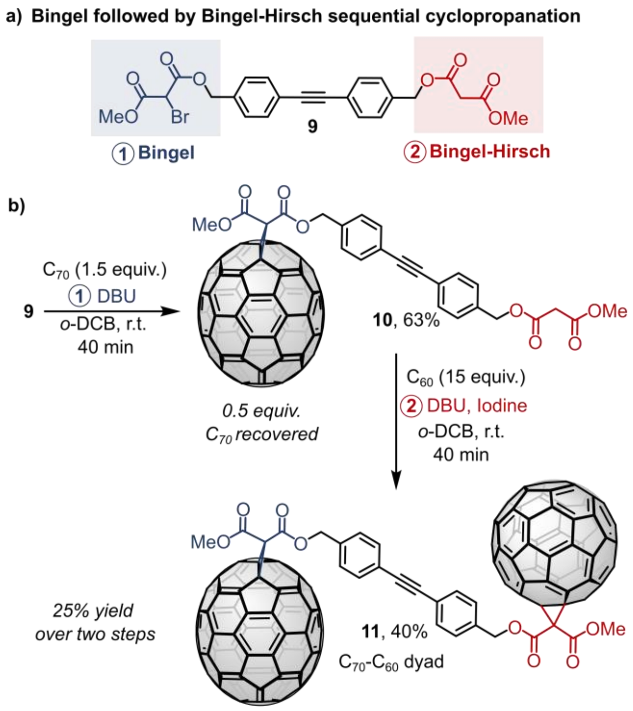

Figure 31: a) Structure of desymmetrised linker **9**, highlighting the orthogonal reactivity of the bromomalonate (blue) and malonate (red) groups under Bingel and Bingel–Hirsch conditions, respectively. b) Demonstration of the sequential functionalisation in the facile synthesis of C_{70}–C_{60} dyad **11**.

We chose to demonstrate the concept with the synthesis of an unsymmetric C_{70}–C_{60} dyad by reacting the desymmetrised bis-malonate linker **9** (obtained in 9% yield over three steps) sequentially with two different fullerenes (Figure S31). The first step, a Bingel cyclopropanation with C_{70}, proceeded selectively with the bromomalonate to give the C_{70} mono-adduct **10** in 63% yield. The reaction afforded a single regioisomer, which we tentatively assign as the *α*-C_{70} mono-adduct.[S6] In the second step, the "dormant"/non-brominated malonate underwent a Bingel–Hirsch reaction with C_{60} in the presence of base and iodine (the latter reagent generates an iodo-malonate *in situ*[S7]). This step yielded the unsymmetric C_{70}–C_{60} dyad **11** in 25% yield over two steps that can easily be conducted within a single day. While syntheses of C_{70}–C_{60} dyads have been reported previously, they often rely on two different cyclopropanation reactions introducing distinct addends on each fullerene to achieve orthogonality,[S8] or require the use of protecting groups to enforce selective reactivity.[S9]

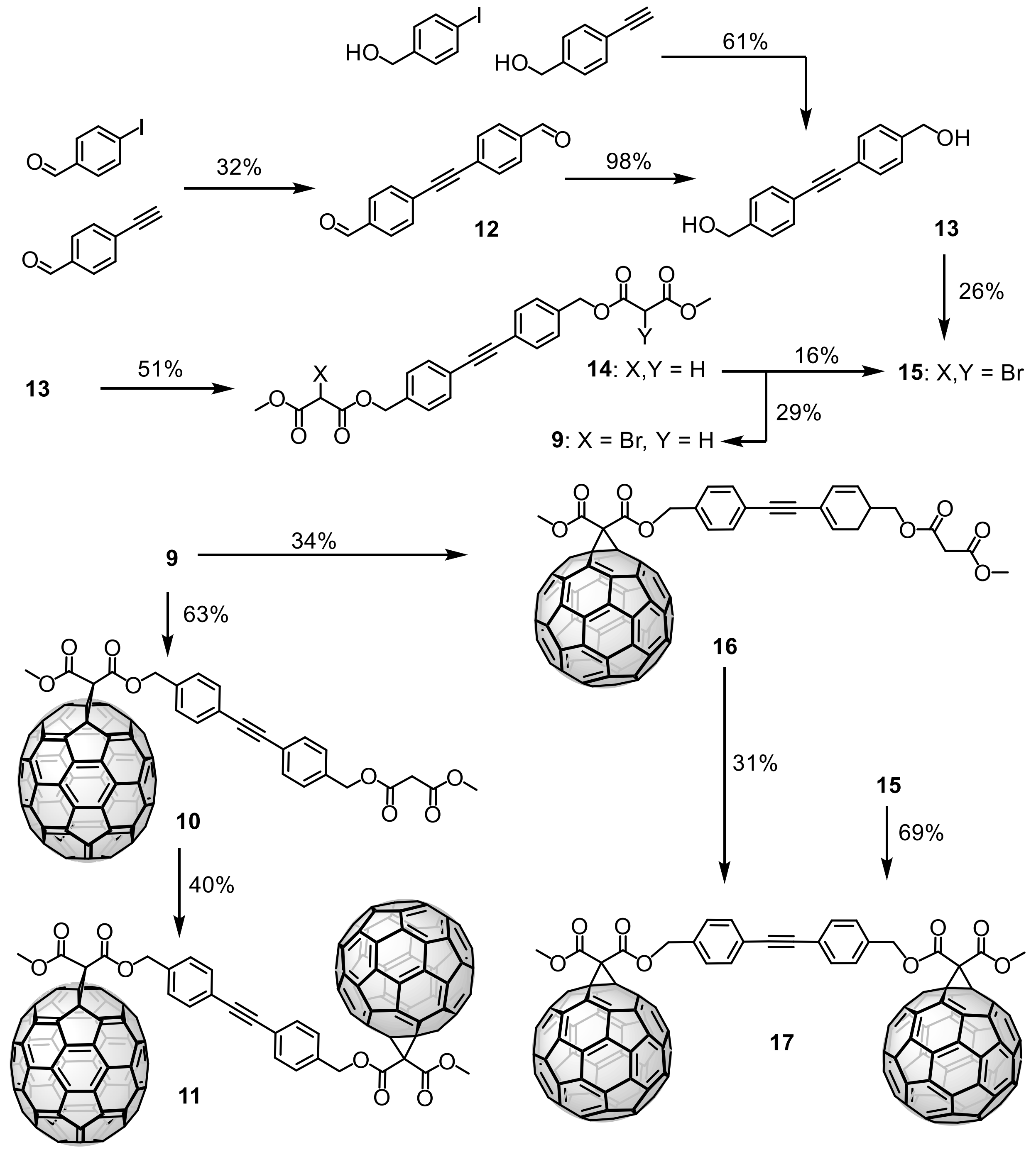

Scheme S2: Overview of synthetic pathway for the C_{60}-C_{70} dyad **11** and related molecules.

Synthesis of di(4-formylphenyl)ethyne (12)

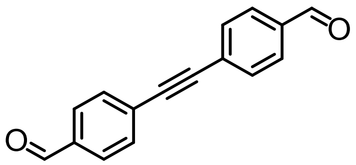

Following published general procedure.[S10]

To a solution of 4-iodobenzaldehyde (0.19 g, 0.80 mmol, 1.0 equiv.) in degassed anh. DMF (20 mL) was added bis(triphenylphosphine)palladium(II) chloride (30 mg, 43 µmol, 0.055 equiv.), copper(I) iodide (2.6 mg, 14 µmol, 0.018 equiv.) and triethyl amine (0.35 mL, 2.5 mmol, 3.2 equiv.). The reaction mixture was stirred for 10 min before 4-ethynylbenzaldehyde (0.10 g, 0.77 mmol, 1.0 equiv.) was added and the reaction was stirred at room temperature in the dark for 19 h. The reaction was stopped by addition of aqueous ammonium chloride solution and the aqueous phase was extracted with DCM (4 x 50 mL). The combined organic phases were dried over sodium sulfate, filtered, and evaporated. After sublimation of residual starting material (60 °C, 0.15 mbar) the product **12** could be sublimated (140 °C, 0.15 mbar) as a colorless solid (59 mg, 0.25 mmol, 32%).

^{1}H NMR (600 MHz, $CDCl_3$, 298 K): δ = 10.04 (s, 2H, $H_{Aldehyde}$), 7.90 (d, ^3J = 8.26 Hz, 4H, H_{Ar}), 7.71 (d, ^3J = 8.17 Hz, 4H, H_{Ar}) ppm.

^{13}C NMR (151 MHz, $CDCl_3$, 298 K): δ = 191.5, 136.1, 132.5, 129.8, 128.8, 92.3 ppm.

NMR data conforms to literature.[S11]

Synthesis of di(4-hydroxymethylphenyl)ethyne (13)

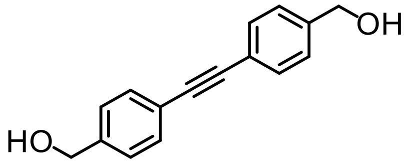

Procedure A:

To a solution of sodium tetrahydroborate (11 mg, 0.29 mmol, 1.2 equiv.) in anh. ethanol (20 mL) was added a suspension of the dialdehyde **12** (57 mg, 0.24 mmol, 1.0 equiv.) in anh. ethanol (5 mL) at 0 °C. The reaction was stirred for 50 min before the reaction was quenched by addition of 2 mL of 2N HCl solution. The reaction mixture was stirred for 30 more minutes at 0 °C. Water was added and the aqueous phase was extracted with diethyl ether (3 x 50 mL), the organic phases were combined, dried over sodium sulfate, filtered and evaporated to yield the product **13** (56 mg, 0.24 mmol, 98%) as a colourless solid.

Procedure B:

Following published general procedure.[S10]

To a solution of (4-iodophenyl)methanol (2.0 g, 8.6 mmol, 1.1 equiv.) in degassed anh. DMF (200 mL) was added bis(triphenylphosphine)palladium(II) chloride (0.25 g, 0.36 mmol, 0.046 equiv.), copper(I) iodide (34 mg, 0.18 mmol, 0.022 equiv.) and triethyl amine (6.0 mL, 43 mmol, 5.5 equiv.). The reaction mixture was stirred for 10 min while degassing with nitrogen before (4-ethynylphenyl)methanol (0.99 g, 8.6 mmol, 1.0 equiv.) was added and the reaction was stirred at room temperature in the dark for 29 h. The reaction mixture was poured onto 5% HCl (700 mL) and the formed precipitate was filtered off. The pure product **13** was obtained by recrystallization from acetonitrile as a yellow solid (1.1 g, 4.8 mmol, 61%).

^{1}H NMR (400 MHz, DMSO, 298 K): δ = 7.50 (d, ^3J = 8.2 Hz 4H, H_{Ar}), 7.36 (d, ^3J = 8.1 Hz, 4H, H_{Ar}), 5.29 (t, ^3J = 5.7 Hz, 2H, -O*H*), 4.53 (d, ^3J = 5.7 Hz, 4H, H_{benzyl}) ppm.

NMR conforms to literature data.[S12]

Synthesis of di(4-(3-methoxy-1,3-dioxopropoxymethyl)phenyl)ethyne (14)

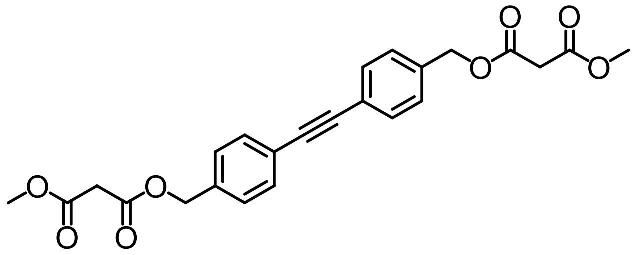

To a solution of the diol **13** (56 mg, 0.24 mmol, 1.0 equiv.) and triethyl amine (0.12 mL, 87 mg, 0.86 mmol, 3.6 equiv.) in anh. THF (20 mL) was added methyl malonyl chloride (65 µL, 82 mg, 0.61 mmol, 2.6 equiv.) dropwise at 0 °C. The reaction was stirred at 0 °C and monitored by thin layer chromatography (silica, petroleum spirit / acetone 3:1). After one hour more malonyl chloride (65 µL, 82 mg, 0.61 mmol, 2.6 equiv.) and triethyl amine (0.24 mL, 0.17 mg, 1.7 mmol, 7.3 equiv.) were added. After stirring at 0 °C for 30 more minutes the reaction was quenched by addition of saturated ammonium chloride solution. Brine was added and the aqueous phase was extracted with DCM (3 x 30 mL). The combined organic phases were dried over sodium sulfate, filtered, and evaporated. Pure product **14** was obtained by flash column chromatography (silica, petroleum spirit / acetone 3:1) as a colourless oil (53 mg, 0.12 mmol, 51%).

^{1}H NMR (600 MHz, $CDCl_3$, 298 K): δ = 7.53 (d, ^3J = 8.2 Hz, 4H, H_{Ar}), 7.34 (d, ^3J = 8.2 Hz, 4H, H_{Ar}), 5.20 (s, 4H, H_{benzyl}), 3.75 (s, 6H, -OCH_3), 3.45 (s, 4H, $H_{malonate}$) ppm.

^{13}C NMR (151 MHz, $CDCl_3$, 298 K): δ = 166.9, 166.4, 135.6, 132.0, 128.3, 123.4, 89.6, 66.9, 52.7, 41.5 ppm.

HRMS (APCI): found m/z = 438.1314; calc. for $C_{24}H_{22}BrO_8$: 438.1315; Δm/m = 0.23 ppm.

Synthesis of di(4-(2-bromo-3-methoxy-1,3-dioxopropoxymethyl)phenyl)ethyne (15)

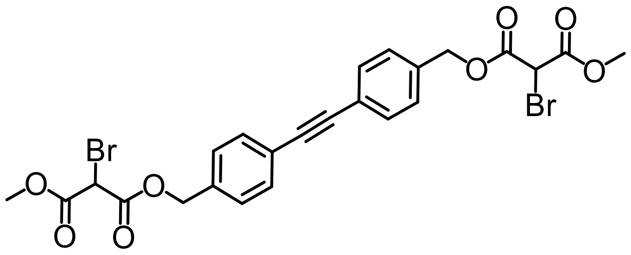

An oven-dried 25 mL pressure tube was charged with methyl hydrogen malonate (0.40 mL, 0.45 g, 3.5 mmol, 10 equiv.) and phosphorous tribromide (0.12 mL, 0.34 g, 1.3 mmol, 3.6 equiv.) and bromine (0.28 mL, 0.87 g, 5.4 mmol, 16 equiv.) and the reaction mixture was stirred at 100 °C for 4 h under nitrogen atmosphere. Excess bromine and formed hydrogen bromide were removed under reduced pressure and the reaction mixture was diluted with anh. THF (2 mL) and added dropwise to a stirred solution of diol **13** (84 mg, 0.35 mmol, 1.0 equiv.) and triethyl amine (0.50 mL, 0.36 g, 3.6 mmol, 10 equiv.) in anh. THF (20 mL) at 0 °C. The reaction was stopped after 1 h by addition of aqueous thiosulfate solution and the aqueous phase was extracted with diethyl ether (3 x 50 mL). The combined organic phases were dried over sodium sulfate, filtered, and evaporated. The product **15** was obtained after purification by flash column chromatography (silica, cyclohexane / ethyl acetate 4:1) as a colourless oil (55 mg, 91 µmol, 26%).

^{1}H NMR (600 MHz, $CDCl_3$, 298 K): δ = 7.56 – 7.51 (m, 4H, H_{Ar}), 7.37 – 7.32 (m, 4H, H_{Ar}), 5.26 (s, 4H, H_{Benzyl}), 4.90 (s, 2H, $H_{Bromomalonate}$), 3.81 (s, 6H, -OCH_3) ppm.

^{13}C NMR (151 MHz, $CDCl_3$, 298 K): δ = 164.9, 164.3, 134.8, 131.9, 128.2, 123.5, 89.6, 68.2, 54.0, 41.8 ppm.

Synthesis of methyl (4-(4-(3-methoxy-1,3-dioxopropoxymethyl)phenyl)ethynylbenzyl) 2-bromomalonate (9)

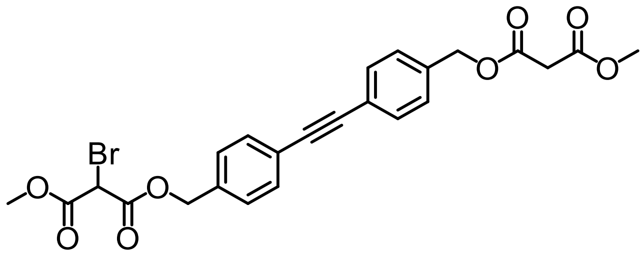

The reaction was performed following a modified literature procedure.[S13]

To a solution of dimalonate **14** (52 mg, 0.12 mmol, 1.0 equiv.) in anh. THF (10 mL) was added DBU (20 µL, 20 mg, 0.13 mmol, 1.1 equiv.) at 0 °C and the reaction mixture was allowed to warm to room temperature over 1 h. The reaction mixture was subsequently cooled to -78 °C and carbon tetrabromide (40 mg, 0.12 mmol, 1.0 equiv.) was added and the reaction progress was monitored by thin layer chromatography (petroleum spirit / ethyl acetate 2:1). The reaction was stirred for 6 h during which more carbon tetrabromide (22 mg, 66 µmol, 0.55 equiv.) and more DBU (10 µL, 10 mg, 67 µmol, 0.50 equiv.) was added to improve conversion. The reaction was stopped by addition of aqueous ammonium sulfate solution and the aqueous phase was extracted with diethyl ether (3 x 30 mL) and the combined organic phases were dried over sodium sulfate, filtered, and evaporated. The pure product **9** was obtained after flash column chromatography (silica, petroleum spirit / ethyl acetate 5:2) as a colourless solid (18 mg, 35 µmol, 29%).

Bisbromomalonate **15** (12 mg, 20 µmol, 16%) was obtained as a side product while some of the starting material **14** (12 mg, 27 µmol, 23%) could be recovered.

^{1}H NMR (600 MHz, $CDCl_3$, 298 K): δ = 7.53 (dd, ^3J = 8.2, 4.5 Hz, 4H; H_{Ar}), 7.40 – 7.31 (m, 4H, H_{Ar}), 5.26 (s, 2H, H_{Benzyl}), 5.20 (s, 2H, H_{Benzyl}), 4.90 (s, 1H, $H_{Bromomalonate}$), 3.82 (s, 3H, -OCH_3), 3.75 (s, 3H, -OCH_3), 3.45 (s, 2H, $H_{Malonate}$) ppm.

^{13}C **NMR** (151 MHz, $CDCl_3$, 298 K): δ = 166.9, 166.4, 165.0, 164.5, 132.0, 132.0, 128.4, 128.3, 123.7, 123.4, 89.8, 89.5, 68.4, 66.9, 54.1, 52.7. 41.9, 41.5 ppm.

HRMS (MALDI): found m/z = 516.0407; calc. for $C_{24}H_{21}BrO_8$: 516.0420; Δm/m = 2.5 ppm.

Synthesis of methyl 4-(4-(3-methoxy-1,3-dioxopropoxymethyl)phenylethynyl)benzyl malonate C_{60}MA (16)

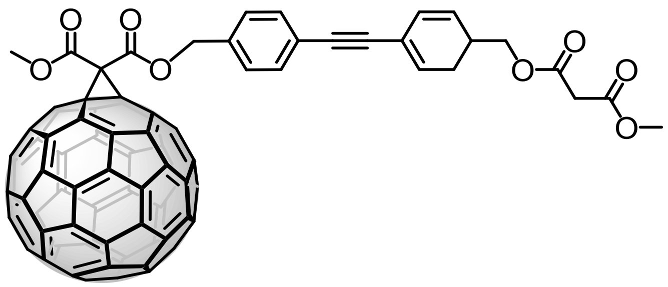

To a solution of C_{60} (11 mg, 15 µmol, 1.5 equiv.) in toluene (10 mL) was added a solution of bromomalonate **9** (5.2 mg, 10 µmol, 1.0 equiv.) in DCM (1 mL). DBU (5.0 µmol, 5.1 mg, 33 µmol, 3.3 equiv.) was added and the reaction mixture was stirred at room temperature for 1 h before the reaction was quenched by addition of saturated ammonium chloride solution. The organic phase was separated off, dried over sodium sulfate, filtered, and evaporated. The pure product **16** was obtained after flash column chromatography (silica, petroleum spirit / toluene 3:1 → toluene / ethyl acetate 20:1) as a brown solid (4.0 mg, 35 µmol, 34%).

^{1}H NMR (600 MHz, CD_2Cl_2, 298 K): δ = 7.61 – 7.49 (m, 6H, H_{Ar}), 7.37 (d, ^3J = 7.9 Hz, 2H, H_{Ar}), 5.53 (s, 2H, H_{benzyl}), 5.19 (s, 2H, H_{benzyl}), 4.03 (d, ^3J = 0.9 Hz, 3H, -OCH_3), 3.72 (d, ^3J = 0.8 Hz, 3H, -OCH_3), 3.44 (s, 2H, $H_{malonate}$) ppm.

^{13}C NMR (151 MHz, CD_2Cl_2, 298 K): δ = 167.2, 166.7, 164.2, 163.7, 145.7 (C_{60}-sp^2, 2C), 145.7 (C_{60}-sp^2, 4C), 145.6 (C_{60}-sp^2, 6C), 145.5 (C_{60}-sp^2, 2C), 145.4 (C_{60}-sp^2, 2C), 145.3 (C_{60}-sp^2, 2C), 145.1 (C_{60}-sp^2, 2C), 145.1 (C_{60}-sp^2, 3C), 145.0 (C_{60}-sp^2, 2C), 145.0 (C_{60}-sp^2, 1C), 145.0 (C_{60}-sp^2, 2C), 144.3 (C_{60}-sp^2, 2C), 144.3 (C_{60}-sp^2, 2C), 143.5 (C_{60}-sp^2, 2C), 143.4 (C_{60}-sp^2, 6C), 143.4 (C_{60}-sp^2, 2C), 142.6 (C_{60}-sp^2, 4C), 142.3 (C_{60}-sp^2, 4C), 141.4 (C_{60}-sp^2, 4C), 139.8 (C_{60}-sp^2, 2C), 139.2 (C_{60}-sp^2, 2C), 136.4, 135.6, 132.2, 132.1, 129.4, 128.5, 124.1, 123.4, 90.1, 89.7, 71.9 (C_{60}-sp^3, 2C), 68.8, 67.0, 54.4, 52.8, 41.7 ppm.

Due to overlap only 22 of the expected 32 carbon signals could be found.

HRMS (MALDI): found m/z = 1156.1155; calc. for $C_{84}H_{20}O_8$: 1156.1158; Δm/m = 0.26 ppm.

Synthesis of C_{60} dumbbell (17)

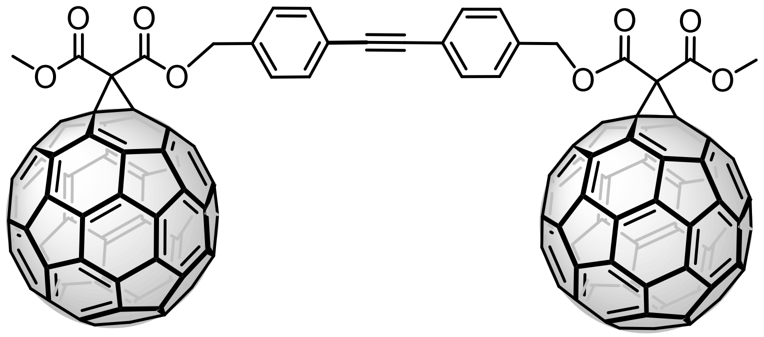

Method A:

To a solution of C_{60} (0.56 g, 0.78 mmol, 10 equiv.) in anh. 1,2-dichlorobenzene (100 mL) and the bisbromomalonate **15** (46 mg, 77 µmol, 1.0 equiv.) in 1,2-dichlorobenzene (2 mL) was added a solution of DBU (70 µL, 71 mg, 0.47 mmol, 6.1 equiv.) in 1,2-dichlorobenzene (10 mL) dropwise and the reaction mixture was continued to stir at room temperature for 5 h. The reaction mixture was filtered and concentrated under vacuum before and the product **17** was obtained after purification by flash column chromatography (silica, petroleum spirit / 1,2-dichlorobenzene 3:1 → 1,2-dichlorobenzene) as a red solution in 1,2-dichlorobenzene. The solution was concentrated under vacuum and the volume was adjusted to 50.0 mL in a volumetric flask. The yield was subsequently determined by qNMR with internal standard tetramethylbenzene in 1,2-dichlorobenzene (0.2 mL) and chloroform-d (0.3 mL): 53 µmol, 0.10 mg, 69%.

The compound was kept as a solution due to the inherently low solubility and poor redissolution behaviour.

For NMR analysis a portion of the product was evaporated and redissolved in deuterated 1,2-dichlorobenzene.

Method B:

To a solution of the mono-adduct **16** (2.5 mg, 2.2 µmol, 1.0 equiv.), C_{60} (9.9 mg, 14 µmol, 6.4 equiv.) and iodine (7.6 mg, 3.0 µmol, 1.4 equiv.) in anh. toluene (12 mL) was added DBU (1.0 µL, 1.0 mg, 6.7 µmol, 3.1 equiv.) and the reaction was stirred at room temperature for 40 minutes. The reaction was quenched by addition of aqueous ammonium sulfate solution and aqueous thiosulfate solution. The aqueous phase was extracted with toluene (3 x 10 mL) and the combined organic phases were dried over sodium sulfate, filtered, and evaporated. The product was obtained after flash column chromatography (silica, petroleum spirit / toluene 3:1 → toluene) as a brown solid (1.3 mg, 0.67 µmol, 31%).

^{1}H NMR (600 MHz, *o*-$C_6D_4Cl_2$, 298 K) δ = 7.55 (d, ^3J = 8.2 Hz, 4H), 7.41 (d, ^3J = 8.2 Hz, 4H), 5.48 (s, 4H, H_{Benzyl}), 3.95 (s, 6H, -CH_3) ppm.

^{13}C NMR (151 MHz, *o*-$C_6D_4Cl_2$, 298 K) δ = 163.6, 163.1, 145.2 (C_{60}-sp^2, 4C), 145.1 (C_{60}-sp^2, 6C), 145.0 (C_{60}-sp^2, 4C), 145.0 (C_{60}-sp^2, 4C), 145.0 (C_{60}-sp^2, 4C),145.0 (C_{60}-sp^2, 6C), 144.9 (C_{60}-sp^2, 4C), 144.7 (C_{60}-sp^2, 4C), 144.5 (C_{60}-sp^2, 12C), 144.4 (C_{60}-sp^2, 6C), 144.4 (C_{60}-sp^2, 4C), 144.4 (C_{60}-sp^2, 2C), 143.7 (C_{60}-sp^2, 8C), 142.8 (C_{60}-sp^2, 12C), 142.8 (C_{60}-sp^2, 4C), 142.0 (C_{60}-sp^2, 8C), 141.7 (C_{60}-sp^2, 4C), 141.7 (C_{60}-sp^2, 4C), 140.8 (C_{60}-sp^2, 4C), 140.8 (C_{60}-sp^2, 4C), 139.4 (C_{60}-sp^2, 4C), 138.9 (C_{60}-sp^2, 4C), 123.9, 71.6 (C_{60}-sp^3, 4C), 68.3, 53.6 ppm.

Due to overlap only 23 of the expected 32 fullerene carbon signals could be found. Only one aromatic carbon signal could be seen, due to overlap with the solvent.

HRMS (MALDI): found m/z = 1874.0957; calc. for $C_{144}H_{18}O_8$: 1874.1002; Δm/m = 2.4 ppm.

Synthesis of methyl 4-(4-(3-methoxy-1,3-dioxopropoxymethyl)phenylethynyl)benzyl malonate *α*-C_{70}MA (10)

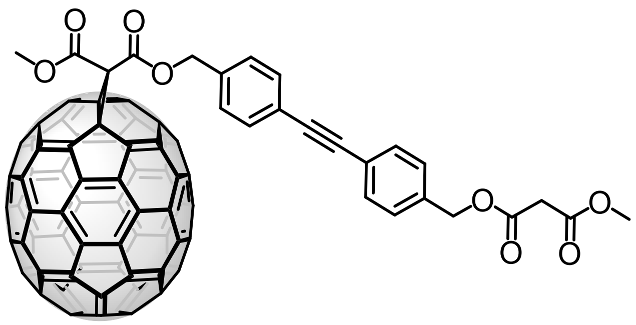

To a solution of C_{70} (10 mg, 12 µmol, 1.5 equiv.) in anh. 1,2-dichlorobenzene (10 mL) was added a solution of bromomalonate **9** (4.4 mg, 8.4 µmol, 1.0 equiv.) in anh. DCM (0.85 mL) and the reaction mixture was stirred for 5 minutes before DBU (10 µL, 10 mg, 67 µmol, 7.9 equiv.) was added and the reaction mixture was continued to stir for 40 minutes. The reaction was quenched by addition of trifluoroacetic acid (10 µL, 15 mg, 0.13 mmol, 16 equiv.), the crude mixture was purified by flash column chromatography (silica, petroleum spirit / toluene 2:1 → toluene /ethyl acetate 20:1) and the pure product **10** was obtained as a brown solid (6.8 mg, 5.3 µmol, 63%).

Note: Unreacted C_{70} (4.9 mg, 5.8 µmol, 47%) could be recovered after flash column chromatography.

^{1}H NMR (400 MHz, $CDCl_3$, 298 K): δ = 7.57 – 7.48 (m, 6H, H_{Ar}), 7.35 – 7.33 (m, 2H, H_{Ar}), 5.48 (s, 2H, H_{Benzyl}), 5.20 (s, 2H, H_{Benzyl}), 3.99 (s, 3H, H_{Methyl}), 3.75 (s, 3H, H_{Methyl}), 3.45 (s, 4H, $H_{Malonate}$) ppm.

^{13}C NMR (101 MHz, $CDCl_3$, 298 K) δ = 166.9, 166.4, 164.0, 163.3, 155.2 (C_{70}-sp^2, 1C), 155.1 (C_{70}-sp^2, 1C), 151.5 (C_{70}-sp^2, 2C), 151.4 (C_{70}-sp^2, 1C), 151.3 (C_{70}-sp^2, 1C), 151.3 (C_{70},-sp^2, 1C), 150.9 (C_{70}-sp^2, 1C), 150.8 (C_{70}-sp^2, 1C), 150.7 (C_{70}-sp^2, 2C), 149.5 (C_{70}-sp^2, 1C), 149.5 (C_{70}-sp^2, 1C), 149.4 (C_{70}-sp^2, 2C), 149.3 (C_{70}-sp^2, 1C), 149.2 (C_{70}-sp^2, 1C), 148.9 (C_{70}-sp^2, 1C) 148.8 (C_{70}-sp^2, 1C), 148.7 (C_{70}-sp^2, 1C) 148.6 (C_{70}-sp^2, 3C), 148.6 (C_{70}-sp^2, 2C), 147.8 (C_{70}-sp^2, 1C), 147.7 (C_{70}-sp^2, 1C), 147.7 (C_{70}-sp^2, 1C), 147.6 (C_{70}-sp^2, 1C), 147.4 (C_{70}-sp^2, 2C), 147.2 (C_{70}-

sp^2, 1C), 147.1 (C_{70}-sp^2, 1C), 146.6 (C_{70}-sp^2, 1C), 146.1 (C_{70}-sp^2, 1C), 146.1 (C_{70}-sp^2, 1C), 146.0 (C_{70}-sp^2, 1C), 146.0 (C_{70}-sp^2, 1C), 145.1 (C_{70}-sp^2, 1C), 144.9 (C_{70}-sp^2, 1C), 144.1 (C_{70}-sp^2, 2C), 144.0 (C_{70}-sp^2, 2C), 143.7 (C_{70}-sp^2, 2C), 143.0 (C_{70}-sp^2, 1C), 143.0 (C_{70}-sp^2, 1C), 142.9 (C_{70}-sp^2, 1C), 142.7 (C_{70}-sp^2, 1C), 142.6 (C_{70}-sp^2, 1C) 142.1 (C_{70}-sp^2, 1C), 141.8 (C_{70}-sp^2, 1C), 141.7 (C_{70}-sp^2, 1C), 141.3 (C_{70}-sp^2, 1C), 140.7 (C_{70}-sp^2, 1C), 137.0 (C_{70}-sp^2, 1C), 136.7 (C_{70}-sp^2, 1C), 135.7, 134.8, 133.7 (C_{70}-sp^2, 2C), 132.9 (C_{70}-sp^2, 2C), 132.1, 132.0, 131.1 (C_{70}-sp^2, 1C), 131.1 (C_{70}-sp^2, 2C), 131.0 (C_{70}-sp^2, 1C), 130.9 (C_{70}-sp^2, 2C), 129.2, 128.3, 124.1, 123.3, 90.0, 89.5, 68.6, 66.9, 66.9 (C_{70}-sp^3, 1C), 66.33 (C_{70}-sp^3, 1C), 54.21, 52.74, 41.46, 36.86 ppm.

Due to spectral overlap, 56 out of 70 expected fullerene resonances could be identified, consistent with a mono-adduct exhibiting C_1 symmetry. Among the most reactive double bonds on C_{70}—the α- and β-positions—only the α-mono-adduct possesses C_1 symmetry. Based on this symmetry match, the product was assigned as the α-mono-adduct.[S14]

HRMS (MALDI): found m/z = 1276.1122; calc. for $C_{94}H_{20}O_8$: 1276.1158; Δm/m = 2.8 ppm.

Synthesis of C_{70}-C_{60} Dyad (11)

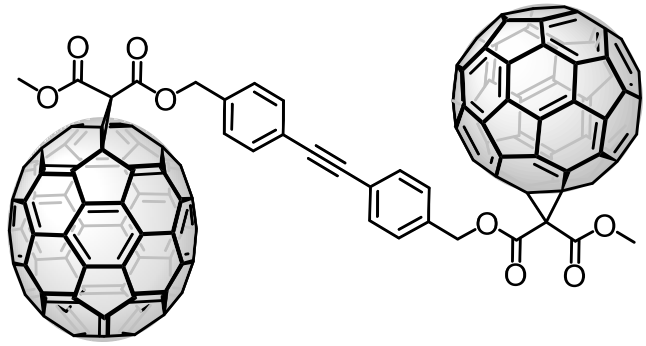

To a solution of the mono-adduct **10** (5.7 mg, 4.5 µmol, 1.0 equiv.), iodine (1.2 mg, 4.7 µmol, 1.0 equiv.) and C_{60} (49 mg, 68 µmol, 15 equiv.) in anh. 1,2-dichlorobenzene (5 mL) was added DBU (3.0 µL, 3.1 mg, 20 µmol, 4.5 equiv.) and the reaction was stirred at room temperature for 40 minutes. The reaction mixture was purified by flash column chromatography (silica, petroleum spirit / 1,2-dichlorobenzene 3:1 → petroleum spirit / toluene 2:1 → toluene) and the product **11** was obtained as a brown solid (3.6 mg, 1.8 µmol, 40%).

^{1}H NMR (600 MHz, $CDCl_3$, 298 K): δ = 7.58 – 7.52 (m, 4H, H_{Ar}), 7.50 – 7.48 (m, 4H, H_{Ar}), 5.53 (s, 2H, $H_{Benzyl\text{-}C60}$), 5.52 – 5.46 (m, 4H, $H_{Benzyl\text{-}C70}$), 4.04 (s, 3H, -OCH_3), 4.00 (s, 3H, -OCH_3) ppm.

^{13}C NMR (151 MHz, $CDCl_3$, 298 K): δ = 164.10, 164.02, 163.52, 163.31, 155.23 (C_{70}-sp^2, 1C), 155.05 (C_{70}-sp^2, 1C), 151.50 (C_{70}-sp^2, 2C), 151.44 (C_{70}-sp^2, 1C), 151.31 (C_{70}-sp^2, 2C), 150.89 (C_{70}-sp^2, 1C), 150.83 (C_{70}-sp^2, 1C), 150.72 (C_{70}-sp^2, 2C), 149.49 (C_{70}-sp^2, 2C), 149.41 (C_{70}-sp^2, 2C), 149.28 (C_{70}-sp^2, 1C), 149.25 (C_{70}-sp^2, 1C), 148.87 (C_{70}-sp^2, 1C), 148.85 (C_{70}-sp^2, 1C), 148.72 (C_{70}-sp^2, 1C), 148.65 (C_{70}-sp^2, 2C), 148.62 (C_{70}-sp^2, 3C), 147.77 (C_{70}-sp^2, 1C), 147.72 (C_{70}-sp^2, 1C), 147.66 (C_{70}-sp^2, 2C), 147.44 (C_{70}-sp^2, 2C), 147.16 (C_{70}-sp^2, 1C), 147.13 (C_{70}-sp^2, 1C), 146.61 (C_{70}-sp^2, 1C), 146.12 (C_{70}-sp^2, 1C), 146.06 (C_{70}-sp^2, 2C), 146.03 (C_{70}-sp^2, 1C), 145.44 (C_{60}-sp^2, 2C), 145.42 (C_{60}-sp^2, 2C), 145.35 (C_{60}-sp^2, 6C), 145.26 (C_{60}-sp^2, 2C), 145.15 (C_{60}-sp^2, 2C), 145.11 (C_{70}-sp^3, 1C), 145.08 (C_{60}-sp^2, 2C), 144.97 (C_{60}-sp^2, 2C), 144.87 (C_{70}/C_{60}-sp^2, 2C), 144.85 (C_{60}-sp^2, 3C), 144.82 (C_{60}-sp^2, 3C), 144.75 (C_{60}-sp^2, 2C), 144.69 (C_{60}-sp^2, 1C), 144.10 (C_{70}-sp^3, 2C), 144.06 (C_{60}-sp^2, 2C), 144.03 (C_{60}-sp^2, 2C), 143.99 (C_{70}-sp^2, 2C), 143.70

(C_{70}-sp^3, 2C), 143.27 (C_{60}-sp^2, 2C), 143.19 (C_{60}-sp^2, 6C), 143.14 (C_{60}-sp^2, 2C), 143.02 (C_{70}-sp^2, 1C), 142.98 (C_{70}-sp^2, 1C), 142.92 (C_{70}-sp^2, 1C), 142.74 (C_{70}-sp^2, 1C), 142.58 (C_{70}-sp^2, 1C), 142.37 (C_{60}-sp^2, 4C), 142.04 (C_{70}/C_{60}-sp^2, 5C), 141.81 (C_{70}-sp^2, 1C), 141.74 (C_{70}-sp^2, 1C), 141.32 (C_{70}-sp^2, 1C), 141.14 (C_{60}-sp^2, 2C), 141.11 (C_{60}-sp^2, 2C), 140.58 (C_{70}-sp^2, 1C), 139.63 (C_{60}-sp^2, 2C), 138.77 (C_{60}-sp^2, 2C), 136.99 (C_{70}-sp^2, 1C), 136.72 (C_{70}-sp^2, 1C), 135.01, 134.89, 133.69 (C_{70}-sp^2, 2C), 132.97 (C_{70}-sp^2, 1C), 132.95 (C_{70}-sp^2, 1C), 132.13, 132.09, 131.08 (C_{70}-sp^2, 3C), 131.00 (C_{70}-sp^2, 1C), 130.96 (C_{70}-sp^2, 2C), 129.31, 129.20, 128.38, 124.05, 123.95, 89.93, 89.88, 71.51 (C_{60}-sp^3, 2C), 68.66, 68.61, 66.91 (C_{70}-sp^3, 1C), 66.35 (C_{70}-sp^3, 1C), 54.23, 54.17, 51.91, 36.88 ppm.

Due to overlap only 52 of the expected 70 C_{70} peaks and only 25 of the expected 32 C_{60} peaks could be found.

HRMS (MALDI): found m/z = 1994.0990; calc. for $C_{154}H_{18}O_8$: 1994.1002; Δm/m = 0.60 ppm.

3. NMR Studies on CPP Shuttling

The Gibbs free energy barrier was calculated from the coalescence temperature T_c, the gas constant R, and the separation Δv of the coalescing resonances at low temperature (in Hz), according to:[S15]

$$\Delta G^{\ddagger} = RT_c\left(22.96 + \ln\left(\frac{T_c}{\Delta\nu}\right)\right) \quad \text{(Eq. S1)}$$

The shuttling rate at room temperature (T = 298 K) was then obtained from the Gibbs free energy barrier using the Boltzmann constant k_B and Planck's constant h:

$$\mathrm{k} = \frac{\mathrm{k_B}T}{h}e^{-\frac{\Delta G^{\ddagger}}{RT}} \quad \text{(Eq. S2)}$$

With $f(T_c, \Delta\nu)$ as defined in Equation S1, corresponding to the calculation of $\Delta G^{\ddagger}$, the uncertainty $\Delta(\Delta G^{\ddagger})$ was estimated by standard error propagation:

$$\Delta(\Delta G^{\ddagger}) = \sqrt{\left(\frac{\delta f}{\delta T_c}\Delta T_c\right)^2 + \left(\frac{\delta f}{\delta(\Delta\nu)}\Delta(\Delta\nu)\right)^2} \quad \text{(Eq. S3)}$$

Resolving the partial derivatives gives the following expression (Eq. S4), where ΔT_c is the uncertainty in the coalescence temperature, and $\Delta(\Delta v)$ is the uncertainty in the frequency separation:

$$\Delta(\Delta G^{\ddagger}) = \sqrt{\left(R\Delta T_c\left(23.96 + \ln\left(\frac{T_c}{\Delta\nu}\right)\right)\right)^2 + \left(-\frac{RT_c}{\Delta\nu}\Delta(\Delta\nu)\right)^2} \quad \text{(Eq. S4)}$$

Assuming a frequency uncertainty $\Delta(\Delta v)$ of 1.0 Hz and temperature uncertainties ΔT_c corresponding to half the VT ^{1}H NMR step size (2.5 K for [2]catenanes **1** and 5.0 K for bis-adducts **3**), we calculated the uncertainties $\Delta(\Delta G^{\ddagger})$ reported in Table S2.

This in turn allowed estimation of the uncertainty Δk in the shuttling rate, using the derivative of Equation S2 with respect to $\Delta G^{\ddagger}$ denoted as $g(\Delta G^{\ddagger})$.

$$\Delta k = \left|\frac{dg}{d(\Delta G^{\ddagger})}\right|\Delta(\Delta G^{\ddagger}) \quad \text{(Eq. S5)}$$

Resolving the derivative in Equation S5 yields the following expression for the uncertainty in the shuttling rate; the corresponding values are reported in Table S2:

$$\Delta k = \left| -\frac{k_B}{hR} e^{-\frac{\Delta G^{\ddagger}}{RT}} \right| \Delta(\Delta G^{\ddagger}) \qquad \text{(Eq. S6)}$$

Table S2: Shuttling rates *k*, Gibbs free energy barriers $\Delta G^{\ddagger}$, and their associated uncertainties, determined from coalescence temperatures T_c and the separation of coalescing resonances Δv at low temperatures for various bis-adduct/[10]CPP mixtures (in ~2:1 stoichiometry) as well as different catenanes in $CDCl_3$or TCE-d_2.

Nr.	Isomer	Type	Solvent	T_c [K]	Δv [Hz]	$\Delta G^{\ddagger}$ [kJ mol^{-1}]	k [s^{-1}]
**1a**	*out,out-trans*-1	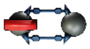	TCE-d_2	> 358[b]	328	> 68.6±0.5	< 5.9±1.2
**3b**	*in,out-trans*-2	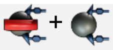	$CDCl_3$	n.a.[a]	n.a.	n.a.	n.a.
**1b**	*in,out-trans*-2	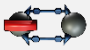	TCE-d_2	353	110	70.8±0.5	2.4±0.5
**3c**	*out,out-trans*-2	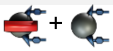	$CDCl_3$	313	77	63.4±1.1	48±20
**3d**	*in,in-trans*-2	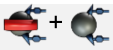	$CDCl_3$	323	95	64.9±1.0	26±11
**3d**	*in,in-trans*-2	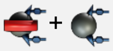	TCE-d_2	338[c]	108	67.7±0.5[c]	8.3±1.8[c]
**1d**	*in,in-trans*-2	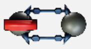	TCE-d_2	358	96	72.3±0.5	1.3±0.3
**3e**	*in,out-trans*-3	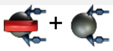	$CDCl_3$	313	89	63.0±1.0	56±24
**3g**	*in,in-trans*-3	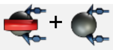	$CDCl_3$	316	90	63.6±1.0	44±19
**1g**	*in,in-trans*-3	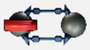	TCE-d_2	348	154[d]	68.8±0.5	5.4±1.1

[a] Could not be determined due to peaks crossing each other during coalescence. [b] Due to the low concentration of the sample, the exact coalescence temperature could not be determined. [c] VT ^{1}H NMR measured with 5 K step size around coalescence temperature. [d] Measured at 293 K due to diasteromeric signal splitting at lower temperatures.

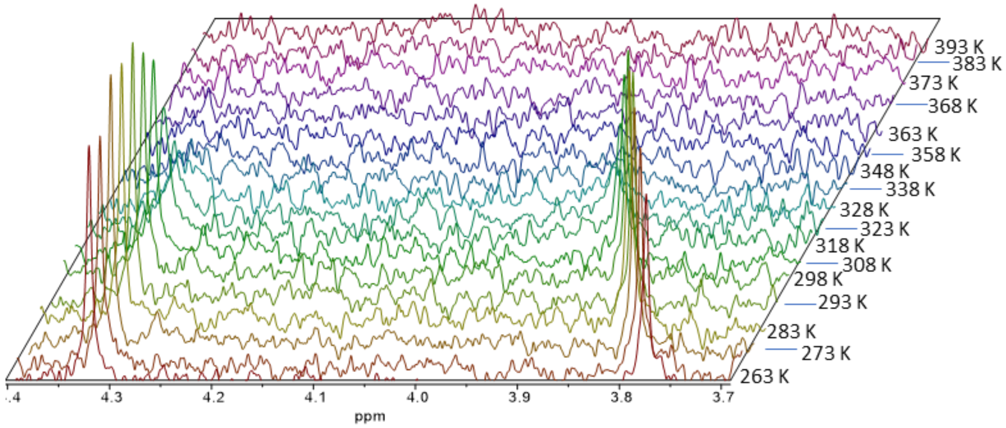

Figure S32: VT ^{1}H NMR (600 MHz, TCE-d_2) spectra stack plot of (*out*,*out*-*trans*-1)$_2$ [2]catenane **1a**.

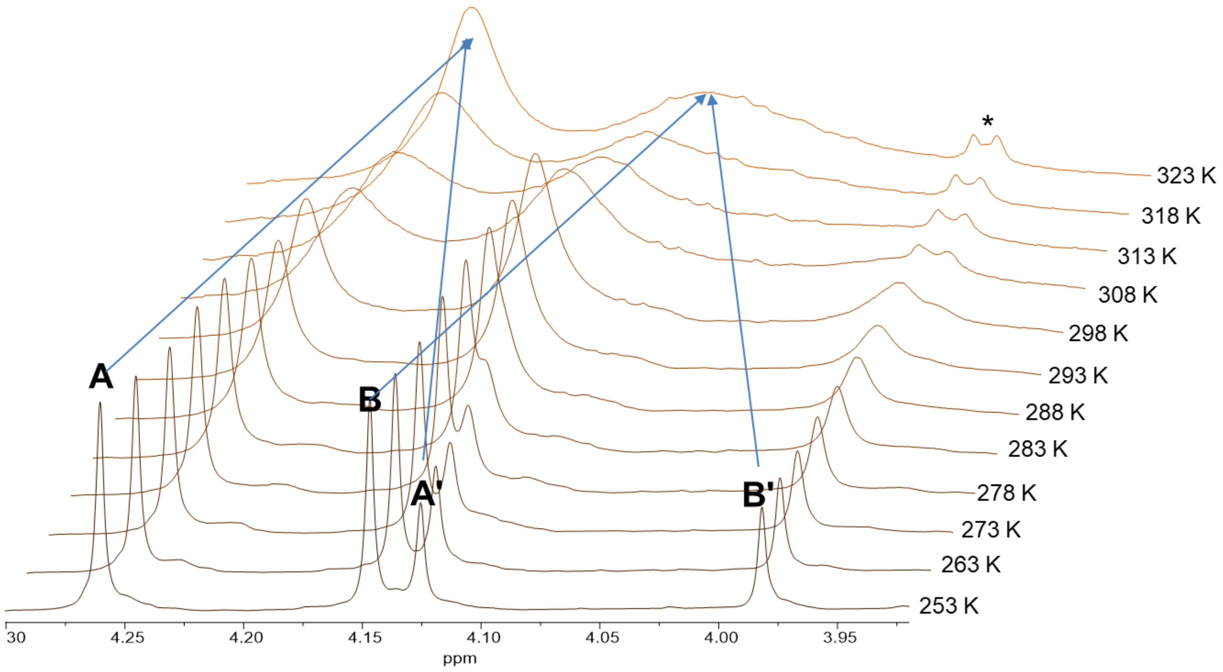

Figure S33: VT ^{1}H NMR (600 MHz, $CDCl_3$) spectra stack plot of a 2:1 mixture of *in*,*out*-*trans*-2 bis-adduct **3b** and [10]CPP. The two different methyl ester resonances A/B with and A'/B' without [10]CPP complexation coalesce to form to new averaged peaks at higher temperature as indicated by the blue arrows.

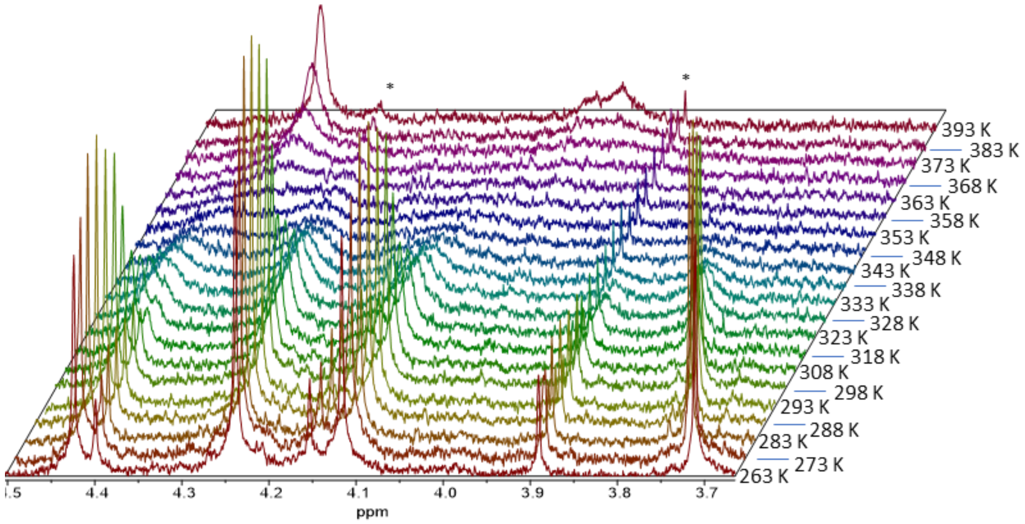

Figure S34: VT ^{1}H NMR (600 MHz, TCE-d_2) spectra stack plot of (*in*,*out*-*trans*-2)$_2$ [2]catenane **1b**. *Residual impurity after HPLC purification.

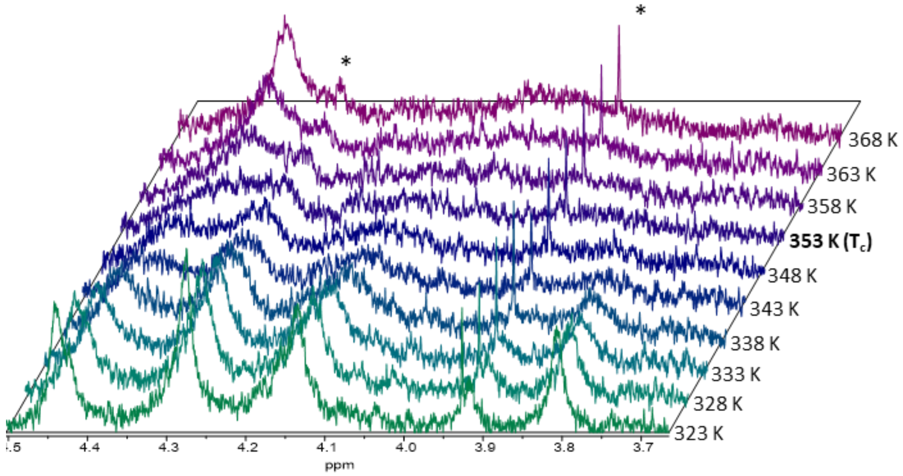

Figure S35: VT ^{1}H NMR (600 MHz, TCE-d_2) spectra stack plot of the coalescence temperature regime of (*in*,*out*-*trans*-2)$_2$ [2]catenane **1b**. *Residual impurity after HPLC purification.

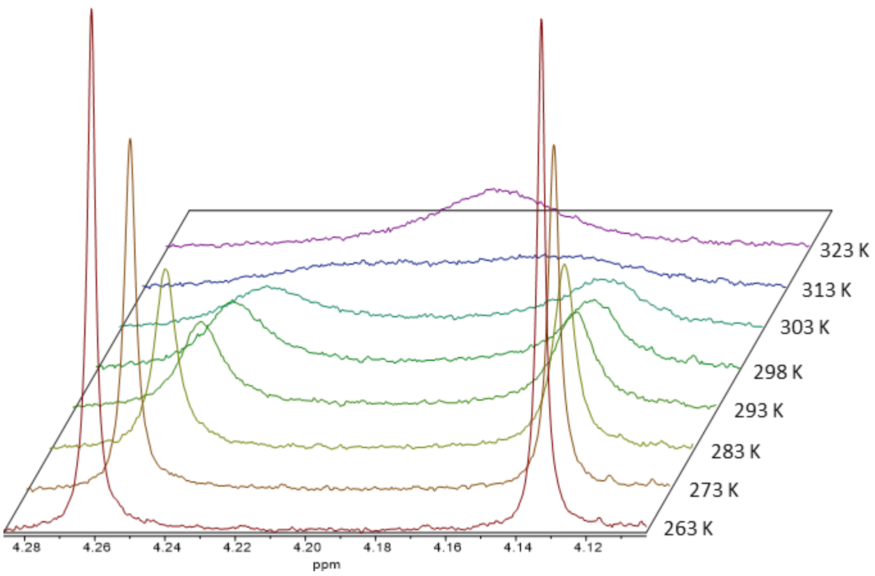

Figure S36: VT ^{1}H NMR (600 MHz, $CDCl_3$) spectra stack plot of a 2:1 mixture of *out*,*out*-*trans*-2 bis-adduct **3c** with [10]CPP.

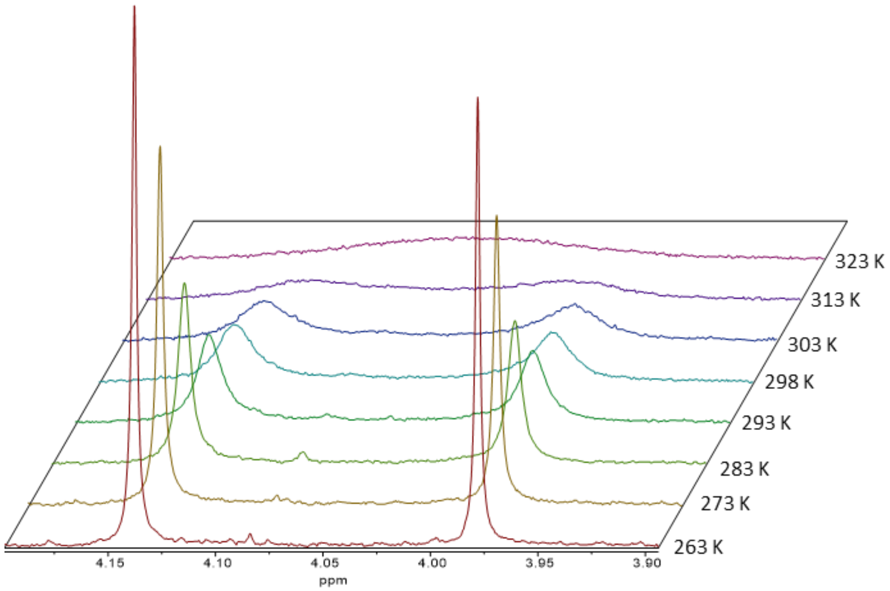

Figure S37: VT ^{1}H NMR (600 MHz, $CDCl_3$) spectra stack plot of a 2:1 mixture of *in*,*in*-*trans*-2 bis-adduct **3d** with [10]CPP.

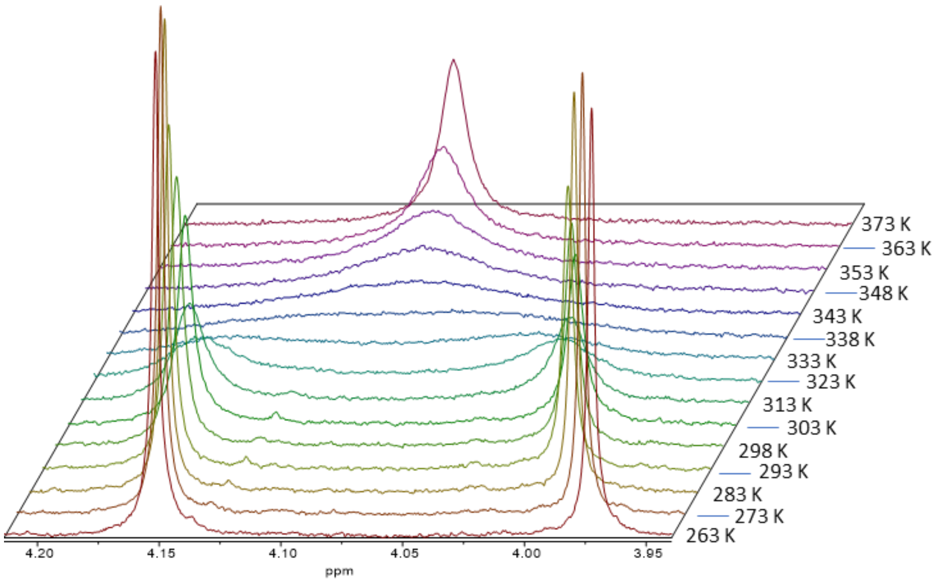

Figure S38: VT ^{1}H NMR (600 MHz, TCE-d_2) spectra stack plot of a 2:1 mixture of *in*,*in*-*trans*-2 bis-adduct **3d** with [10]CPP.

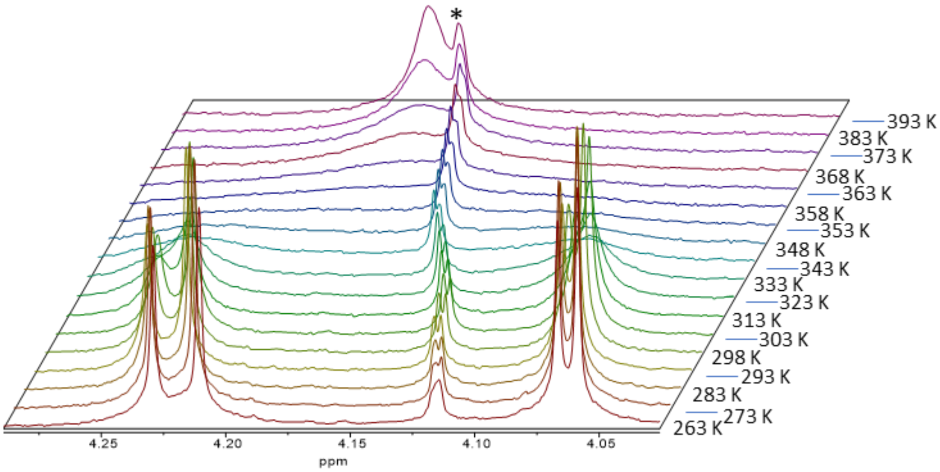

Figure S39: VT ^{1}H NMR (600 MHz, TCE-d_2) spectra stack plot of (*in*,*in*-*trans*-2)$_2$ [2]catenane **1d**. *Residual impurity after HPLC purification.

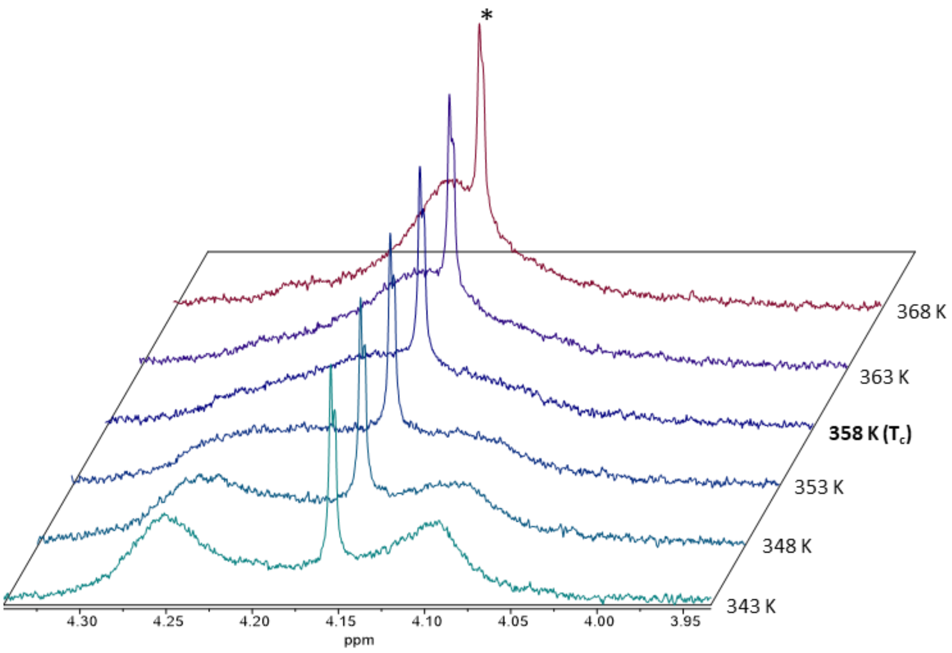

Figure S40: VT ^{1}H NMR (600 MHz, TCE-d_2) spectra stack plot of the coalescence temperature regime of (*in*,*in*-*trans*-2)$_2$ [2]catenane **1d**. *Residual impurity after HPLC purification.

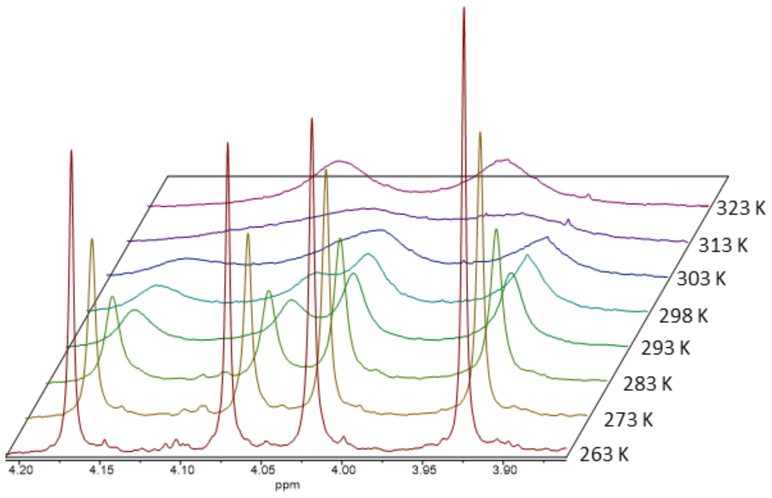

Figure S41: VT ^{1}H NMR (600 MHz, $CDCl_3$) spectra stack plot of a 2:1 mixture of *in*,*out*-*trans*-3 bis-adduct **3e** and [10]CPP.

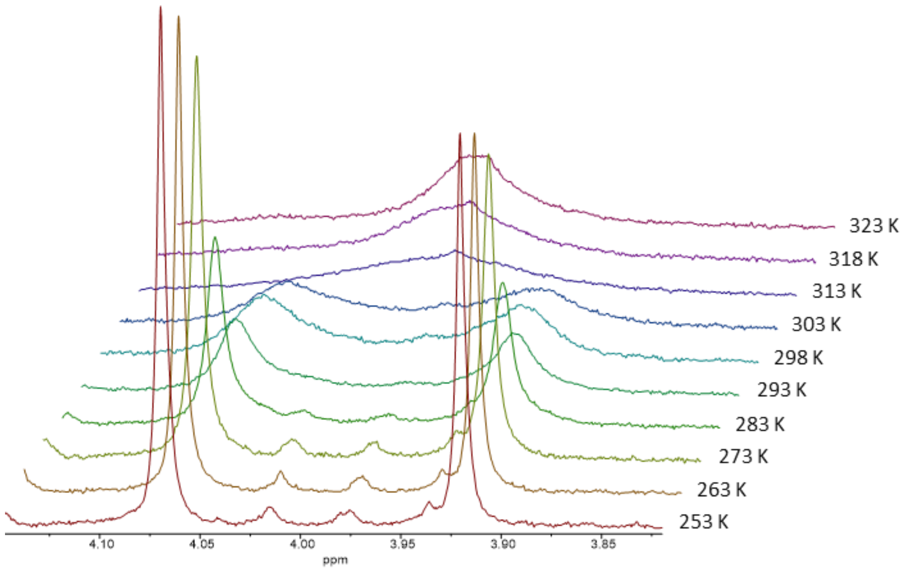

Figure S42: VT ^1H NMR (600 MHz, $CDCl_3$) spectra stack plot of a 2:1 mixture of *in,in-trans*-3 bis-adduct **3g** and [10]CPP.

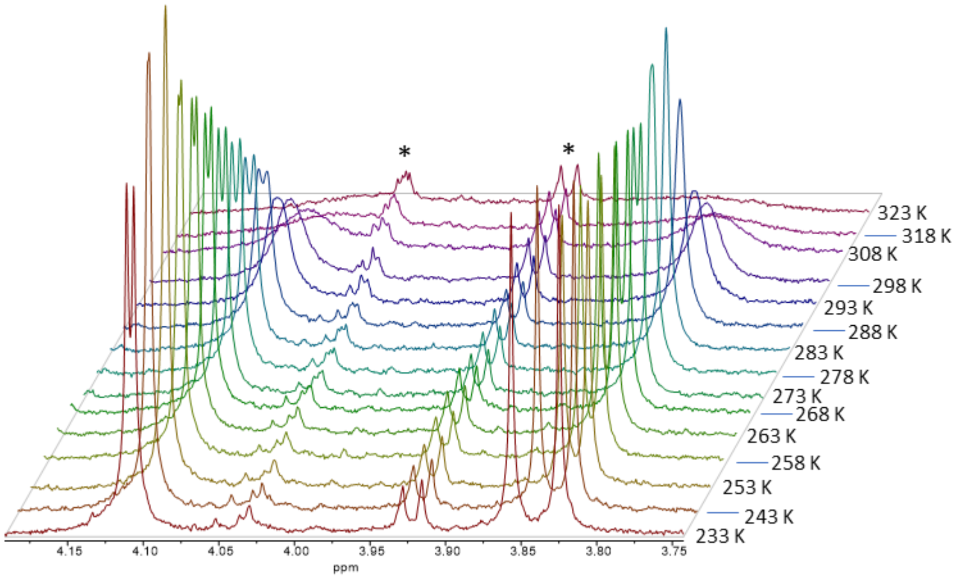

Figure S43: VT ^1H NMR (600 MHz, $CDCl_3$) spectra stack plot of (*in*,*in-trans*-3)$_2$ [2]catenane **1g**.
*Residual impurity after HPLC purification.

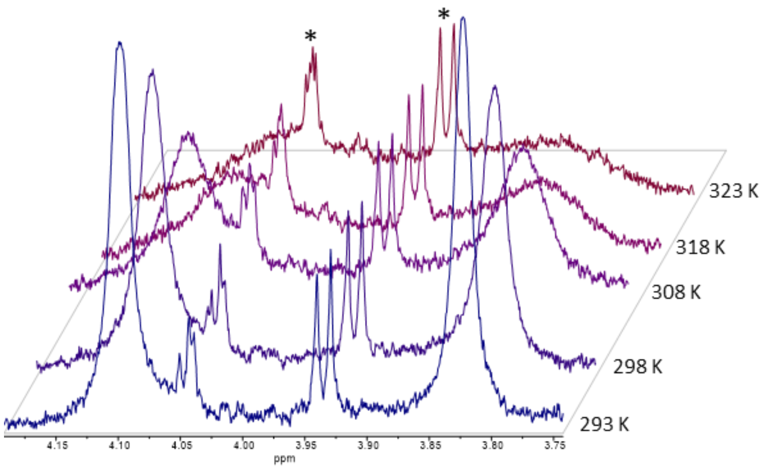

Figure S44: VT ^{1}H NMR (600 MHz, $CDCl_3$) spectra stack plot approaching the coalescence temperature regime of (*in*,*in*-*trans*-3)$_2$ [2]catenane **1g** (up to the solvent temperature limit). *Residual impurity after HPLC purification.

4. Fluorescence Quenching Titrations

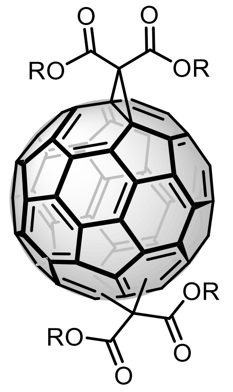

5: R = Me
6: R = Et

Bis-adducts **5** and **6** were synthesized according to published procedures[S16,S17] and purified by preparative TLC (silica, toluene + 3-5% hexane depending on the isomer):

Dimethyl malonate C_{60} mono-adduct **5**:

HRMS (MALDI): found m/z = 980.04981; calc. for $C_{70}H_{12}O_8$: 980.05322; Δm/m = 3.5 ppm.

trans-2 **5a**:

¹H NMR (600 MHz, $CDCl_3$, 298 K): δ = 4.23 (s, 6H, -OCH_3), 4.07 (s, 6H, -OCH_3) ppm.

Analytical data conforms to literature.[S17]

Yield: 8.2 mg, 8.4 µmol, 1%

trans-3 **5b**:

¹H NMR (600 MHz, $CDCl_3$, 298 K): δ = 4.12 (s, 6H, -OCH_3), 4.01 (s, 6H, -OCH_3) ppm.

Analytical data conforms to literature.[S17]

Yield: 27 mg, 27 µmol, 4%

trans-4 **5c**:

¹H NMR (600 MHz, $CDCl_3$, 298 K): δ = 4.08 (s, 3H, -OCH_3), 4.01 (s, 3H, -OCH_3) ppm.

^{13}C NMR (151 MHz, $CDCl_3$, 298 K): δ = 164.3, 164.3, 148.4 (C_{60}-sp^2), 147.2 (C_{60}-sp^2, 2C), 146.8 (C_{60}-sp^2), 146.3 (C_{60}-sp^2, 4C), 145.8 (C_{60}-sp^2, 2C), 145.6 (C_{60}-sp^2, 2C), 145.5 (C_{60}-sp^2, 2C), 145.5 (C_{60}-sp^2), 145.3 (C_{60}-sp^2, 2C), 145.2 (C_{60}-sp^2, 2C), 145.0 (C_{60}-sp^2, 2C), 144.9 (C_{60}-sp^2, 2C), 144.4 (C_{60}-sp^2, 2C), 144.2 (C_{60}-sp^2, 2C), 143.3 (C_{60}-sp^2, 2C), 143.2 (C_{60}-sp^2, 2C), 143.1 (C_{60}-sp^2, 2C), 143.0 (C_{60}-sp^2, 2C), 142.5 (C_{60}-sp^2, 2C), 142.3 (C_{60}-sp^2, 2C), 142.2 (C_{60}-sp^2, 2C), 142.2 (C_{60}-sp^2, 2C), 141.8 (C_{60}-sp^2, 2C), 141.6 (C_{60}-sp^2), 141.2 (C_{60}-sp^2, 2C), 140.7 (C_{60}-sp^2, 2C), 139.3 (C_{60}-sp^2, 2C), 138.7 (C_{60}-sp^2, 2C), 135.8 (C_{60}-sp^2, 2C), 71.2 (C_{60}-sp^3, 2C), 70.9 (C_{60}-sp^3, 2C), 54.2, 54.1, 50.1 ppm.

Due to overlap only 31 of 32 expected resonances were found.

Yield: 2.1 mg, 2.1 µmol, 0.3%

equatorial **5d**:

^{1}H NMR (600 MHz, $CDCl_3$, 298 K): δ = 4.03 (s, 3H, -OCH_3), 4.01 (s, 6H, -OCH_3), 3.98 (s, 3H, -OCH_3) ppm.

Analytical data conforms to literature.[S17]

Yield: 45 mg, 46 µmol, 7%

Diethyl malonate C_{60} mono-adduct **6**:

LRMS (MALDI-TOF): found m/z = 1036.1192; calc. for $C_{74}H_{20}O_8$: 1036.1160; Δm/m = 3.1 ppm.

trans-2 **6a**:

^{1}H NMR (600 MHz, $CDCl_3$, 298 K): δ = 4.72 – 4.69 (m, 2H, -OCH_2-), 4.53 (q, J = 7.1 Hz, 2H, -OCH_2-), 1.60 (t, ^3J = 7.1 Hz, 3H, -CH_3), 1.47 (t, ^3J = 7.1 Hz, 3H, -CH_3) ppm.

^{13}C NMR (151 MHz, $CDCl_3$, 298 K): δ = 164.1, 163.7, 148.2 (C_{60}-sp^2, 2C), 147.2 (C_{60}-sp^2, 2C), 146.5 (C_{60}-sp^2, 2C), 146.4 (C_{60}-sp^2, 2C), 146.0 (C_{60}-sp^2, 2C), 145.8 (C_{60}-sp^2, 2C), 145.7 (C_{60}-

sp^2, 2C), 145.3 (C_{60}-sp^2, 2C), 145.3 (C_{60}-sp^2, 2C), 144.7 (C_{60}-sp^2, 2C), 144.4 (C_{60}-sp^2, 2C), 144.3 (C_{60}-sp^2, 4C), 144.2 (C_{60}-sp^2, 2C), 143.9 (C_{60}-sp^2, 2C), 143.4 (C_{60}-sp^2, 2C), 143.3 (C_{60}-sp^2, 2C), 143.2 (C_{60}-sp^2, 2C), 142.7 (C_{60}-sp^2, 2C), 142.5 (C_{60}-sp^2, 2C), 142.5 (C_{60}-sp^2, 2C), 142.3 (C_{60}-sp^2, 2C), 142.2 (C_{60}-sp^2, 2C), 141.8 (C_{60}-sp^2, 2C), 140.9 (C_{60}-sp^2, 2C), 140.3 (C_{60}-sp^2, 2C), 137.8 (C_{60}-sp^2, 2C), 137.7 (C_{60}-sp^2, 2C), 71.8 (C_{60}-sp^3, 2C), 71.2 (C_{60}-sp^3, 2C), 63.7, 63.5, 50.1, 14.5, 14.4 ppm.

Due to overlap only 29 of 30 expected fullerene resonances were found.

Yield: 33 mg, 32 µmol, 5%

trans-3 **6b**:

^{1}H NMR (600 MHz, $CDCl_3$, 298 K): δ = 4.62 – 4.55 (m, 2H, -OCH_2-), 4.51 – 4.44(m, 2H, -OCH_2-), 1.51 (t, ^3J = 7.15 Hz, -CH_3), 1.42 (t, ^3J = 7.11 Hz, -CH_3) ppm.

^{13}C NMR (151 MHz, $CDCl_3$, 298 K): δ = 163.7, 163.7, 147.4 (C_{60}-sp^2, 2C), 147.1 (C_{60}-sp^2, 2C), 146.7 (C_{60}-sp^2, 2C), 146.7 (C_{60}-sp^2, 2C), 146.7 (C_{60}-sp^2, 2C), 146.6 (C_{60}-sp^2, 2C), 146.6 (C_{60}-sp^2, 2C), 146.3 (C_{60}-sp^2, 2C), 146.2 (C_{60}-sp^2, 2C), 145.8 (C_{60}-sp^2, 2C), 145.7 (C_{60}-sp^2, 2C), 145.5 (C_{60}-sp^2, 2C), 144.8 (C_{60}-sp^2, 2C), 144.6 (C_{60}-sp^2, 2C), 144.3 (C_{60}-sp^2, 2C), 144.0 (C_{60}-sp^2, 2C), 143.8 (C_{60}-sp^2, 2C), 143.7 (C_{60}-sp^2, 2C), 143.6 (C_{60}-sp^2, 2C), 143.2 (C_{60}-sp^2, 2C), 142.7 (C_{60}-sp^2, 2C), 142.4 (C_{60}-sp^2, 2C), 142.3 (C_{60}-sp^2, 2C), 142.1 (C_{60}-sp^2, 2C), 141.8 (C_{60}-sp^2, 2C), 140.5 (C_{60}-sp^2, 2C), 139.3 (C_{60}-sp^2, 2C), 138.7 (C_{60}-sp^2, 2C), 71.9 (C_{60}-sp^3, 2C), 71.5 (C_{60}-sp^3, 2C), 63.5, 63.4, 53.6, 51.8, 14.4, 14.3 ppm.

All 30 of 30 expected fullerene resonances were found.

Yield: 77 mg, 74 µmol, 11%

equatorial **6d**:

^{1}H NMR (600 MHz, $CDCl_3$, 298 K): δ = 4.52 – 4.43 (m, 4H, -OCH_2-), 1.45 – 1.39 (m, 6H, -CH_3) ppm.

^{13}**C NMR** (151 MHz, $CDCl_3$, 298 K): δ = 163.7, 163.7, 163.6, 147.8 (C_{60}-sp^2, 2C), 147.4 (C_{60}-sp^2, 2C), 146.7 (C_{60}-sp^2), 146.6 (C_{60}-sp^2, 2C), 146.4 (C_{60}-sp^2, 2C), 146.2 (C_{60}-sp^2, 2C), 145.7 (C_{60}-sp^2, 2C), 145.6 (C_{60}-sp^2, 2C), 145.3 (C_{60}-sp^2, 2C), 145.2 (C_{60}-sp^2), 144.9 (C_{60}-sp^2, 2C), 144.9 (C_{60}-sp^2, 2C), 144.8 (C_{60}-sp^2, 2C), 144.6 (C_{60}-sp^2, 2C), 144.6 (C_{60}-sp^2, 2C), 144.3 (C_{60}-sp^2, 2C), 144.2 (C_{60}-sp^2, 2C), 144.0 (C_{60}-sp^2, 2C), 143.9 (C_{60}-sp^2, 2C), 143.6 (C_{60}-sp^2, 2C), 143.5 (C_{60}-sp^2, 2C), 143.4 (C_{60}-sp^2, 2C), 143.1 (C_{60}-sp^2, 2C), 142.5 (C_{60}-sp^2, 2C), 142.0 (C_{60}-sp^2, 2C), 141.8 (C_{60}-sp^2, 2C), 141.7 (C_{60}-sp^2, 2C), 139.0 (C_{60}-sp^2, 2C), 138.9 (C_{60}-sp^2, 2C), 71.8 (C_{60}-sp^3), 71.8 (C_{60}-sp^3, 2C), 70.5 (C_{60}-sp^3), 63.4, 63.4, 63.4, 53.7, 51.5, 14.3, 14.3.

All 32 of 32 expected fullerene resonances were found. Due to overlap only two out of three -$\mathit{C}H_3$ resonances were found.

Yield: 69 mg, 67 µmol, 10%

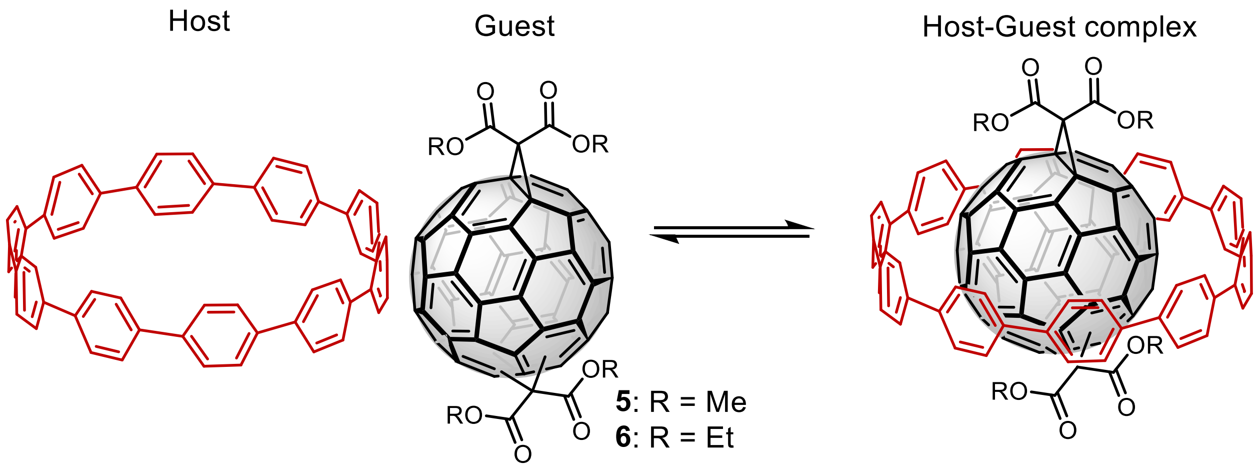

Figure S45: Reaction scheme showing the Host-Guest complexation equilibrium studied by the fluorescence quenching titrations of [10]CPP (host) with regioisomers of bis-adducts **5** and **6** (guests).

Table S3: Average association constants K_a for [10]CPP binding to fullerene bis-adducts **5** and **6** as determined by fluorescence quenching titrations (1:1 model, Nelder-Mead fit, supramolecular.org).[S18] Errors represent the standard deviation from triplicate measurements.

Regioisomer	Bis-adduct **5** [L mol^{-1}]	Bis-adduct **6** [L mol^{-1}]
trans-2	$9.7 \pm 0.1 \times 10^5$	$9.2 \pm 0.8 \times 10^5$
trans-3	$6.09 \pm 0.02 \times 10^5$	$5.3 \pm 0.2 \times 10^5$
trans-4	$9.6 \pm 0.6 \times 10^4$	n.a.[a]
equatorial	$1.4 \pm 0.3 \times 10^5$	$1.3 \pm 0.1 \times 10^5$

[a] *Trans*-4 **6c** was not obtained in high enough purity and is therefore absent in the data.

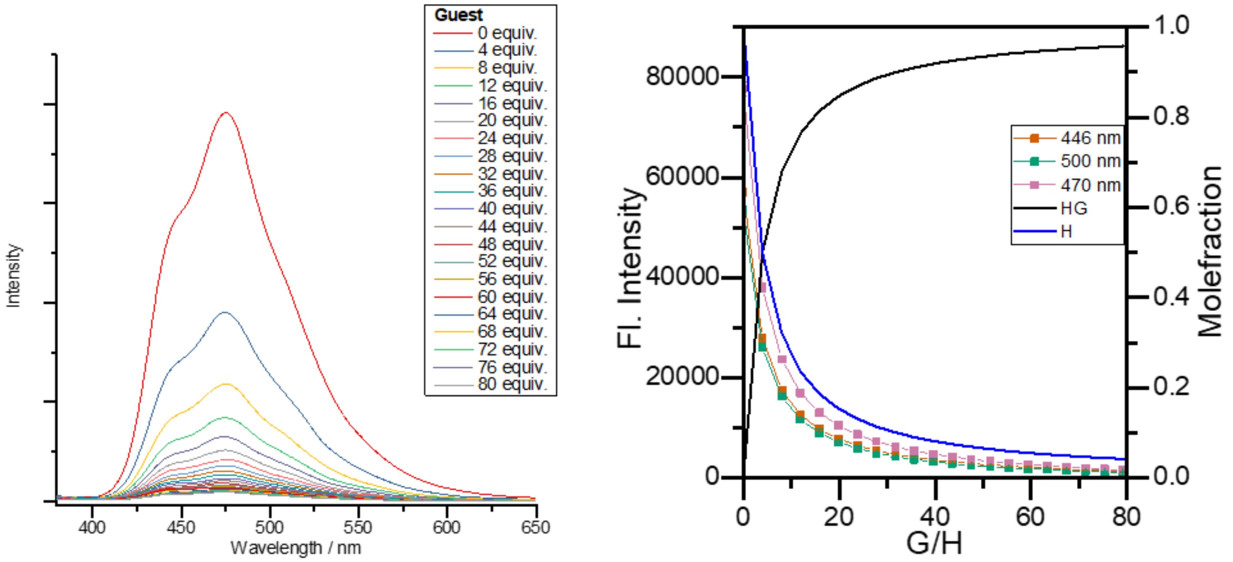

Figure S46: Representative stacked fluorescence spectra showing the titration of 0–80 equivalents of *trans*-2 diethylmalonate C_{60} bis-adduct **5a** into a 0.3 µM solution of [10]CPP in

toluene. Corresponding intensity plots at 446, 470, and 500 nm are shown, along with mole fraction plots for the free host (H) and the host–guest complex (HG).

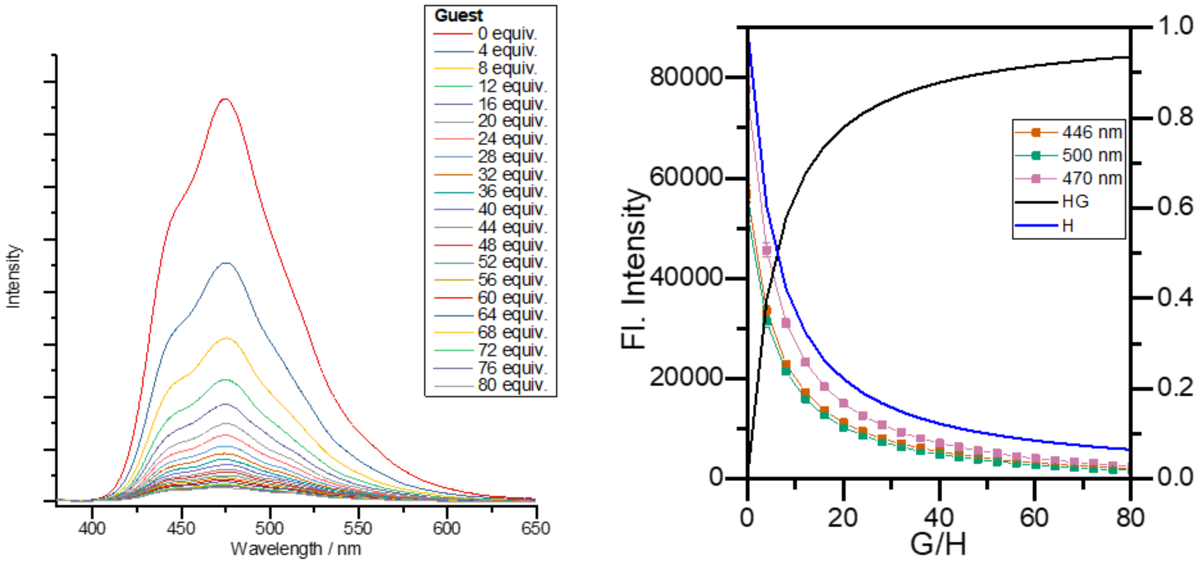

Figure S47: Representative stacked fluorescence spectra showing the titration of 0–80 equivalents of *trans*-3 diethylmalonate C_{60} bis-adduct **5b** into a 0.3 μM solution of [10]CPP in toluene. Corresponding intensity plots at 446, 470, and 500 nm are shown, along with mole fraction plots for the free host (H) and the host–guest complex (HG).

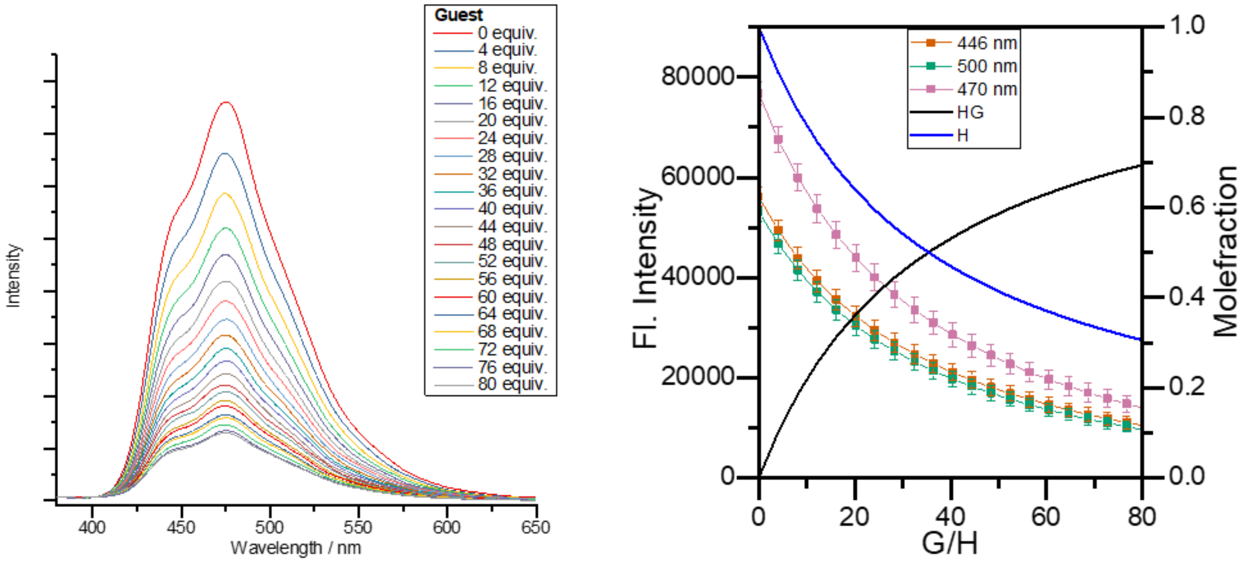

Figure S48: Representative stacked fluorescence spectra showing the titration of 0–80 equivalents of *trans*-4 diethylmalonate C_{60} bis-adduct **5c** into a 0.3 μM solution of [10]CPP in

toluene. Corresponding intensity plots at 446, 470, and 500 nm are shown, along with mole fraction plots for the free host (H) and the host–guest complex (HG).

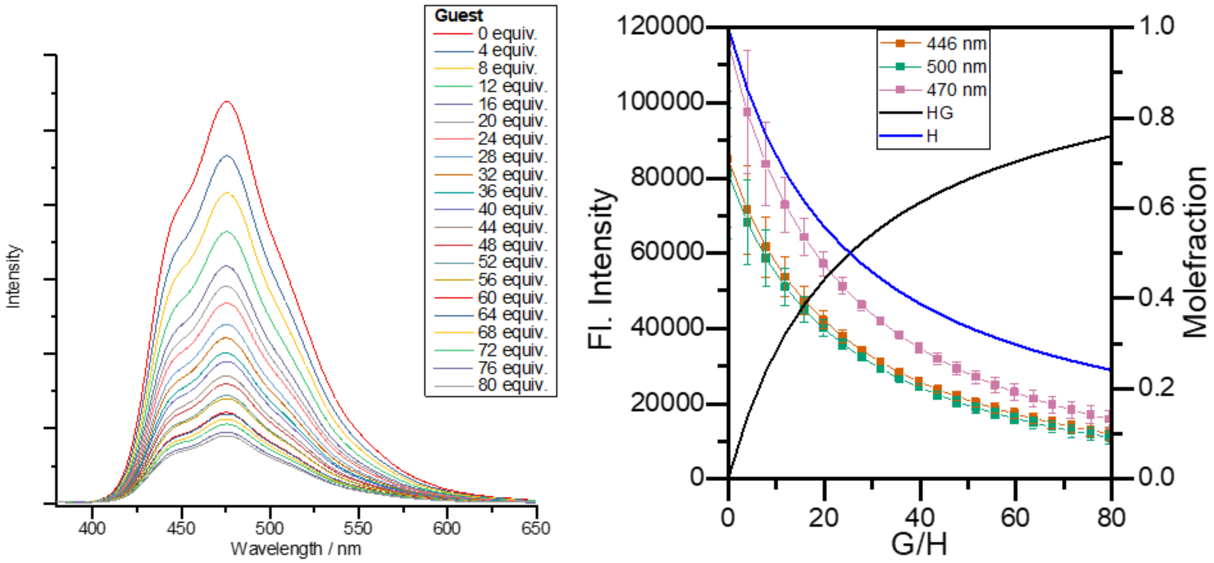

Figure S49: Representative stacked fluorescence spectra showing the titration of 0–80 equivalents of *equatorial* diethylmalonate C_{60} bis-adduct **5d** into a 0.3 μM solution of [10]CPP in toluene. Corresponding intensity plots at 446, 470, and 500 nm are shown, along with mole fraction plots for the free host (H) and the host–guest complex (HG).

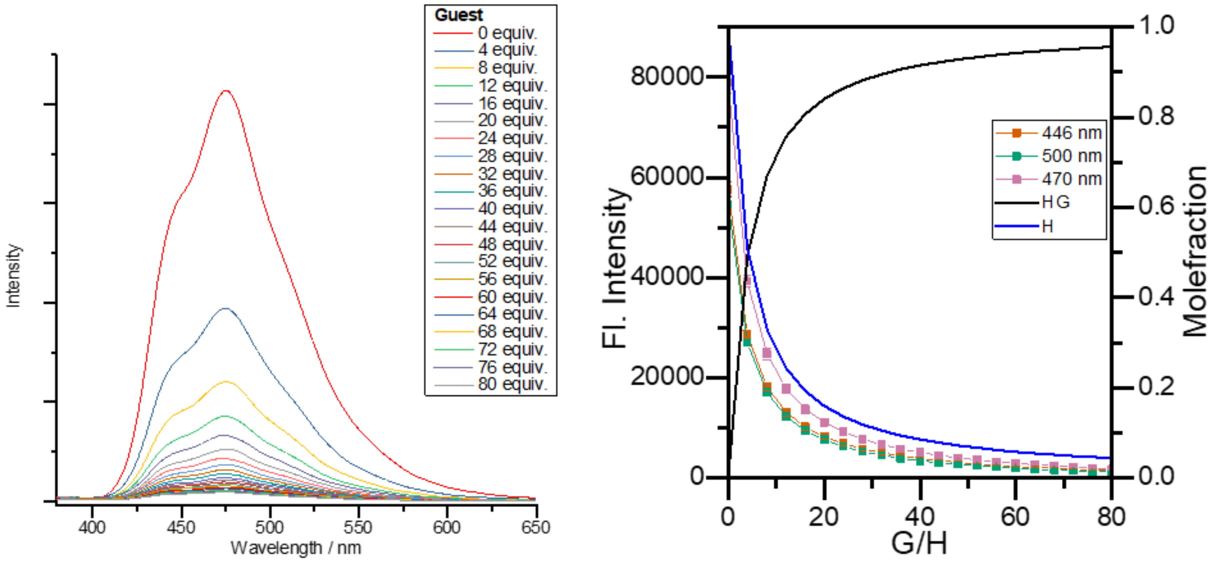

Figure S50: Representative stacked fluorescence spectra showing the titration of 0–80 equivalents of *trans*-2 diethylmalonate C_{60} bis-adduct **6a** into a 0.3 μM solution of [10]CPP in

toluene. Corresponding intensity plots at 446, 470, and 500 nm are shown, along with mole fraction plots for the free host (H) and the host–guest complex (HG).

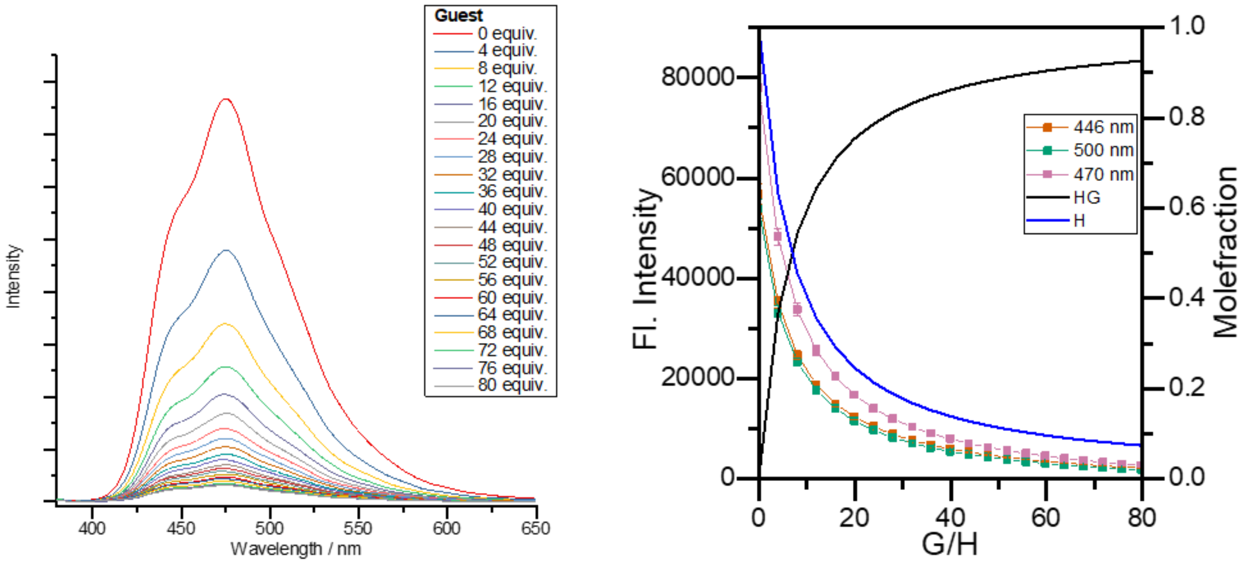

Figure S51: Representative stacked fluorescence spectra showing the titration of 0–80 equivalents of *trans*-3 diethylmalonate C_{60} bis-adduct **6b** into a 0.3 μM solution of [10]CPP in toluene. Corresponding intensity plots at 446, 470, and 500 nm are shown, along with mole fraction plots for the free host (H) and the host–guest complex (HG).

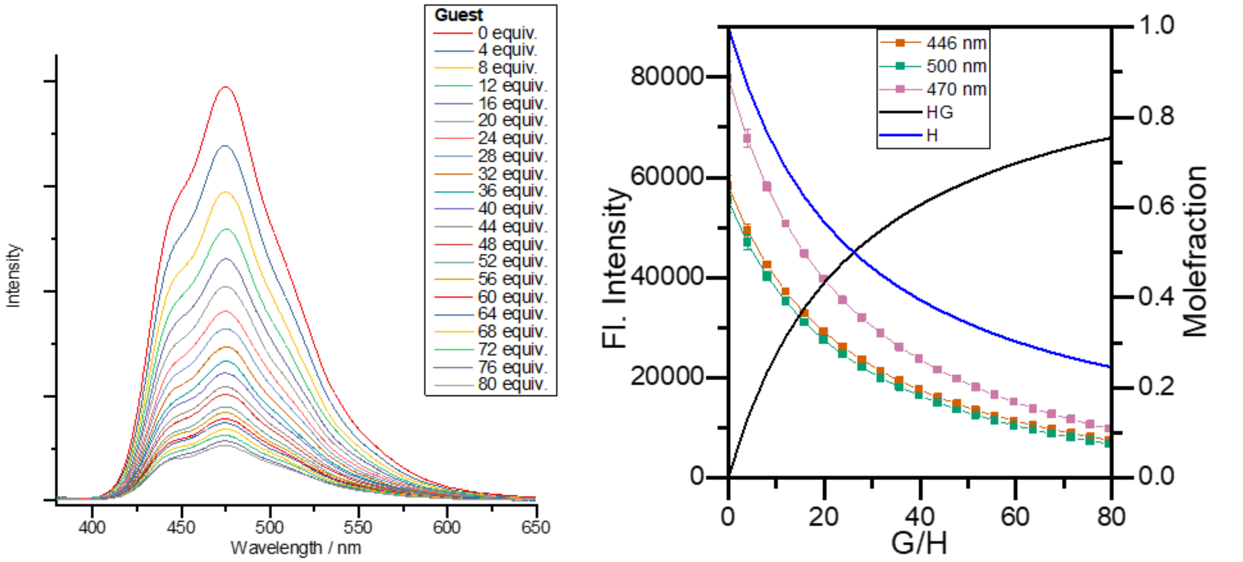

Figure S52: Representative stacked fluorescence spectra showing the titration of 0–80 equivalents of *equatorial* diethylmalonate C_{60} bis-adduct **6d** into a 0.3 μM solution of

[10]CPP in toluene. Corresponding intensity plots at 446, 470, and 500 nm are shown, along with mole fraction plots for the free host (H) and the host–guest complex (HG).

Table S4: Data links for the host-guest titrations on supramolecular.org.[S18]

Host	Links
**5a**	http://app.supramolecular.org/bindfit/view/9ea14391-9ed6-4fd2-af56-d57213d93e63
	http://app.supramolecular.org/bindfit/view/6adc4645-3078-44b6-a2a8-146f5240af4a
	http://app.supramolecular.org/bindfit/view/54a72ba1-453c-470d-8155-bea8c6669fea
**5b**	http://app.supramolecular.org/bindfit/view/80651aa6-68cc-4478-a3bb-7409aef832a3
	http://app.supramolecular.org/bindfit/view/faa8e43d-50b3-46cb-8414-00747d5953e3
	http://app.supramolecular.org/bindfit/view/148aac5a-7ee7-4a94-82f4-d6fa3cb1e225
**5c**	http://app.supramolecular.org/bindfit/view/c0b01219-0172-439a-8360-101376d54fd3
	http://app.supramolecular.org/bindfit/view/c1fb17be-eb8f-4896-b7a9-6364c0528a56
	http://app.supramolecular.org/bindfit/view/9ca35b11-2dd1-414b-a66d-e96451b4eb58
**5d**	http://app.supramolecular.org/bindfit/view/4f7a418c-f279-45ba-95e3-7cacf9393017
	http://app.supramolecular.org/bindfit/view/6531aac1-e6a6-47de-a4b7-ba6f424f13e7
	http://app.supramolecular.org/bindfit/view/c291775e-29d2-4c16-9281-1fc8cfcce868
**6a**	http://app.supramolecular.org/bindfit/view/e67ec0f2-fc3b-4d30-aaa6-6b03afa87065
	http://app.supramolecular.org/bindfit/view/f256e91c-0b12-402c-bbc6-09d0e9f59c6a
	http://app.supramolecular.org/bindfit/view/1928a568-018d-4508-9329-87220a657a9b
**6b**	http://app.supramolecular.org/bindfit/view/18774508-af4c-4774-a130-2af4fa3c0773
	http://app.supramolecular.org/bindfit/view/7a6ea21f-59a3-4e11-a71c-2aefe0ea841e
	http://app.supramolecular.org/bindfit/view/a63868ef-6347-4cdd-9591-babc7df16e14
**6d**	http://app.supramolecular.org/bindfit/view/dc878a64-28fe-4694-8180-7c07c69841c1
	http://app.supramolecular.org/bindfit/view/4e241693-21d9-43ff-9896-1145c4cb5271
	http://app.supramolecular.org/bindfit/view/3c3f1d3f-ac33-4288-8a79-6375a85a5467

5. Crystallographic Data

Co-crystal of *in*,*in*-*trans*-3 C_{60} bis-adduct **3g** and [10]CPP. was obtained by slow evaporation from $CDCl_3$.

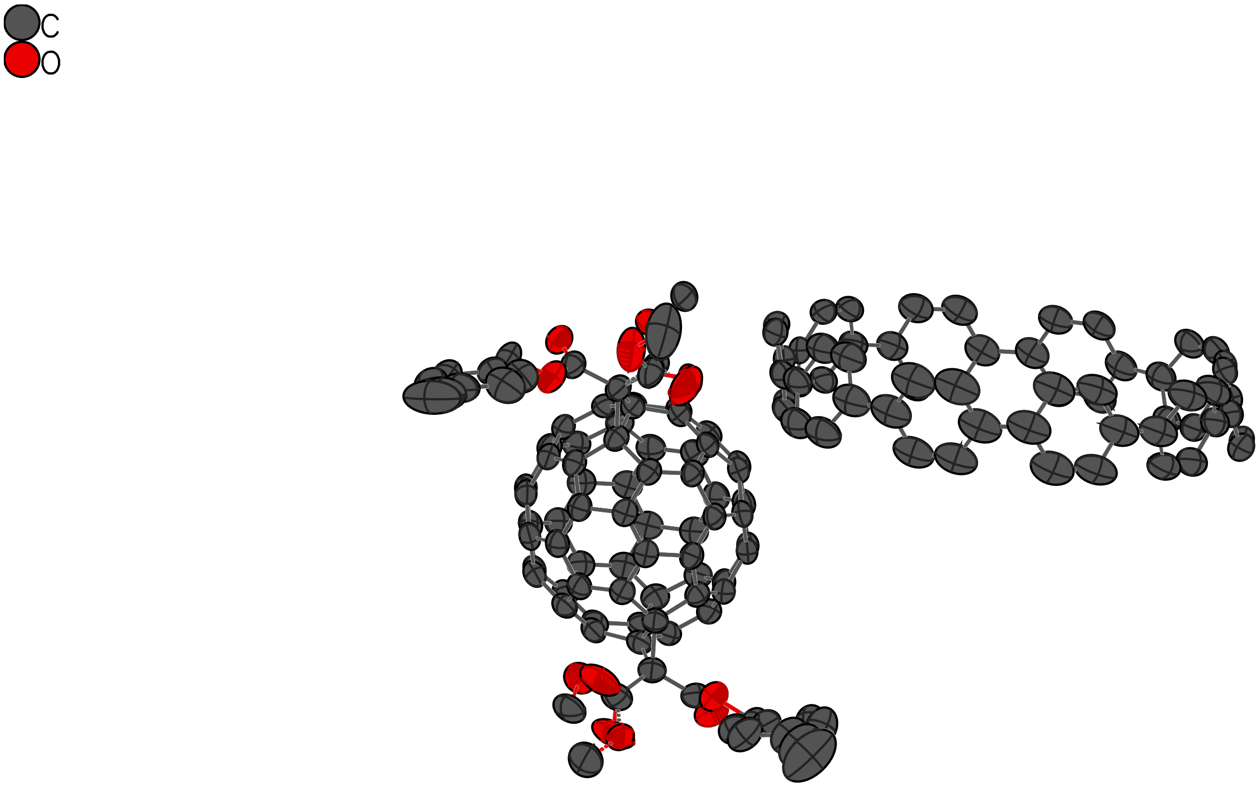

Figure S53: Asymmetric unit for the crystal structure of the [10]CPP co-crystal with the *in*,*in*-*trans*-3 C_{60} bis-adduct **3g** (ellipsoids at 50% probability). Only one enantiomer of the racemic pair is shown. The methyl ester groups are disordered over two positions.

Disorder and refinement details: Two methyl ester groups on the fullerene bis-adduct were modelled as disordered over two positions. The first ester group is disordered between the atom sets [C1, O2, O3, C4] and [C1A, O2A, O3A, C4A], with refined occupancies of 0.433 and 0.567, respectively. The second ester group is disordered between the atom sets [O02F, O02G, O03O, O03P, C6] and [C6A, C02Q], with refined occupancies of 0.468 and 0.532, respectively. Appropriate restraints (SADI, SIMU, ISOR) were applied to maintain chemically reasonable bond lengths, angles, and atomic displacement parameters across both disorder models.

The structure crystallises as a racemic twin in space group *P*-1; only the asymmetric unit is shown.

CheckCIF alerts: A high Rint value (0.2425) is observed, attributed to crystal quality and extensive disorder—particularly in the solvent region. Attempts to model the solvent molecules explicitly resulted in unstable refinement with large positional shifts, suggesting poorly ordered or diffuse solvent content. Nevertheless, the refinement converged with acceptable residuals and chemically reasonable geometry. No A-level alerts were observed in the final structure validation.

Table S5: Crystal data and structure refinement for 2024S032_FSt_B1_230_SM_TMB.

Identification code	2024S032_FSt_B1_230_SM_TMB
CCDC deposition number:	2448367
Empirical formula	$C_{149.83}H_{60.86}Cl_{11.85}O_8$
Formula weight	2408.88
Temperature/K	161.00
Crystal system	triclinic
Space group	P-1
a/Å	15.9594(12)
b/Å	16.2718(12)
c/Å	20.8553(15)
α/°	90.963(5)
β/°	94.519(5)
γ/°	97.050(5)

Volume/Å^3	5356.3(7)
Z	2
ρ_{calc}g/cm^3	1.494
μ/mm^{-1}	3.356
F(000)	2451.0
Crystal size/mm^3	0.052 × 0.032 × 0.031
Radiation	CuKα (λ = 1.54178)
2Θ range for data collection/°	4.252 to 133.58
Index ranges	-18 ≤ h ≤ 19, -19 ≤ k ≤ 19, -24 ≤ l ≤ 24
Reflections collected	164702
Independent reflections	18955 [R_{int} = 0.2425, R_{sigma} = 0.1141]
Data/restraints/parameters	18955/3542/1456
Goodness-of-fit on F^2	0.928
Final R indexes [I>=2σ (I)]	R_1 = 0.0816, wR_2 = 0.2119
Final R indexes [all data]	R_1 = 0.1424, wR_2 = 0.2590
Largest diff. peak/hole / e Å^{-3}	0.79/-0.25

Table S6: Solvent masks information for 2024S032_FSt_B1_230_SM_TMB.

Number	X	Y	Z	Volume	Electron count	Content
1	-0.500	0.000	0.500	1226.6	457.1	?
2	0.226	0.400	0.238	24.0	0.0	?
3	0.774	0.600	0.762	24.0	0.0	?

Crystals of the *out,out-trans*-1 macrocycle **4a** were obtained from slow evaporation of a solution in $CDCl_3$.

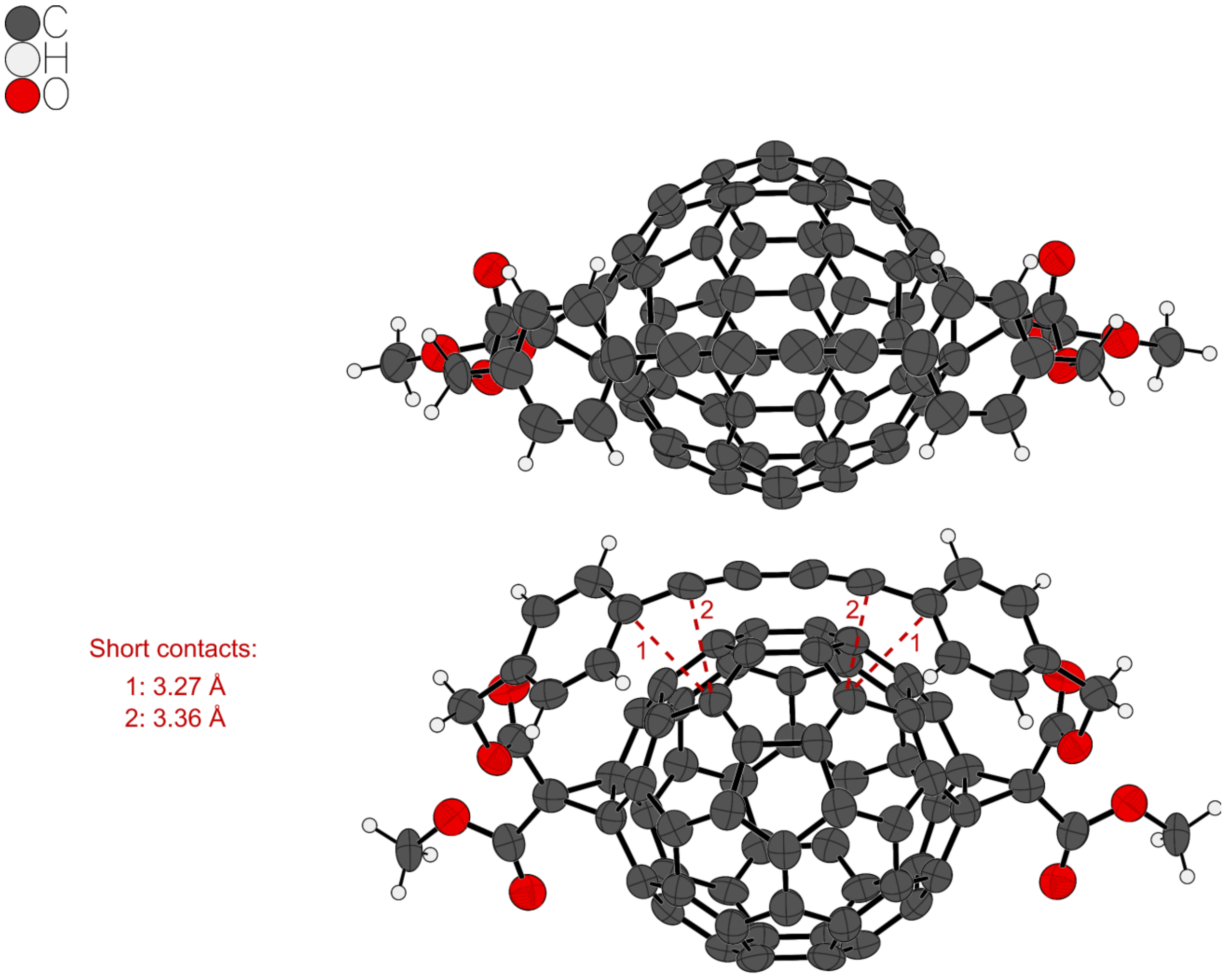

Figure S54: Crystal structure of the *out,out-trans*-1 macrocycle **4a** (ellipsoids at 50% probability). Short contacts between the diphenylbutadiyne linker and the fullerene are indicated in red and the distances are given.

Refinement details: Several weak or unreliable reflections were omitted from the refinement due to poor data quality, large residuals, or instability in the model. As a result, the measured data completeness at full θ is slightly reduced. The final model is chemically reasonable and stable. A solvent mask was applied to treat disordered chloroform solvent, as explicit modeling led to large, erratic shifts and refinement instability.

The structure crystallizes in the $P2_1/m$ space group.

CheckCIF alerts: Several B-level alerts were issued in the checkCIF report due to limitations in data quality. The crystal used for data collection was very small and diffracted weakly, resulting in a relatively high wR2 value (0.42) and modest bond precision (C–C = 0.0123 Å). A small number of weak or unstable high-angle reflections were omitted from the refinement to improve stability, leading to a slightly reduced measured data fraction at full theta (0.942). The disordered chloroform solvent could not be modeled satisfactorily and was therefore treated using a solvent mask. The largest residual peak (+1.27 $eÅ^{-3}$) is located near the masked region and likely arises from diffuse solvent contributions or Fourier truncation artifacts. Despite these limitations, the model refined cleanly with anisotropic displacement parameters for all non-hydrogen atoms, and the molecular geometry is chemically reasonable and consistent with expectations.

Table S7: Crystal data and structure refinement for FST25044_auto.

Identification code	FST25044_auto
CCDC deposition number:	2448364
Empirical formula	$C_{88.36}H_{20.36}Cl_{7.09}O_8$

Formula weight	1461.07
Temperature/K	149.9(2)
Crystal system	monoclinic
Space group	$P2_1/m$
a/Å	13.8349(16)
b/Å	24.4206(16)
c/Å	18.1092(18)
α/°	90
β/°	109.120(11)
γ/°	90
Volume/Å^3	5780.8(10)
Z	4
ρ_{calc}g/cm^3	1.679
μ/mm^{-1}	3.780
F(000)	2940.0
Crystal size/mm^3	0.36 × 0.06 × 0.04
Radiation	Cu Kα (λ = 1.54184)
2Θ range for data collection/°	7.24 to 153.714
Index ranges	$-17 \le h \le 17$, $-30 \le k \le 24$, $-20 \le l \le 22$
Reflections collected	29565
Independent reflections	11454 [R_{int} = 0.1524, R_{sigma} = 0.1693]

Data/restraints/parameters	11454/942/863
Goodness-of-fit on F^2	1.063
Final R indexes [I>=2σ (I)]	R_1 = 0.1296, wR_2 = 0.3363
Final R indexes [all data]	R_1 = 0.1922, wR_2 = 0.4202
Largest diff. peak/hole / e Å^{-3}	1.27/-0.61

Table S8: Solvent masks information for FST25044_auto.

Number	X	Y	Z	Volume	Electron count	Content
1	0.500	-0.911	0.500	1383.4	517.8	9.45 chloroform
2	0.165	0.750	0.517	24.6	0.0	?
3	0.470	0.750	0.097	24.2	0.0	?
4	0.530	0.250	0.903	24.2	0.0	?
5	0.835	0.250	0.483	24.6	0.0	?

Crystals of the *out*,*out*-*trans*-2 macrocycle **4c** were obtained from slow evaporation of a solution in chloroform.

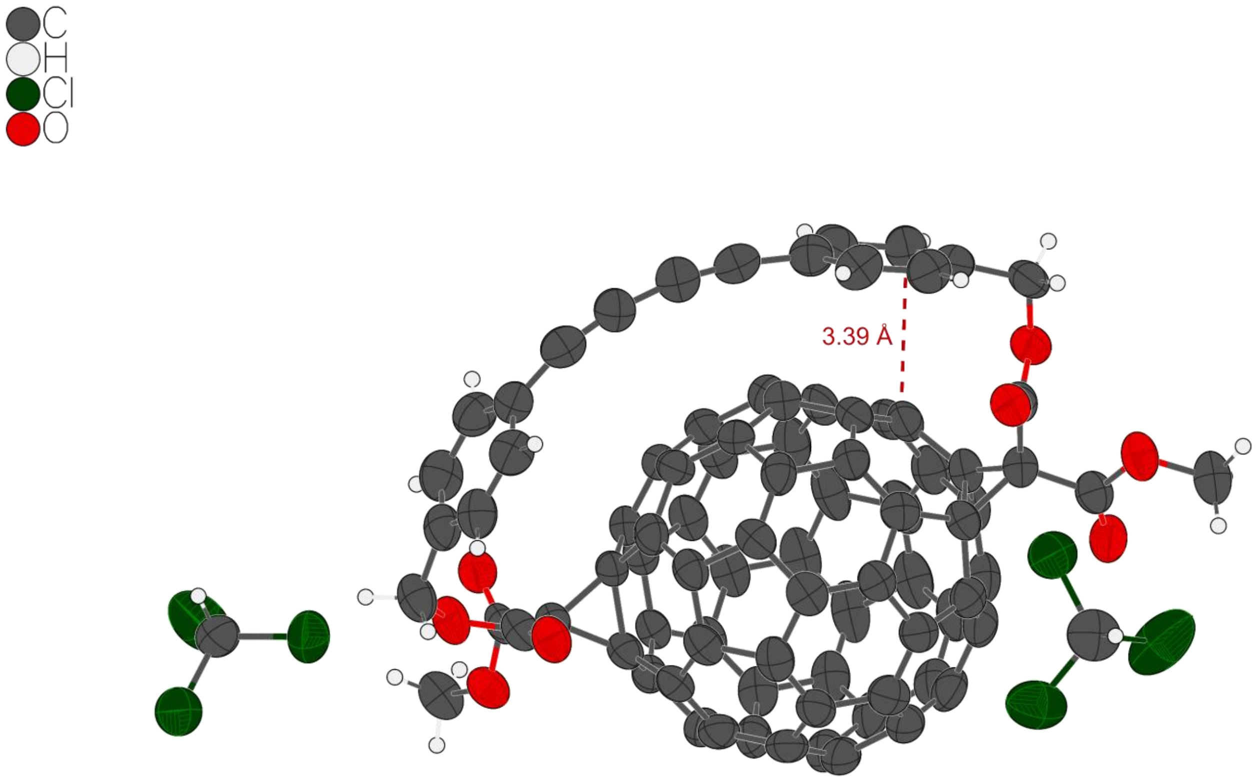

Figure S55: Asymmetric unit for the crystal structure of the *out*,*out*-*trans*-2 macrocycle **4c** with two chloroform molecules (ellipsoids at 50% probability). Only one enantiomer of the racemic pair is shown. A short contact between the diphenylbutadiyne linker and the fullerene is indicated in red and the distance is given.

Refinement details: The structure was refined using data from a small, weakly diffracting crystal. Several weak or high-residual reflections were omitted to improve refinement stability and reduce R-factors. Chloroform solvent molecules were modeled explicitly, and a SWAT instruction was included to account for remaining diffuse solvent contribution. All non-hydrogen atoms were refined anisotropically. The final model is chemically reasonable and converged without significant issues.

CheckCIF alerts: Several A- and B-level alerts were triggered due to limited data quality. The crystal used for measurement was very small and diffracted weakly, resulting in a high Rint value (0.384), a low observed-to-unique reflection ratio (36%), and modest bond precision (C–C = 0.0126 Å). A small number of unstable reflections were omitted to stabilize the refinement, which led to a minor discrepancy between reported and recalculated R1 values

and the number of reflections with I > 2σ(I). The chloroform solvent molecules were successfully modeled explicitly. A SWAT instruction was applied to account for residual diffuse solvent contributions not captured by discrete atom modeling. Despite these limitations, the model is chemically sensible, and the refinement converged with anisotropic displacement parameters for all non-hydrogen atoms.

Table S9: Crystal data and structure refinement for FST25042_auto.

Identification code	FST25042_auto
CCDC deposition number:	2448366
Empirical formula	$C_{88}H_{20}Cl_6O_8$
Formula weight	1417.74
Temperature/K	150.00(14)
Crystal system	monoclinic
Space group	$P2_1/c$
a/Å	12.8371(6)
b/Å	27.752(3)
c/Å	16.345(2)
α/°	90
β/°	104.348(8)
γ/°	90
Volume/Å^3	5641.3(9)
Z	4

ρ_{calc}g/cm^3	1.669
μ/mm^{-1}	3.389
F(000)	2856.0
Crystal size/mm^3	0.2 × 0.07 × 0.06
Radiation	Cu Kα (λ = 1.54184)
2Θ range for data collection/°	7.108 to 153.3
Index ranges	$-15 \le h \le 11$, $-34 \le k \le 34$, $-19 \le l \le 20$
Reflections collected	54962
Independent reflections	11695 [R_{int} = 0.3837, R_{sigma} = 0.3052]
Data/restraints/parameters	11695/0/923
Goodness-of-fit on F^2	0.937
Final R indexes [I>=2σ (I)]	R_1 = 0.1031, wR_2 = 0.2272
Final R indexes [all data]	R_1 = 0.2319, wR_2 = 0.3138
Largest diff. peak/hole / e Å^{-3}	0.03/-0.02

6. Computational Data

Parameters of the [2]catenane **1g** were generated within the antechamber module using the General Amber Force Field (GAFF),[S19] with partial charges set to fit the electrostatic potential generated at the B3LYP/6-31G* level by the RESP model.[S20] The charges were calculated using the Merz-Singh-Kollman with the Gaussian 16 package.[S21]

Molecular dynamic simulations were performed using chloroform as solvent and GROMACS 2022.5[S22] patched with PLUMED v.2.8.[S23] The system was minimized using the steepest-descent algorithm and equilibrated under NPT condition (constant N: number of particles, P: pressure, T: temperature). Production runs were carried out in NPT ensemble for 1 μs. Temperature and pressure were maintained constant at 300 K and 1 bar, respectively, using the v-rescale thermostat with coupling constants of 0.2 ps, and the Berendsen barostat with coupling constants of 4 ps. Electrostatic interactions were treated using the Particle-Mesh-Ewald (PME) method and a cutoff of 1.0 nm. Van der Waals interactions were truncated at 1.0 nm. Bond constraints involving hydrogen atoms were enforced using the LINCS algorithm. The leap-frog integrator was used to propagate the dynamics with a 2 fs time step.

6.1. Kinetics

Infrequent well-tempered metadynamics (WT-MetaD)[S24] were performed starting from different configurations. Starting from the “bound” state, where the [10]CPP is surrounding one C_{60} position, the number of contacts between the nanoring and the fullerene was used as collective variable. The metadynamics bias was added every 80 ps, using Gaussians with an initial height of 1 kJ/mol, a width of 0.5, and a bias factor of 20. The CV value was checked every 500 steps with the COMMITTOR function implemented in PLUMED and the simulation was stopped if CV was below 8.0. To study the movement going from one phenyl ring position to the other from the same chain, a different collective variable was used. The difference between the distance from the center of masses (COM) of the [10]CPP and each of the two C_{60} positions was used. The metadynamics bias was added every 100 ps, using Gaussian with an initial height of 1 kJ/mol, a width of 0.01 nm and a bias factor of 20. The COMMITTOR checked the value of the CV every 500 steps and stopped the simulation if CV was below -

0.5 nm. To study the binding towards the C_{60}, starting with the [10]CPP interacting with one phenyl position of the chain, the same previous difference of distances was used as CV. The metadynamics bias was added every 100 ps, using Gaussian with an initial height of 1 kJ/mol, a width of 0.01 nm and a bias factor of 20. The COMMITTOR checked the value of the CV every 500 steps and stopped the simulation if CV was below 2.2 nm.

The empirical cumulative distribution function (ECDF) obtained from multiple infrequent WT-MetaD runs was fitted with the Poisson distribution expected for rare events:

$$P_{n\geq 1} = 1 - e^{-\frac{t}{\tau}} \quad \text{(Eq. S7)}$$

where τ is the characteristic timescale of the observed transition. The associated kinetic barrier is calculated using the Eyring equation from the characteristic time.

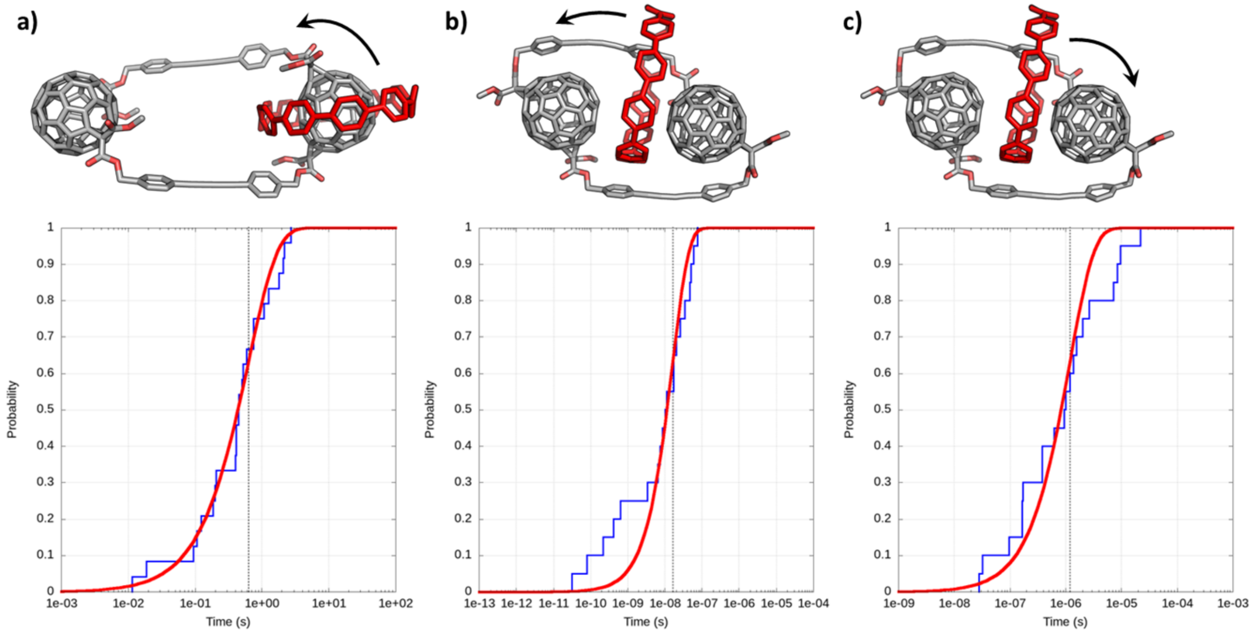

Figure S56: Studied transitions where the [10]CPP movement is indicated by the black arrow. Transition times estimated via multiple infrequent WT-MetaD runs (blue steps) are fitted with Poisson distribution (red curve). The vertical dashed line represents the characteristic timescale τ of the transition. a) Unbinding from the C_{60}. b) Phenyl to phenyl ring transition. c) Binding to the C_{60} from the phenyl ring position.

The ring movement over 0.38 μs can be viewed in Supporting Video S1.

7. References

[S1] CrysAlisPro, Agilent Technologies XRD Products.

[S2] a) G. M. Sheldrick, *Acta Crystallogr. C*, 2015, **71**, 3–8; b) O. V. Dolomanov, L. J. Bourhis, R. J. Gildea, J. A. K. Howard, H. Puschmann, *J. Appl. Crystallogr.*, 2009, **42**, 339–341.

[S3] F. Djojo, A. Herzog, I. Lamparth, F. Hampel, A. Hirsch, *Chem. Eur. J.*, 1996, **2**, 1537–1547.

[S4] Ö. Unsal, A. Godt, *Chem. Eur. J.*, 1999, **5**, 1728–1733.

[S5] L. K. Wasserthal, A. Kratzer, A. Hirsch, Eur. J. Org. Chem., 2013, 2355–2361.

[S6] For selected examples, see: (a) A. Duarte-Ruiz, H. Iuele, S. A. Torres-Cortés, A. Meléndez, J. D. Velásquez and M. N. Chaur, *Rev. Colomb. Quim.*, 2021, **50**, 86–97; (b) A. Herrmann, F. Diederich, C. Thilgen, H.-U. T. Meer and W. H. Müller, *Helv. Chim. Acta*, 1994, **77**, 1689–1706.

[S7] X. Camps and A. Hirsch, *J. Chem. Soc., Perkin Trans. 1*, 1997, 1595–1596.

[S8] J. L. Delgado, E. Espíldora, M. Liedtke, A. Sperlich, D. Rauh, A. Baumann, C. Deibel, V. Dyakonov and N. Martín, *Chem. Eur. J.*, 2009, **15**, 13474–13482.

[S9] M. Urbani, B. Pelado, P. de la Cruz, K. Yamanaka, O. Ito and F. Langa, *Chem. Eur. J.*, 2011, **17**, 5432–5444.

[S10] W. F. Fobare, D. A. Quagliato, W. R. Solvibile, M. S. Malamas, P. Zhou, J. J. Erdei, P. M. Andrae, Y. Yan, I .S. Gunawan, *Amino-5-(5-membered)hetero-arylimidazolone compounds and the use thereof for beta-secretase modulation*. U.S. Pat., US2007004786 A1, 2007, *Wyeth Corp.*

[S11] Y.-Y. Li, T. Wei, C. Liu, Z. Zhang, L.-F. Wu, M. Ding, S. Yuan, J. Zhu, J.-L. Zuo, *Chem. Eur. J.*, 2023, **29**, e202301048.

[S12] E.-K. Yum, J.-W. Son, S.-K. Kim, S.-N. Kim, K.-M. Kim, C.-W. Lee, *Bull. Korean Chem. Soc.*, 2010, **31**, 2097–2099.

[S13] D. Armspach, E. C. Constable, F. Diederich,C. E. Housecroft, J.-F. Nierengarten, *Chem. Eur. J.*, 1998, **4**, 723–733.

[S14] W. W. H. Wong, J. Subbiah, J. M. White, H. Seyler, B. Zhang, D. J. Jones, A. B. Holmes, *Chem. Mater.*, 2014, **26**, 1686–1689.

[S15] H. Günther, *NMR Spectroscopy*, Wiley-VCH, Weinheim, Germany, 3rd edn., 2013.

[S16] A. Hirsch, I. Lamparth, H. R. Karfunkel, *Angew. Chem. Int. Ed.*, 1994, **33**, 437–438.

[S17] F. M. Steudel, E. Ubasart, L. Leanza, M. Pujals, T. Parella, G. M. Pavan, X. Ribas, M. von Delius, *Angew. Chem. Int. Ed.*, 2023, **62**, e202309393.

[S18] (a) P. Thordarson, *Chem. Soc. Rev.*, 2011, **40**, 1305–1323; (b) D. B. Hibbert, P. Thordarson, *Chem. Commun.*, 2016, **52**, 12792–12805.

[S19] J. Wang, R. M. Wolf, J. W. Caldwell, P. A. Kollman, D. A. Case, *J. Comput. Chem.*, 2004, **25**, 1157–1174.

[S20] C. I. Bayly, P. Cieplak, W. Cornell, P. A. Kollman, *J. Phys. Chem.*, 1993, **97**, 10269–10280.

[S21] Gaussian 16, Revision A.03, Gaussian Inc.: Wallingford CT, 2016

[S22] M. J. Abraham, T. Murtola, R. Schulz, S. Páll, J. C. Smith, B. Hess, E. Lindahl, *SoftwareX*, 2015, **1–2**, 19–25.

[S23] a) The PLUMED consortium, *Nat. Methods*, 2019, **16**, 670–673; b) G. A. Tribello, M. Bonomi, D. Branduardi, C. Camilloni, G. Bussi, *Comput. Phys. Commun.*, 2014, **185**, 604–613.

[S24] A. Barducci, G. Bussi, M. Parrinello, *Phys. Rev. Lett.*, 2008, **100**, 020603–020606.

8. Spectra

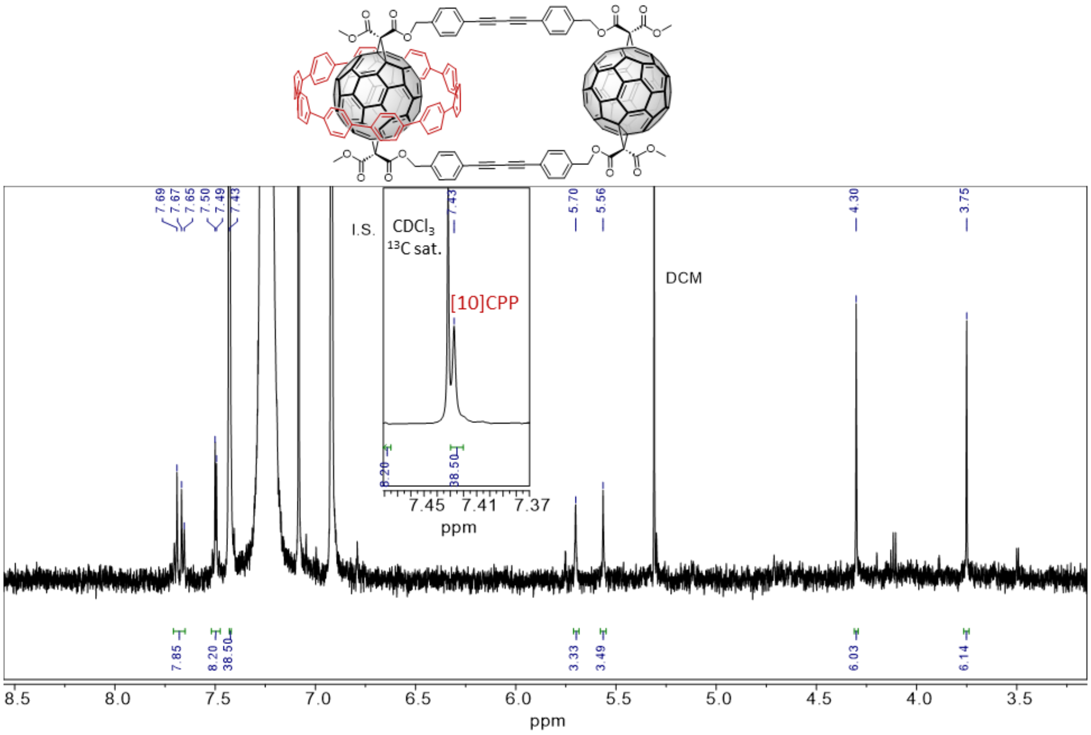

Figure S57: ^{1}H NMR (600 MHz, $CDCl_3$, 263 K) spectrum of (*out*,*out-trans*-1)$_2$ [2]catenane **1a**. Contains 1,2,4,5-tetramethylbenzene as internal standard (I.S.).

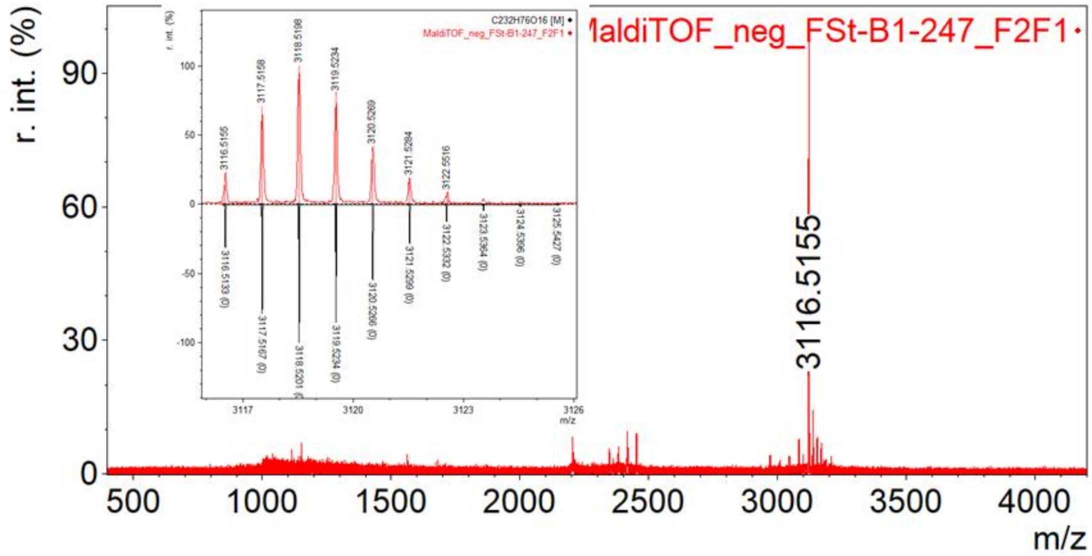

Figure S58: HRMS-MALDI spectrum of compound **1a** and comparison with the calculated isotopic pattern (black).

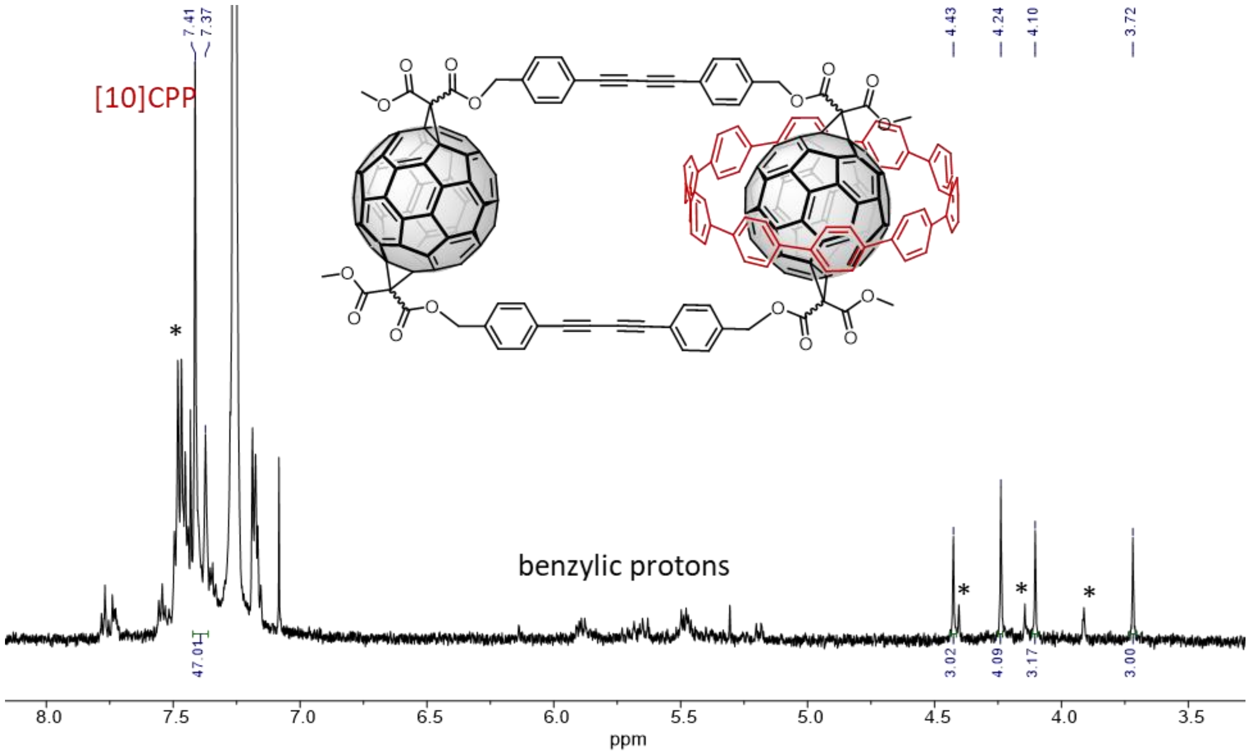

Figure S59: ^{1}H NMR (600 MHz, $CDCl_3$, 273 K) spectrum of (*in,out-trans*-2)$_2$ [2]catenane **1b**. Due to the low concentration of the compound and overlap with *impurities the aromatic and benzylic protons could not be assigned.

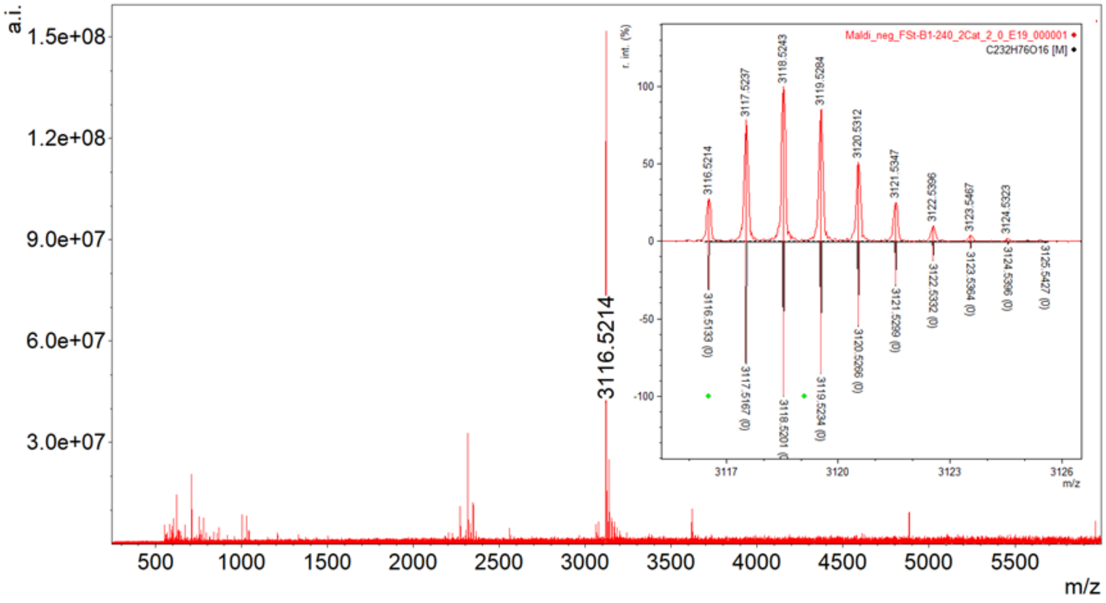

Figure S60: HRMS-MALDI spectrum of (*in*,*out-trans*-2)$_2$ [2]catenane **1b** and comparison with the calculated isotopic pattern (black).

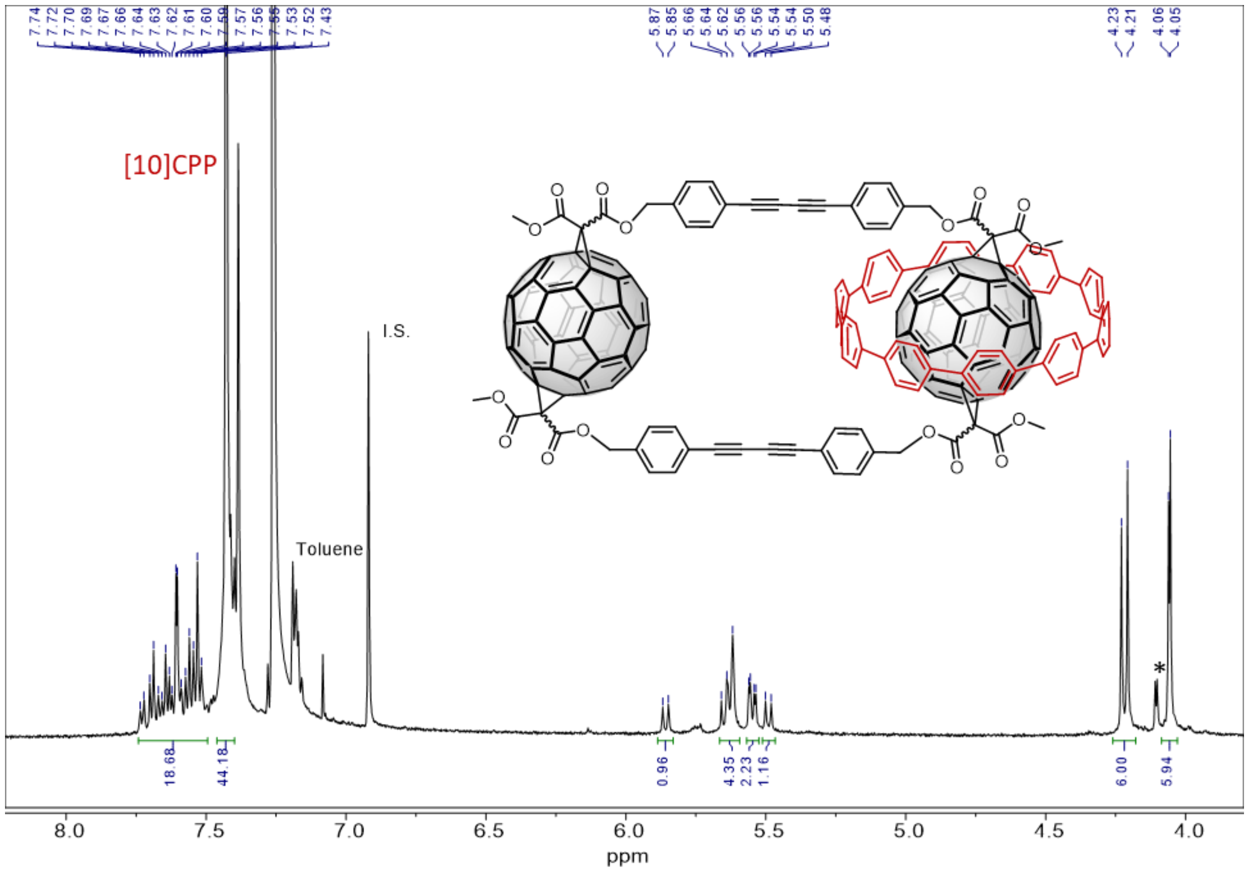

Figure S61: ^{1}H NMR (600 MHz, $CDCl_3$, 263 K) spectrum of (*in*,*in*-*trans*-2)$_2$ [2]catenane **1d**. Contains 1,2,4,5-tetramethylbenzene as internal standard (I.S.).

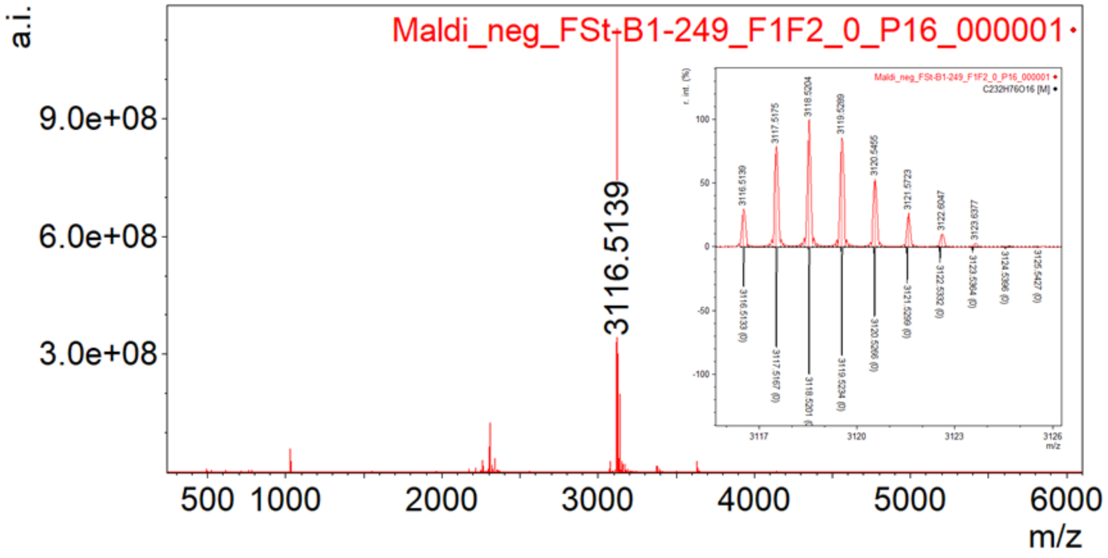

Figure S62: HRMS-MALDI spectrum of compound **1d** and comparison with the calculated isotopic pattern (black).

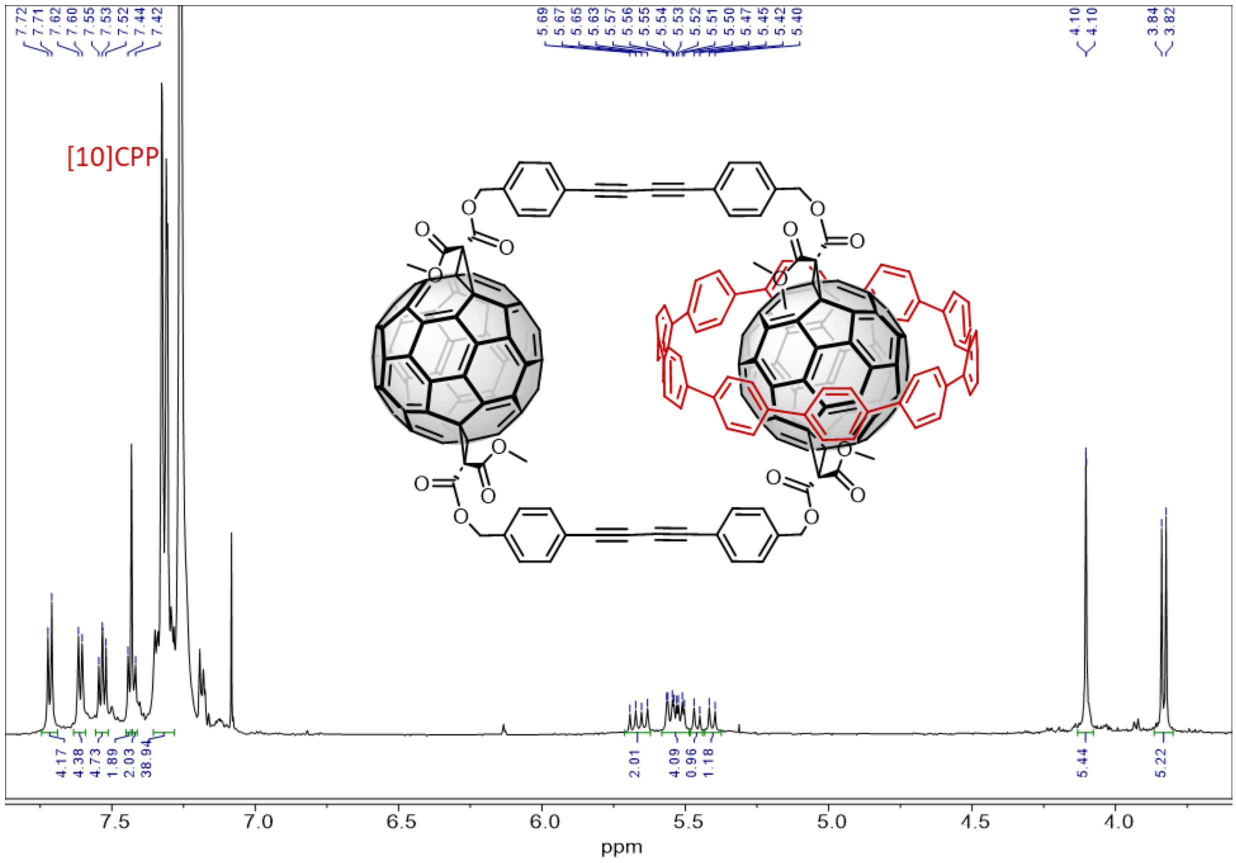

Figure S63: ^{1}H NMR (600 MHz, $CDCl_3$, 253 K) spectrum of (*in*,*in*-*trans*-3)$_2$ [2]catenane **1g**.

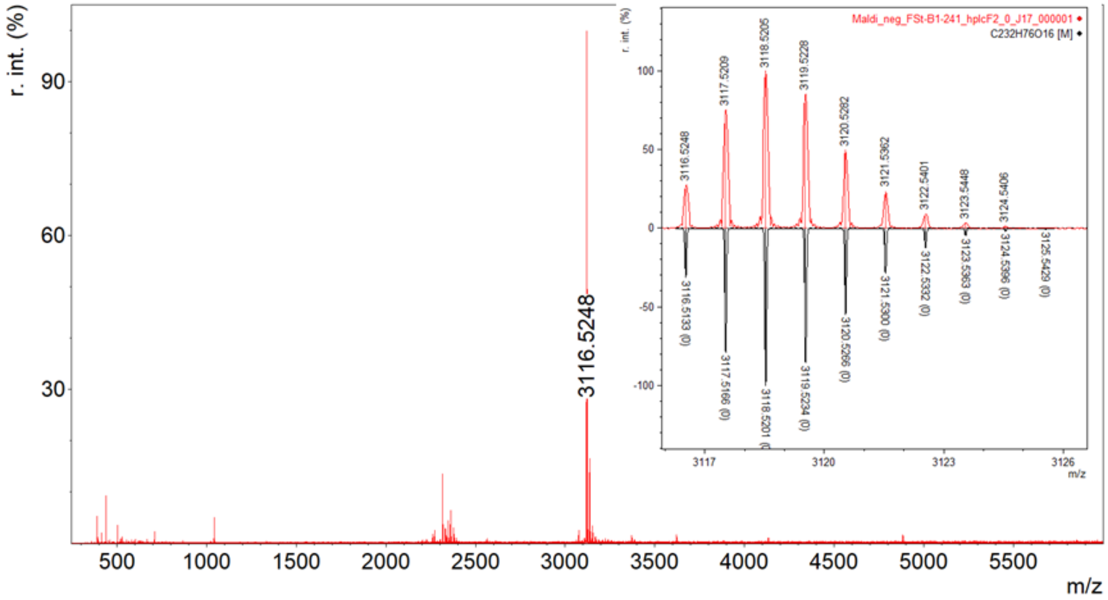

Figure S64: HRMS-MALDI spectrum of compound **1g** and comparison with the calculated isotopic pattern (black).

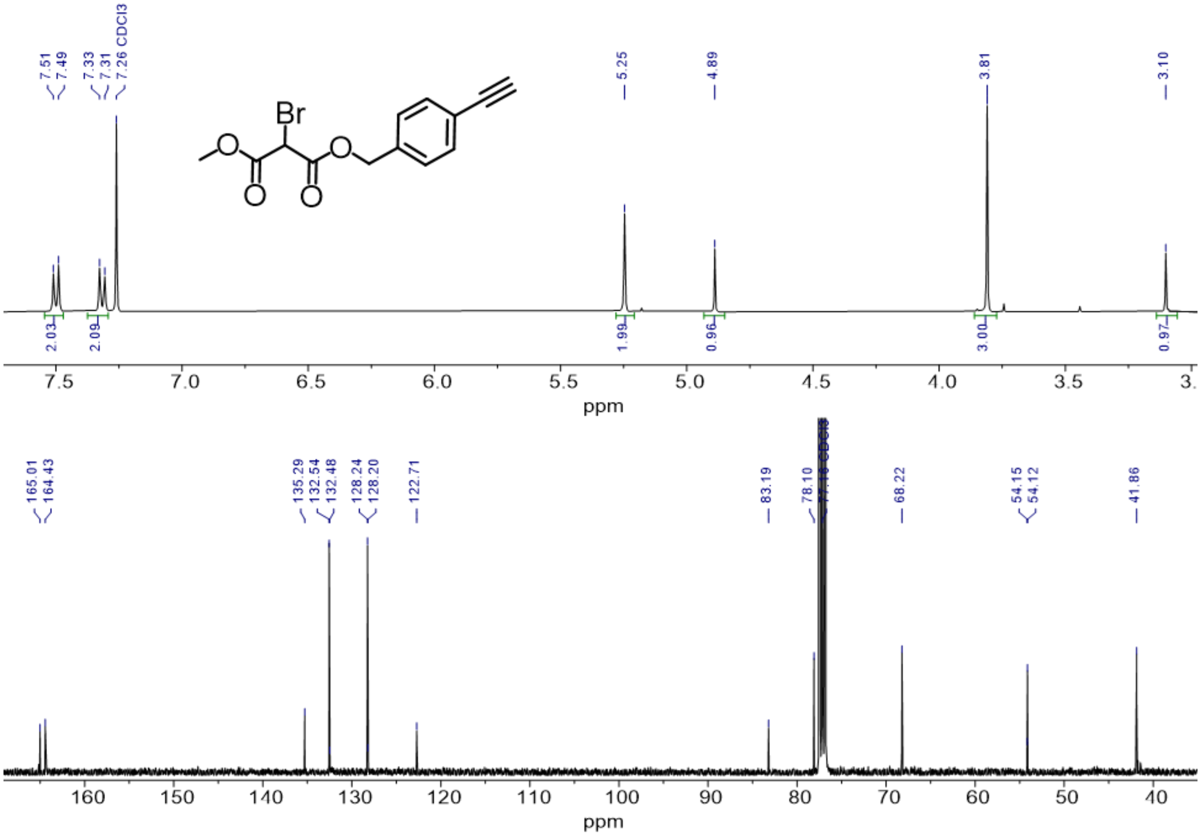

Figure S65: ^{1}H NMR (400 MHz, $CDCl_3$, 298 K) and ^{13}C NMR (101 MHz, $CDCl_3$, 298 K) spectra of bromomalonate **2**.

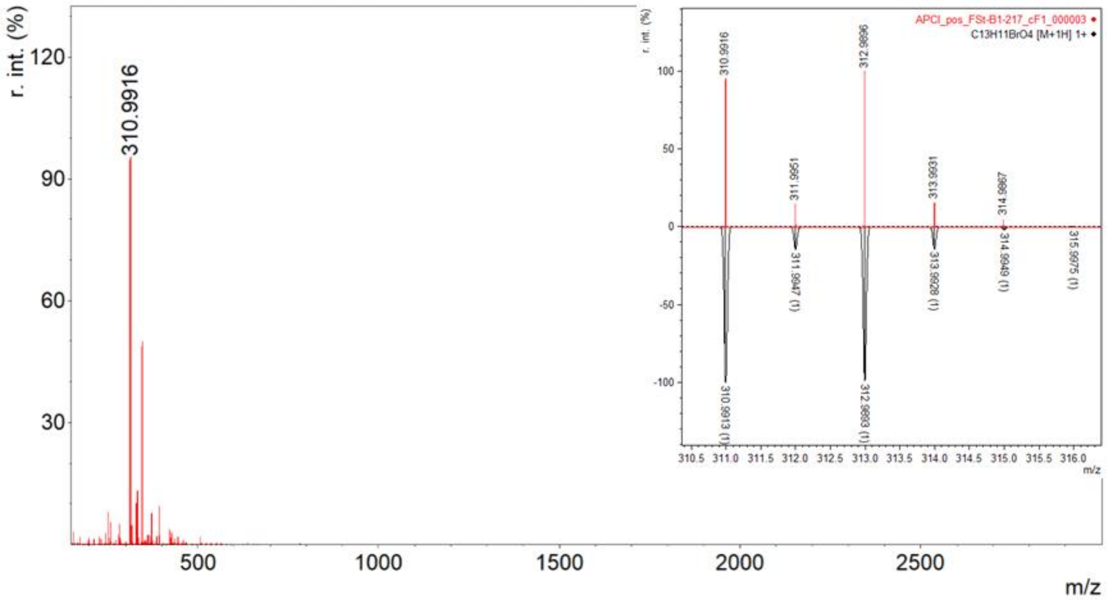

Figure S66: HRMS-APCI spectrum of bromomalonate **2** and comparison with the calculated isotopic pattern (black).

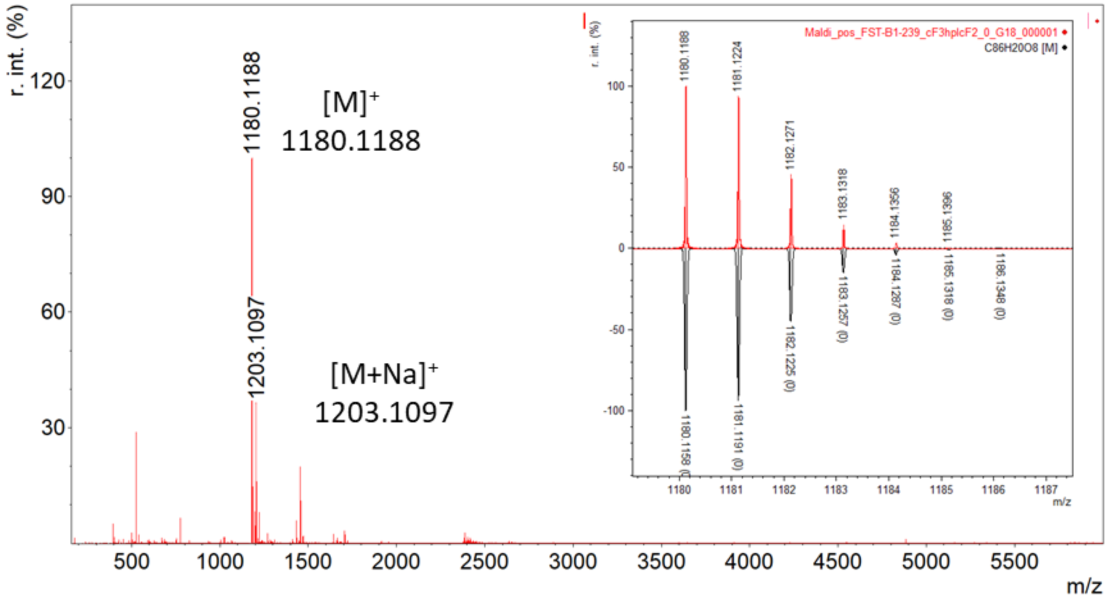

Figure S67: HRMS-MALDI spectrum of bis-adducts **3** and comparison with the calculated isotopic pattern (black).

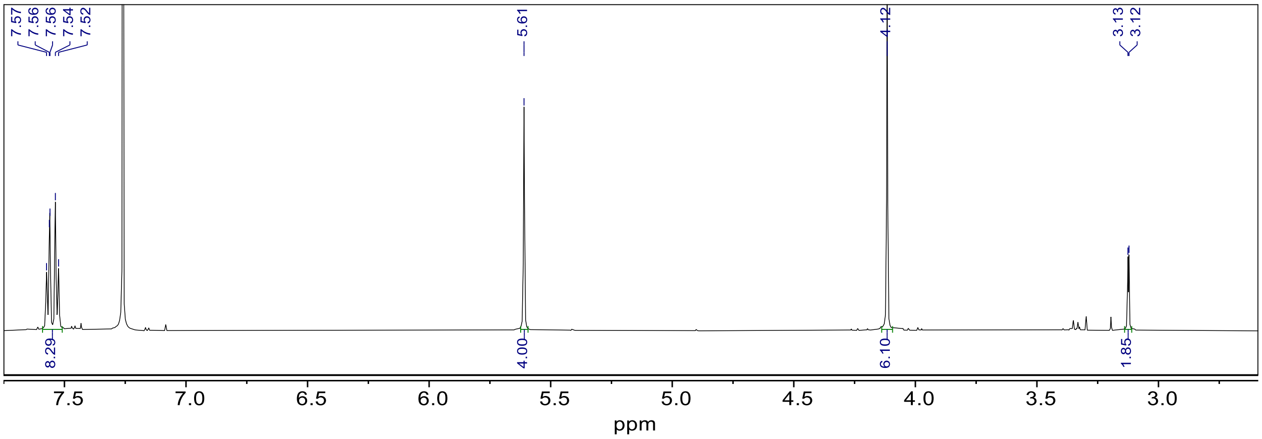

Figure S68: ^{1}H NMR (600 MHz, $CDCl_3$, 298 K) spectrum of *out*,*out-trans*-1 bis-adduct **3a**.

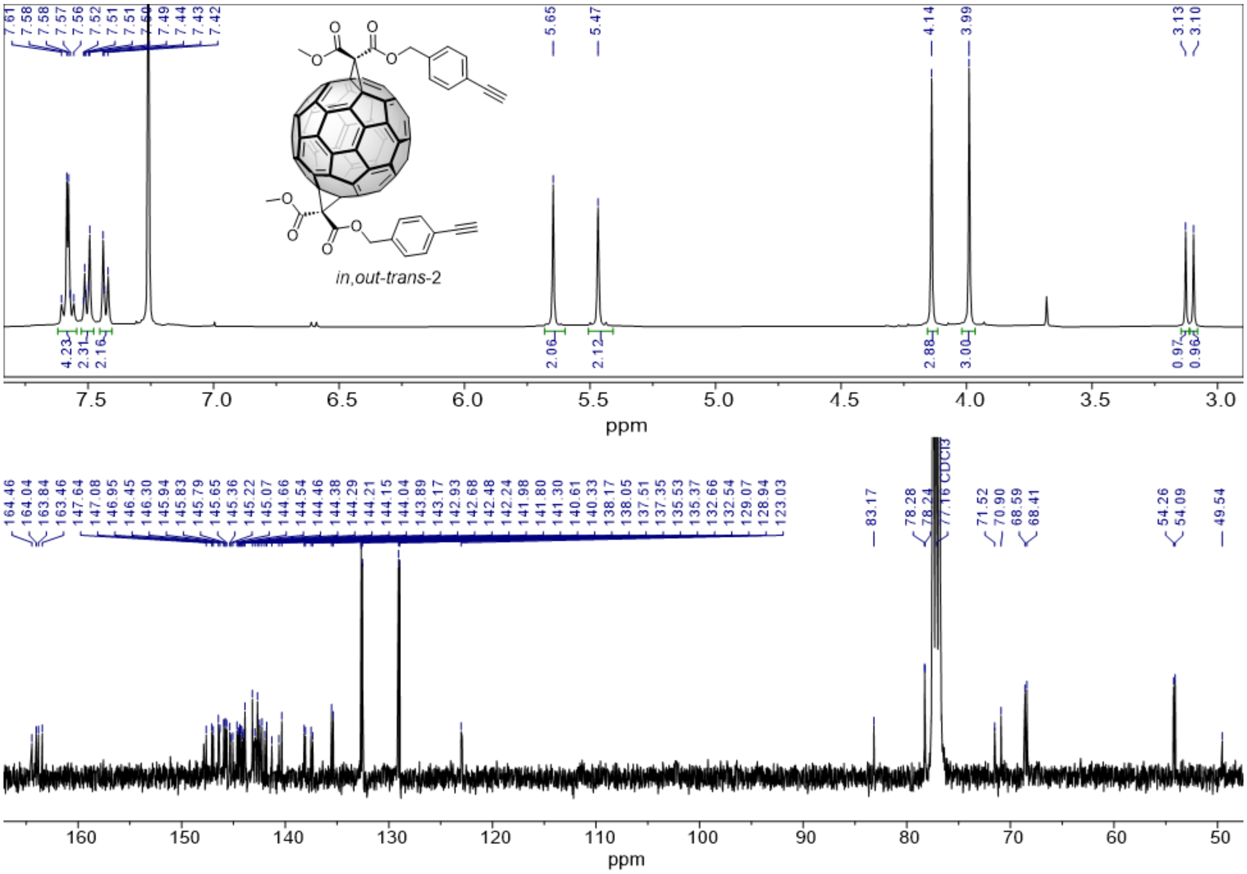

Figure S69: ^{1}H NMR (400 MHz, $CDCl_3$, 298 K) and ^{13}C NMR (101 MHz, $CDCl_3$, 298 K) spectra of *in*,*out*-*trans*-2 bis-adduct **3b**.

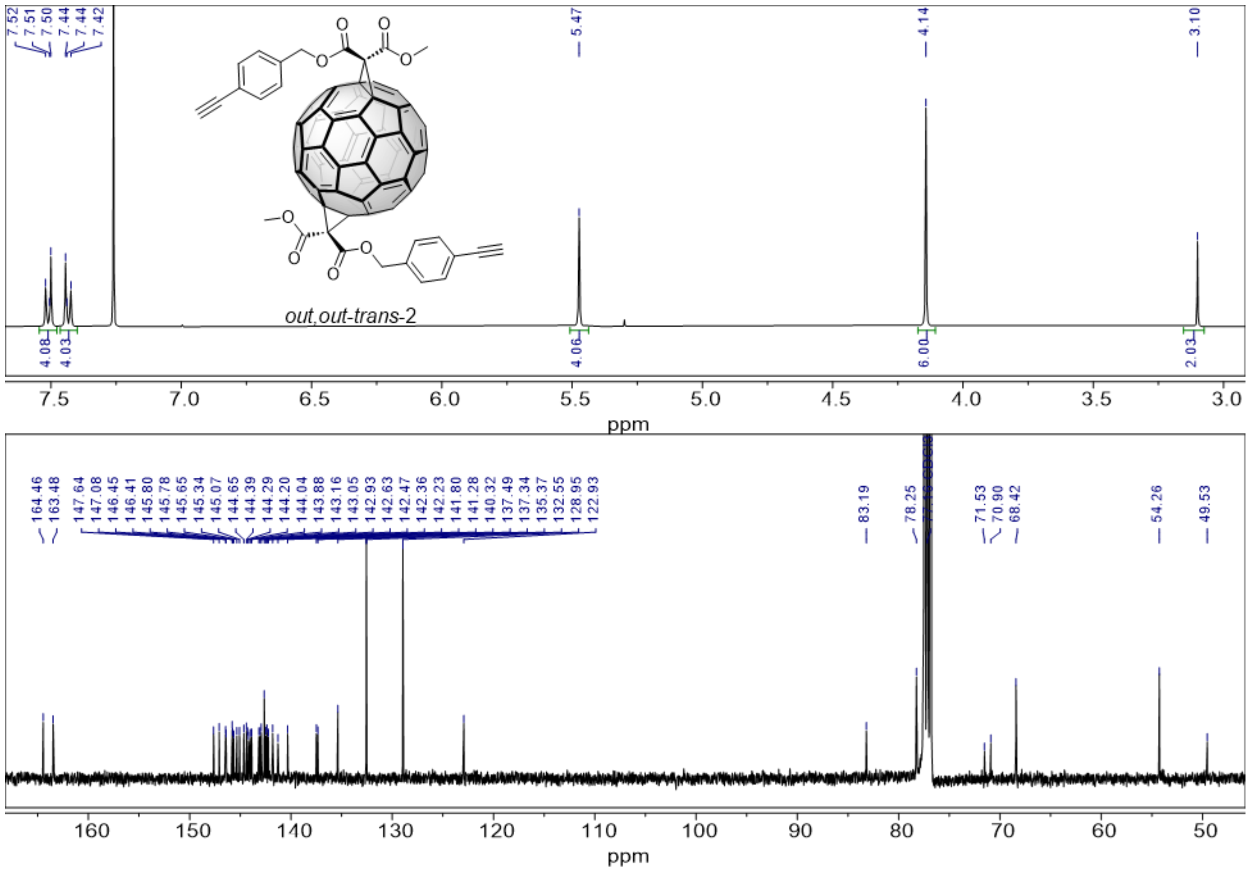

Figure S70: ^{1}H NMR (400 MHz, $CDCl_3$, 298 K) and ^{13}C NMR (101 MHz, $CDCl_3$, 298 K) spectra of *out*,*out*-*trans*-2 bis-adduct **3c**.

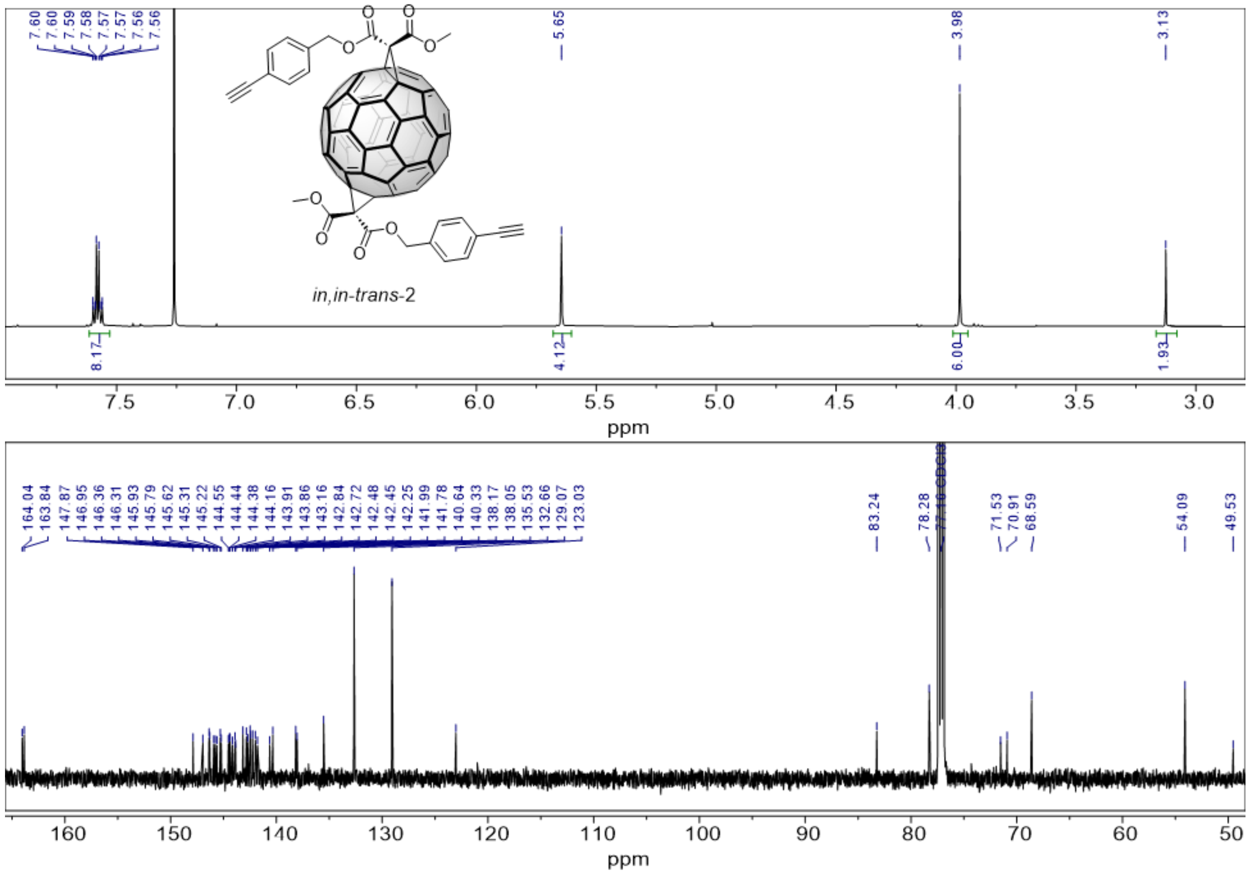

Figure S71: ^{1}H NMR (600 MHz, $CDCl_3$, 298 K) and ^{13}C NMR (151 MHz, $CDCl_3$, 298 K) spectra of *in*,*in*-*trans*-2 bis-adduct **3d**.

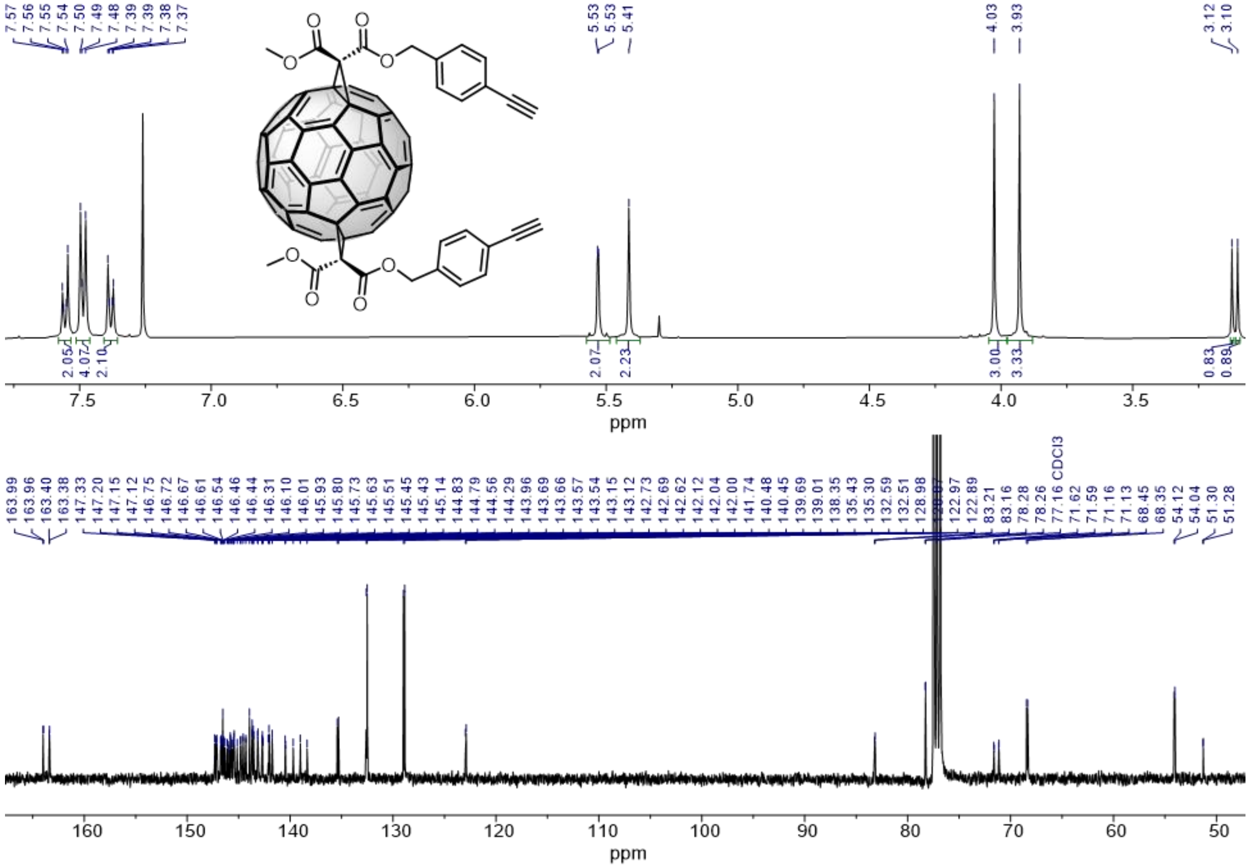

Figure S72: ^{1}H NMR (400 MHz, $CDCl_3$, 298 K) and ^{13}C NMR (101 MHz, $CDCl_3$, 298 K) spectra of *in*,*out*-*trans*-3 bis-adduct **3e**.

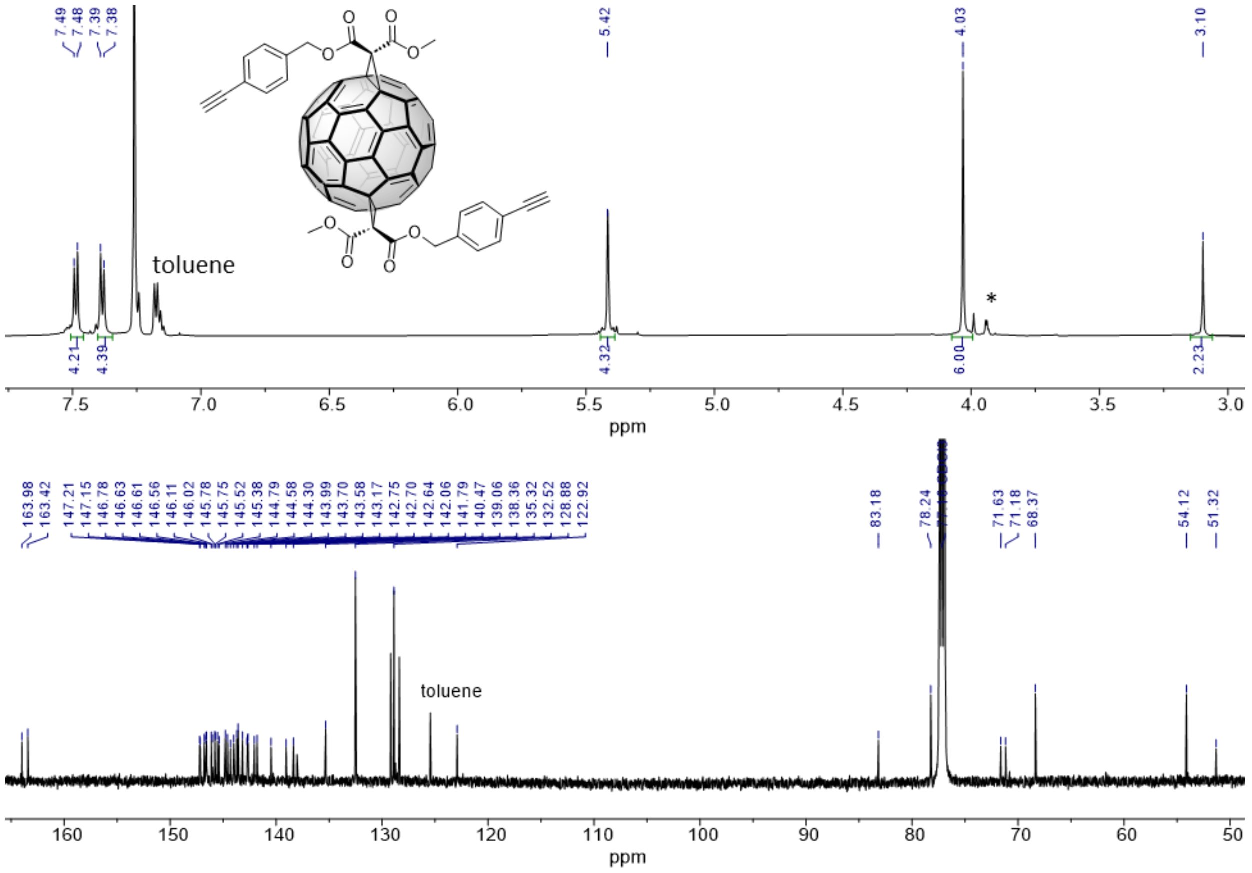

Figure S73: ^{1}H NMR (600 MHz, $CDCl_3$, 298 K) and ^{13}C NMR (151 MHz, $CDCl_3$, 298 K) spectra of *out*,*out*-*trans*-3 bis-adduct **3f**. *Denotes minor impurities in the spectrum.

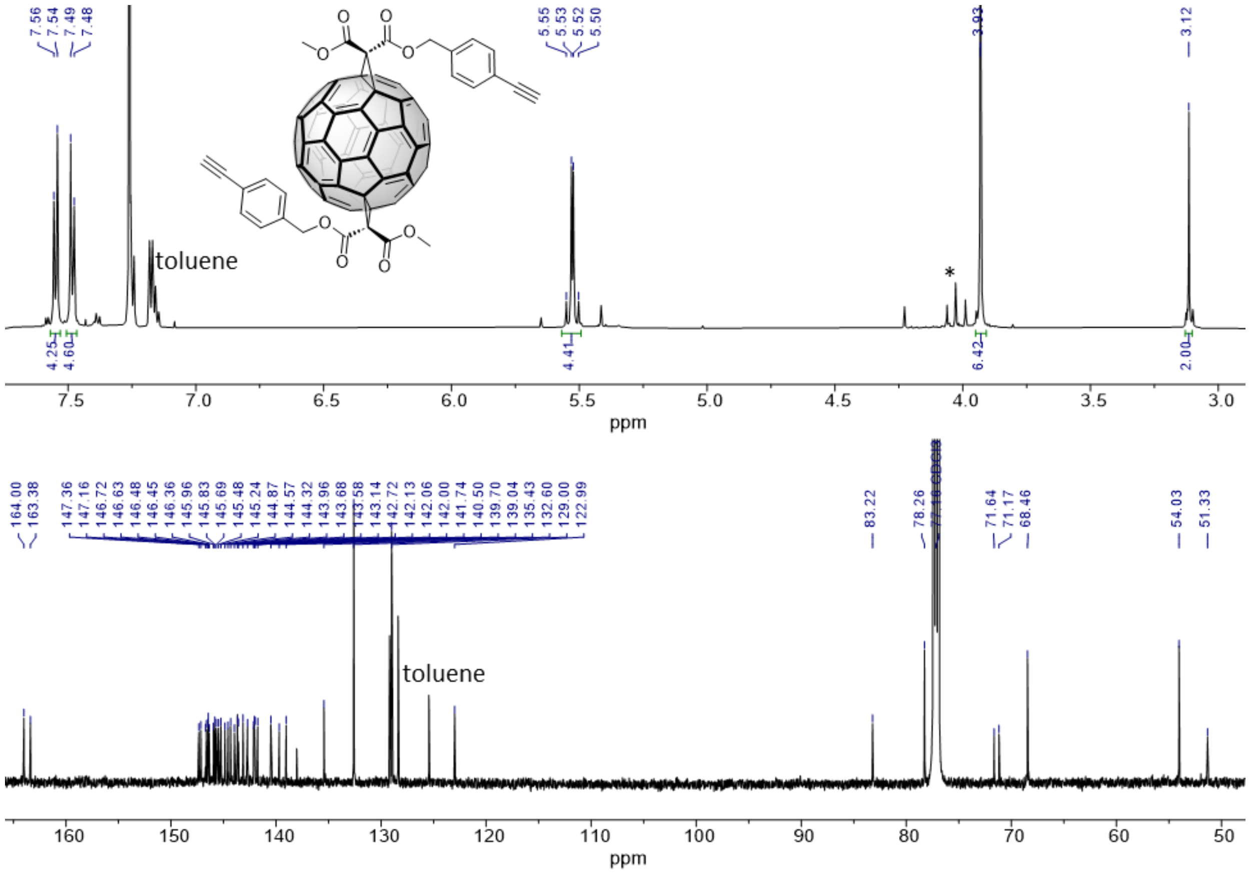

Figure S74: ^{1}H NMR (600 MHz, $CDCl_3$, 298 K) and ^{13}C NMR (151 MHz, $CDCl_3$, 298 K) spectra of *in*,*in*-*trans*-3 bis-adduct **3g**. *Denotes minor impurities in the spectrum.

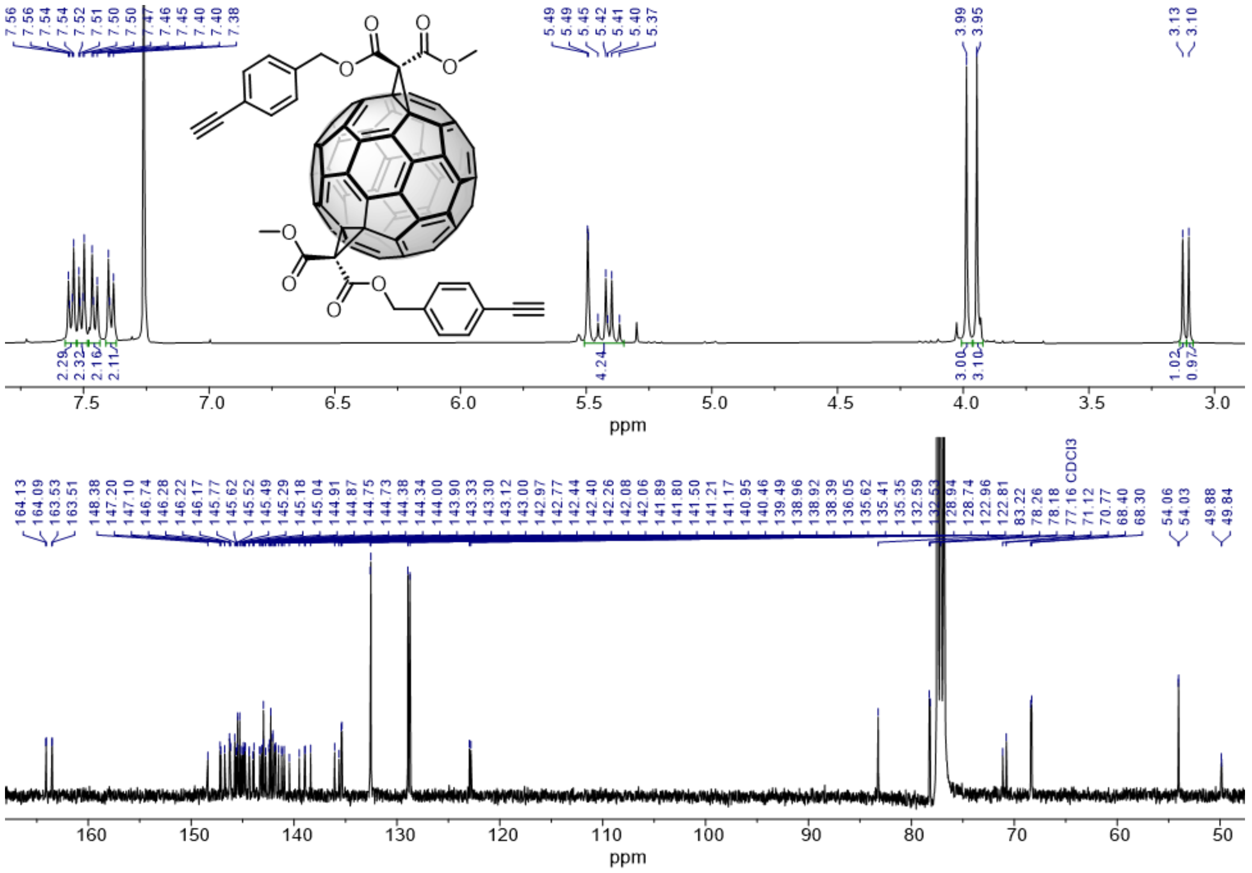

Figure S75: ^{1}H NMR (400 MHz, $CDCl_3$, 298 K) and ^{13}C NMR (101 MHz, $CDCl_3$, 298 K) spectra of *in*,*out*-*trans*-4 bis-adduct **3h**.

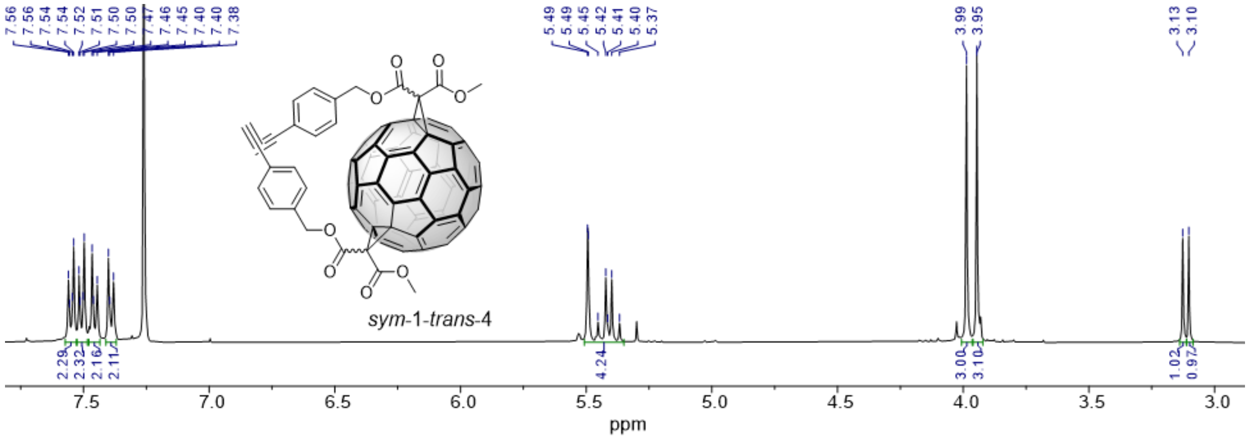

Figure S76: ^{1}H NMR (400 MHz, $CDCl_3$, 298 K) spectrum of *trans*-4 (b) bis-adduct **3i**.

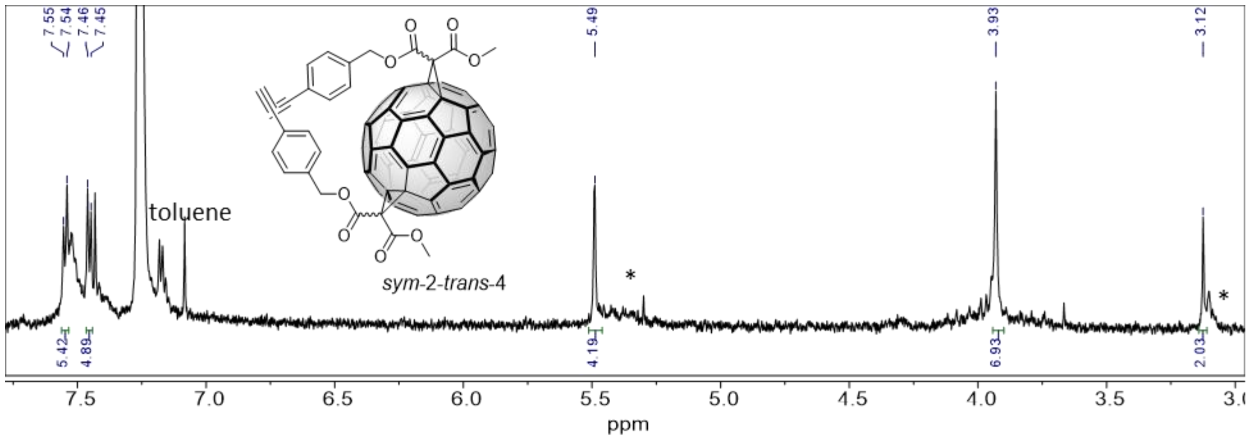

Figure S77: ^{1}H NMR (600 MHz, $CDCl_3$, 298 K) spectrum of *trans*-4 (c) bis-adduct **3j**. *Impurities.

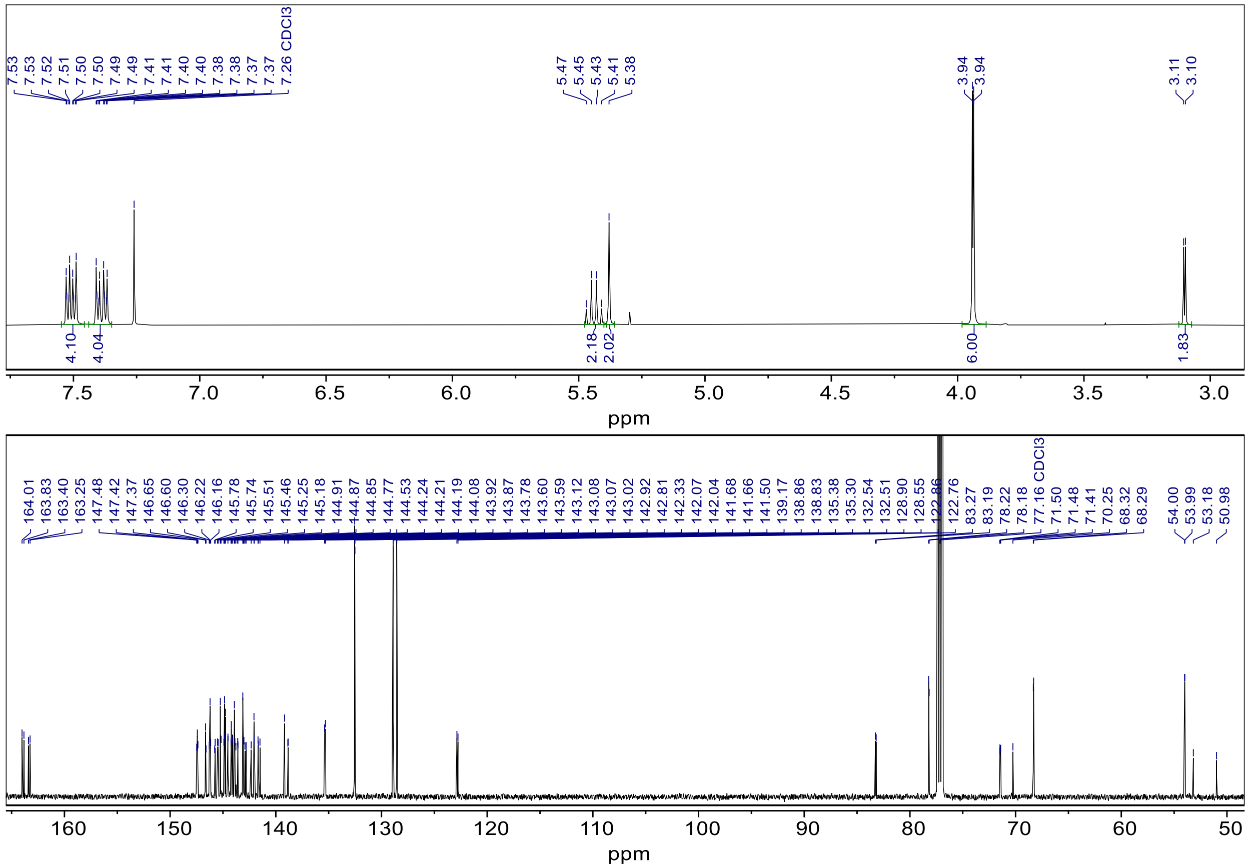

Figure S78: ^{1}H NMR (600 MHz, $CDCl_3$, 298 K) and ^{13}C NMR (151 MHz, $CDCl_3$, 298 K) spectra of *equatorial* (a) bis-adduct **3k**.

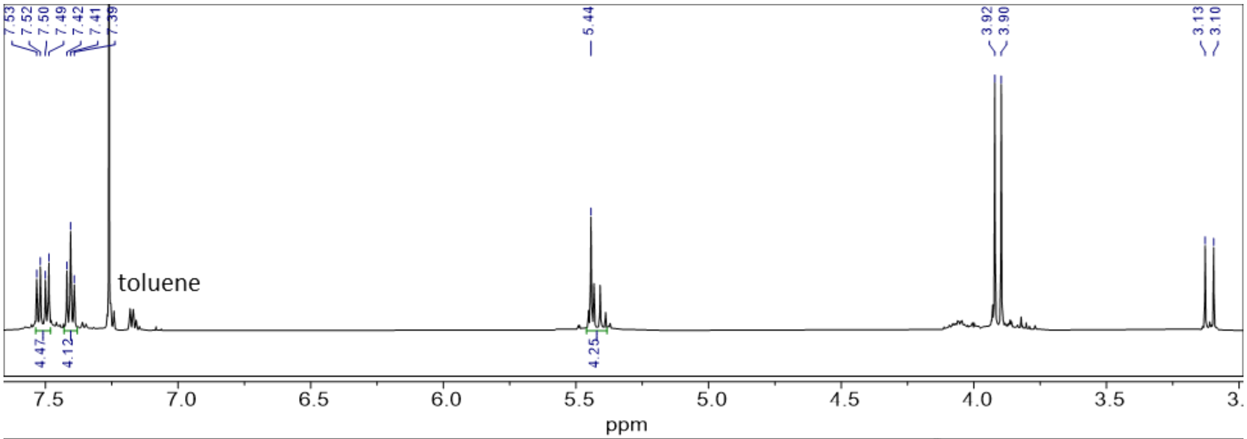

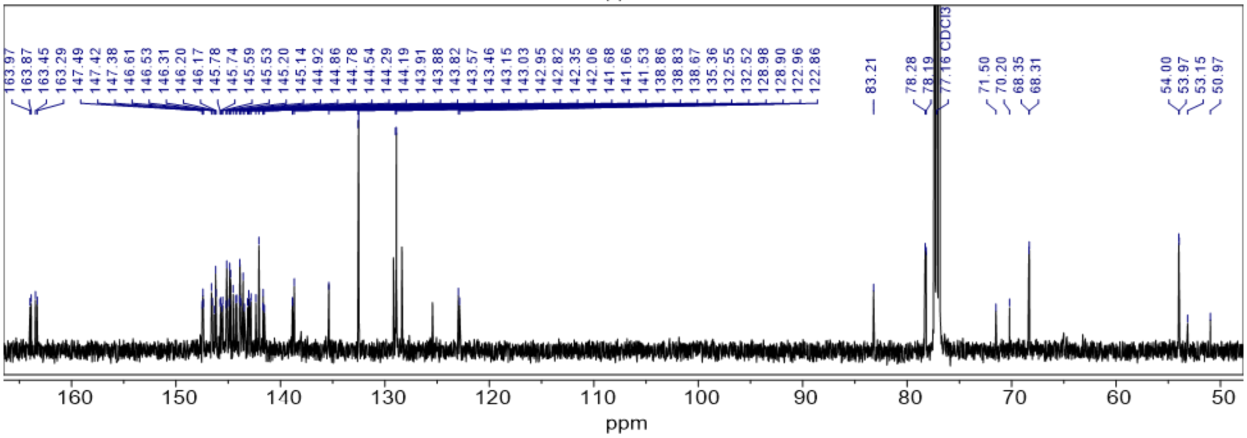

Figure S79: ^{1}H NMR (600 MHz, $CDCl_3$, 298 K) and ^{13}C NMR (151 MHz, $CDCl_3$, 298 K) spectra of *equatorial* (b) bis-adduct **3m**.

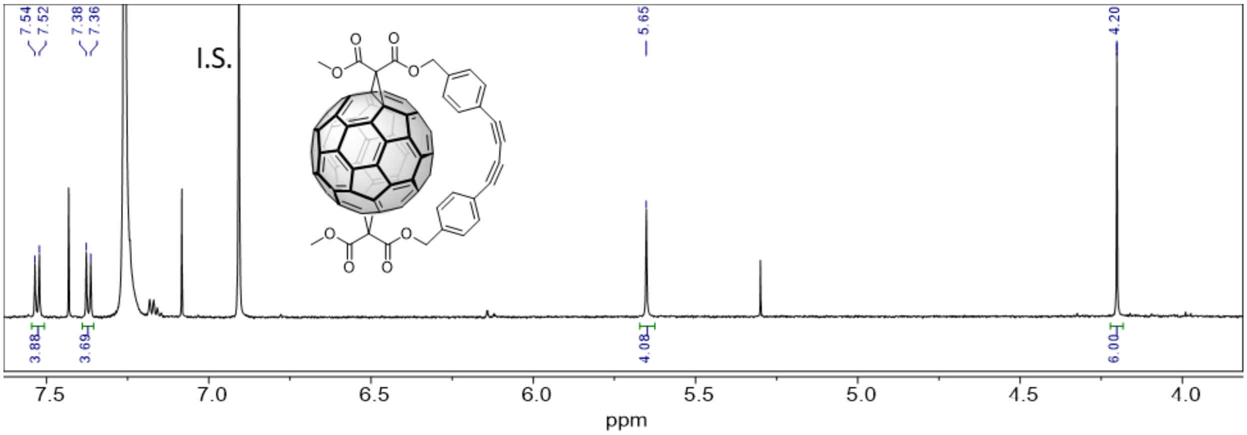

Figure S80: ^{1}H NMR (600 MHz, $CDCl_3$, 298 K) spectrum of **4a.** Contains 1,2,4,5-tetramethylbenzene as internal standard (I.S.).

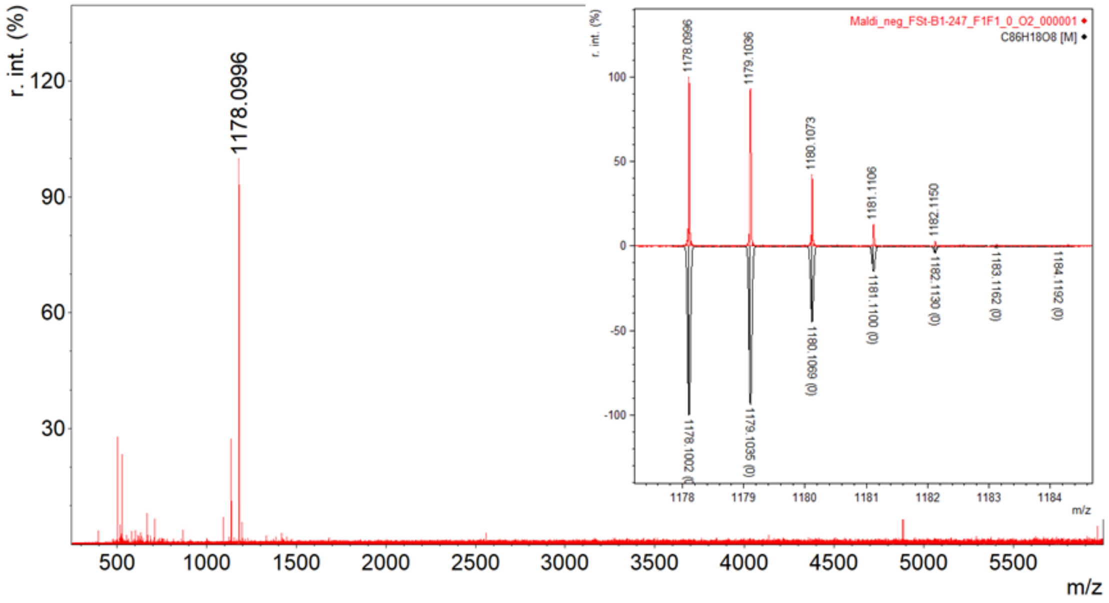

Figure S81: HRMS-MALDI spectrum of **4a** and comparison with the calculated isotopic pattern (black).

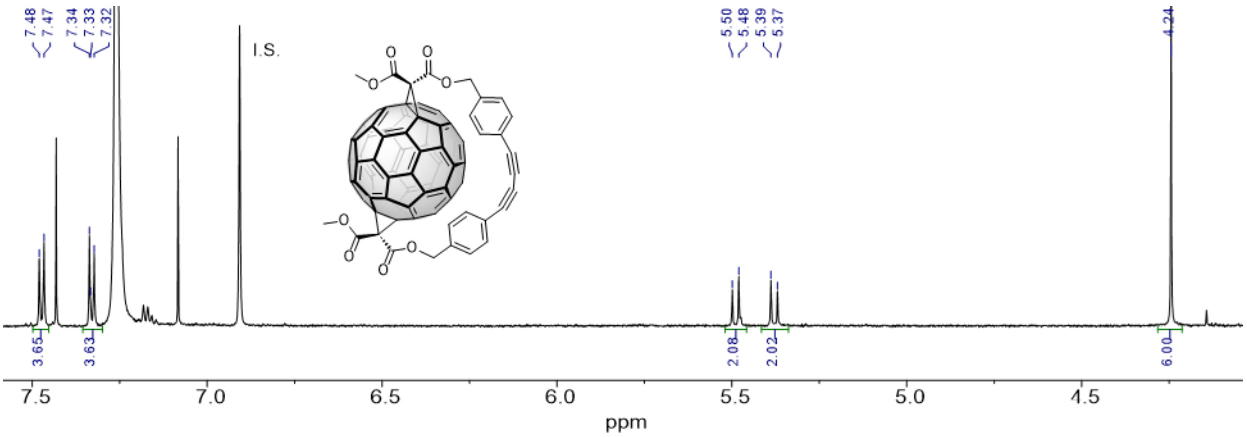

Figure S82: ^{1}H NMR (600 MHz, $CDCl_3$, 298 K) spectrum of **4c**. Contains 1,2,4,5-tetramethylbenzene as internal standard (I.S.).

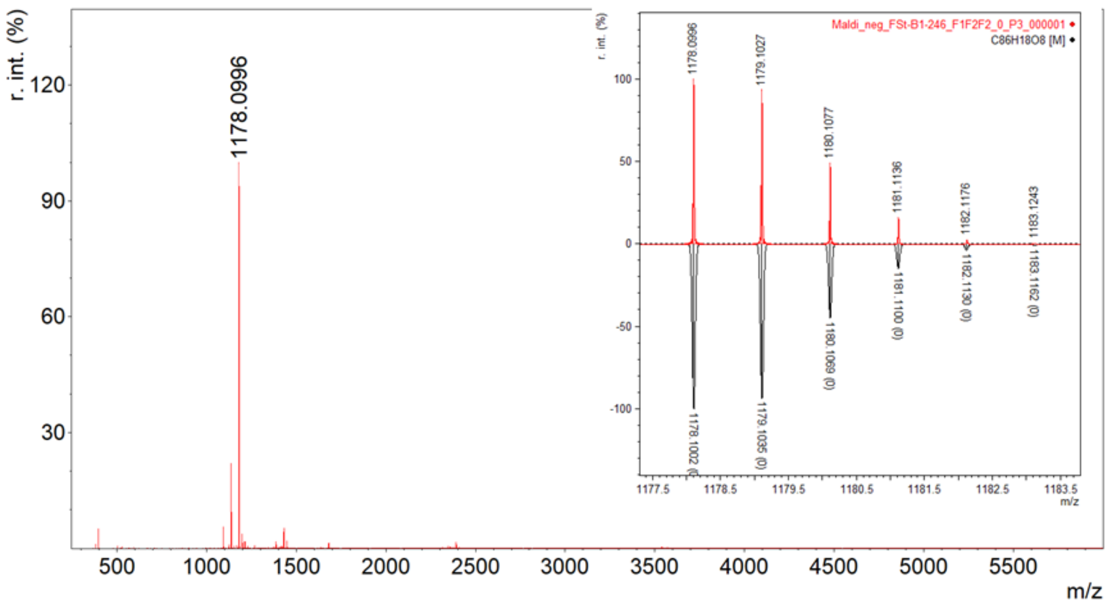

Figure S83: HRMS-MALDI spectrum of **4c** and comparison with the calculated isotopic pattern (black).

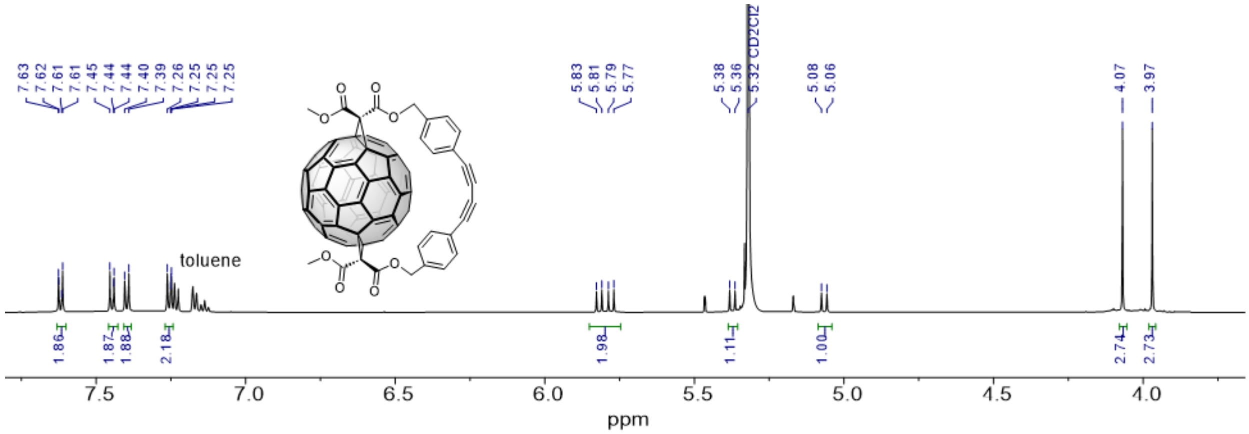

Figure S84: ^{1}H NMR (600 MHz, CD_2Cl_2, 298 K) spectrum of **4e**.

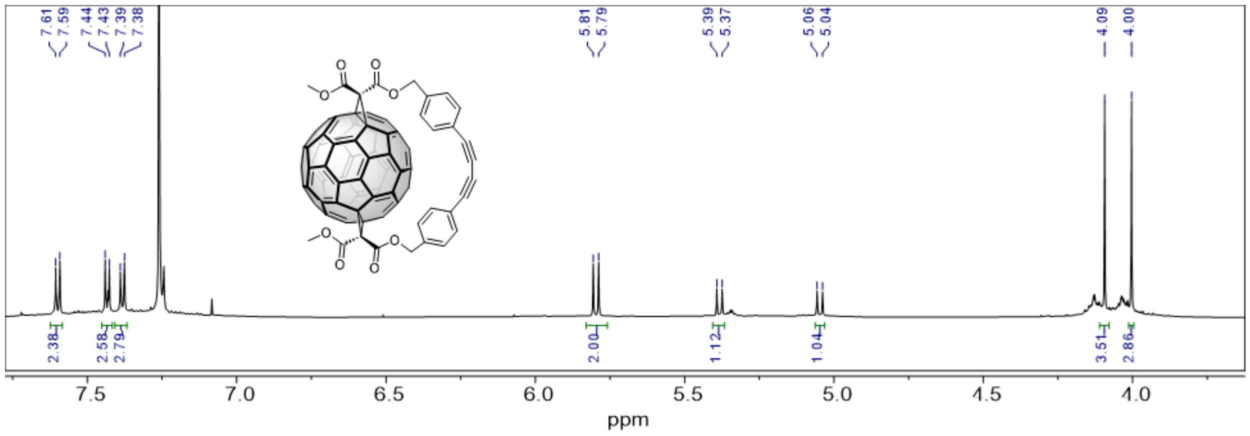

Figure S85: ^{1}H NMR (600 MHz, $CDCl_3$, 298 K) spectrum of **4e**. Slight signs of degradation are visible around the methyl ester signals at 4.1 ppm.

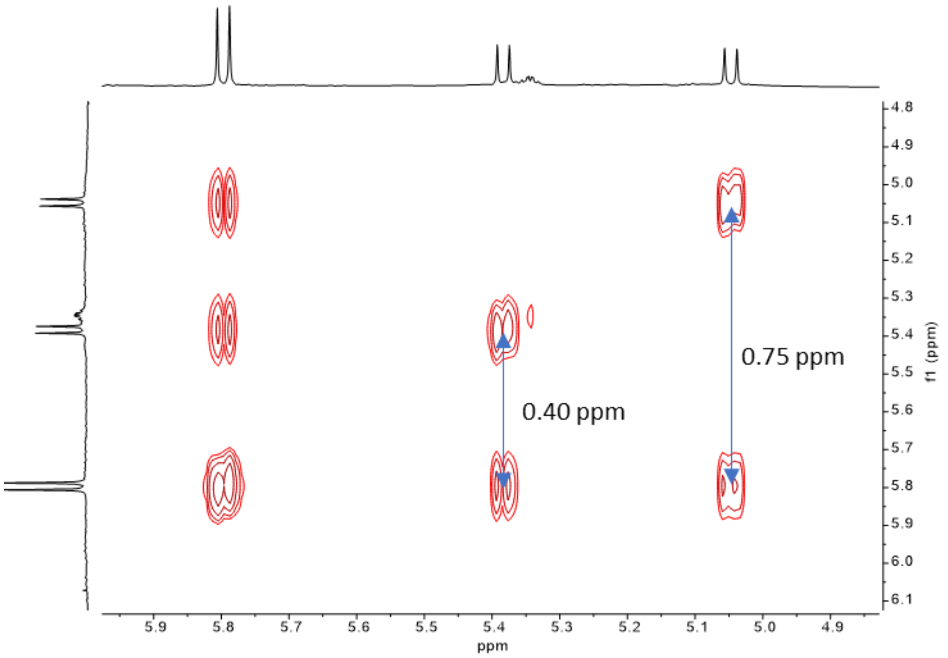

Figure S86: Zoomed-in region of the COSY (600 MHz, $CDCl_3$, 298 K) spectrum of **4e**, showing geminal coupling between diastereotopic benzylic protons with chemical shift separations of approximately 0.40 and 0.75 ppm arising from the rigid structure and proximity to the fullerene.

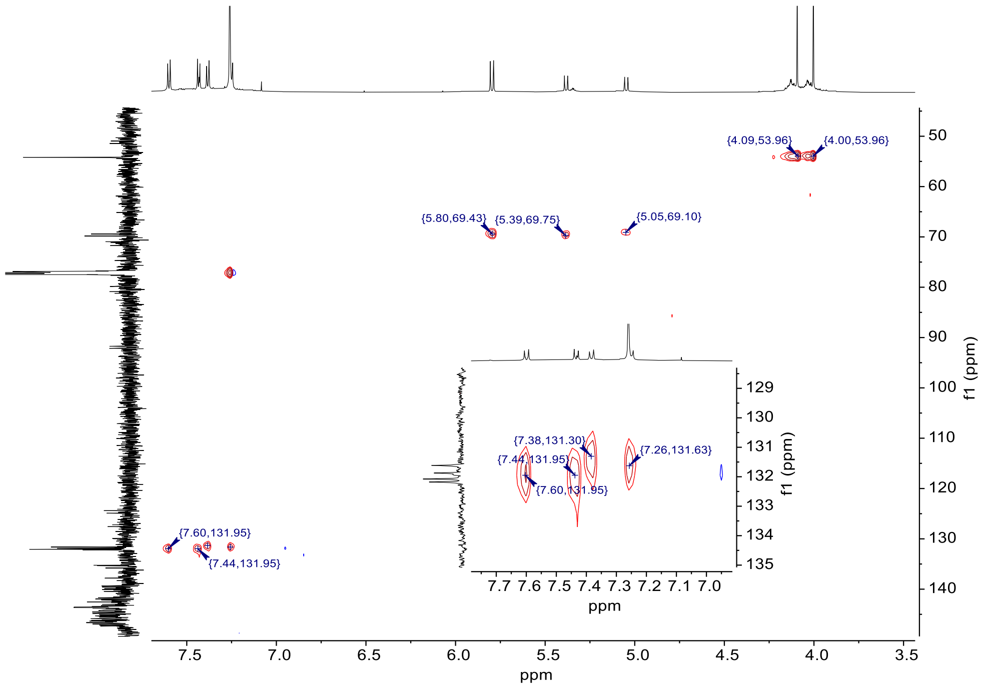

Figure S87: HSQC spectrum (^{1}H–^{13}C) of compound **4e** in $CDCl_3$ at 298 K, recorded at 600 MHz (^{1}H) and 151 MHz (^{13}C).

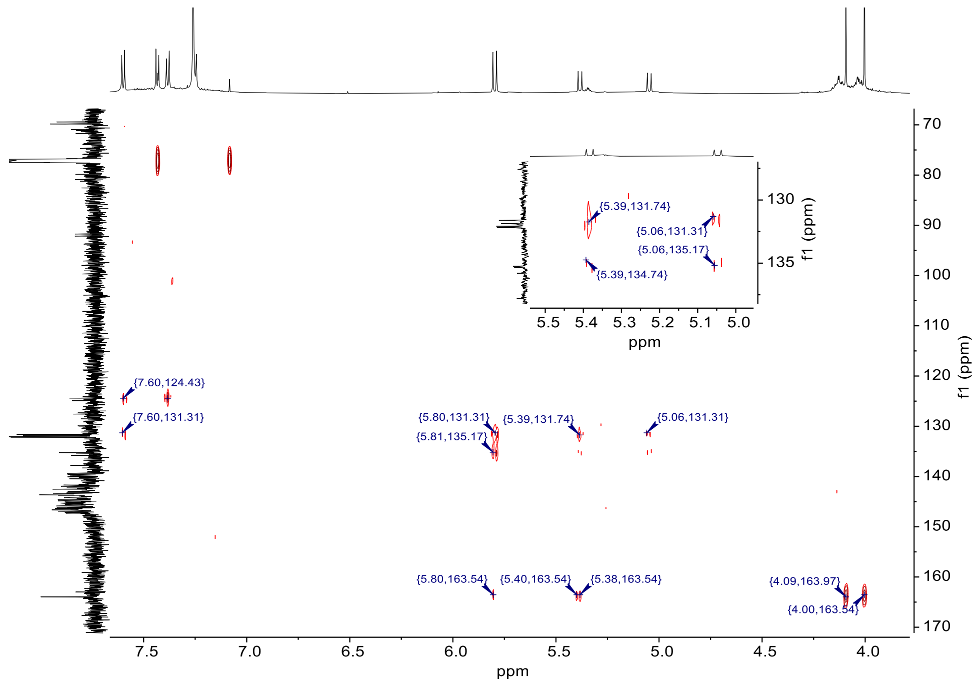

Figure S88: HMBC spectrum (^{1}H–^{13}C) of compound **4e** in $CDCl_3$ at 298 K, recorded at 600 MHz (^{1}H) and 151 MHz (^{13}C).

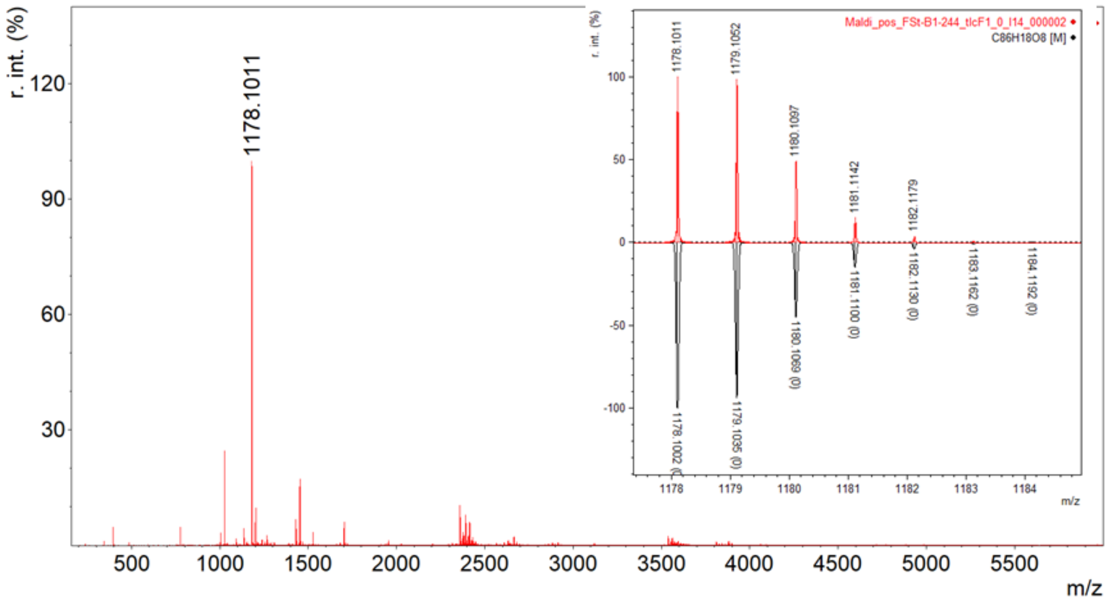

Figure S89: HRMS-MALDI spectrum of **4e** and comparison with the calculated isotopic pattern (black).

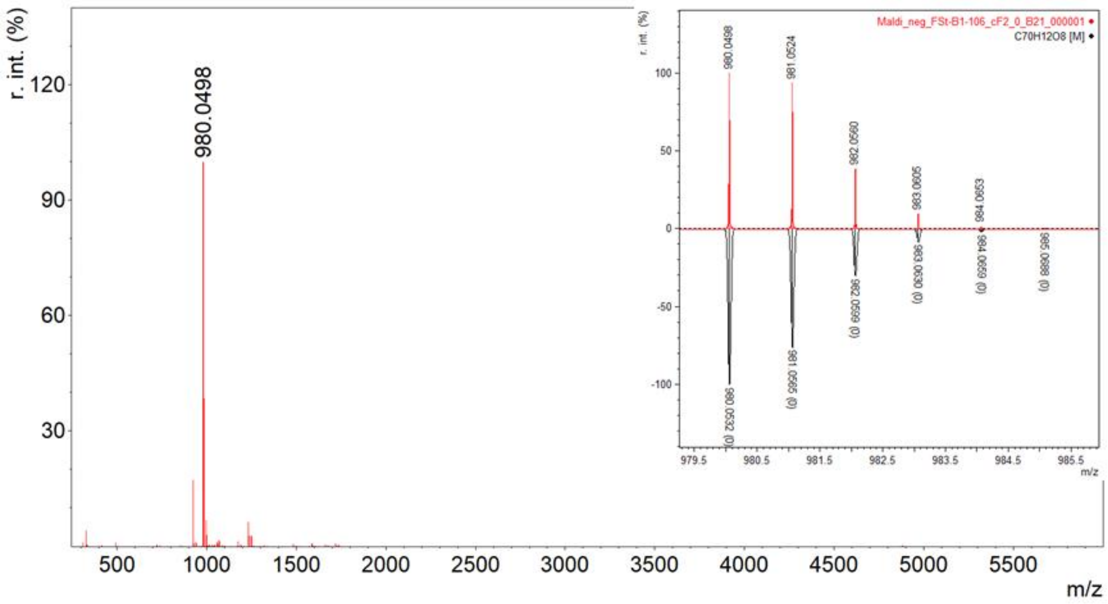

Figure S90: HRMS-MALDI spectrum of **5** and comparison with the calculated isotopic pattern(black).

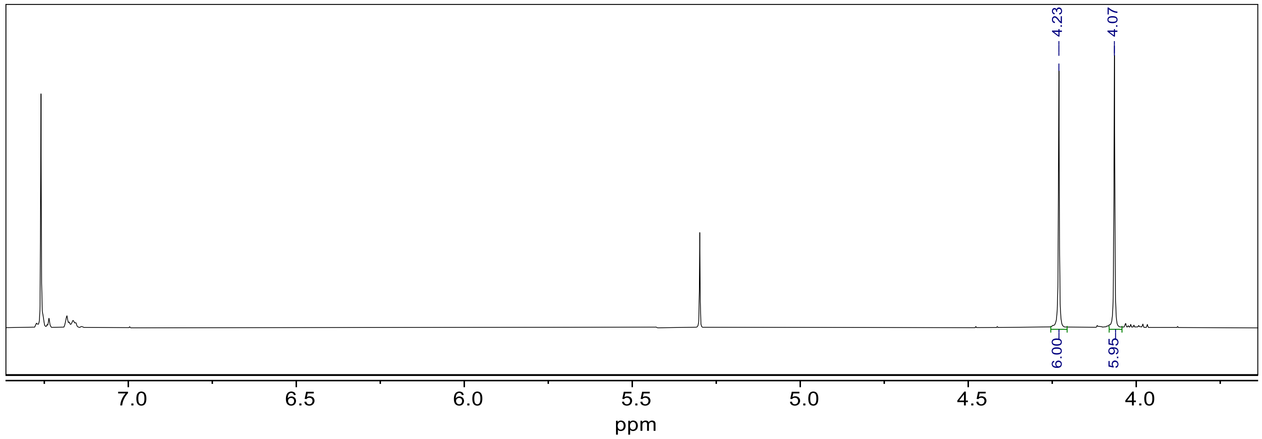

Figure S91: ^{1}H NMR (400 MHz, $CDCl_3$, 298 K) spectrum of the *trans*-2 bis-adduct **5a**.

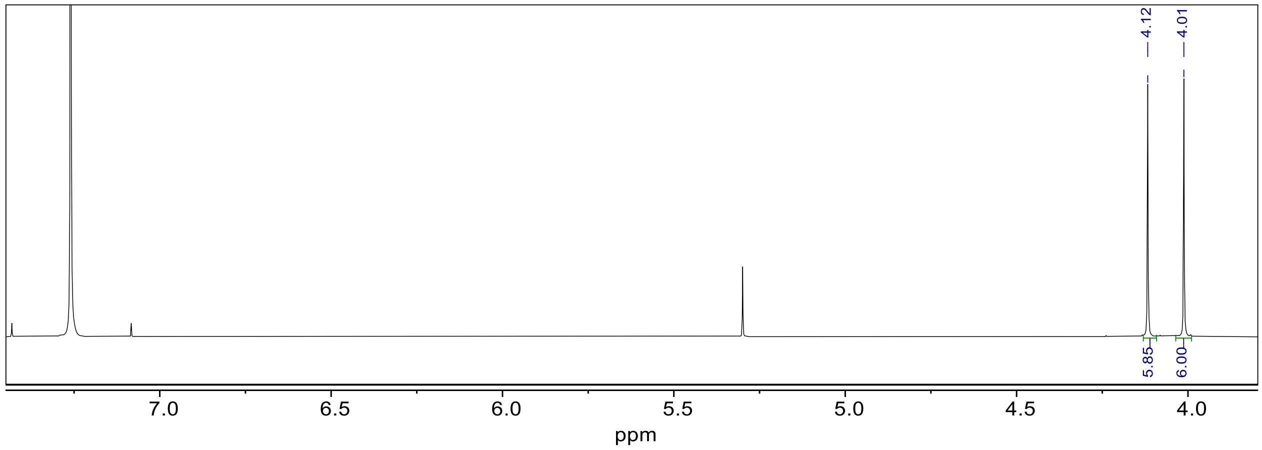

Figure S92: ^{1}H NMR (600 MHz, $CDCl_3$, 298 K) spectrum of the *trans*-3 bis-adduct **5b**.

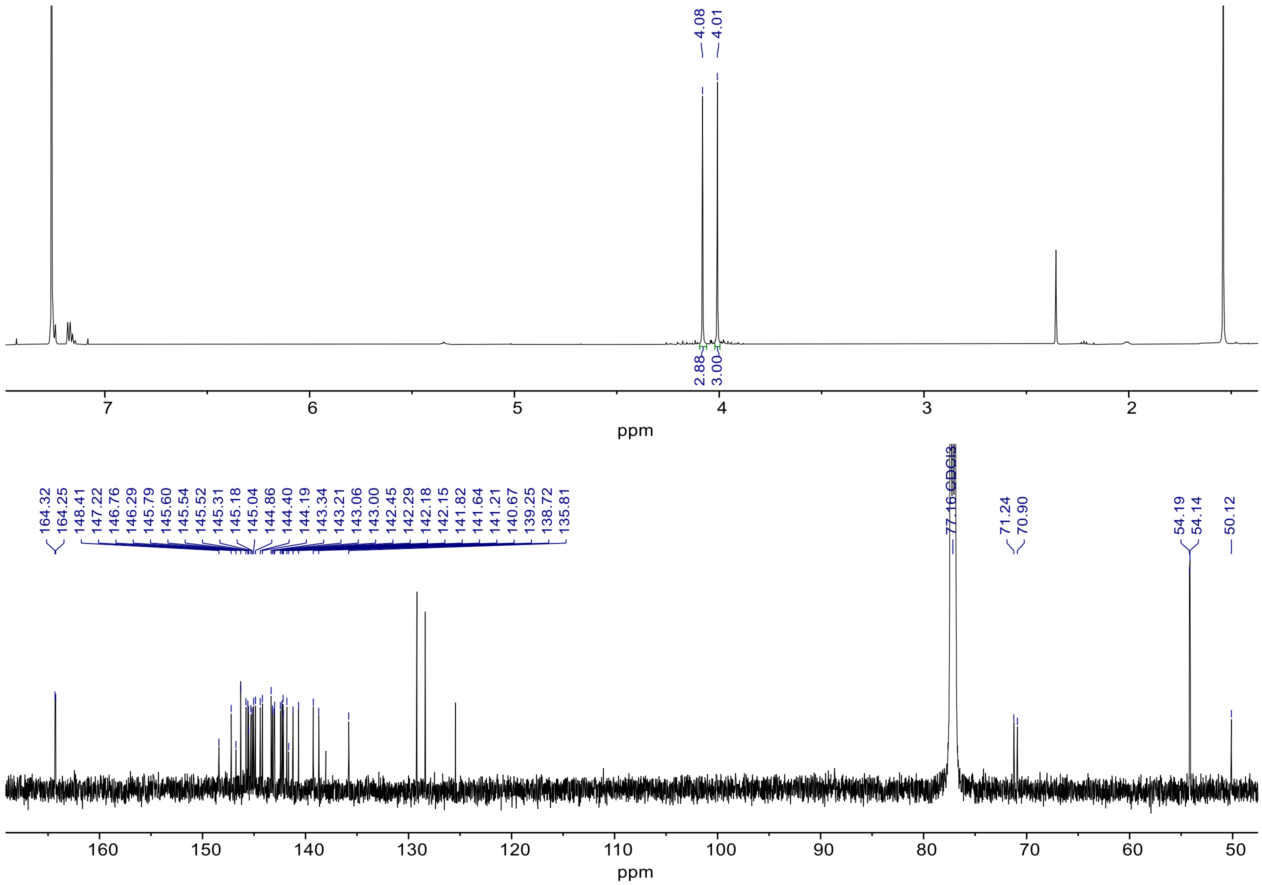

Figure S93: ^{1}H NMR (600 MHz, $CDCl_3$, 298 K) and ^{13}C NMR (151 MHz, $CDCl_3$, 298 K) spectra of the *trans*-4 bis-adduct **5c**.

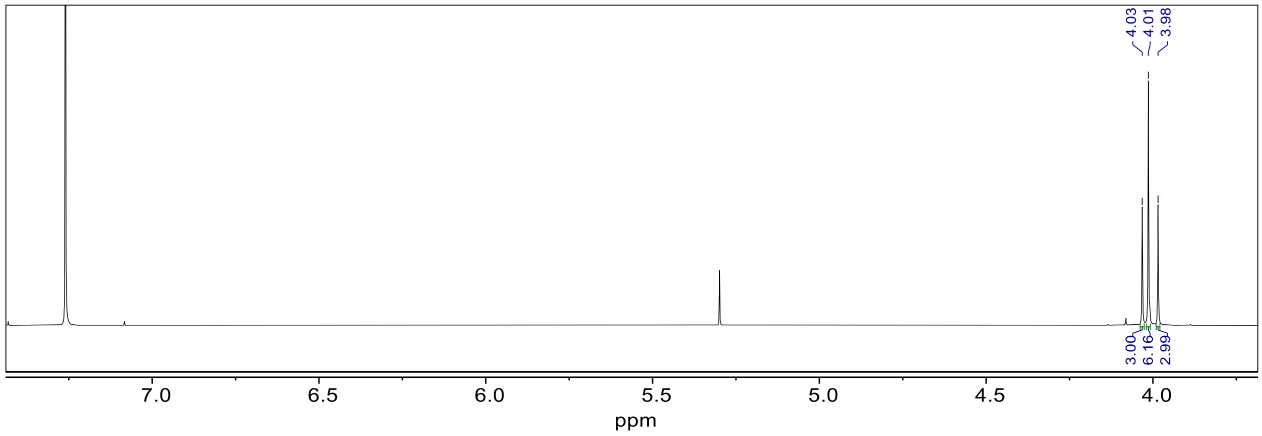

Figure S94: ^{1}H NMR (600 MHz, $CDCl_3$, 298 K) spectrum of the *equatorial* bis-adduct **5d**.

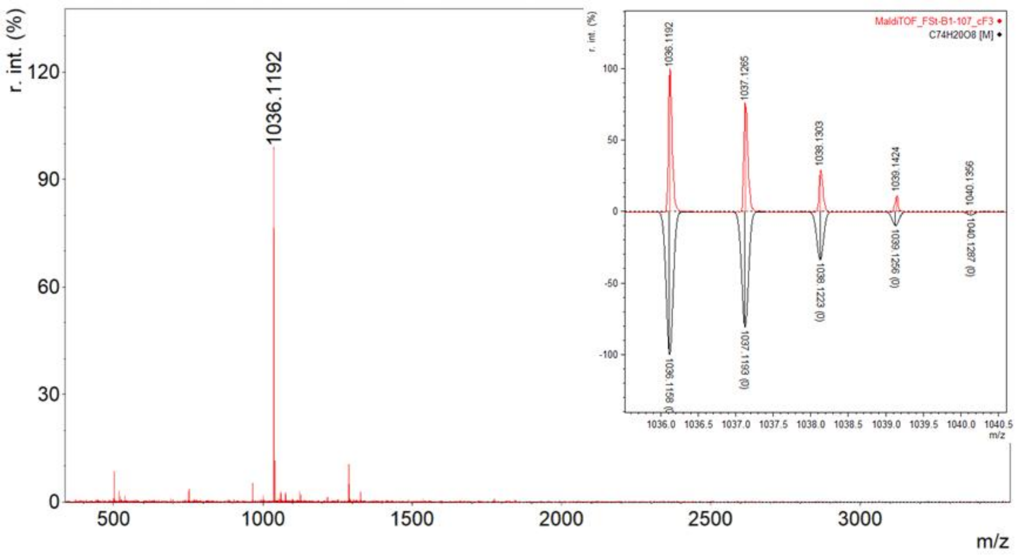

Figure S95: LRMS-MALDI-TOF spectrum of **6** and comparison with the calculated isotopic pattern (black).

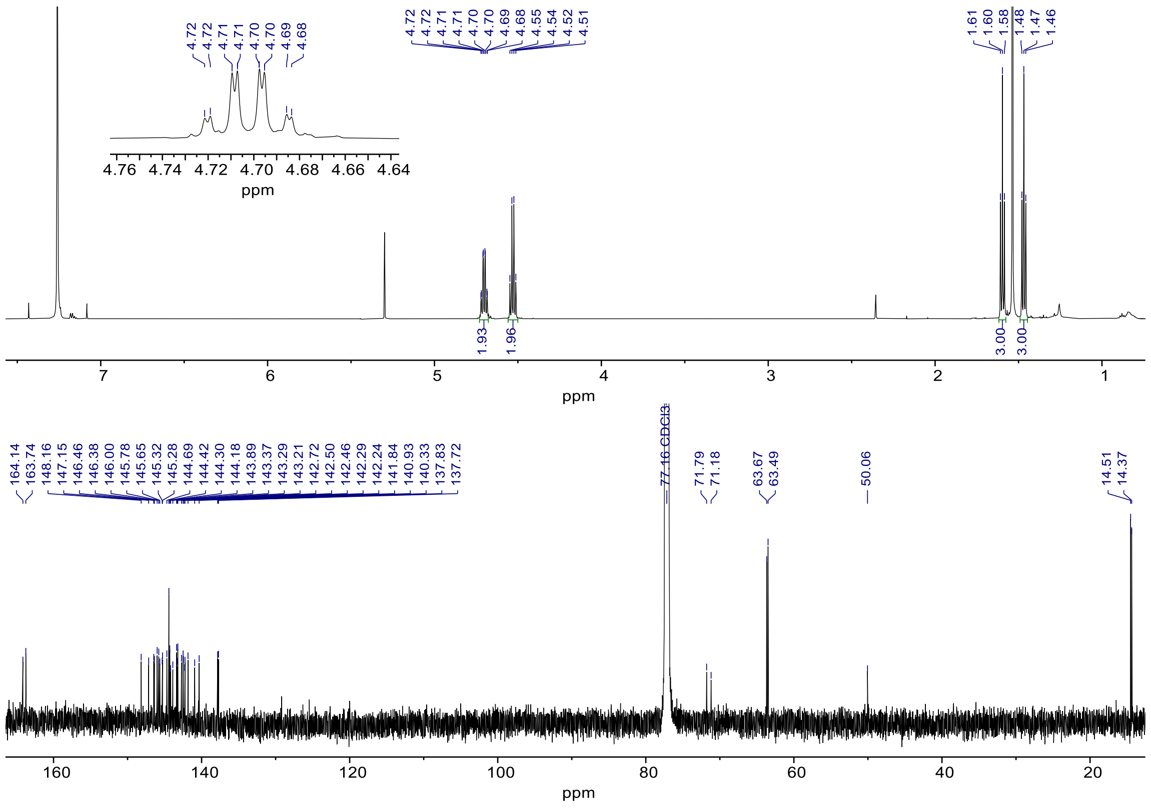

Figure S96: ^{1}H NMR (600 MHz, $CDCl_3$, 298 K) and ^{13}C NMR (151 MHz, $CDCl_3$, 298 K) spectra of the *trans*-2 bis-adduct **6a**.

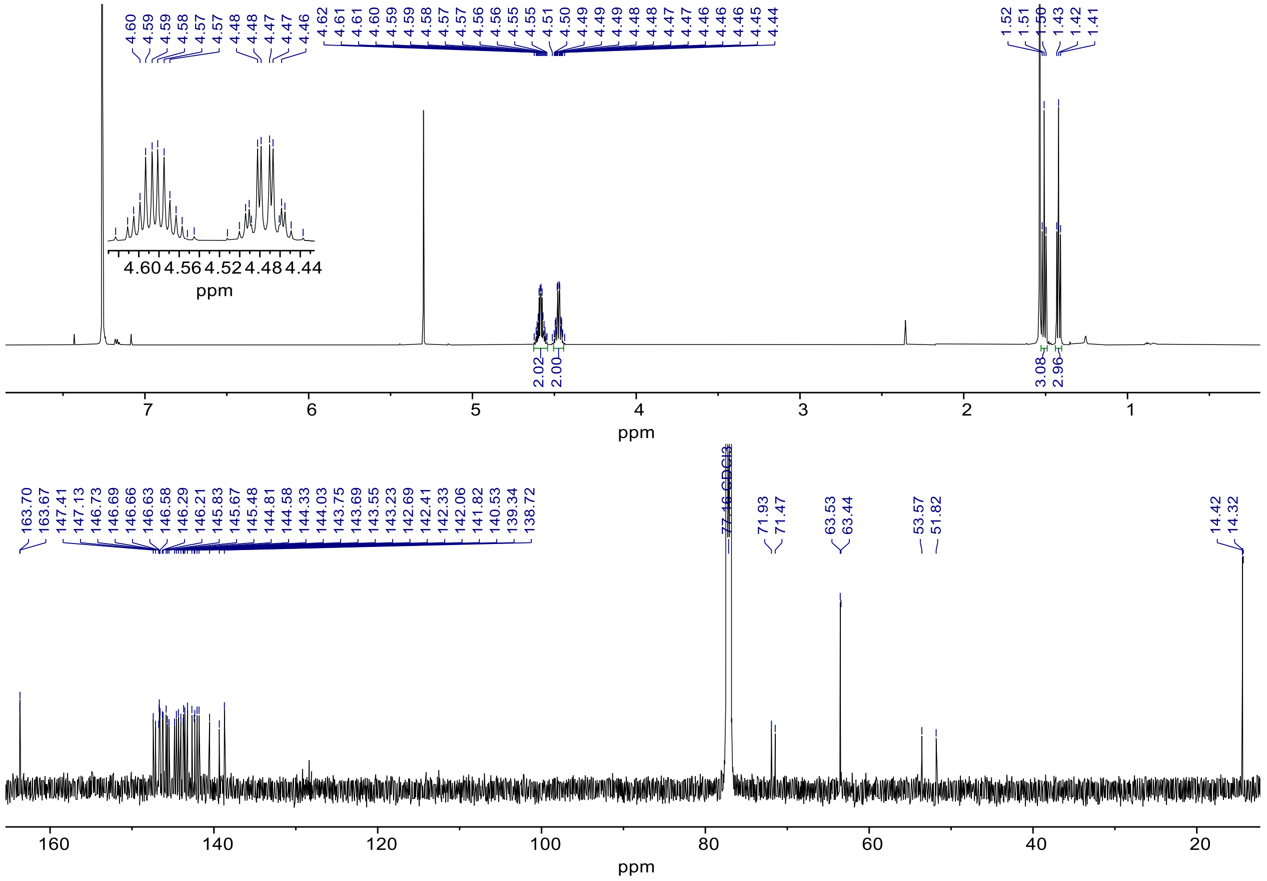

Figure S97: ^{1}H NMR (600 MHz, $CDCl_3$, 298 K) and ^{13}C NMR (151 MHz, $CDCl_3$, 298 K) spectra of the *trans*-3 bis-adduct **6b**.

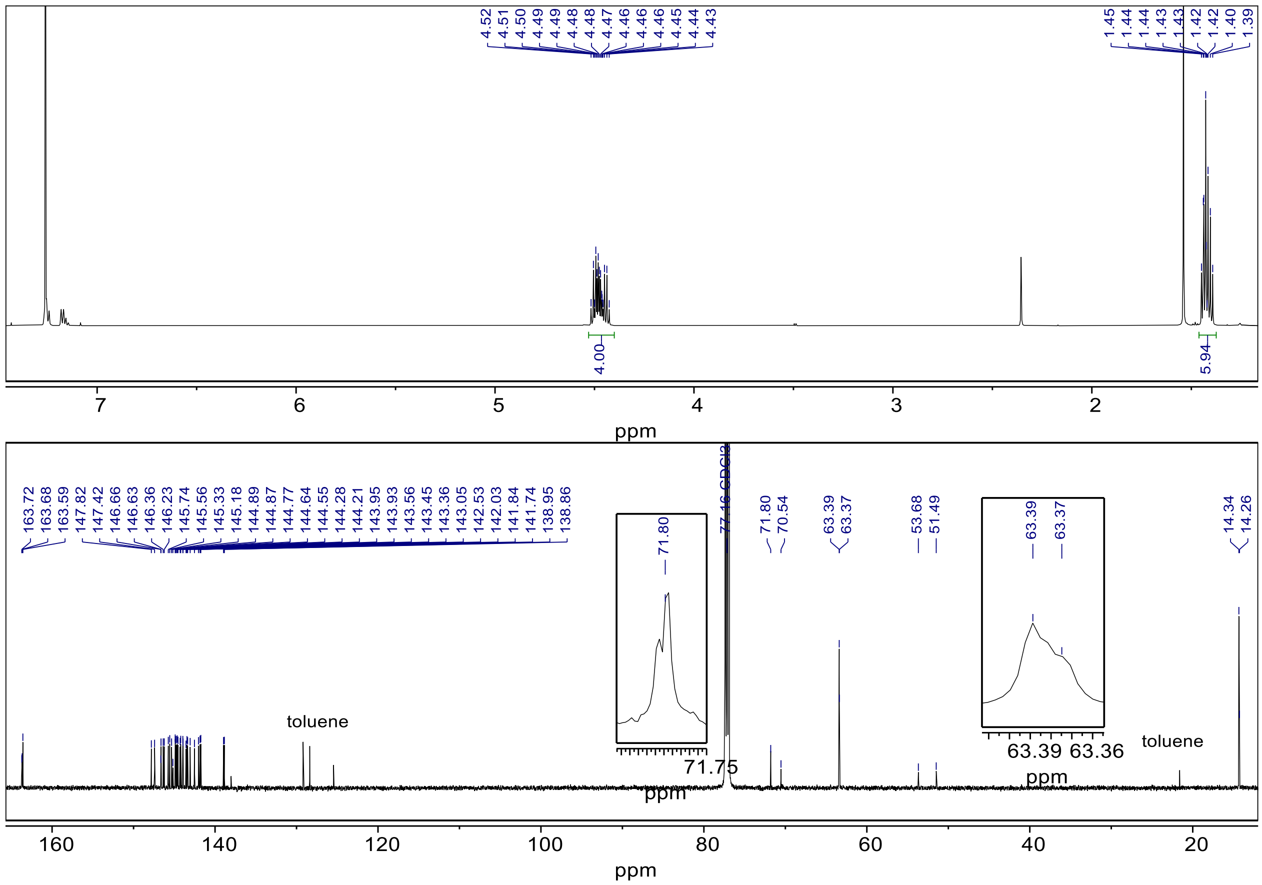

Figure S98: ^{1}H NMR (600 MHz, $CDCl_3$, 298 K) and ^{13}C NMR (151 MHz, $CDCl_3$, 298 K) spectra of the *equatorial* bis-adduct **6d**.

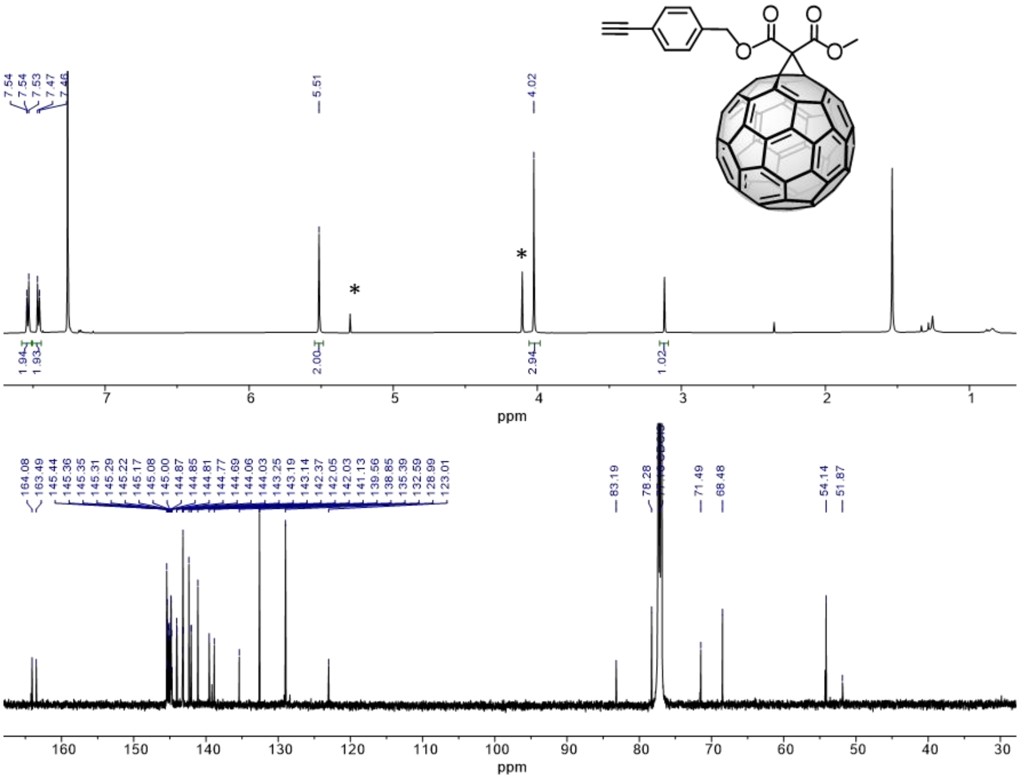

Figure S99: ^{1}H NMR (600 MHz, $CDCl_3$, 298 K) and ^{13}C NMR (151 MHz, $CDCl_3$, 298 K) spectra of C_{60} mono-adduct **7**. *Impurity from dimethyl malonate C_{60} mono-adduct (bromomalonate **2** contained dimethyl bromomalonate impurity before distillation).

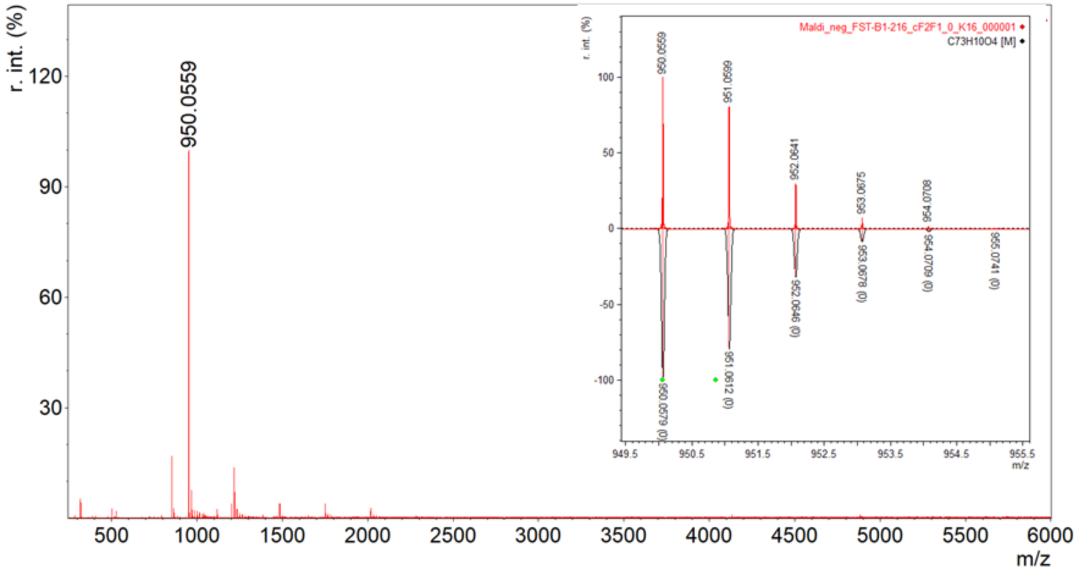

Figure S100: HRMS-MALDI spectrum of C_{60} mono-adduct **7** and comparison with the calculated isotopic pattern (black).

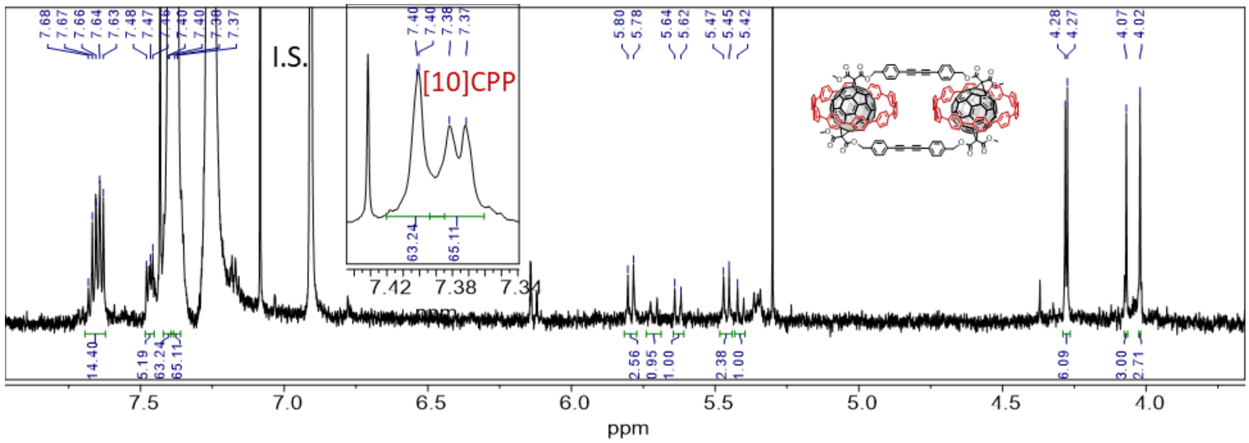

Figure S101: ^{1}H NMR (600 MHz, $CDCl_3$, 298 K) spectrum of [3]catenane **8b** Due to low concentration and overlap with impurities, assignment could only be done for selected resonances. Contains 1,2,4,5-tetramethylbenzene as internal standard (I.S.).

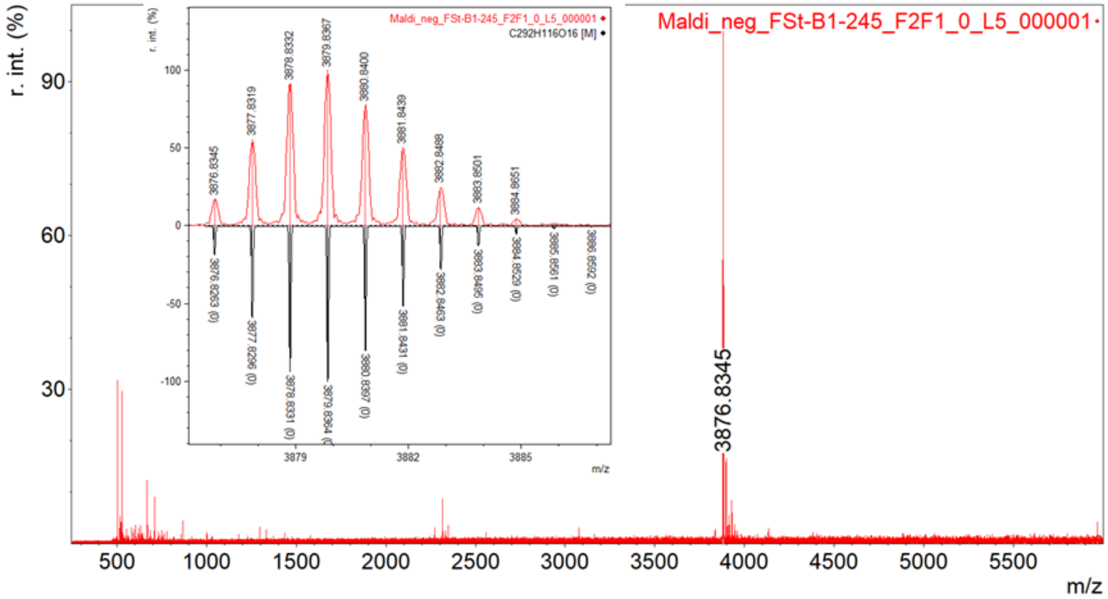

Figure S102: HRMS-MALDI spectrum of [3]catenane **8b** and comparison with the calculated isotopic pattern (black).

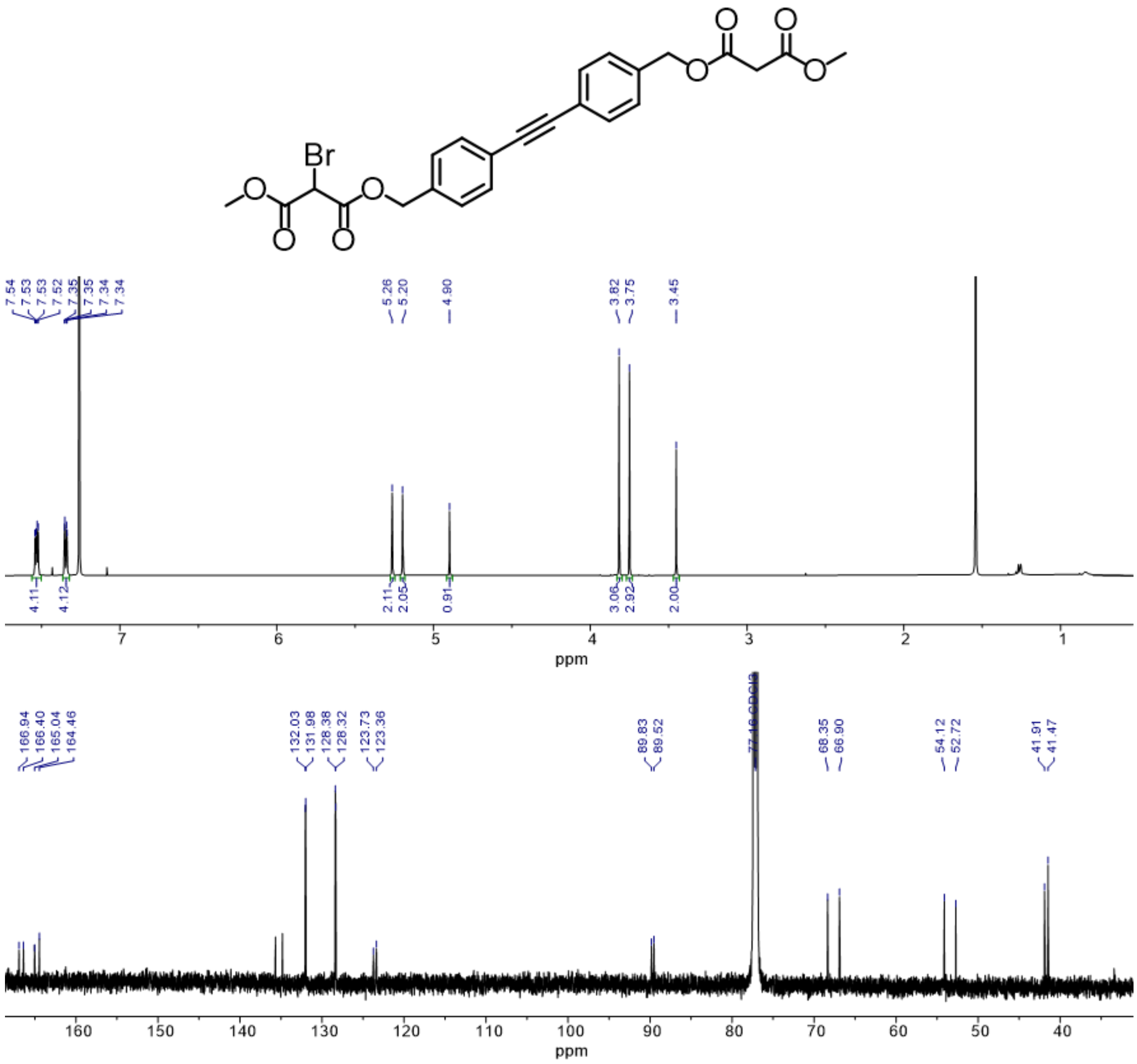

Figure S103: ^{1}H NMR (600 MHz, $CDCl_3$, 298 K) and ^{13}C NMR (151 MHz, $CDCl_3$, 298 K) spectra of the desymmetrized linker **9**.

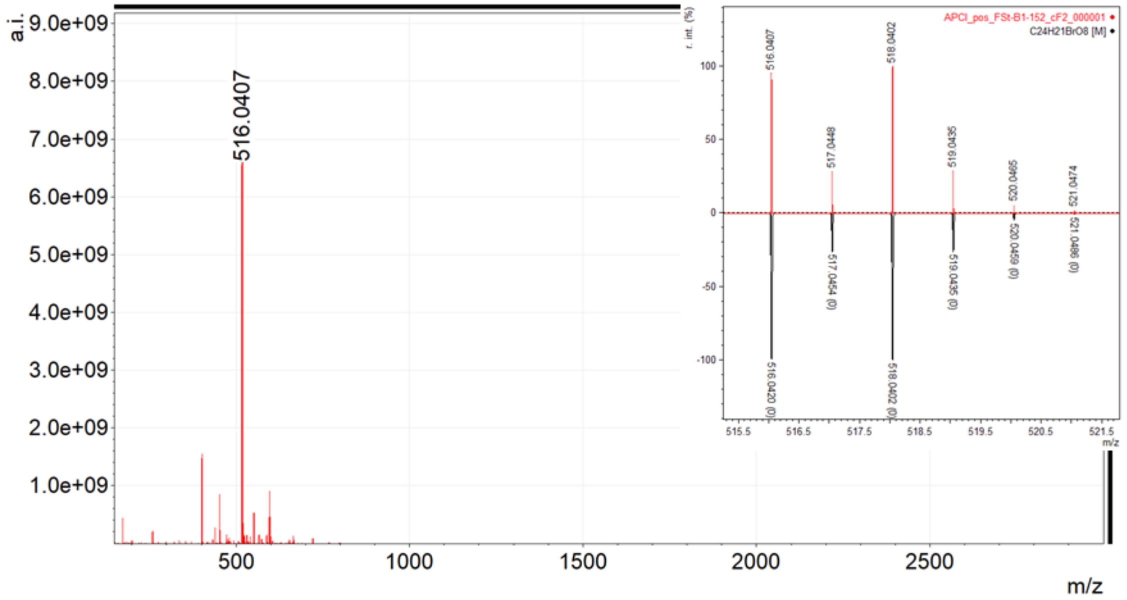

Figure S104: HRMS-APCI spectrum of desymmetrized linker **9** and comparison with the calculated isotopic pattern (black).

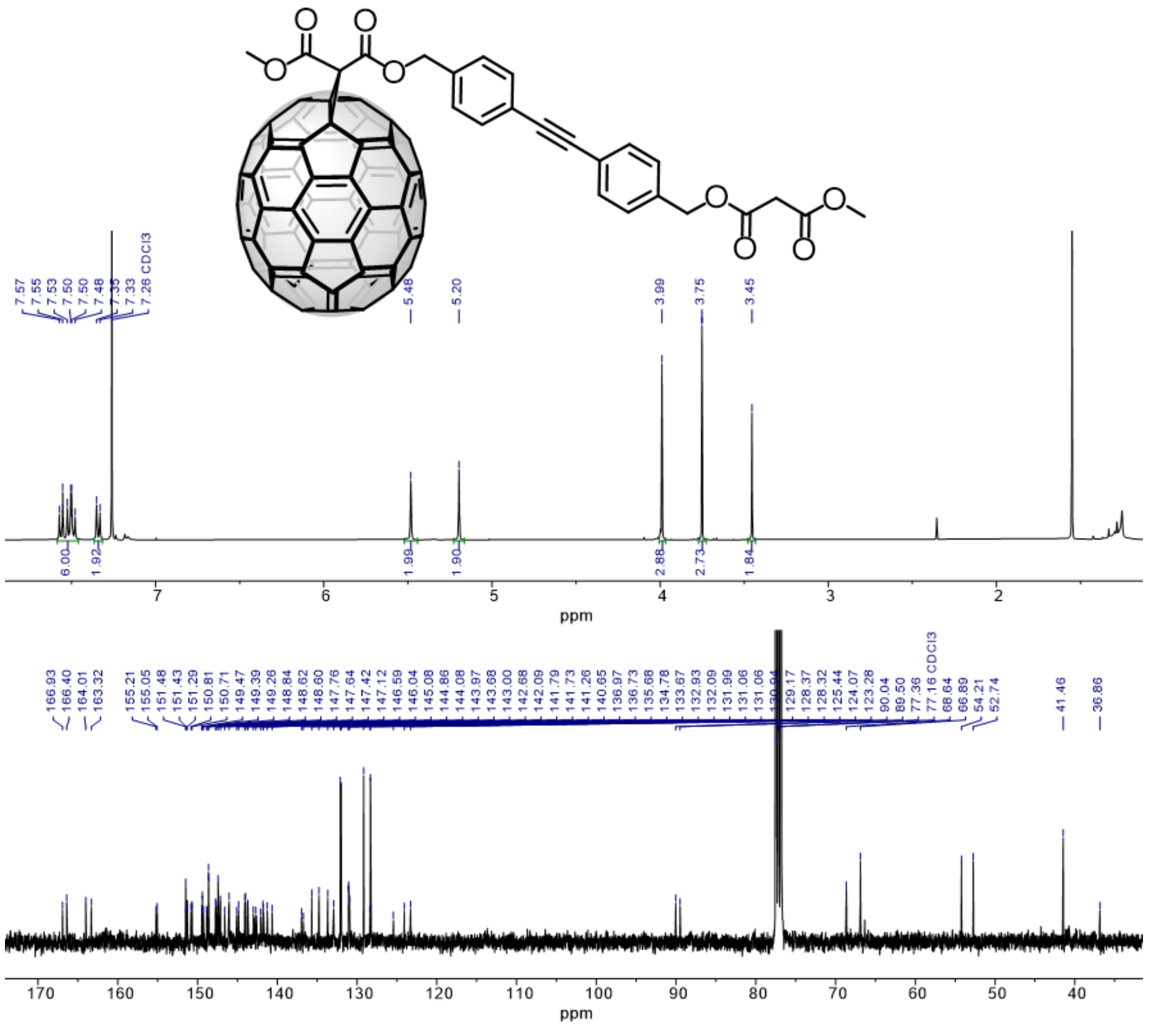

Figure S105: ^{1}H NMR (400 MHz, $CDCl_3$, 298 K) and ^{13}C NMR (101 MHz, $CDCl_3$, 298 K) spectra of C_{70} mono-adduct **10**.

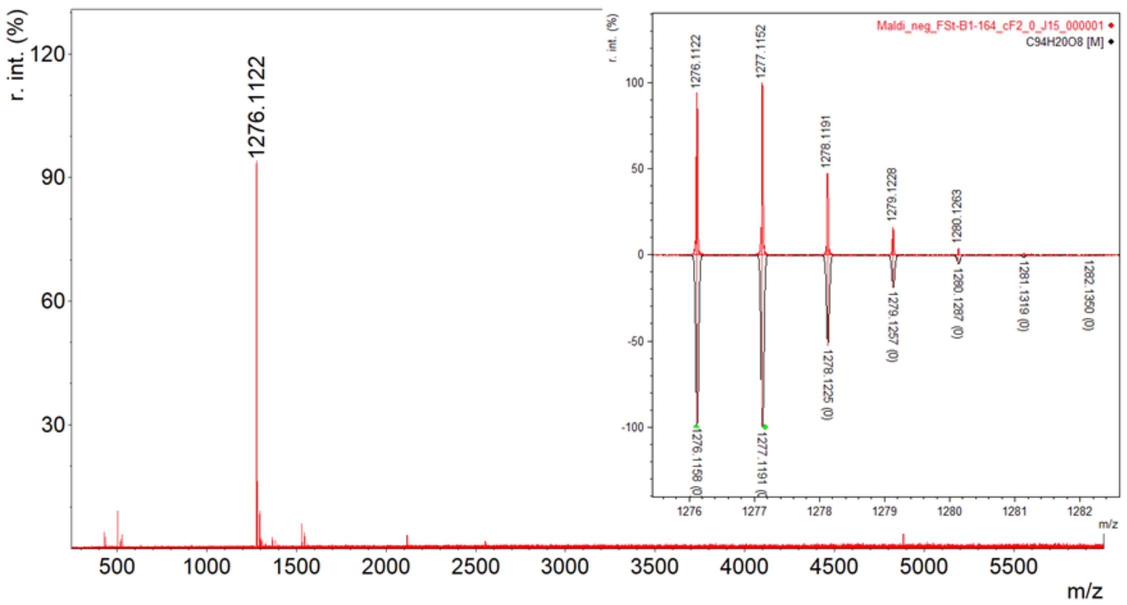

Figure S106: HRMS-MALDI spectrum of **10** and comparison with the calculated isotopic pattern (black).

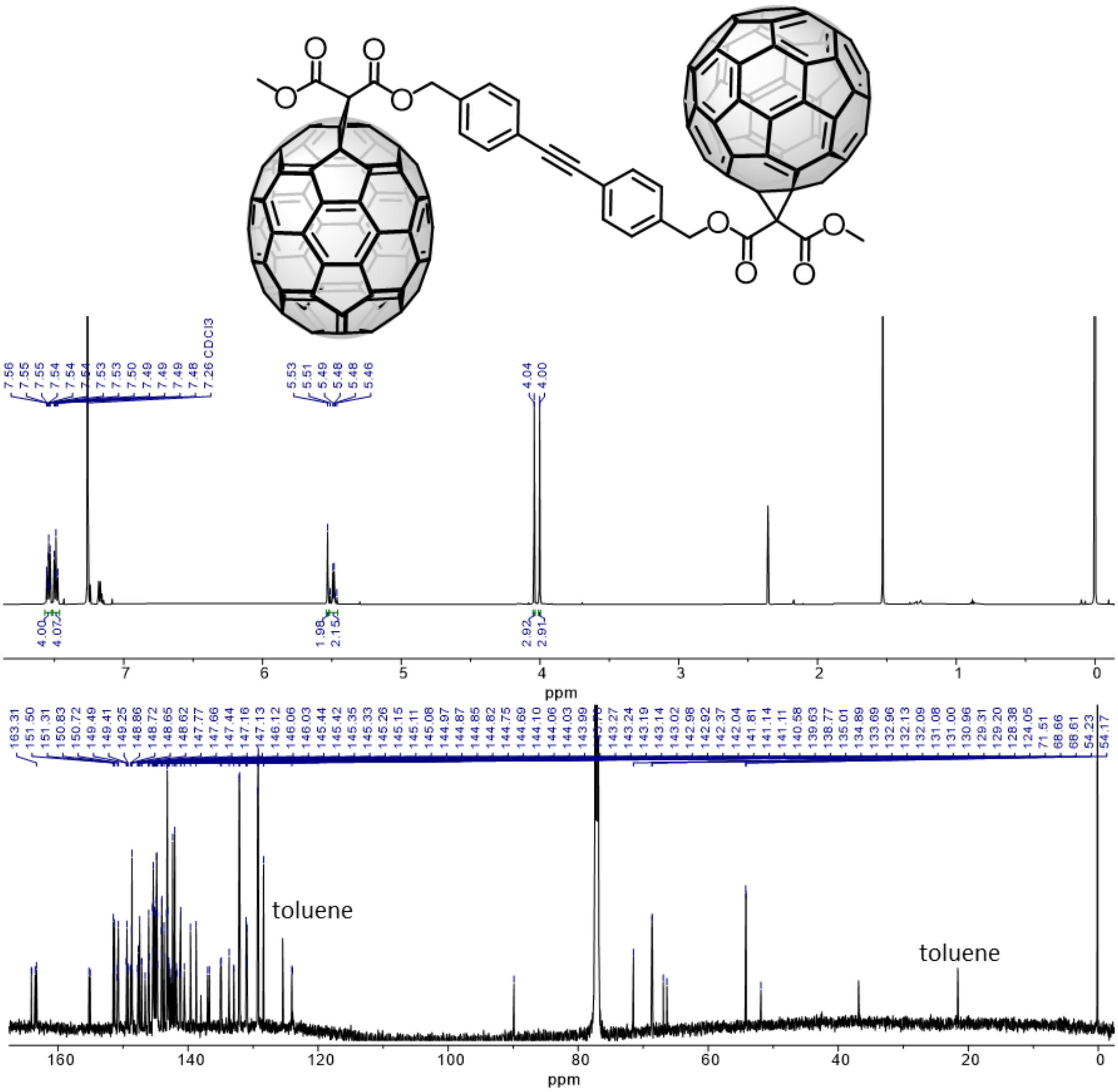

Figure S107: ^{1}H NMR (600 MHz, $CDCl_3$, 298 K) and ^{13}C UDEFT-NMR (151 MHz, $CDCl_3$, 298 K) spectra of C_{70}-C_{60} dyad **11**.

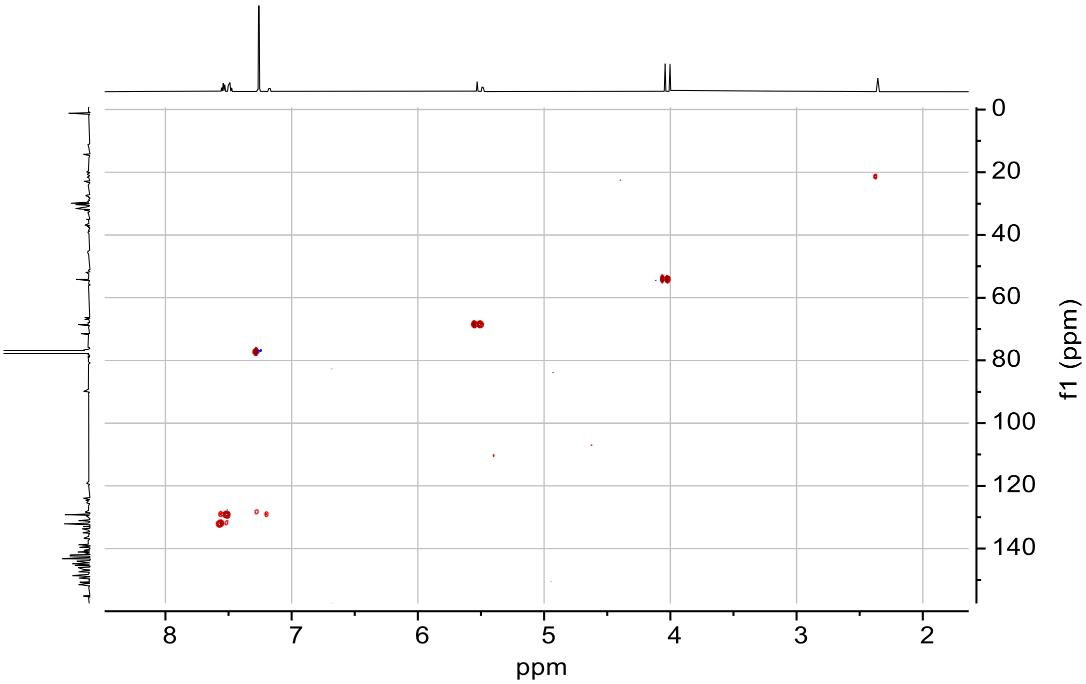

Figure S108: HSQC spectrum (^{1}H–^{13}C) of compound **11** in $CDCl_3$ at 298 K, recorded at 600 MHz (^{1}H) and 151 MHz (^{13}C).

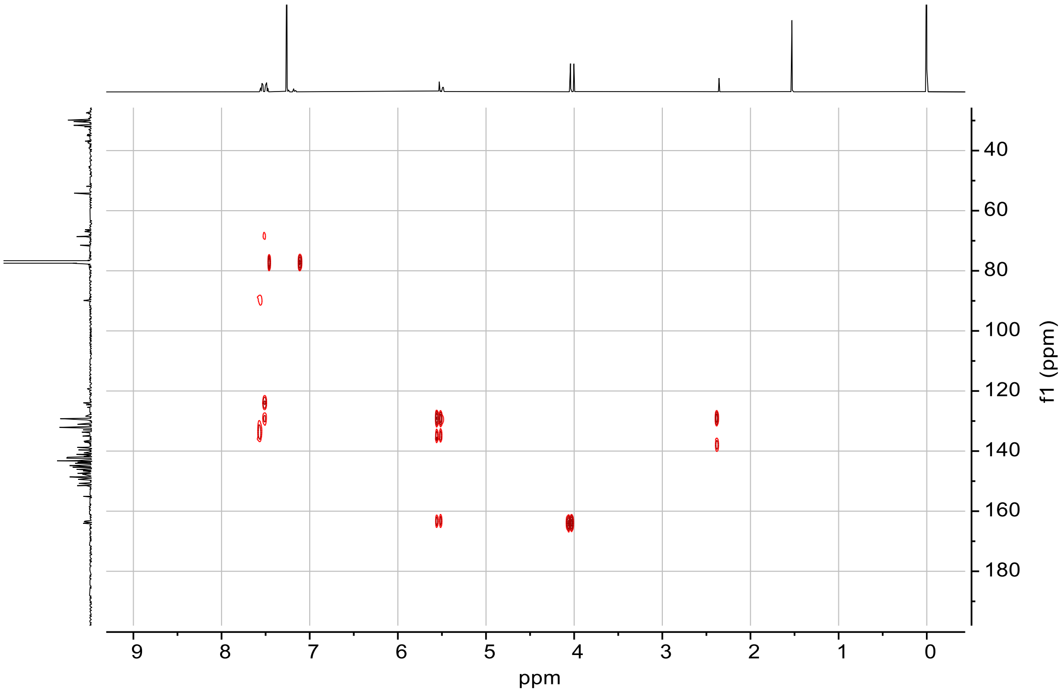

Figure S109: HMBC spectrum (^{1}H–^{13}C) of compound **11** in $CDCl_3$ at 298 K, recorded at 600 MHz (^{1}H) and 151 MHz (^{13}C).

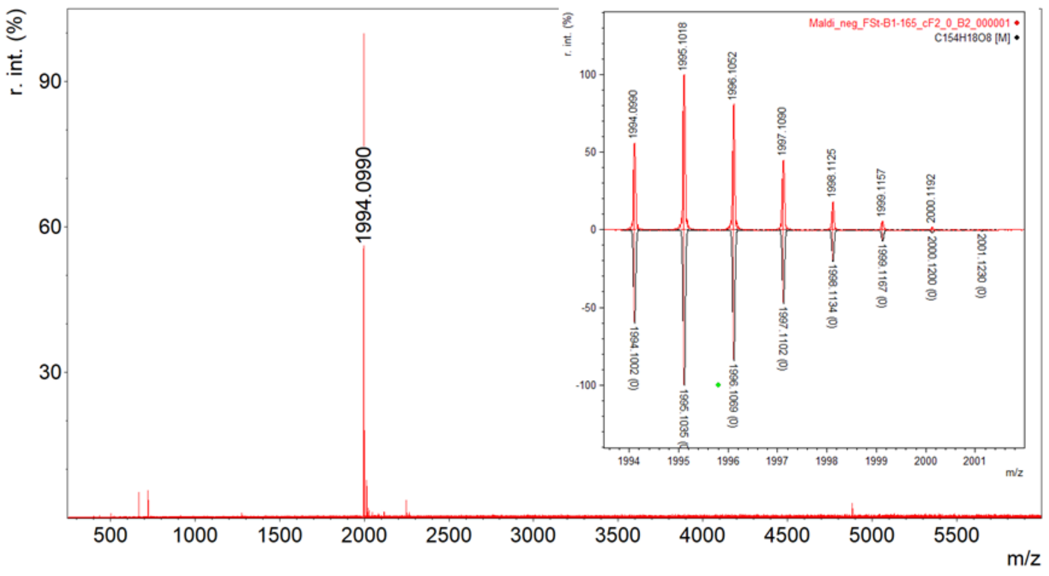

Figure S110: HRMS-MALDI spectrum of C_{70}-C_{60} dyad **11** and comparison with the calculated isotopic pattern (black).

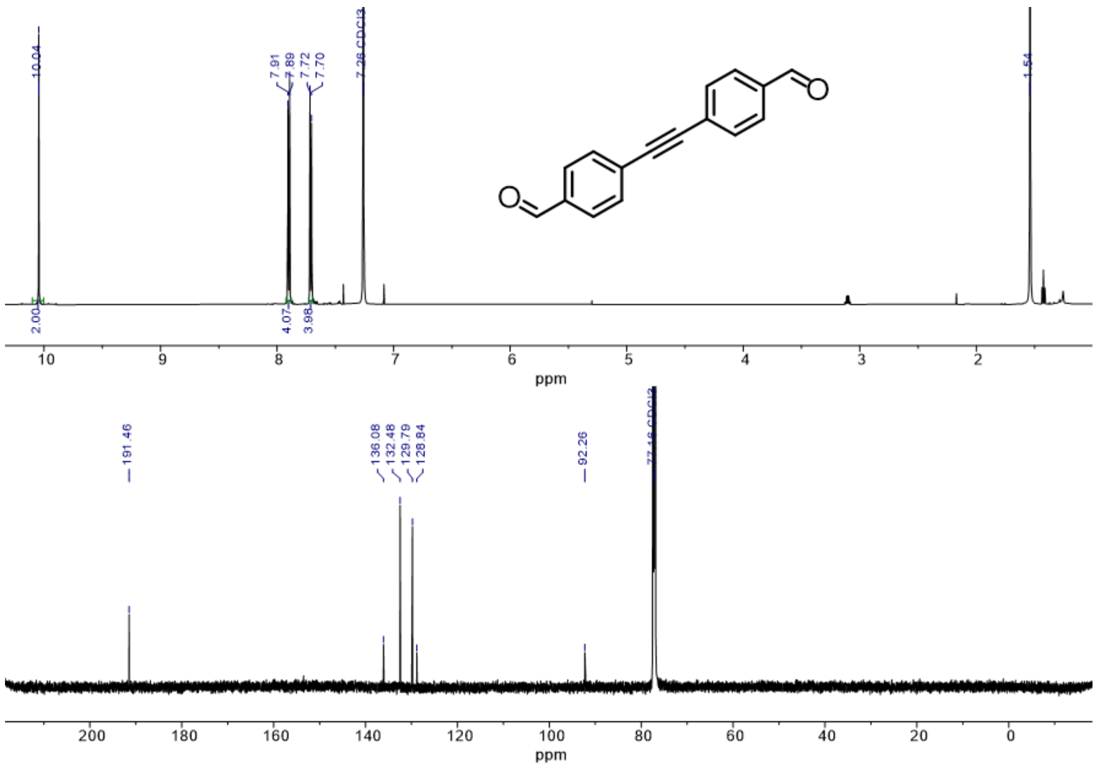

Figure S111: ^{1}H NMR (600 MHz, $CDCl_3$, 298 K) and ^{13}C NMR (151 MHz, $CDCl_3$, 298 K) spectra of dialdehyde **12**.

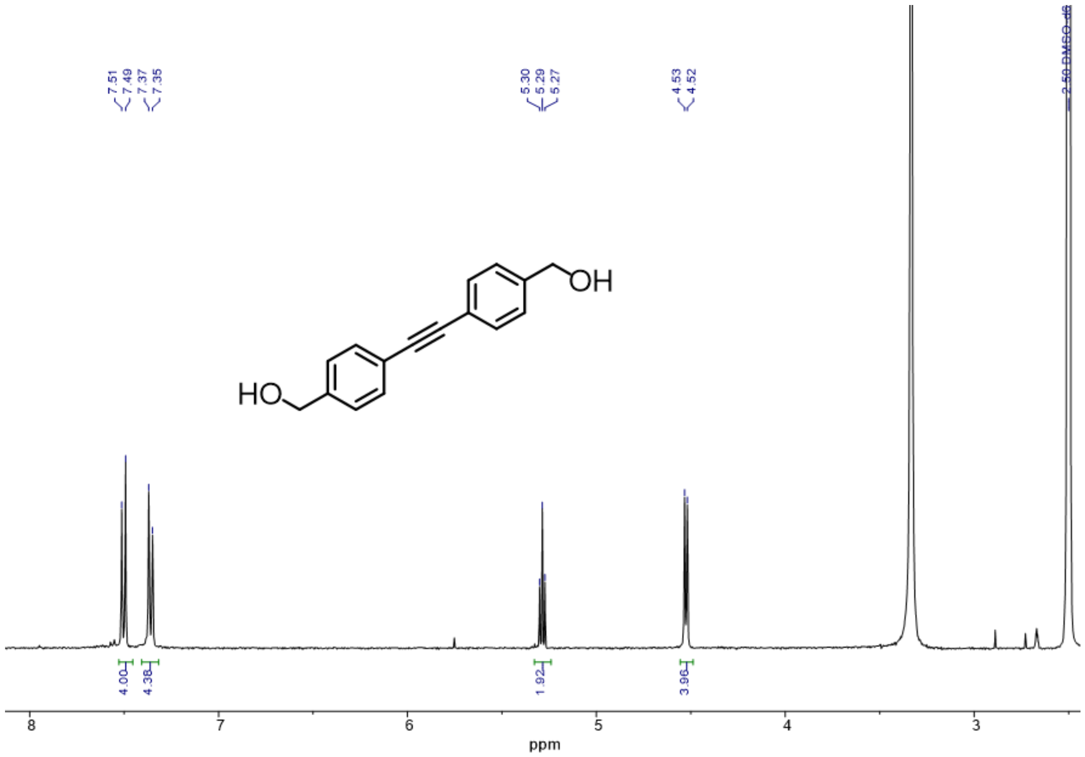

Figure S112: ^{1}H NMR (400 MHz, $CDCl_3$, 298 K) spectrum of the diol **13**.

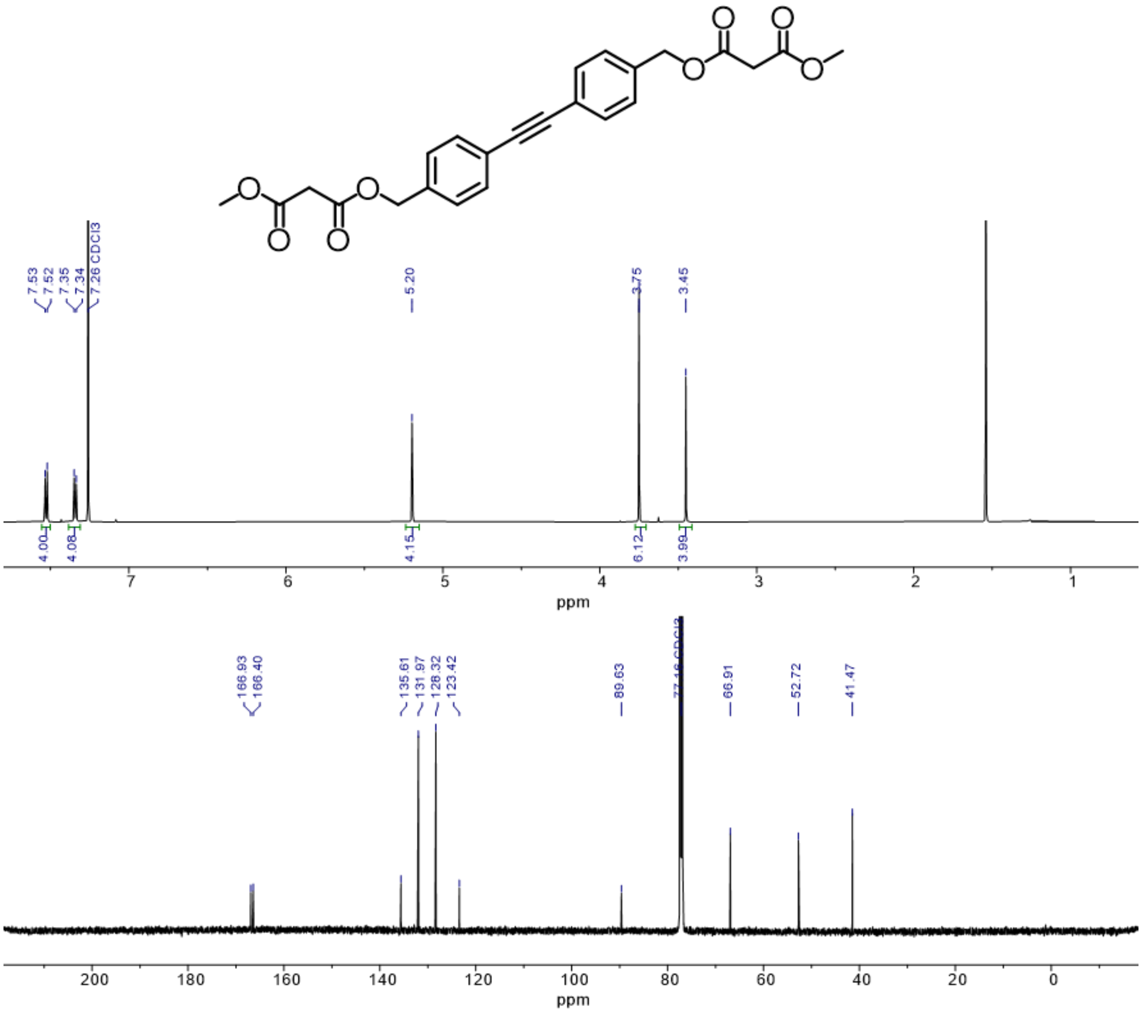

Figure S113: ^{1}H NMR (600 MHz, $CDCl_3$, 298 K) and ^{13}C NMR (151 MHz, $CDCl_3$, 298 K) spectra of the dimalonate **14**.

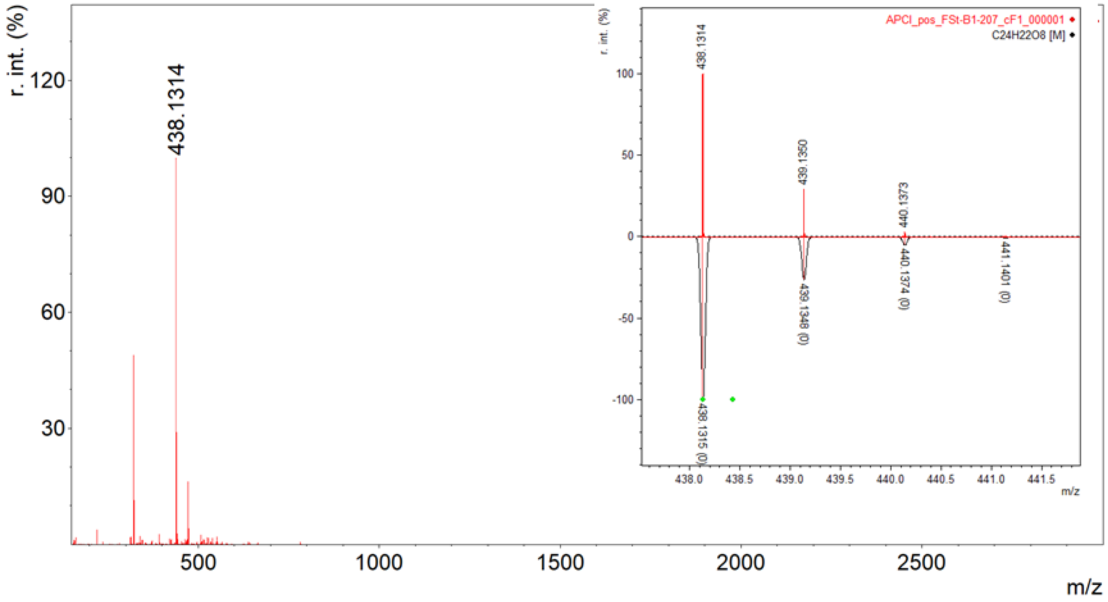

Figure S114: HRMS-APCI spectrum of dimalonate **14** and comparison with the calculated isotopic pattern (black).

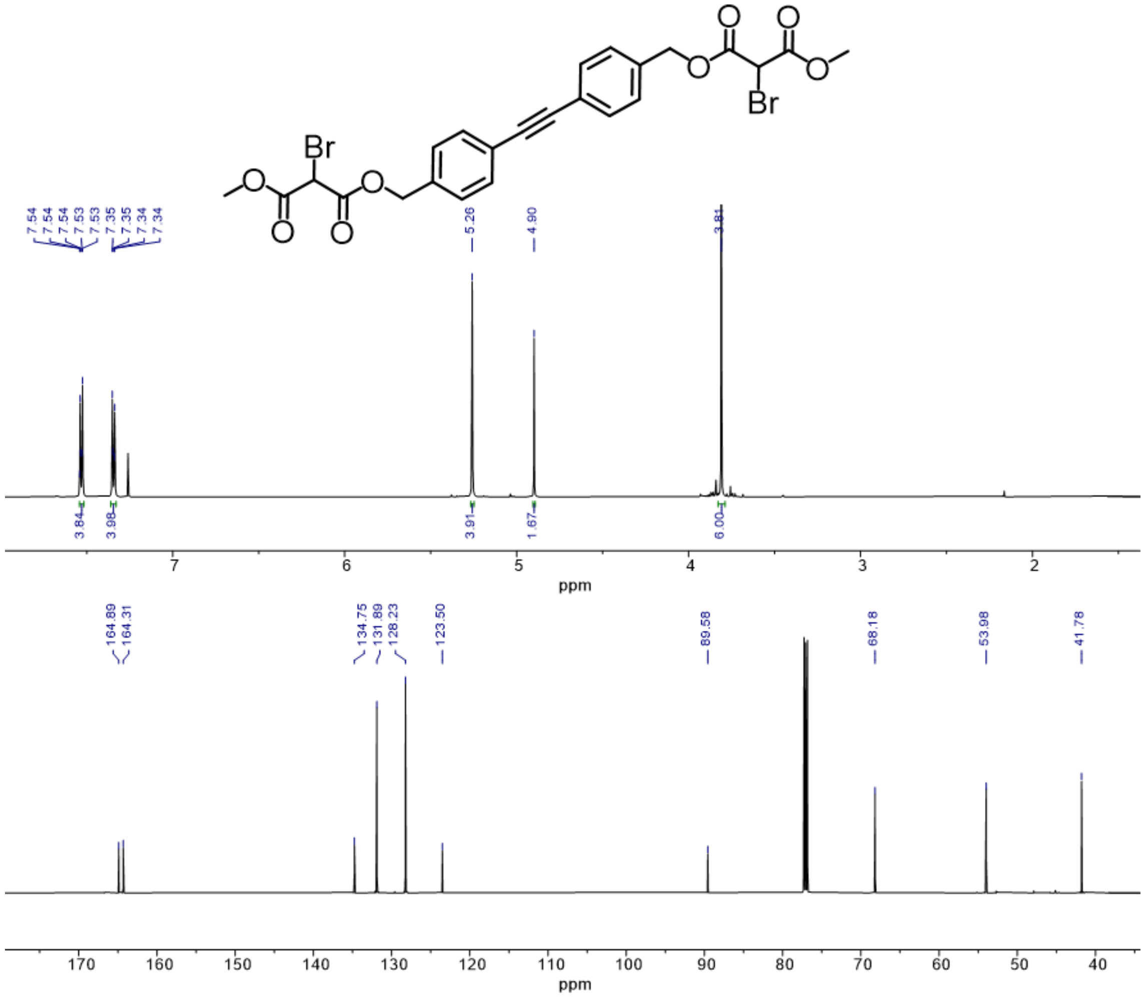

Figure S115: ^{1}H NMR (600 MHz, $CDCl_3$, 298 K) and ^{13}C NMR (151 MHz, $CDCl_3$, 298 K) spectra of the bis-bromomalonate **15**.

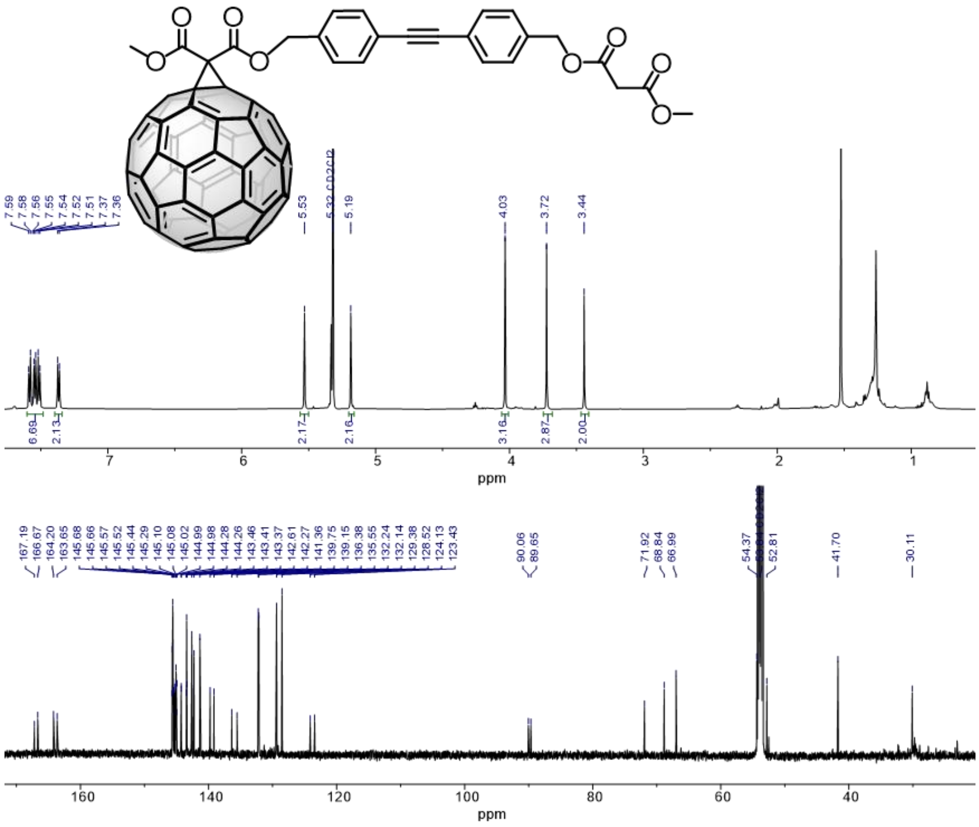

Figure S116: ^{1}H NMR (600 MHz, CD_2Cl_2, 298 K) and ^{13}C NMR (151 MHz, CD_2Cl_2, 298 K) spectra of C_{60} mono-adduct **16**.

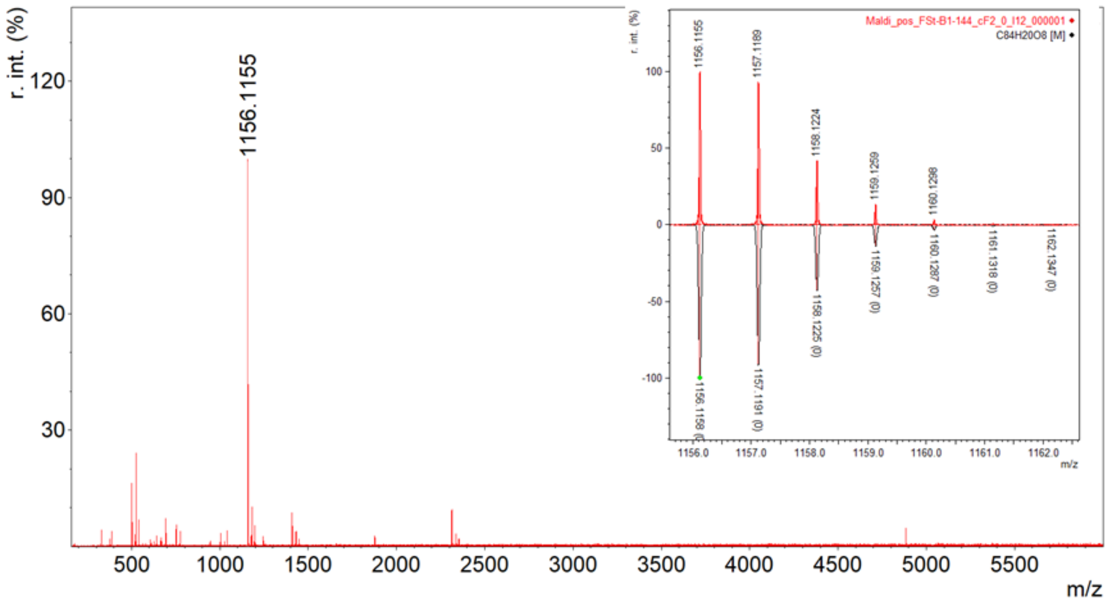

Figure S117: HRMS-MALDI spectrum of **16** and comparison with the calculated isotopic pattern (black).

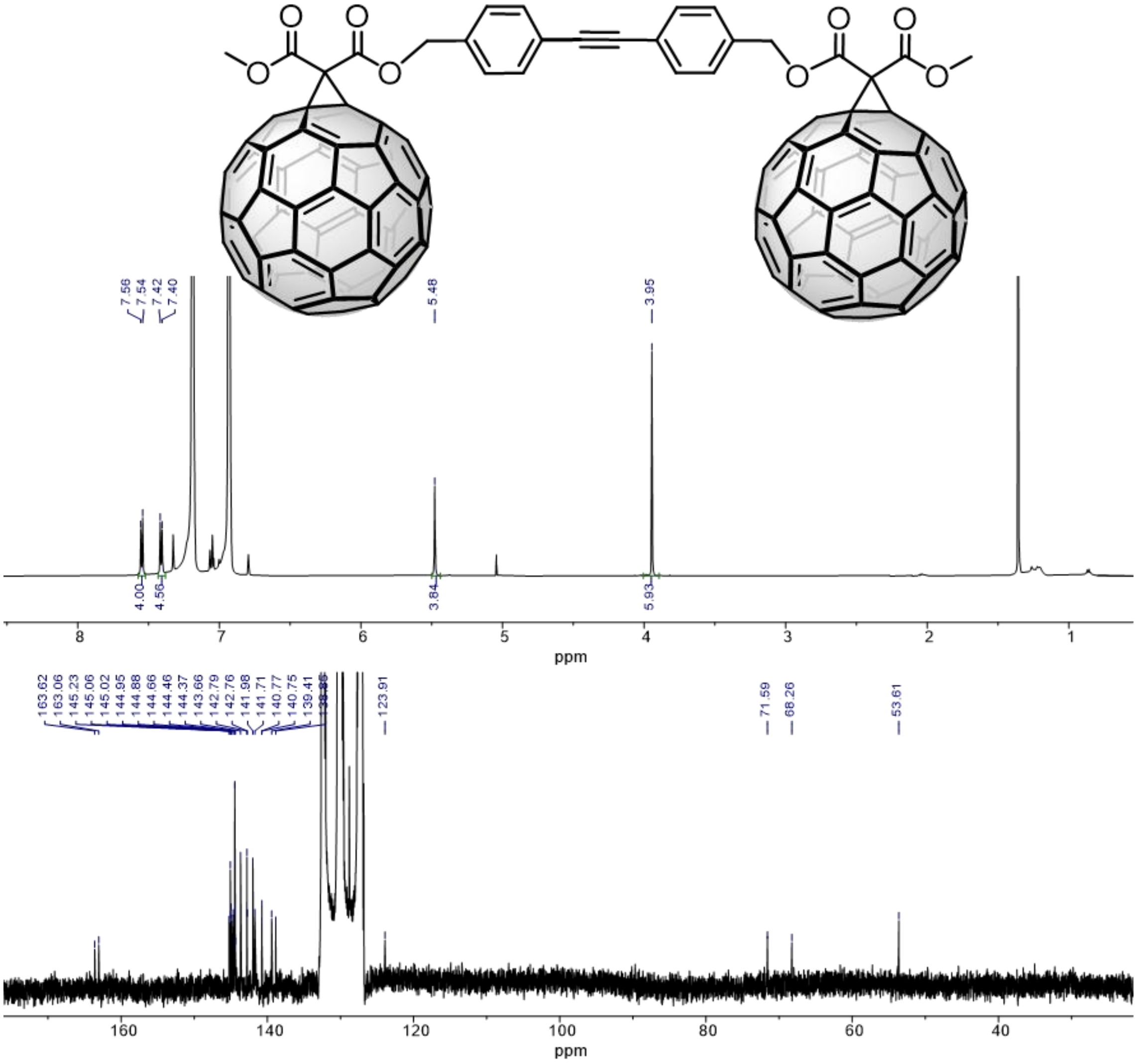

Figure S118: ^{1}H NMR (600 MHz, *o*-DCB-d_4, 298 K) and ^{13}C NMR (151 MHz, *o*-DCB-d_4, 298 K) spectra of dumbbell **17**.

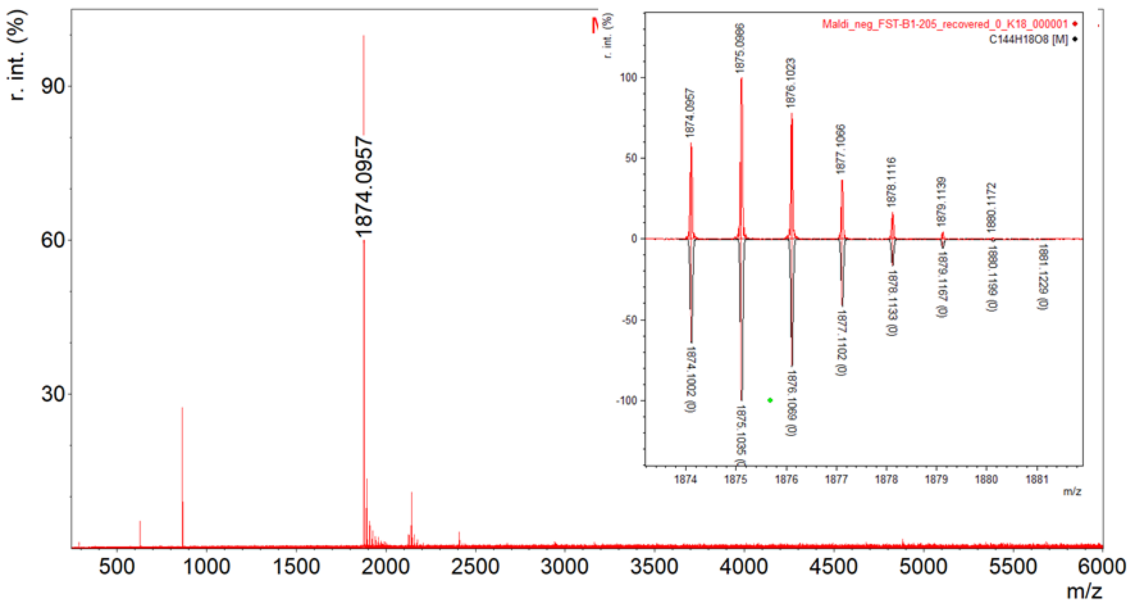

Figure S119: HRMS-MALDI spectrum of **17** and comparison with the calculated isotopic pattern (black).
